# A taxonomic review of the pericaline ground-beetles in Taiwan, with descriptions of new species (Coleoptera, Carabidae, Lebiini)

**DOI:** 10.3897/zookeys.816.29738

**Published:** 2019-01-14

**Authors:** Wesley Hunting, Man-Miao Yang

**Affiliations:** 1 Department of Entomology, National Chung Hsing University, Taichung, 40227 Taiwan National Chung Hsing University Taichung Taiwan

**Keywords:** Asia, Carabidae, Lebiini, new genus, new species, Pericalina, Taiwan

## Abstract

A taxonomic review of the known Taiwanese taxa of the pericaline Lebiini, this paper includes a key to the genera, keys to species, descriptions, and redescriptions of all species, illustrations, geographic range maps, re-rankings, and new synonymies. In total 34 species are treated, nine of which are described as new. A new genus and new species are as follows: *Bellavalentis***gen. n.**, (type species *Dolichoctiskuzugamii* Shibata, 1967); *Amphimenesabsensacidus***sp. n.**; *Amphimenesbeichatiensis***sp. n.**; *Amphimenescarinacaulis***sp. n.**; Catascopus(s. str.)asaharti**sp. n.**; Catascopus(s. str.)viridiorchis**sp. n.**; Coptodera (Coptoderina) occulta**sp. n.**; *Dolichoctisbadiadorsis***sp. n.**; *Dolichoctisdilatata***sp. n.**; *Moctherusobscurabasis***sp. n.** After close examination of the type material of several species, we were able to determine that *Coptoderinachaudoirianguilipennis* (Nakane and Okhura) is a junior synonym of Coptodera (Coptoderina) chaudoiri Andrewes, **syn. n.** and *Coptoderanobilis* Jedlička is also a junior synonym of *C.chaudoiri*, **syn. n.***Dolichoctisstriatusformosanus* Habu is a junior synonym of *Dolichoctisrotundata* (Schmidt-Goebel), **syn. n.**Dolichoctis (Mochtherus) uenoi Habu is a junior synonym of *Mochtherusluctuosus* Putzeys, **syn. n.***Pericalusformosanus* Dupuis was recently ranked as a subspecies of *Pericalusornatusformosanus* Dupuis. After consideration of the several consistent taxonomic characteristics and also considering its allopatric distribution with all other species of *Pericalus*, we believe *Pericalusformosanus* Dupuis to be a valid species, **stat. resurr.** The monobasic genus *Pseudomenarus* (type species *Pseudomenarusflavomaculatus* Shibata, 1964) is established as conspecific with members of the genus *Formosiella* Jedlička, 1951, **comb. n.**

Species previously recorded from Taiwan that are not present here include: *Amphimenespiceolus* Bates; *Catascopusaequatus* Dejean; *Catascopusfacialis* (Wiedemann) *Coptoderainterrupta* Schmidt-Goebel *Coptoderaflexuosa* Schmidt-Goebel and *Peripristusater* (Laporte). The pericaline taxa of Taiwan are arranged in 14 genera, five subgenera, and 34 currently known species. Notes on collecting circumstances, habits, and habitat are included when known.

## Introduction

During the past 130 years, few researchers have studied the ground-beetle fauna of Taiwan. Previous major taxonomic contributions include those of Miwa (1931), Jedlička (1932, 1940, 1963), Habu (1967, 1978), Terada et al. 2005, and Terada 2006. Of these contributions, those restricted to Taiwan (Miwa and Terada et al.) are merely lists of taxon names. Various other, less extensive publications deal with elements of the Taiwanese pericaline fauna though most of these only include a species description of a specific taxon and are often not restricted to Taiwan in scope. The latest checklist of Carabidae from Taiwan has a total of 467 species belonging to 34 tribes have been described to date but acknowledges that additional taxa still await discovery in the rich landscape of Taiwan (Terada 2006). In that checklist there were 28 species of the subtribe Pericalina recorded.

After visiting all of the major carabid collections in Taiwan, in became clear that informed taxonomic work based on already collected specimens would be impossible. This was due to an inadequate numbers of specimens as well as a lack of a full and reliable reference collection. Reference collections that did exist were often based on damaged and/or misidentified material^1^. In fact, we estimate that fewer than half of all species of pericalines (and also carabids in general) recorded from Taiwan, existed in any Taiwanese collection. To add to the uncertainty, many of what would be considered important and valuable carabid specimens held at the Taiwan Agricultural Research Institute (previously the largest carabid holding in Taiwan) are erroneously labeled as cotypes and also misidentified at the species level. The material was examined and these specimens are not at all associated with the type material or the authors who described them. To exacerbate this, all label data have been stripped from them and all that remains is a small, circular paper that declares “cotype” and an additional small label with a reference number to a book that has been irretrievable since the death of the person who “organized” the collection. Sadly, we have concluded that the vast majority of carabid material with a “cotype” label from TARI is of no taxonomic value.

And so, in an effort to increase collections and better know the Taiwan fauna, we collected for the next three years (2011–2014) using several methods that included u.v. light, m.v. light, sweep netting, malaise trapping, pitfall trapping, sugar baits painted on tree trunks, hand collecting, and insecticidal fogging. Over the three-year period, we were able to substantially increase the collections of the Taiwan Carabidae. Also as a result of this fieldwork, a much better understanding of the pericalines represented in Taiwan was developed. This paper describes or re-describes 34 pericaline species (nine new), from 14 genera (one new), and five subgenera. Four new synonymies, one new combination, and one status resurrection are also recognized. Through this work we were also able to determine that some of the pericaline species previously recorded from Taiwan were misidentified by previous authors and are in fact not present here. These species include *Amphimenespiceolus* Bates, *Catascopusaequatus* Dejean, *Catascopusfacialis* (Wiedemann), *Coptoderainterrupta* Schmidt-Goebel, *Coptoderaflexuosa* Schmidt-Goebel, and *Peripristusater* (Laporte).

## Materials and methods

This revision is based on the study of more than 1600 adult specimens representing 34 taxa belonging to the subtribe Pericalina. Specimens of many carabid taxa were collected from 2011 to 2014 and are housed at the entomology museum of National Chung Hsing University, Taichung, Taiwan (NCHU). Additional adult specimens were borrowed from the collections of various individuals and institutions listed below, along with a four-letter or five-letter coden (Arnett et al. 1993) to identify sources of specimens. Names in parentheses below, indicate curator of collection.

**CMNH** Section of Invertebrate Zoology, Carnegie Museum of Natural History, 4400 Forbes Avenue, Pittsburgh, Pennsylvania, U.S.A. 15213- 4080 (RL Davidson)

**NCHU** Department of Entomology, National Chung Hsing University, Taichung City 402, Taiwan (Man-Miao Yang)

**NHNM** Department of Life Sciences (Entomology), Natural History Museum, Cromwell Road, London, SW7 5BD, UK (Beulah Garner)

**NIAES**National Institute for Agro-Environmental Sciences, Kannondai 3-1-3, Tsukuba, Ibaraki, 305-8604, Japan (H Yoshitake)

**NMNS**National Museum of Natural Science, One Guancian Road Taichung City 404, Taiwan (Jing-Fu Tsai)

**NMPC**National Museum, Entomology Department Cirkusová 1740, 193 00, Praha 9 - Horní Počernice, Czech Republic (Lukáš Sekerka)

**NSMT**National Science Museum, Department of Zoology, Hyakunin-cho 3-23-1, Shinjuku-ku, Tokyo, 169-0073, Japan (Shuhei Nomura)

**OMNH**Osaka Museum of Natural History, Nagai Park, Higashi-sumiyoshi-ku, Osaka, 546-0034, Japan (Shigehiko Shiyake)

**SDEI**German Entomological Institute Eberswalder Straße 90, 15374, Müncheberg (Stephan Blank)

**TARI** Insect Collection of the Taiwan Agricultural Research Institute, Wufeng District, Taichung City 41362, Taiwan (Chi-Feng Lee)

**TFRI**Taiwan Forestry Research Institute, No. 53, Nan-Hai Road, Taipei, Taiwan, ROC (JTChao)

**UASM** E.H. Strickland Entomology Museum, University of Alberta, Edmonton, Alberta, Canada, T6G 2H1 (Danny Shpeley)

**UMHU** Systematic Entomology, Graduate School of Agriculture, Hokkaido University, Sapporo, 060-8589 Japan (Masashi Ohara)

All specimens have been databased and incorporated into the University of Alberta, EH Strickland Virtual Entomology Museum Database. This includes NCHU reference numbers, full locality data, dates of collection, collectors, and codens. It can be accessed at http://www.entomology.ualberta.ca.

Standard methods were used for mounting, dissecting, preparing genitalia, and other technical methods (Ball and Hilchie 1983, Frania and Ball 2007). Genitalia and other small structures were preserved in glycerine and stored in microvials that were pinned beneath the specimen from which they had been removed.

Photographs of species habitus were taken using a Nikon D7100 fitted with an AF-S VR Micro-NIKKOR 105mm f/2.8G IF-ED lens and mounted on a copy stand. Photographs of genitalia were taken with a Nikon D7100 mounted on an Olympus SZX16 trinocular stereoscopic microscope and layered together using Zerene Stacker (Zerene Systems LLC, Richland, WA). Line drawings of the female genital tracts and other external characters were prepared by taking photographs with a Nikon D7100 and then importing them into Adobe Illustrator 11.0 (Adobe Systems, Inc., Mountainview, CA). Plates were also prepared using Adobe Illustrator 11.0. All photographs taken in the field were taken by Dash Hwang.

Geographic range maps were prepared using a modified map from Ginkgo Maps (http://www.ginkgomaps.com); projection used is NAD Lambert Conformal Conic, 1983.

**Figure 1.**
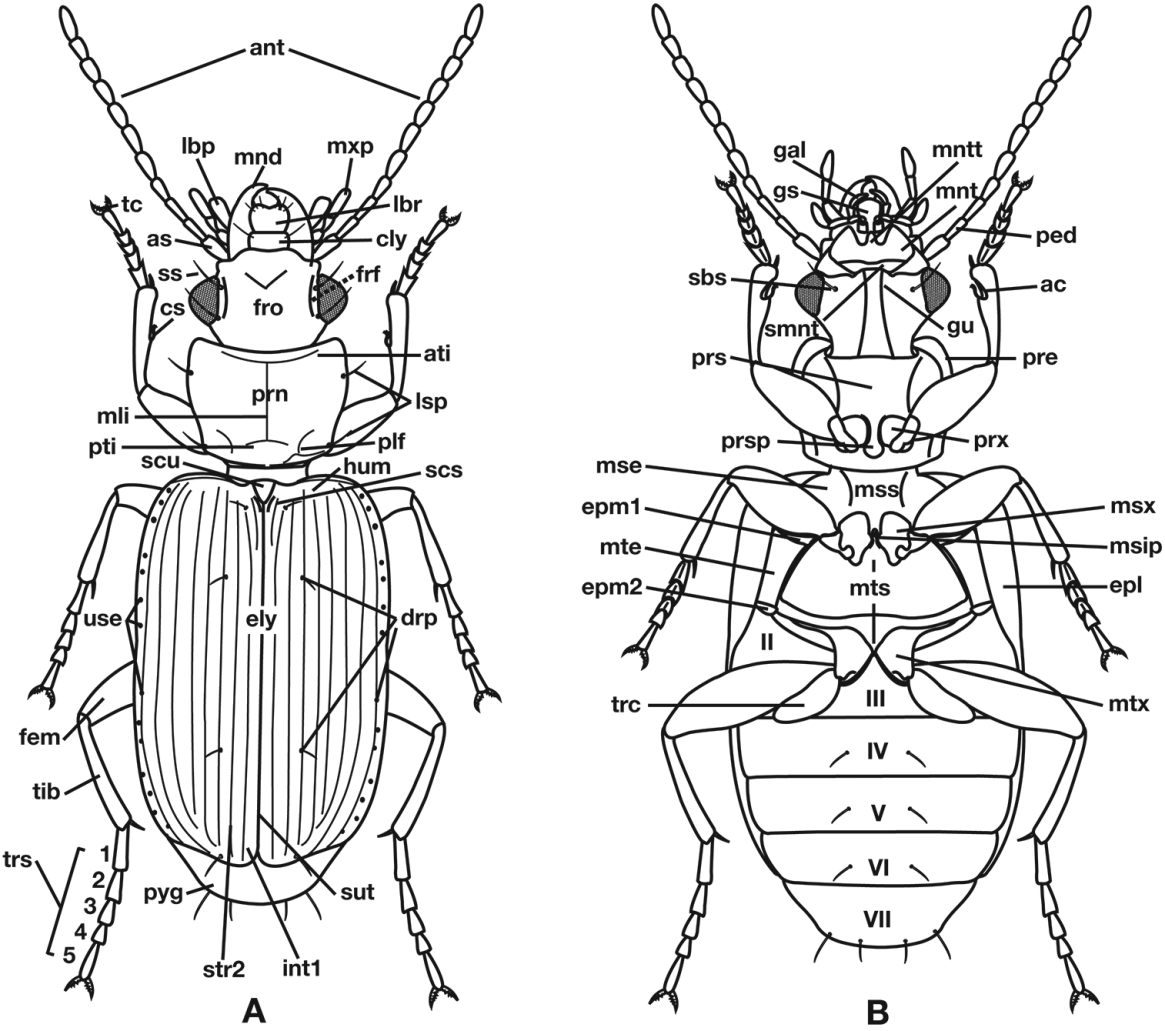
Structure of a generalized lebiine ground beetle (Carabidae) **A** dorsal view **B** ventral view. Adapted from Hunting, 2013.

Measurements were made at 25× with a Wild M5 stereoscopic microscope fitted with an ocular micrometer. Various measurements are expressed in the text by these abbreviations previously used by Ball and Shpeley (2005) and Hunting (2013). Terms used for structural characters follow Shpeley and Ball (1983), and Hunting (2008, 2013) and other authors (see also Figure 1 and Legend for Figure 1). For some characters of the genital tract of both males and females, no nomenclature has been developed, so in these instances informal descriptive words or phrases are used.

**HL** Length of head, measured on left side, from base of left mandible to posterior margin of compound eye,

**HW** Width of head, maximum transverse distance across head, including eyes,

**PL** Length of pronotum along midline,

**PWM** Maximum width of pronotum,

**ML** Metepisternum length,

**MW** Metepisternum width,

**EL** Length of elytra from basal ridge to apex,

**EW** Maximum width of elytra,

**OBL** Overall body length.

The shape of the head and pronotum is shown by the ratio of the width over length (HW/HL; PWM/PL, ML/MW), and elytral shape is indicated by the ratio of the length to the width (EL/EW).

### Classification of the supraspecific taxa of the subtribe Pericalina, tribe Lebiini, of Taiwan

Family Carabidae

Subfamily Lebiinae

Tribe Lebiini (*s. str*)

Subtribe Pericalina

Genus *Amphimenes* Bates

*A.asahinai* Nakane

*A.absensacidus* sp. n.

*A.beichatiensis* sp. n.

*A.carinacaulis* sp. n.

Genus *Bellavalentis* gen. n.

*B.kuzugamii* (Shibata)

Genus *Brachichila* Chaudoir

*B.hypocrita* Chaudoir

Genus *Catascopus* Kirby

Subgenus Catascopus (s. str.)

*C.asaharti* sp. n.

*C.ignicinctus* Bates

*C.sauteri* Dupuis

*C.viridiorchis* sp. n.

Subgenus Catascopoides Habu

*C.horni* Jedlička

Genus *Coptodera* Dejean

Subgenus Coptoderina Jeannel

*C.chaudoiri* Andrewes

*C.eluta* Andrewes

*C.japonica* Bates

*C.maculata* (Dupuis)

*C.marginata* (Dupuis)

*C.occulta* sp. n.

*C.proksi* Jedlička

*C.taiwana* (Nakane)

Genus *Dolichoctis* Schmidt-Goebel

Subgenus Dolichoctis (s. str.)

*D.badiadorsis* sp. n.

*D.dilatata* sp. n.

*D.rotundata* (Schmidt-Goebel)

*D.taiwanensis* Baehr

Genus *Formosiella* Jedlička

*F.brunnea* Jedlička

*F.flavomaculata* (Shibata)

Genus *Holcoderus* Chaudoir

*H.formosanus* Jedlička

Genus *Horniulus* Jedlička

*H.andrewesi* Jedlička

Genus *Lioptera* Chaudoir

*L.erotyloides* Bates

Genus *Miscelus* Klug

*M.javanus* Klug

Genus *Mochtherus* Schmidt-Goebel

*M.luctuosus* Putzeys

*M.obscurabasis* sp. n.

*M.tetraspilotus* (MacLeay)

Genus *Pericalus* MacLeay

Subgenus Pericalus (s. str.)

*P.formosanus* Dupuis

Genus *Serrimargo* Chaudoir

*S.schenklingi* (Dupuis)

**Legend for Figure 1**

**ac** antenna cleaner

**ant** antenna

**as** antennal scape (antennomere 1)

**ati** anterior transverse impression of pronotum

**cly** clypeus

**cs** clip setae of antenna cleaner

**drp** discal punctures of elytra

**ely** elytra

**epl** epipleuron of elytron

**epm1** epimeron of mesosternum

**epm2** epimeron of metasternum

**fem** femur

**frf** frontal furrow

**fro** frons

**gal** galea

**gs** glossal sclerite

**gu** gula

**hum** humerus

**lbp** labial palpus

**lbr** labrum

**lsp** lateral setae of pronotum

**mli** median longitudinal impression

**mnd** mandible

**mnt** mentum

**mntt** mental tooth

**mse** mesepisternum

**msip** mesosternal intercoxal process

**mss** mesosternum

**msx** mesocoxa

**mte** met-episternum

**mts** meta-sternum

**mtx** hindcoxa

**mxp** maxillary palpus

**ped** pedicel (antennomere 2)

**plf** posteriolateral fovea of pronotum

**pre** proepisternum

**prn** pronotum

**prs** prosternum

**prsp** prosternal intercoxal process

**prx** forecoxa

**pti** posterior transverse impression

**pyg** pygidium (= tergum VII)

**sbs** suborbital setae

**scs** scutellar stria

**scu** scutellum

**smnt** submentum

**ss** supraorbital setae

**sut** suture of elytra

**tc** tarsal claw

**tib** tibia

**trc** trochanter

**trs** tarsus (labeled 1–5)

**use** umbilical setae of elytra

**int1** elytral interval 1

**str2** elytral stria 2

**II–VII** pregenital sterna

To indicate range of body size of each species, the overall body length (OBL) was measured from the apex of the extended mandibles, to the apex of the elytra of both the largest and smallest individual of the species (Frania and Ball 2007).

Size of male genitalia was determined by drawing a straight line between the apical area and the basal lobe of the phallus. Size of female genitalia was determined by drawing a straight line across the outside margin of widest portion of left lateral tergite to outside margin of widest portion of right lateral tergite.

To reduce repetition, character states of lower ranking taxa recorded in the descriptions of higher-ranking taxa are not repeated in the descriptions of the included lower ranking taxa. As such, a complete species description will require reading both the recognition of the genus as well as the species description.

For type material, information from each label is reproduced using ordinary type. Information on each label is contained in quotation marks, with a semicolon marking the end of each label. Information on color of paper (other than white), printing (other than black), form of paper (other than rectangular), and coden for the collection in which material is housed, is contained in square brackets.

## Taxonomy

### Order Coleoptera Linnaeus, 1758

#### Family Carabidae Latreille, 1802

##### Subfamily Lebiinae Bonelli, 1810

###### Subtribe Pericalina

**Classification.** For a detailed account see Ball (1975) and Shpeley and Ball (2000).

####### Key to the Taiwanese genera and subgenera of the subtribe *Pericalina* Hope

**Table:** 

1	Tarsal claws smooth	**2**
–	Tarsal claws denticulate, three or more denticles per claw	**7**
2	Dorsal surface of head with two pairs of supraorbital setae between eyes	**3**
–	Dorsal surface of head with single pair of supraorbital setae between eyes	***Miscelus* Klug**
3	Pronotum with one to two pairs of latero-marginal fixed setae	**4**
–	Pronotum with three pairs of latero-marginal fixed setae	***Horniulus* Jedlička**
4	Pronotum with two pairs of latero-marginal fixed setae, elytral surface shiny or metallic, not distinctively granulate	**5**
–	Pronotum with one pair of baso-lateral fixed setae, elytral surface distinctively granulate	***Serrimargo* Chaudoir**
5	Elytral surface concolorous or nearly so, with metallic sheen	**6**
–	Elytral surface black with eight yellowish to testaceous maculae, shiny but not metallic	***Pericalus* MacLeay**
6	Mandibles elongate and almost straight, distinctively asymmetric, left mandible with obtuse tooth on inner margin, before apex, black	**subgenus Catascopoides Habu**
–	Mandibles curved, not distinctively asymmetric, color various but not black	**subgenus Catascopus s. str. Kirby**
7	Mentum with tooth (Fig. 8A)	**8**
–	Mentum without tooth (Fig. 8B)	**11**
8	Pronotum with margin somewhat explanate to widely explanate, dorsal color various but not metallic	**9**
–	Pronotum with margin very narrow, finely and evenly raised along lateral margins, dorsal color metallic blue to metallic green	***Holcoderus* Chaudoir**
9	Dorsum of head and pronotum with scattered setigerous punctures, setae easily visible in lateral view at 50× magnification	**10**
–	Dorsum of head and pronotum glabrous	***Amphimenes* Bates**
10	Elytra with three fixed setae in interval 3, apical article of labial and maxillary palpi distinctly acuminate (Fig. 50B), sternum VII of male distinctly bilobed	***Formosiella* Jedlička**
–	Elytra with two fixed setae in interval 2, apical article of labial and maxillary palpi subtruncate (i.e., Fig. 50A) (typical of most pericalines), sternum VII of males not distinctly bilobed	***Mochtherus* Schmidt-Goebel**
11	Size moderate to small, overall body length less than 11 mm, elytral striae moderately to deeply impressed	**12**
–	Size large, overall body length more than 11 mm, elytral striae very shallowly impressed	***Lioptera* Chaudoir**
12	Medium size, overall body length more than 6 mm, with elytral maculae	**13**
–	Small size, overall body length less than 6 mm, with or without elytral maculae	**14**
13	Elytron with two or more fixed discal setae, pronotum with margins widely explanate and broadly rounded latero-apically	**subgenus Coptoderina Jeannel**
–	Elytron with a single discal seta near apex of stria 2, pronotum only slightly explanate latero-apically	***Brachichila* Chaudoir**
14	Body form convex, elytral margins rounded, overall body length less than 4 mm	***Bellavalentis* gen. n.**
–	Body form moderately flattened, eltyral margins only slightly rounded medially, overall body length more than 4 mm	***Dolichoctis* Schmidt-Goebel**

######## Genus Amphimenes

Taxon classificationAnimaliaColeopteraCarabidae

Bates

Amphimenes Bates, 1873: 322; Csiki 1932: 1353; Jedlička 1963: 366; Habu 1964: 472; 1967: 113; 1982: 90; Nakane 1957: 43; Lorenz 2005: 456; Fedorenko 2010: 17; 2014: 305.
Pseudosinurus Kirschenhofer, 1999: 74.

######### Type species.

*Amphimenespiceolus* Bates, 1873 (monobasic).

######### Type locality.

Nagasaki, Japan

######### Recognition of Taiwanese species of *Amphimenes*

*Color.* Various.

*Pilosity*. Dorsum of head and pronotum glabrous.

*Fixed setae.* Two pairs of supraorbital setae; clypeus with two lateral setae; labrum with six setae along apical margin; one pair of suborbital setae; pronotum with two setae along each margin; elytra with one seta in basal third of stria 3, two setae in apical third of stria 2; 16–17 lateral (umbilical) setae in interval 9; two setae on each of abdominal sterna III to VI, two setae along apical margin of sternum VII in males, four setae along apical margin of sternum VII in females.

*Luster*. Head capsule and pronotum dull.

*Head*. Mentum with single broad tooth; labium and palpi typical for genus *Amphimenes*.

*Pronotum*. Anterior and posterior transverse impressions shallow; median longitudinal impression shallow; apical margin curved forming two latero-apical lobes.

*Elytra*. Striae moderately impressed; elytral apices truncate.

*Legs*. Tarsal claws pectinate, three to five denticles per claw. Males with adhesive vestiture ventrally, two rows of squamo-setae on tarsomeres 1–3 of fore-leg.

*Male.* Ostium left pleuropic. Phallus cylindrical.

*Female genitalia*. Gonocoxite 2 (gc2) long and narrow; two lateral ensiform (les) setae and one dorsal ensiform seta (des) present. Sensory furrow, furrow pegs and associated nematiform setae not observed.

######### Taxonomic notes.

Jedlička (1963) recorded the type species, *A.piceolus* Bates from both Taiwan and Fukien, China. The type of *A.piceolus* from BMNH, London was examined as well as the material from both Taiwan and Fukien, China that Jedlička was referring to in his work at NMPC, Prague. After dissection and comparison of these specimens the example that Jedlička had from Taiwan was not *A.piceolus*, but rather *A.carinacaulis* sp. n. The specimen from Fukien, China, was also not *A.piceolus*, but something different from all other species examined from Asia and likely an undescribed species. After also examining the types of *A.asahinai* Nakane, *A.ryukuensis* Habu and undetermined material from several collections, it appears that *A.piceolus* is likely restricted to Japan only. One other specimen examined from Okinawa, Japan, on loan from NCHU, also appears to be an undescribed species.

######## Key to the Taiwanese species of the genus *Amphimenes* Bates

**Table:** 

1	Fully winged, metepisternum long, 1.50× as long as wide or longer, elytra with basolateral angles of humeri almost parallel	**2**
–	Wingless, metepisternum short, less than 1.40× as long as wide; elytra with basolateral angles of humeri obliquely rounded	***A.asahinai* Nakane**
2	Elytra disc with cross striations of striae very distinctive to somewhat fine; always present, pronotum with margins not widely explanate	**3**
–	Elytra disc without cross striations on striae, pronotum with margins widely and relatively explanate	***A.absensacidus* sp. n.**
3	Elytral with suture clearly brunneous to rufo-brunneous in apical half, cross striations fine, punctures not easily evident	***A.carinacaulis* sp. n.**
–	Elytral with suture black in apical half, cross striations deep, randomly scattered punctures +/- irregularly shaped in basal half of disc	***A.beichatiensis* sp. n.**

######### Amphimenes asahinai

Taxon classificationAnimaliaColeopteraCarabidae

Nakane

**Figure 2.**
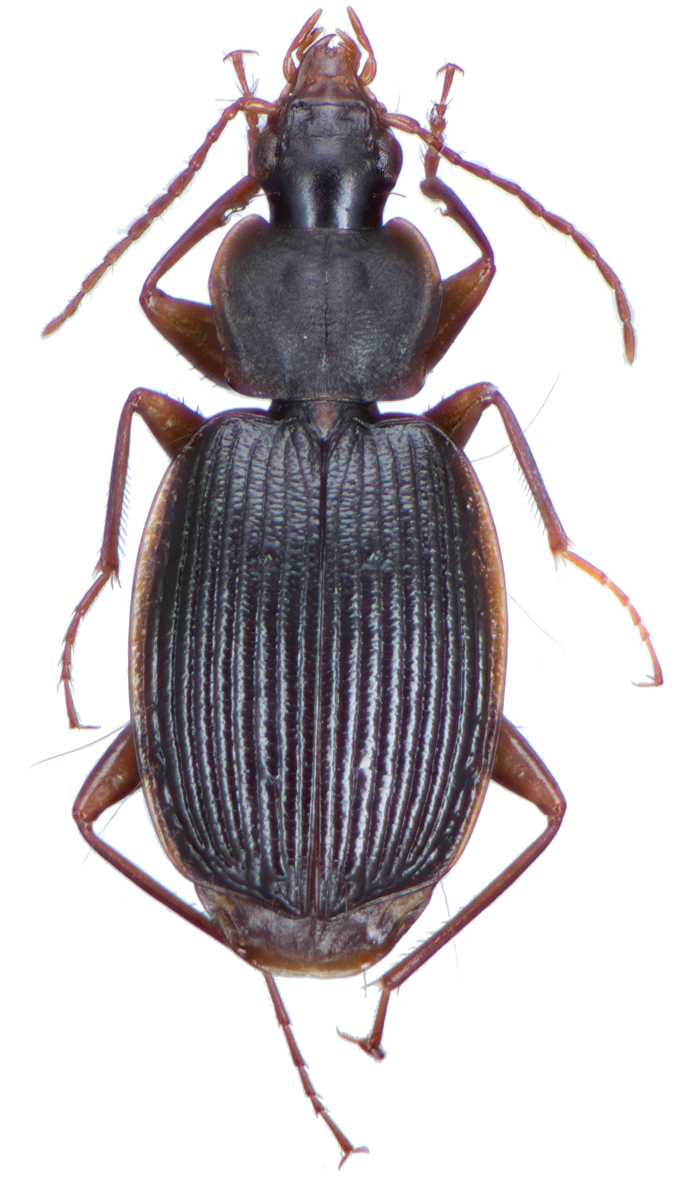
Dorsal habitus and color pattern of *Amphimenesasahinai* Nakane. (OBL 6.20 mm).

**Figure 3.**
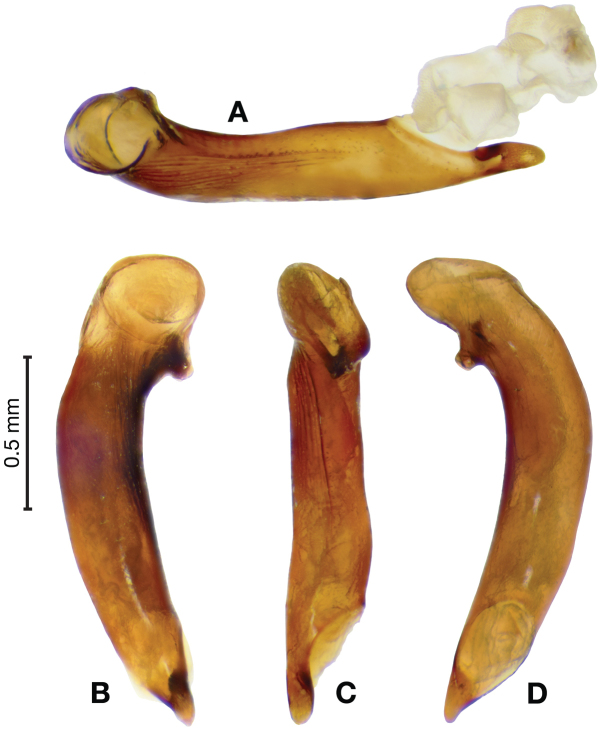
Digital images of male genitalia of *Amphimenesasahinai* Nakane. **A** ventral aspect, endophallus everted **B** right lateral aspect **C** ventral aspect **D** left lateral aspect.

Figs 2 , 3A–D , 11A , 12

Amphimenes asahinai Nakane, 1957: 237 fig. 2 ; Habu 1964: 472 – 474, 477, 478; Fedorenko 2010: 14–50; Lorenz 2005: 456.

########## Types and other material examined.

**Holotype** (male) labeled:”HOLOTYPE” [rectangular, red]; “Jujiro/Near Mt. Ari,/Formosa./VI-8 1938/Coll. Yoshio Yano”; Yoshio Yano;s/Collection/No. 7543”; ”No. 2555/Yoshio Yano/Collection”; ”Y. Yano’s/Collection/Type No. 443”Nakane coll./Sehu Japan/1999”; “0000000552/Sys. Ent/Hokkaido Univ./Japan [SEHU]”; “NCHU#/101135”. 455 specimens, 236 males and 219 females. For further details see EH Strickland Virtual Entomology Museum Database.

########## Type locality.

Taiwan. The type is a specimen collected by Yoshio Yano in 1938. The label data indicates that it is from “Jujiro, near Mt. Ari”. Jujiro is now referred to as Shihtzulu and Mt. Ari is Alishan, Chiayi County.

########## Diagnosis.

Specimens of this species are distinguished from other Taiwanese *Amphimenes* by being brachypterous, having a short metepisternum and more humeri with basal angles obliquely rounded.

########## Redescription.

OBL 5.3 – 7.6 mm. Length (n = 30 males, 30 females): head 0.50 – 0.72, pronotum 1.00 – 1.44, elytra 3.00 – 4.17, metepisternum 0.52 – 0.80 mm; width: head 0.96 – 1.32, pronotum 1.40 – 2.10, elytra 2.17 – 3.17, metepisternum 0.44 – 0.60 mm.

*Body proportions*. HW/HL 1.70 – 2.06; PWM/PL 1.33 – 1.50; EL/EW 1.21 – 1.38; ML/MW 1.18 – 1.42

*Color*. Fig. 2. Dorsum of head brunneous to brunneo-piceous; clypeus and mentum rufo-testaceous; pronotum brunneous with margins diffusely pale; proepipleuron rufo-brunneous; antennae rufo-brunneous to brunneous; palpi rufo-brunneous to brunneous; elytral disc brunneous, margins rufo-brunneous, translucent; elytral epipleura brunneous; thoracic sclerites brunneous to brunneo-piceous; abdominal sterna rufo-brunneous medially and brunneous at lateral margins; legs with trochanter and femora testaceous to rufo-testaceous, tibia rufo-testaceous to brunneous.

*Microsculpture*. Dorsum of head and pronotum with distinctive isodiametric mesh pattern easily visible at 50× magnification; elytra with shallow and markedly elongate, transverse sculpticells faintly visible throughout; ventral surface of head, prosternum, proepipleuron, mesepisternum and metepisternum with sculpticells forming a moderately deep, transverse mesh.

*Macrosculpture*. Elytra with distinct cross-striations along length of intervals, deeper and more distinctive towards base; striae faintly punctate along length.

*Pilosity*. Elytra with scattered micro-punctures; striae punctures each bearing a small seta not visible at 50× magnification.

*Luster*. Elytra moderately glossy to moderately dull; ventral thoracic sterna and abdominal sterna moderately dull.

*Head*. Labrum bilobed; eyes somewhat flattened in appearance, following contour of head.

*Pronotum*. Lateral margins sinuate toward base, distinctly angled from lateral setae towards base; posterio-lateral margins obtuse almost right-angled.

*Elytra*. Humeri narrowly rounded; striae moderately impressed; elytral disc with distinctive form at base, sloping laterally from base of stria 6 to lateral margin; lateral margin smooth, broadly rounded; elytral apices truncate.

*Hind wings*. Brachypterous, wings markedly reduced.

*Legs*. Meso-tibia with or without several shallow notches from mid-way to base along ventral surface.

*Abdominal sterna.* Abdominal sterna IV–VI smooth between fixed apical setae; abdominal sternum VII not bilobed.

*Male genitalia*. Fig. 3A–D. Length 1.36–1.64 mm. Phallus with several carinae from base to mid-phallus, decreasing in length from center to right of center when viewed ventrally; endophallus short, distinctive form, small internal endophallic sclerite near apex.

*Female genitalia*. Fig. 11A. Width 0.84–0.92mm. Two spermathecae; spermatheca 1 (sp1) and 2 (sp2) with ducts ribbed and lumpy in appearance. One spermathecal accessory gland (sg); spermathecal gland duct (sgd) attachment site on spermatheca 1 duct, well beyond attachment site of spermatheca 2.

########## Habitat, habits, and seasonal occurrence.

The known elevational range of *A.asahinai* is from 500 to 2290 meters. Only five specimens of the 455 collected were from below 1000 meters and most were collected at over 1800 meters. Adults of this species are found in mixed primary and secondary forest of montane areas. Adults are crepuscular or nocturnal with most activity observed on live tree trunks at night. Specimens have been collected all year round but are most commonly collected from March to October. Methods of collecting include u.v. light, m.v. light, sweep netting, sugar baits painted on tree trunks, hand collecting, and insecticidal fogging at night. Confirmed tree species that *A.asahinai* has been collected from includes: *Pinusmorrisonicola* Hayata, *Neolitseavariabilima* (Hayata), *Schimasuperba* Gard. and *Castanopsiseyrei* (Champ ex. Benth).

########## Geographical distribution.

*Amphimenesasahinai* is known only from Taiwan. See Figure 12.

######### Amphimenes absensacidus sp. n.

Taxon classificationAnimaliaColeopteraCarabidae

http://zoobank.org/01478AFD-2061-453B-95EF-D82FF1FC73F1

**Figure 4.**
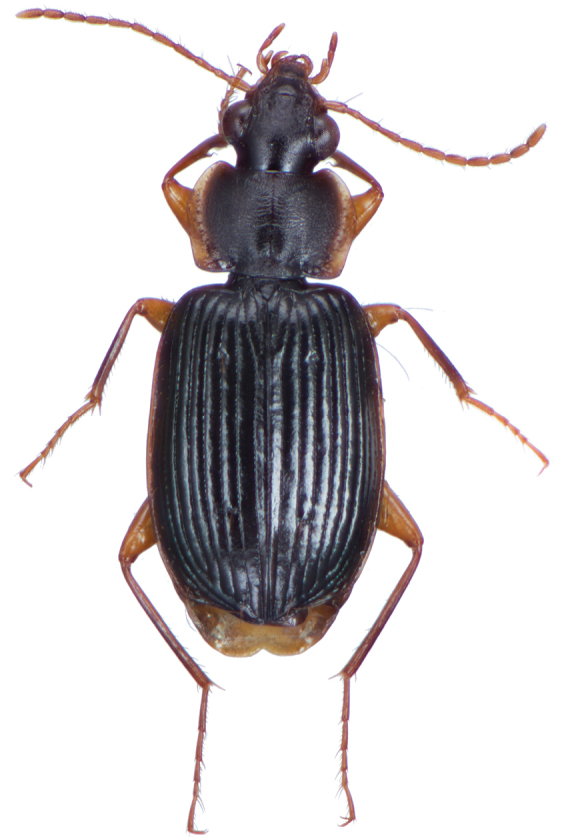
Dorsal habitus and color pattern of *Amphimenesabsensacidus* sp. n.. (OBL 6.40 mm).

**Figure 5.**
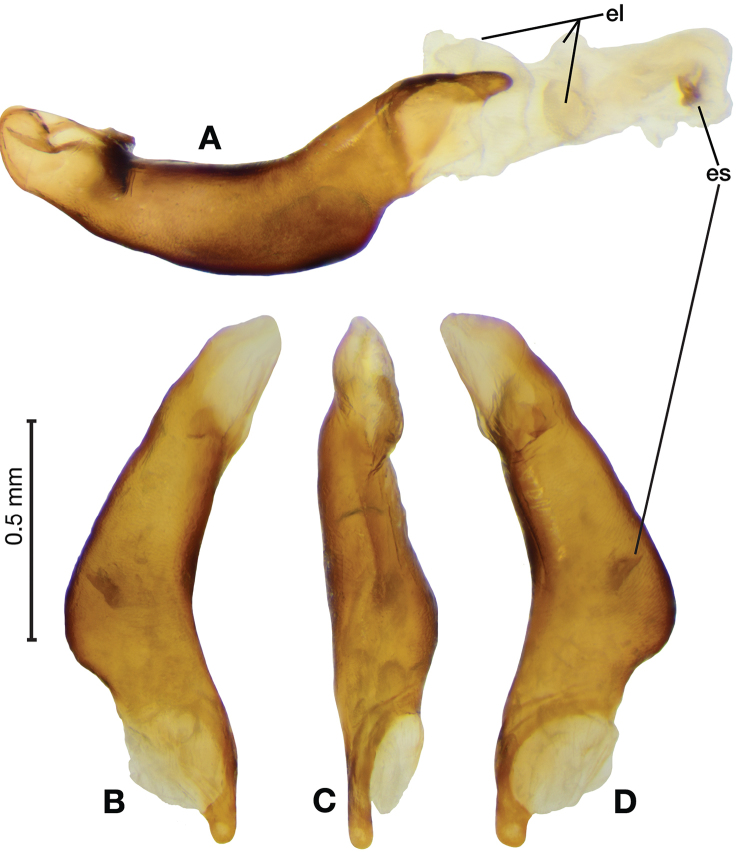
Digital images of male genitalia of *Amphimenesabsensacidus* sp. n.. **A** right lateral aspect, endophallus everted **B** right lateral aspect **C** ventral aspect **D** left lateral aspect. Legend: **el** endophallic lobes; **es** endophallic sclerite.

Figs 4 , 5A–D , 11B , 12

########## Specific epithet.

From Latin, *absens* and *acidus*, in reference to the lack of cross striations on the intervals of the elytral disc, typical in many species of this genus and all other species known from Taiwan.

########## Types and other material examined.

**Holotype** (male) labeled “Holotype” [circular, ringed with red]; “TAIWAN: “TAIWAN: Nantou Co./Huisun Forest Station /Hotel, May 26, 2012/24.0932N, 121.0310E”; “hand coll., veg. nr./Frog Rock trail, night,/~740m, Acc. Ti-170a/Coll. W. M. Hunting”; “NCHU#/100728”. 28 **paratypes**, 13 males and 15 females. For further details see EH Strickland Virtual Entomology Museum Database.

########## Type locality.

Taiwan. Huisun Forest Station, Nantou county.

########## Diagnosis.

Specimens of this species are distinguished from other Taiwanese *Amphimenes* by lacking cross striations on the elytral disc.

########## Description.

OBL 5.8 – 6.8 mm. Length (n = ten males, ten females): head 0.58 – 0.70, pronotum 1.08 – 1.24, elytra 3.16 – 3.80, metepisternum 0.72 – 0.84 mm; width: head 1.12 – 1.36, pronotum 1.64 – 2.04, elytra 2.17 – 2.75, metepisternum 0.44 – 0.50 mm.

*Body proportions*. HW/HL 1.87 – 2.14; PWM/PL 1.52 – 1.67; EL/EW 1.31 – 1.47; ML/MW 1.50 - 1.82

*Color*. Fig. 4. Dorsum of head piceous; clypeus piceous; labrum piceous with brunneo-piceous margins; pronotum piceous with margins markedly pale, rufo-testaceous; proepipleuron rufo-testaceous to darker; antennae rufo-brunneous to brunneous; palpi rufo-brunneous to brunneous; elytral disc piceous, margins rufo-brunneous, translucent; elytral epipleura piceous to brunneo-piceous; thoracic sclerites rufo-brunneous to piceous; abdominal sterna rufo-brunneous medially and brunneo-piceous to piceous at lateral margins; legs with trochanter and femora testaceous to rufo-testaceous, tibia rufo-testaceous to brunneo-piceous.

*Microsculpture*. Dorsum of head and pronotum with distinctive isodiametric mesh pattern easily visible at 50× magnification; elytra with transverse sculpticells visible throughout, moderately elongate; ventral surface of head, prosternum, proepipleuron, mesepisternum and metepisternum with sculpticells forming a moderately distinctive transverse mesh.

*Macrosculpture*. Pronotum with scattered punctures in lateral margins; elytral striae evenly punctate along length.

*Pilosity*. Elytra with scattered micro-punctures, each bearing a small seta; not visible at 50× magnification.

*Luster*. Elytra moderately glossy to moderately dull; ventral thoracic sterna and abdominal sterna moderately glossy.

*Head*. Labrum more or less quadrate, few with slight indentation along apical margin but not bilobed; eyes distinctly convex, distinctly wider across than width of neck.

*Pronotum*. Lateral margins distinctly explanate, broadly rounded; posterio-lateral margins rounded; posterio-lateral fovea relatively deep; basal lobe present.

*Elytra*. Humeri broadly rounded; striae moderately impressed; lateral margins nearly parallel; elytral apices truncate.

*Hind wings*. Macropterous.

*Legs*. Meso-tibia with or without several shallow notches from mid-way to base along ventral surface.

*Abdominal sterna.* Abdominal sterna IV–VI with two to four irregular punctures between fixed apical setae; abdominal sternum VII deeply bilobed.

*Male genitalia*. Fig. 5A–D. Length 1.28 – 1.44 mm. Phallus with one carina from base to mid-phallus, centered when viewed ventrally. Left side of shaft distinctively enlarged medially; endophallus with distinctive form, relatively large internal endophallic sclerite (es) near apex and several endophallic lobes (el).

*Female genitalia*. Fig. 11B. Width 0.84 – 0.92mm. Two spermathecae, spermatheca 1 (sp1) and 2 (sp2) with ducts relatively smooth and even throughout length; One spermathecal accessory gland (sg); spermathecal gland duct (sgd) attachment site at base of spermatheca 1 duct, basal to attachment site of spermatheca 2 and associated diverticulum (div); one diverticulum, elongate.

########## Habitat, habits, and seasonal occurrence.

The known elevational range of *A.absensacidus* sp. n. is from 700 to 1850 meters. Adults of this species are found in mixed primary and secondary forest of montane areas. Adults are crepuscular or nocturnal with most activity observed on live tree trunks at night. Specimens have been collected from May to August. Methods of collecting include u.v. light and hand collecting at night.

########## Geographical distribution.

*Amphimenesabsensacidus* is known only from Taiwan. See Figure 12.

######### Amphimenes beichatiensis sp. n.

Taxon classificationAnimaliaColeopteraCarabidae

http://zoobank.org/9296F080-66B8-42A0-B244-A59A5B9EACE1

**Figure 6.**
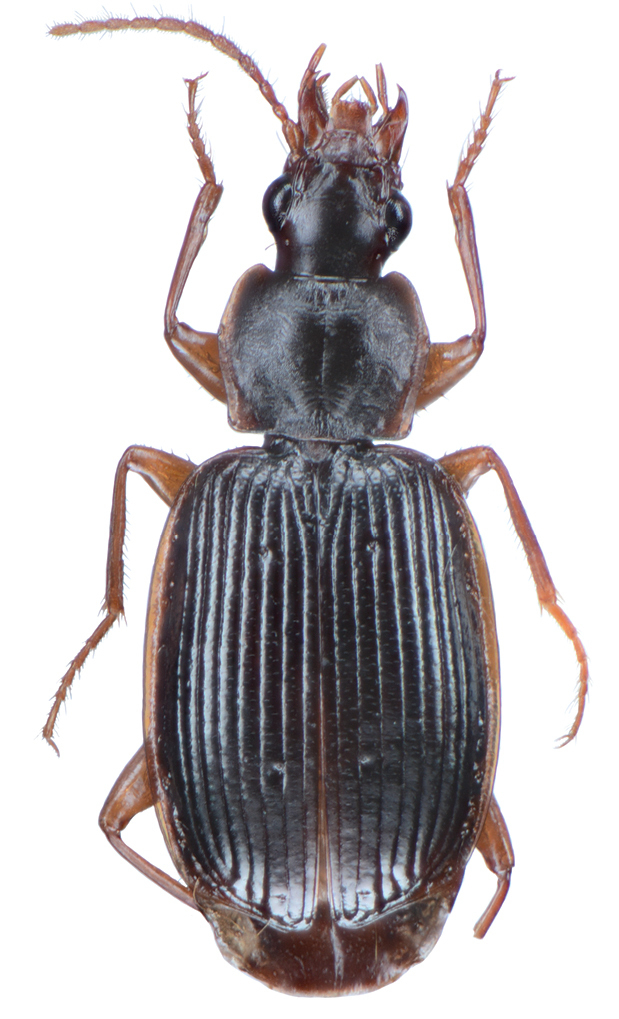
Dorsal habitus and color pattern of *Amphimenesbeichatiensis* sp. n.. (OBL 6.30 mm).

**Figure 7.**
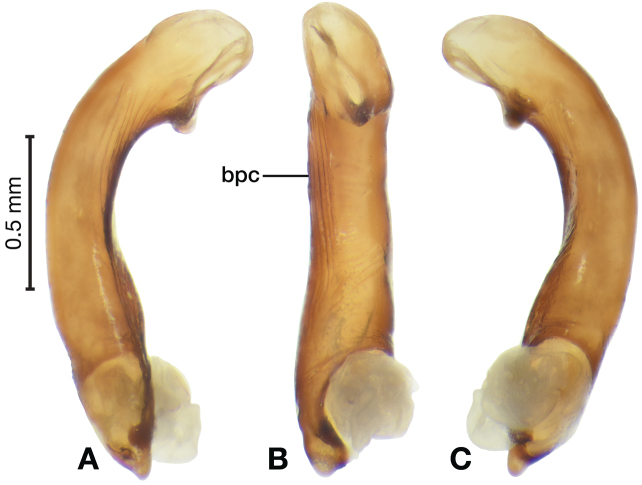
Digital images of male genitalia of *Amphimenesbeichatiensis* sp. n.. **A** right lateral aspect **B** ventral aspect **C** left lateral aspect. Legend: **bpc** basal phallic carina.

**Figure 8.**
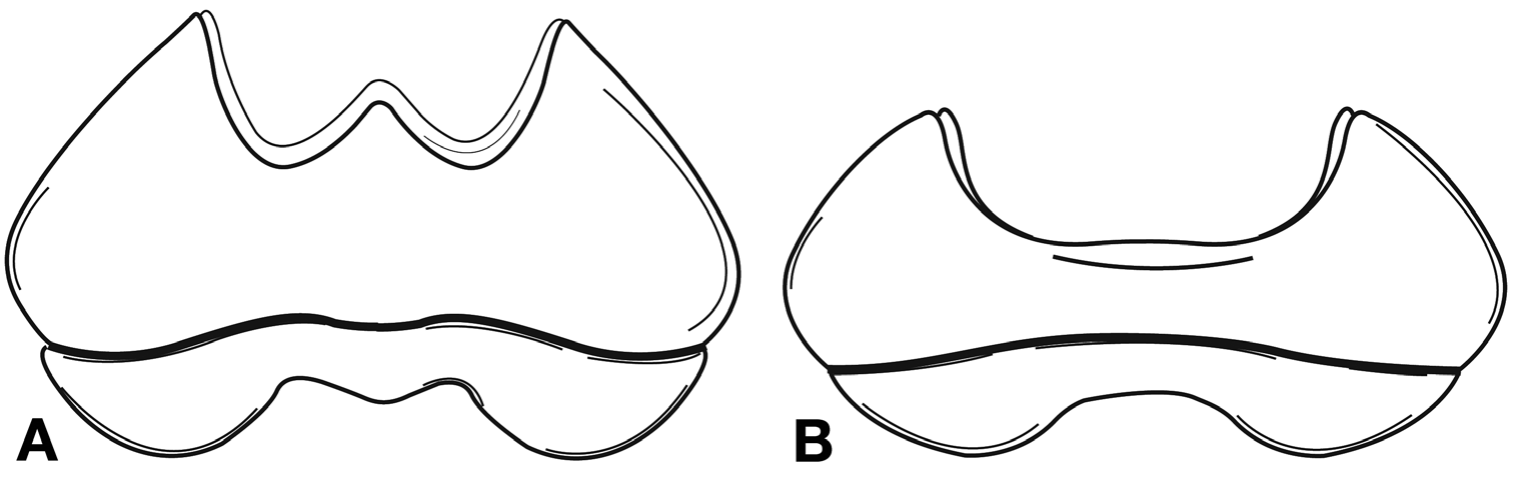
Line drawing and comparison of menta of **A***Amphimenesbeichatiensis* sp. n. (with tooth) **B***Brachichilahypocrita* Chaudoir (without tooth).

Figs 6 , 7A–C , 8A , 12

########## Specific epithet.

The name of this species refers to the locality, Beichatien Mountain (北插天山), where the single specimen was collected.

########## Types and other material examined.

**Holotype** (male) labeled “Holotype” [circular, ringed with red]; “TAIWAN: Taoyuan Co./Fuxing township,/Beichatien Mountain/24.7906N, 121.4410E”; “hand collecting, night/~1320m,/Acc. Ti-96b /June 17, 2011/Coll. W. M. Hunting”; “NCHU/100698”.

########## Type locality.

Beichatien Mountain (北插天山).

########## Diagnosis.

This species is distinguished from other Taiwanese *Amphimenes* by a combination of elytra with a black suture in apical half and a disc with randomly scattered punctures on entire dorsal surface of, +/- irregularly shaped in basal half.

########## Description.

OBL 6.3 mm. Length: head 0.64, pronotum 1.20, elytra 3.50, metepisternum 0.84 mm; width: head 1.12, pronotum 1.56, elytra 2.5, metepisternum 0.52 mm.

*Body proportions*. HW/HL 1.75; PWM/PL 1.30; EL/EW 1.40; ML/MW 1.62.

*Color*. Fig. 6. Dorsum of head brunneo-piceous; clypeus and labrum rufo-brunneous; pronotum nearly piceous with margins rufo-brunneous; proepipleuron rufo-brunneous; antennae rufo-brunneous; palpi rufo-brunneous; elytral disc nearly piceous, lateral margins and sutural margin rufo-brunneous, translucent; elytral epipleura rufo-brunneous; thoracic sclerites brunneo-piceous; abdominal sterna brunneo-piceous medially and nearly piceous at lateral margins; legs with trochanter and femora rufo-testaceous, tibia rufo-brunneous to brunneo-piceous.

*Microsculpture*. Dorsum of head and pronotum with isodiametric mesh pattern easily visible at 50× magnification; elytra with transverse sculpticells forming elongate mesh, faintly visible at 50× magnification.

*Macrosculpture and pilosity*. Elytra randomly scattered punctures on intervals, more or less evenly spaced throughout length, -/+ irregularly shaped in basal half; striae faintly punctate along length.

*Pilosity*. Elytra with scattered punctures visible, each bearing a small seta not visible at 50× magnification.

*Luster*. Elytra moderately glossy; ventral thoracic sterna and abdominal sterna moderately glossy.

*Head*. Fig. 8A. Labrum with indentation along apical margin, somewhat bilobed; eyes moderately convex.

*Pronotum*. Lateral margins narrow; posterio-lateral margins obtuse, almost right-angled; basal lobe present.

*Elytra*. Humeri broadly rounded; striae moderately impressed; lateral margins somewhat rounded; elytral apices truncate.

*Hind wings*. Macropterous.

*Legs*. Meso-tibia with several shallow notches from mid-way to base along ventral surface.

*Male genitalia*. Fig. 7A–C. Length 1.60 mm. Phallus with several carina from base (bpc), longer to shorter from center to right of center when viewed ventrally; ventral carina from median of shaft almost to ostium margin; endophallus with small internal endophallic sclerite near apex.

*Female genitalia*. Female unknown.

########## Habitat, habits, and seasonal occurrence.

The single specimen was collected from a live tree trunk in a montane mixed secondary forest. It was hand collected one night in June, at an elevation of 1320 meters.

########## Geographical distribution.

Known only from type locality. See Figure 12. This locality was not sampled well or often so it is possible that many more specimens will be collected in the near future.

######### Amphimenes carinacaulis sp. n.

Taxon classificationAnimaliaColeopteraCarabidae

http://zoobank.org/43DE8B60-4ED1-4BDB-AF68-A1AEB78E20DE

**Figure 9.**
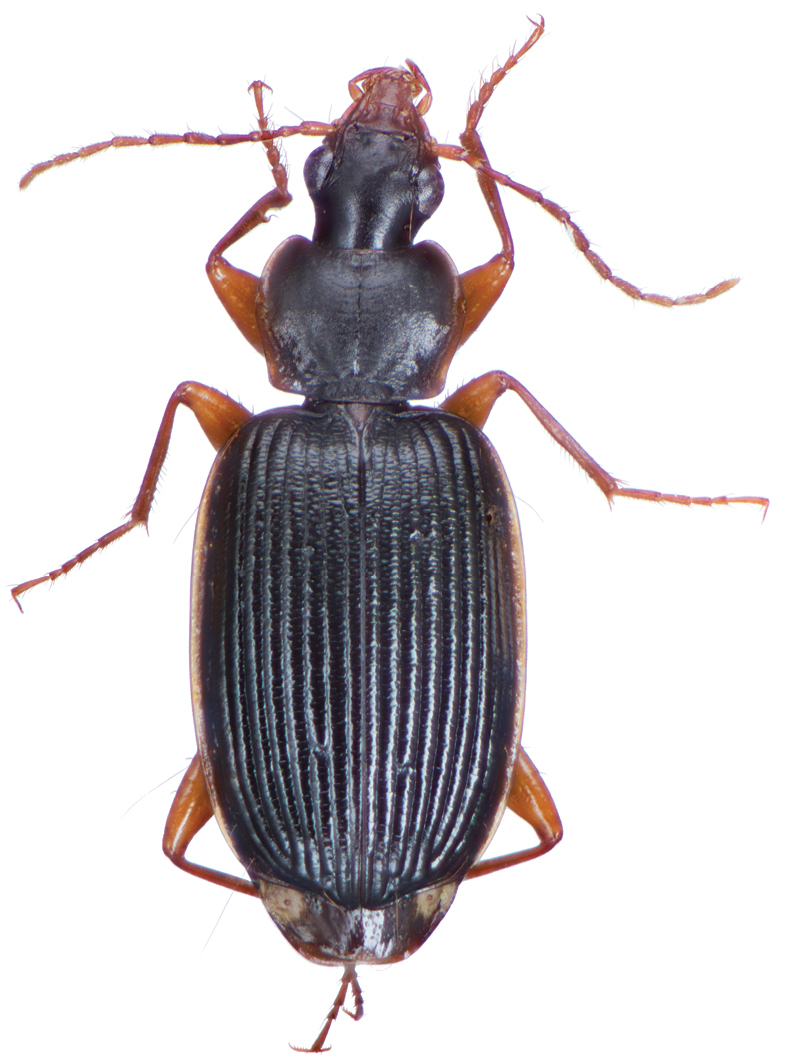
Dorsal habitus and color pattern of *Amphimenescarinacaulis* sp. n.. (OBL 7.35 mm).

**Figure 10.**
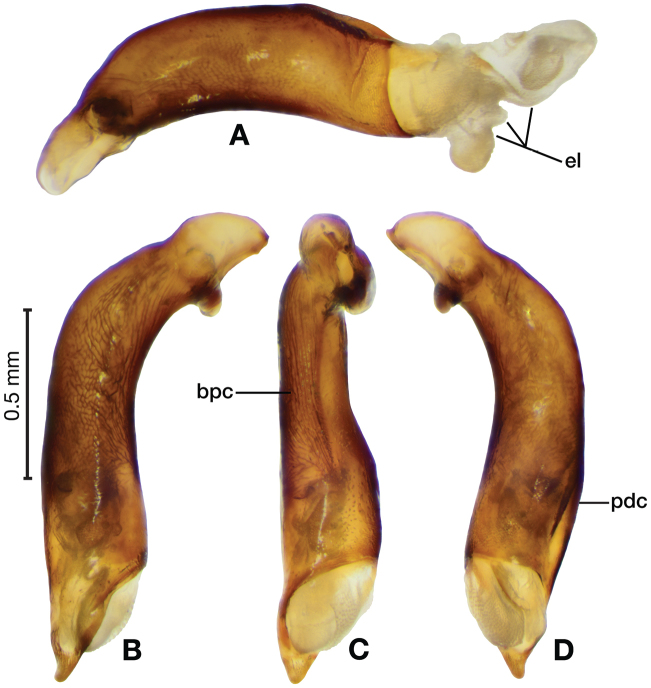
Digital images of male genitalia of *Amphimenescarinacaulis* sp. n.. **A** left lateral aspect, endophallus everted **B** right lateral aspect **C** ventral aspect **D** left lateral aspect. Legend: **bpc** basal phallic carina; **el** endophallic lobes; **pdc** preapical dorsolateral carina (apical endophallic sclerite not visible).

**Figure 11.**
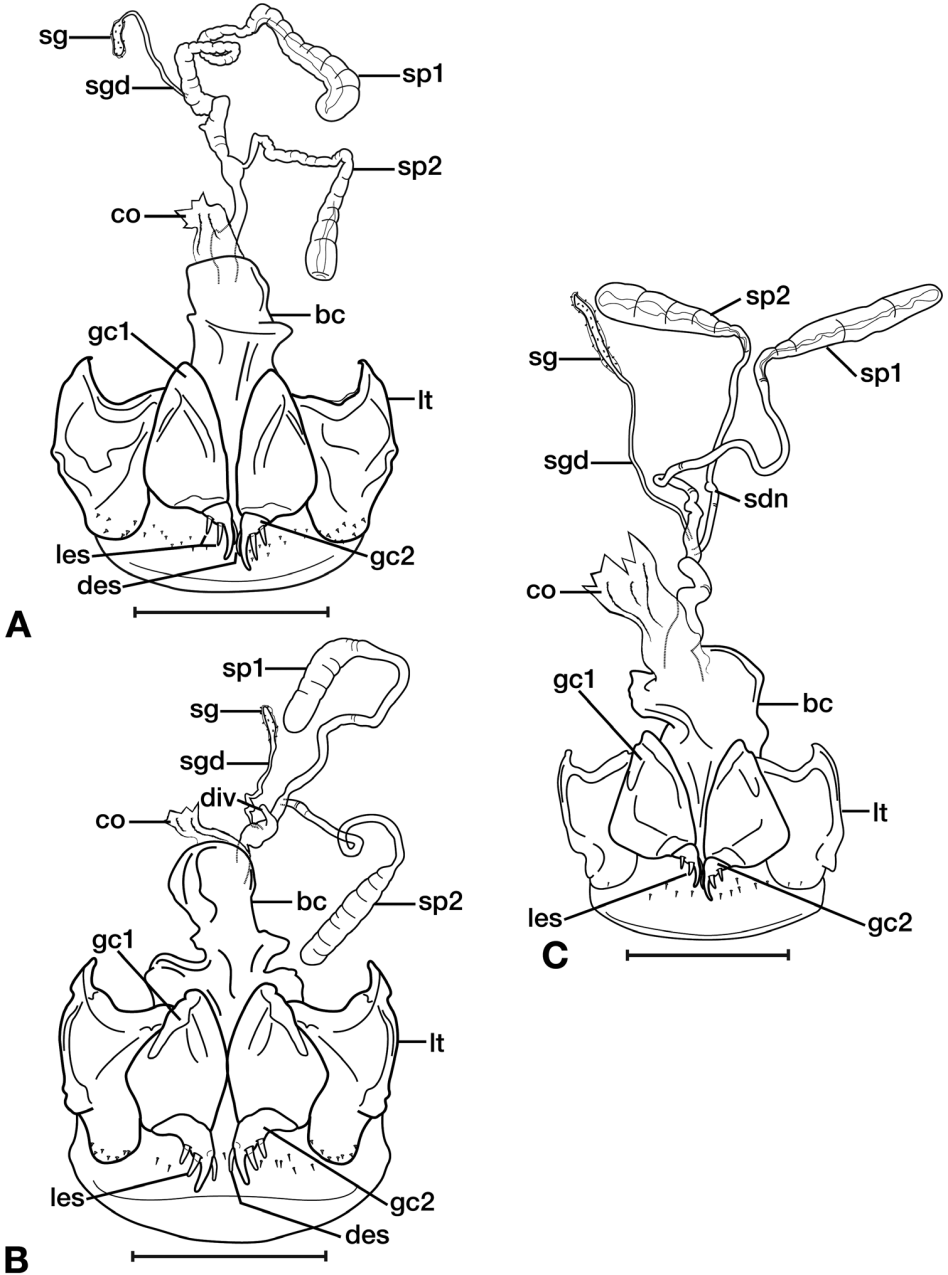
Line drawings of the female reproductive tract of species of the genus *Amphimenes* Bates, known from Taiwan, ventral aspect. **A***A.asahinai* Nakane **B***A.absensacidus* sp. n. **C***A.carinacaulis* sp. n.. Legend: **bc** bursa copulatrix; **co** common oviduct; **des** dorsal ensiform setae; **gc1** gonocoxite 1; **gc2** gonocoxite 2; **les** lateral ensiform setae; **lt** lateral tergite; **sg** spermathecal gland; **sgd** spermathecal gland duct; **sp1** spermatheca 1; **sp2** spermatheca 2. Scale bars 0.5 mm.

**Figure 12.**
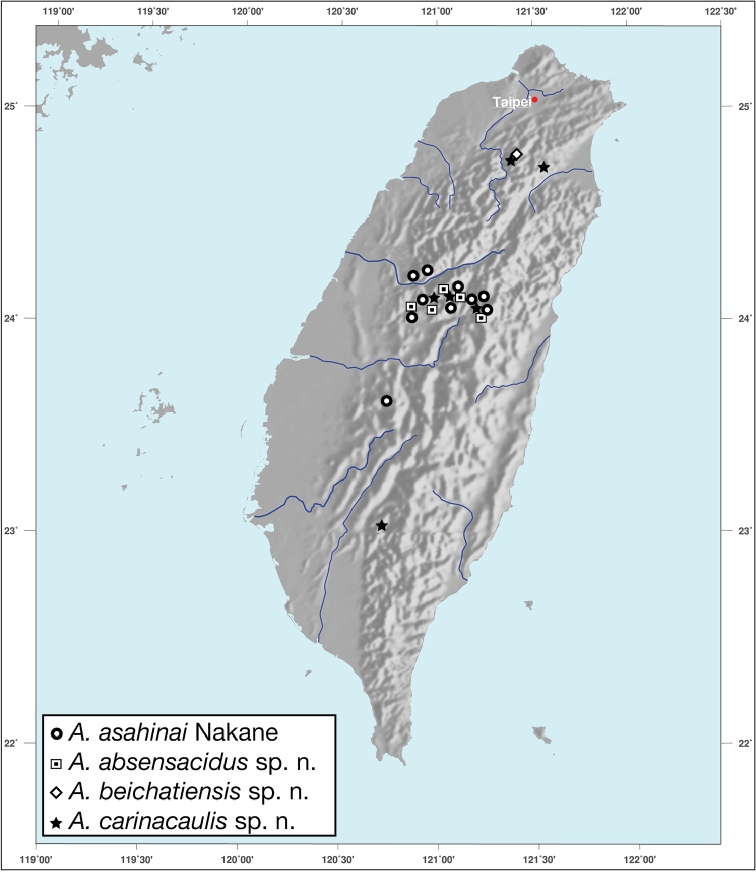
Map showing known localities for species of the genus *Amphimenes* Bates, in Taiwan.

Figs 9 , 10A–D , 11C , 12

########## Specific epithet.

From Latin *carina* and *caulis*, in reference to the strong preapical dorsolateral carina of the male phallus.

########## Types and other material examined.

**Holotype** (male) labeled “Holotype” [circular, ringed with red]; “TAIWAN: Yilan Co./Yuanshan Twp. Fushan/ Botanical Garden Area/ 24.7562N, 121.5924E”; “hand collecting, ~640m/ Acc. Ti-211b, April 16, 2014/ D. Hwang & W. M. Hunting”; “NCHU/100952”. Eleven **paratypes**, eight males and four females. For further details see EH Strickland Virtual Entomology Museum Database.

########## Type locality.

Taiwan. Fushan Botanical Garden, Yilan county.

########## Diagnosis.

Specimens of this species are distinguished from other Taiwanese *Amphimenes* by: being macropterous, having a brunneus elytral suture in apical half, and cross striations on disc of elytra.

########## Description.

OBL 6.50 – 7.83 mm. Length (n = ten males, ten females): head 0.64 – 0.76, pronotum 1.20 – 1.40, elytra 3.42 – 4.50, metepisternum 0.80 – 1.00 mm; width: head 1.12 – 1.36, pronotum 1.64 – 1.92, elytra 2.60 – 3.10, metepisternum 0.46 – 0.60 mm.

*Body proportions*. HW/HL 1.67 – 1.94; PWM/PL 1.34 – 1.42; EL/EW 1.28 – 1.53; ML/MW 1.50 – 2.08.

*Color*. Fig. 9. Dorsum of head piceous; clypeus and labrum rufo-brunneous; pronotum nearly piceous with margins rufo-brunneous; proepipleuron rufo-brunneous to brunneous; antennae rufo-brunneous to brunneous; palpi rufo-brunneous; elytral disc nearly piceous, margins rufo-brunneous, translucent; elytral epipleura rufo-brunneous; thoracic sclerites brunneo-piceous; abdominal sterna rufo-brunneous medially and brunneo-piceous at lateral margins; legs with trochanter and femora rufo-testaceous, tibia rufo-brunneous to brunneo-piceous.

*Microsculpture*. Dorsum of head and pronotum with isodiametric mesh pattern easily visible at 50× magnification; elytra with transverse sculpticells forming elongate and irregular mesh, faintly visible at 50× magnification; striae faintly punctate along length.

*Macrosculpture*. Elytra with cross-striations on intervals, more or less evenly spaced throughout length.

*Pilosity*. Elytra with scattered micro-punctures, setae visible.

*Luster*. Elytra moderately glossy to moderately dull; ventral thoracic sterna and abdominal sterna moderately dull.

*Head*. Labrum with indentation along apical margin, somewhat bilobed; eyes somewhat flattened in appearance, following contour of head.

*Pronotum*. Lateral margins narrow; posterio-lateral margins obtuse to almost right-angled; basal lobe present.

*Elytra*. Humeri broadly rounded; lateral margins nearly parallel.

*Hind wings*. Macropterous.

*Legs*. Meso-tibia with or without several shallow notches from mid-way to base along ventral surface.

*Male genitalia*. Fig. 10A–D. Length 1.32–1.44 mm. Phallus with basal phallic carina (bpc) from base to mid-phallus, decreasing in length from center to right of center when viewed ventrally; left side of shaft moderately enlarged medially, with a strong preapical dorsolateral carina (pdc); endophallus with small internal endophallic sclerite near apex, few distinctive endophallic lobes (el).

*Female genitalia*. Fig. 11C. Width 0.92–0.96mm. Two spermathecae, spermatheca 1 (sp1) and 2 (sp2) with ducts relatively smooth and even throughout length; one spermathecal accessory gland (sg); spermathecal gland duct (sgd) attachment site on spermatheca 1 duct, proximal to attachment site of spermatheca 2; one small node (sdn), near base of spermatheca 2 duct.

########## Habitat, habits, and seasonal occurrence.

The known elevational range of *A.carinacaulis* sp. n. is from 640 to 1850 meters. Adults of this species are found in mixed primary and secondary forest of montane areas. Adults are crepuscular or nocturnal with most activity observed on live tree trunks at night. Specimens have been collected from April to July. All known specimens were hand collected.

########## Geographical distribution.

*Amphimenescarinacaulis* is known only from Taiwan. See Figure 12.

######### Genus Bellavalentis gen. n.

Taxon classificationAnimaliaColeopteraCarabidae

http://zoobank.org/2E546E32-3EC8-44E8-B6FC-96B38FCFFC71

########## Type species.

*Dolichoctiskuzugamii* Shibata, 1967: 65; here designated.

########## Etymology.

From Latin *bella* and *valentis*, in reference to the beautiful and relatively robust elytral form.

########## Recognition.

Size small, elytra broadly rounded and distinctly convex, female genitalic characteristics very distinctive.

########## Included species and remarks.

As of now, *Bellavalentis* includes only one species, *B.kuzugamii.* It appears to us that with further work, all species considered to be part of the “*Dolichoctisstriata* complex” of Baehr (2013a) will form the genus *Dolichoctis* s. str. and other species currently included in the genus *Dolichoctis* will be designated to other taxons.

While there is some variation throughout the genus, the somewhat flattened and at least slightly elongate body form of *Dolichoctis* appears thoughout the striata complex. Additionally, the female genitalic features of *Dolichoctis* (Fig. 71A–D) appear to be very consistent. This type of consistency exists as a general rule, throughout the pericaline lebiines. *Bellavalentiskuzugamii* has gentalic characteristics (Fig. 15A) that are markedly different from all species of *Dolichoctis* encountered.

Material of *B.kuzugamii* labeled by HE Andrewes as being “very near *D.angulicollis*Chaudoir” (1869) was examined. The description of *D.globosa*Andrewes (1930) fits well with the general body form of *B.kuzugamii*. Dissections of the females of these species and others that share similarities in external morphology will be very helpful in determining their placement within these genera.

######### Bellavalentis kuzugamii

Taxon classificationAnimaliaColeopteraCarabidae

(Shibata)

**Figure 13.**
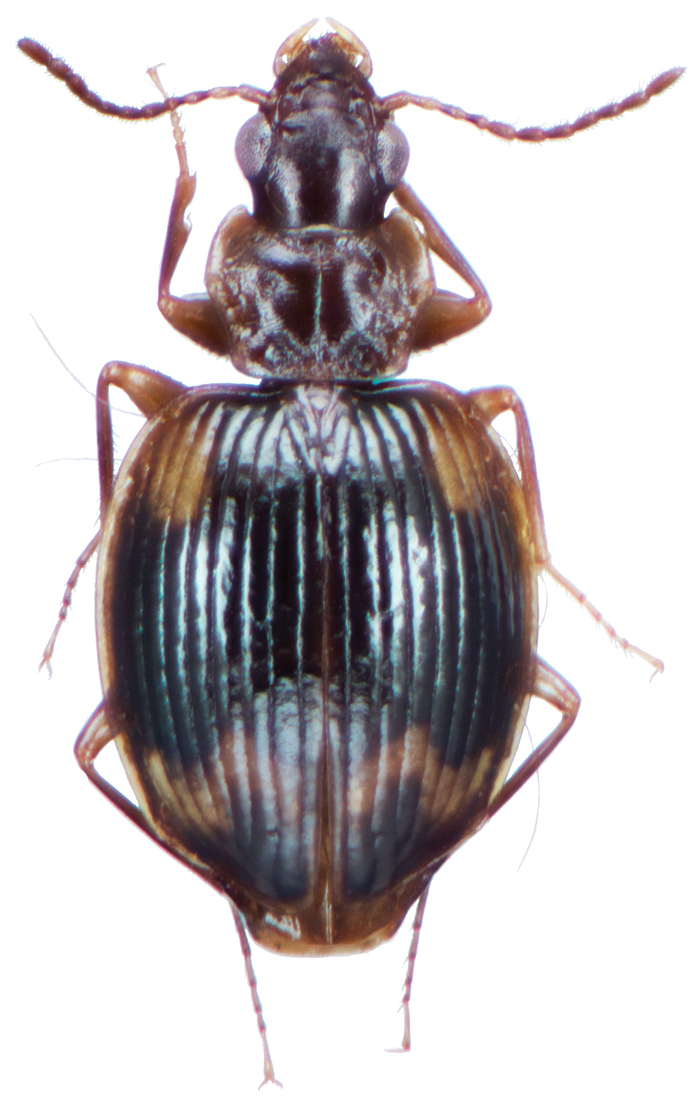
Dorsal habitus and color pattern of *Bellavalentiskuzugamii* (Shibata). (OBL 3.26 mm).

**Figure 14.**
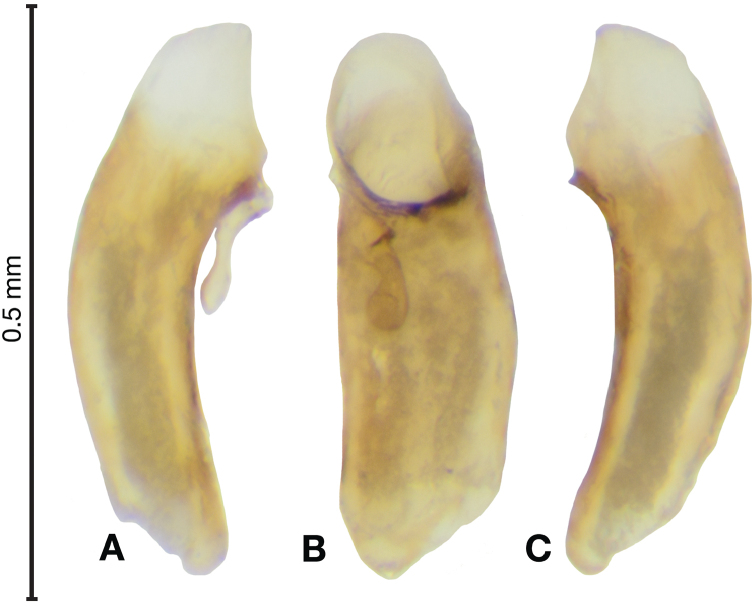
Digital images of male genitalia of *Bellavalentiskuzugamii* (Shibata). **A** right lateral aspect **B** ventral aspect **C** left lateral aspect.

**Figure 15.**
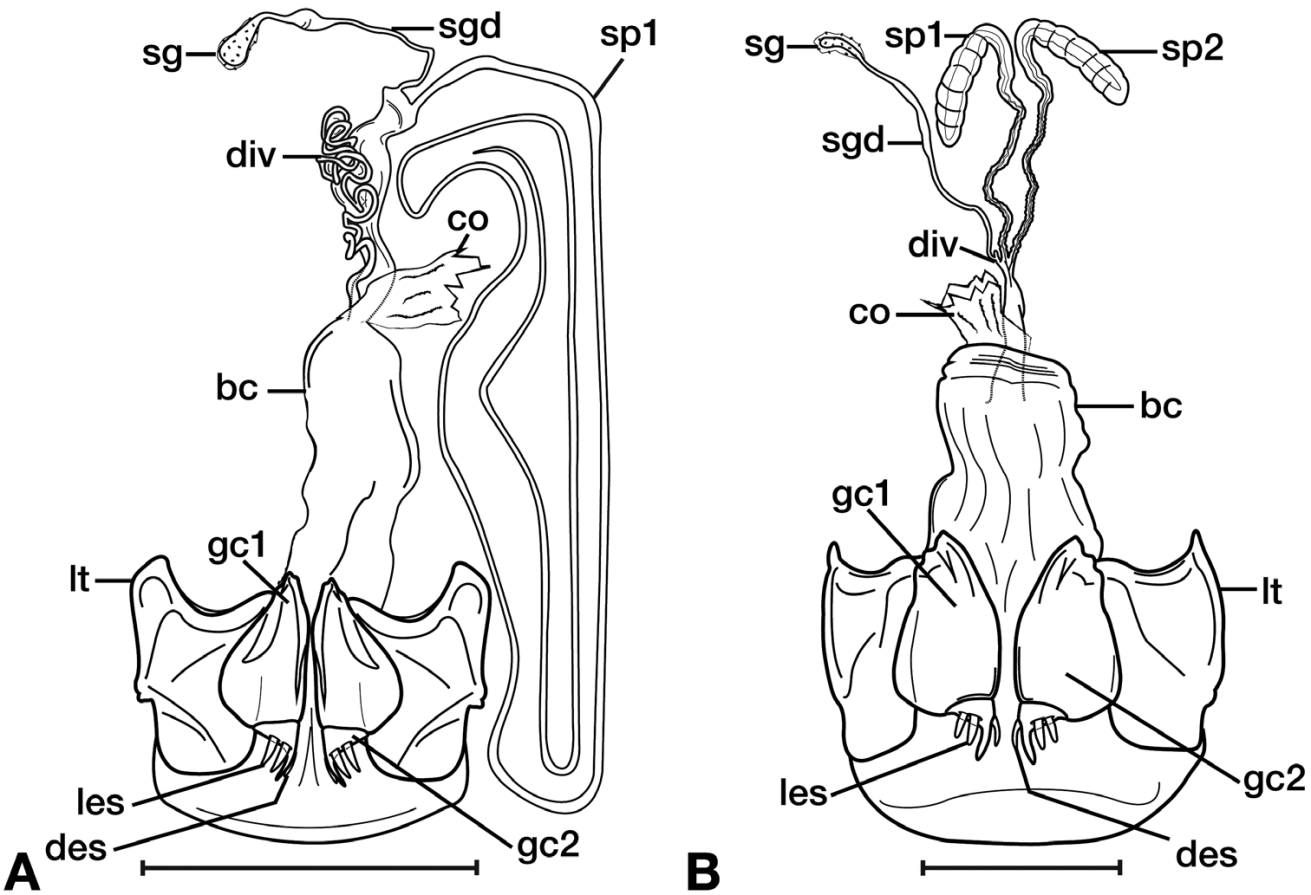
Line drawings of the female reproductive tract, ventral aspect, of **A***Bellavalentiskuzugamii* (Shibata) **B***Brachichilahypocrita* Chaudoir. Legend: **bc** bursa copulatrix; **co** common oviduct; **des** dorsal ensiform setae; **div** diverticulum **gc1** gonocoxite 1; **gc2** gonocoxite 2; **les** lateral ensiform setae; **lt** lateral tergite; **sg** spermathecal gland; **sgd** spermathecal gland duct; **sp1** spermatheca 1; **sp2** spermatheca 2. Scale bars: 0.5 mm.

**Figure 16.**
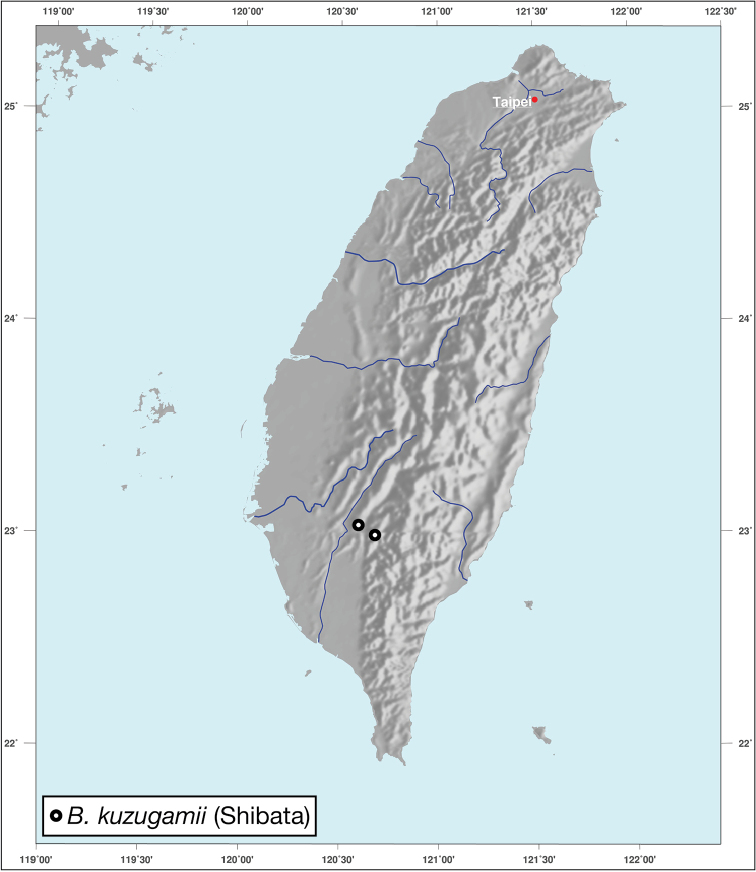
Map showing known localities for species of the genus *Bellavalentis* gen. n., in Taiwan.

Figs 13 , 14A–C , 15A , 16

Dolichoctis kuzugamii Shibata, 1967: 65; Lorenz 2005: 459.

########## Types and other material examined.

**Holotype** (female) labeled “LIUKUEI/TAIWAN/18. IV. 1978/K. KUZUGAMI” [yellow, handwritten]; “HOLOTYPE/Dolichoctis/kuzugamii/Shibata, 1987” [barn door, red]; “NCHU#/101475”. 69 specimens of *B.kuzugamii*: 34 males and 35 females. For further details see EH Strickland Virtual Entomology Museum Database.

########## Type locality.

Taiwan. Kaoshiung City, Maolin District “Liukuei” on the holotype label refers to the Liouguei Research Center.

########## Diagnosis.

This species is readily separated from all other Taiwanese pericalines by its small size, as well as distinctively rounded and convex elytra.

########## Redescription.

OBL 3.16 – 3.76 mm. Length (n = ten males, ten females): head 0.28 – 0.40, pronotum 0.52 – 0.64, elytra 1.84 – 2.24, metepisternum 0.28 – 0.44 mm; width: head 0.60 – 0.74, pronotum 0.80 – 1.00, elytra 1.52 – 1.92, metepisternum 0.24 – 0.32 mm.

*Body proportions*. HW/HL 1.67 – 2.25; PWM/PL 1.50 – 1.69; EL/EW 1.13 – 1.21; ML/MW 1.13 – 1.43.

*Color*. Fig. 13. Various. Dorsum of head, clypeus and labrum brunneo-testaceous to brunneo-piceous, clypeus and labrum somewhat darker centrally, antennae and palpi testaceous to rufo-brunneous; disc of pronotum brunneous to rufo-brunneous, lateral margins somewhat translucent, slightly lighter in color; elytral disc piceous, with four testaceous to brunneo-testaceous maculae, two anterior and two posterior, anterior macula near humerus, from stria 6 to elytra margin, subsquare in shape, posterior macula, extended from interval 1 to interval 8, closest to base in interval 4, closest to apex in interval 6 and 7; margins of elytra somewhat translucent, brunneous to rufo-brunneous; ventral surface testaceous to brunneo-testaceous, metepisternum darker, apical edge of abdominal sterna rufo-brunneous to piceous; legs with trochanter and femora testaceous, tibia with dorsal surface partially piceous.

*Microsculpture*. Dorsum of head with microsculpture somewhat granulate, easily visible at 50× magnification, slightly transverse to almost isodiametric; pronotum with shallow, transverse mesh pattern; elytra with shallow, transverse sculpticells; ventral surface of head with microsculpture transverse, faintly visible at 50×; prosternum, proepipleuron, mesepisternum, and metepisternum with sculpticells forming a shallow transverse mesh.

*Macrosculpture*. Dorsum of head smooth; pronotum with shallowly rugulose to smooth, disc with one small circular impression medially on each side; elytra with intervals somewhat flat.

*Fixed setae.* Two pairs of supraorbital setae; clypeus with two lateral setae; labrum with six setae along apical margin; one pair of suborbital setae; pronotum with two pairs of setae, one at base of lateral margin and one on lateral margin at pronotum max width; elytra with two setae in stria 2, one just before mid-length of elytra and one in apical 1/5; 16 lateral (umbilical) setae in interval 9; Ventral surface with fine, scattered setigerous punctures, two setae on each of abdominal sterna III to VI; two setae along apical margin of sternum VII in males, females with four setae near apical margin of sternum VII.

*Luster*. Head capsule and pronotum moderately glossy; elytra glossy; ventral thoracic sterna and abdominal sterna moderately glossy.

*Head*. Mandibles curved at apex, relatively short, mostly covered by labrum; labrum rounded at apex, longer than wide; mentum with no tooth; eyes suboval; palpi cylindrical, elongate, setose.

*Pronotum*. At least 1.5× wider than long. Disc with one round shallow depression on either side; anterior transverse impression shallow; posterior transverse impression moderately shallow; median longitudinal impression moderately shallow; lateral margins somewhat explanate, apical edge highly emarginate and apico-lateral margins acutely rounded forming distinctive lobes, posterio-lateral margins slightly sinuate, obtuse.

*Elytra*. Humeri broadly rounded, disc distinctly rounded and convex in lateral profile; lateral margins slightly explanate.

*Hind wings*. Reduced.

*Metepisternum*. Subquadrate, least 1.13× longer than wide.

*Legs*. Tarsal claws denticulate, five denticles per claw, males with adhesive vestiture ventrally, two rows of squamo-setae on tarsomeres 2 and 3 of fore-leg.

*Male genitalia*. Fig. 14A–C. Length 0.40 – 0.44 mm. Ostium anopic. Phallus cylindrical but somewhat flattened dorso-ventrally, apical area with very short, bluntly rounded apex, positioned to the right of center in ventral view, endophallus not observed due to small size.

*Female genitalia*. Fig. 15A. Width 0.44 – 0.56 mm. Gonocoxite 2 (gc2) wide at base, narrowing significantly and slightly curved towards apex; two lateral ensiform setae (les) and one dorsal ensiform seta (des) present. Sensory furrow, furrow pegs and associated nematiform setae not observed. Our interpretation of the following characters of this species is based on similar species within the percalines. One diverticulum (div), curled along length and attached to base of spermathecal base; one spermatheca (sp1), narrowing sharply into long, narrow tube-like apex; one spermathecal accessory gland (sg) with attachment site near the narrowing point of the spermatheca.

########## Habitat, habits, and seasonal occurrence.

The known elevational range of *B.kuzugamii* is from 640 to 850 meters. Adults of this species are found in mixed forest of montane areas, and many specimens of this species were all found on deadwood. Specimens have been collected in April and August in Taiwan and the only known method of collection has been hand collecting.

########## Geographical distribution.

*Bellavalentiskuzugamii* is known from Okinawa, Japan and Taiwan. For Taiwan localities see Figure 16.

######### Genus Brachichila

Taxon classificationAnimaliaColeopteraCarabidae

Chaudoir

Brachichila Chaudoir, 1869: 123; Bates 1892: 406; Habu 1967: 112; Lorenz 2005: 456; Fedorenko 2010: 17.
Brachychila Csiki, 1932: 1354 (unjustified emendation); Jedlička 1963: 361; Kirschenhofer 1994: 1011; 1996: 765; 2010: 28.

########## Type species.

*Brachichilahypocrita* Chaudoir, 1869: 123 (monotypic).

########## Type locality.

Hong Kong.

######### Brachichila hypocrita

Taxon classificationAnimaliaColeopteraCarabidae

Chaudoir

Figs 8B , 15B , 18A–D , 19A–C , 20

Brachichila Chaudoir, 1869: 123–124; Lorenz 2005: 456.
Brachychila hypocrita : Csiki, 1354: 1354; Jedlička: 361; Kirschenhofer 1994: 1011; Kirschenhofer 1996: 765; Kirschenhofer 2010: 28.

########## Types and other material examined.

123 specimens of *B.hypocrita*, 67 males and 56 females. For further details see EH Strickland Virtual Entomology Museum Database.

########## Type locality.

Hong Kong.

########## Diagnosis.

This species is readily separated from all other Taiwanese pericalines by having a combination of: a mentum with no tooth, four elytral maculae, an overall body length of more than 6mm and elytra with a single discal seta near the apex of stria 2.

########## Redescription.

OBL 6.3 – 8.2 mm. Length (n = 15 males, 15 females): head 0.64 – 0.78, pronotum 1.12 – 1.44, elytra 3.75 – 4.92, metepisternum 0.9 – 1.16 mm; width: head 1.24 – 1.60, pronotum 1.64 – 2.20, elytra 2.54 – 4.46, metepisternum 0.52 – 0.68 mm.

*Body proportions*. HW/HL 1.79 – 2.23; PWM/PL 1.46 – 1.65; EL/EW 1.28 – 1.51; ML/MW 1.53 – 2.00.

**Figure 17.**
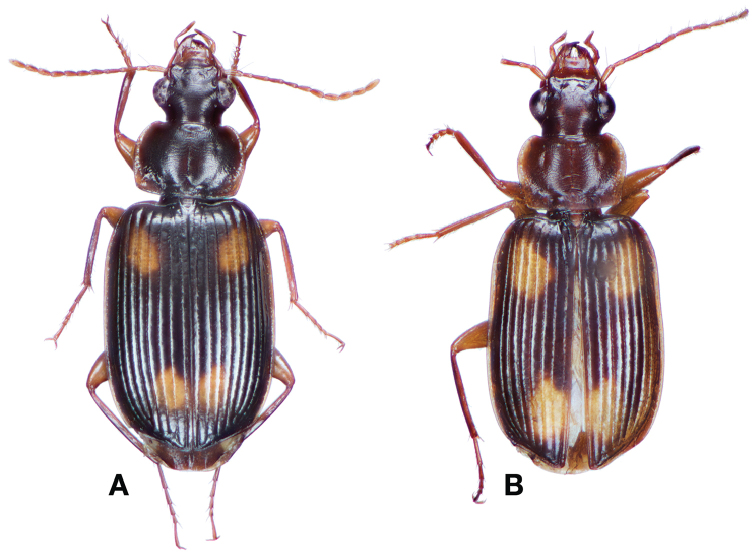
Dorsal habitus and intrapopulation variation of color pattern of *Brachichilahypocrita* Chaudoir **A** small elytral macula (OBL 7.20 mm) **B** large elytral macula (OBL 6. 60 mm).

*Color*. Fig. 17A, B. Dorsum of head and clypeus rufous to brunneo-piceous; labrum, palpi and antennae rufous; pronotum rufo-brunneous to brunneo-piceous with margins somewhat translucent; elytra with disc rufo-brunneous to rufo-piceous, with four testaceous to rufo-testaceous maculae, two anterior and two posterior, anterior macula from outside of interval 3 to outside of interval 7, nearest to base at interval 7 but not touching base, closest to apex in interval 7 but ending in first third of basal portion of elytra, posterior macula from suture (stria 1 in some individuals) to inside of interval 5 (stria 4 in some individuals), closest to base in interval 3 but not extended past apical third of disc, nearest to apex at interval 3–4 but not touching apex, margins of elytra rufo-brunneous, translucent; ventrally with elytral epipleura rufo-brunneous to brunneous; thoracic sclerites and abdominal sterna rufo-testaceous to rufo-brunneous; legs with trochanter and femora testaceous to rufo-testaceous, tibia rufo-testaceous to brunneous.

########## Notes on variation.

Southern specimens are typically lighter in dorsal coloration. Male genitalia typically less sclerotized but this is somewhat variable within populations. Females similar throughout range.

*Microsculpture*. Dorsum of head with isodiametric mesh pattern easily visible at 50× magnification; pronotum with disc isodiametric, meshes somewhat stretched at posteriolateral angles; elytra with shallow, transverse sculpticells faintly visible throughout; ventral surface of head, prosternum, proepipleuron, mesepisternum, and metepisternum with sculpticells forming a moderately transverse mesh.

*Macrosculpture*. Pronotum with disc faintly rugulose to smooth, margins punctate; striae punctate along length.

*Pilosity*. Dorsum of head and pronotum glabrous, ventral surface of head with some to no fine seta visible; striae with punctures each bearing a fine seta hardly visible at 50× magnification; thoracic sclerites and abdominal sterna with scattered fine setae throughout, punctures not visible.

*Fixed setae.* Two pairs of supraorbital setae; clypeus with two lateral setae; labrum with six setae along apical margin; one pair of suborbital setae; ligula with six setae on apical margin between lobes; pronotum with two setae along each margin; elytra with one seta near apex of interval 2; 19 lateral (umbilical) setae in interval 9; two setae on each of abdominal sterna III to VI, two setae along apical margin of sternum VII in males, females with four setae near apical margin of sternum VII.

*Luster*. Head capsule and pronotum moderately dull; elytra moderately glossy to moderately dull; ventral thoracic sterna and abdominal sterna moderately dull.

*Head*. Fig. 8B. Apical margin somewhat emarginate, bilobed to almost rectangular; mentum without tooth; ligula distinctly bilobed (see Habu 1967); eyes moderately convex.

*Pronotum*. Anterior transverse impression shallow, posterior transverse impression deep; basal fovea deep and broad; median longitudinal impression shallow; disc convex, apical angles slightly emarginate, basal angles obtuse, somewhat rounded; lateral margins more explanate towards base, more so in males.

*Elytra*. Striae moderately impressed; elytral disc convex; lateral margin smooth, parallel along length; elytral apices truncate.

*Hind wings*. Macropterous.

*Legs*. Tarsal claws pectinate, three to four denticles per claw. Males with adhesive vestiture ventrally, two rows of squamo-setae on tarsomeres 1–3 of fore-leg; male meso-tibia with two deep notches on ventral surface, near base.

*Abdominal sterna.* Males with abdominal sternum VII faintly emarginate.

**Figure 18.**
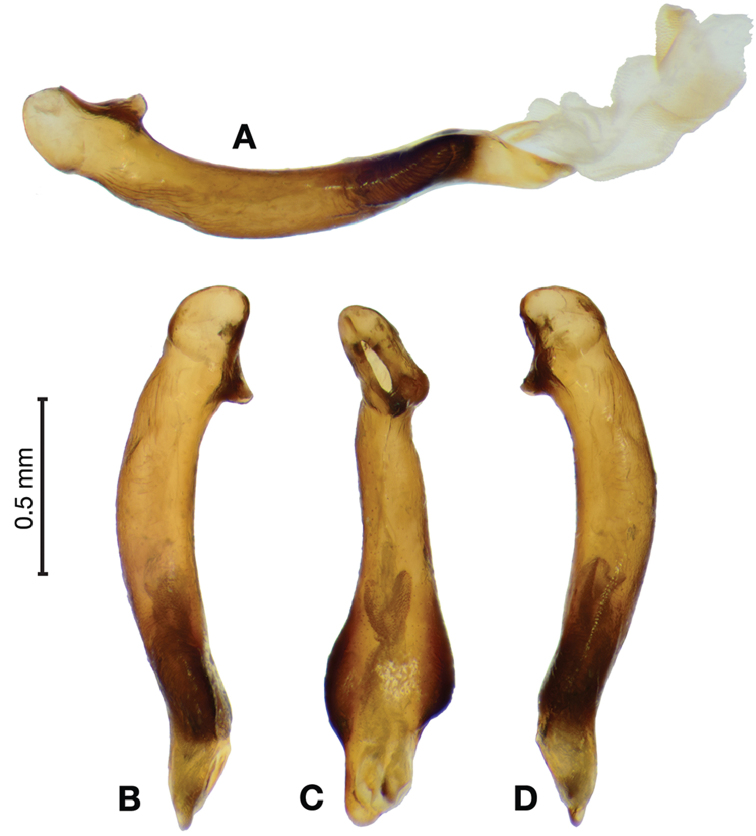
Digital images of male genitalia of *Brachichilahypocrita* Chaudoir. **A** left lateral aspect, endophallus everted **B** right lateral aspect **C** ventral aspect **D** left lateral aspect.

**Figure 19.**
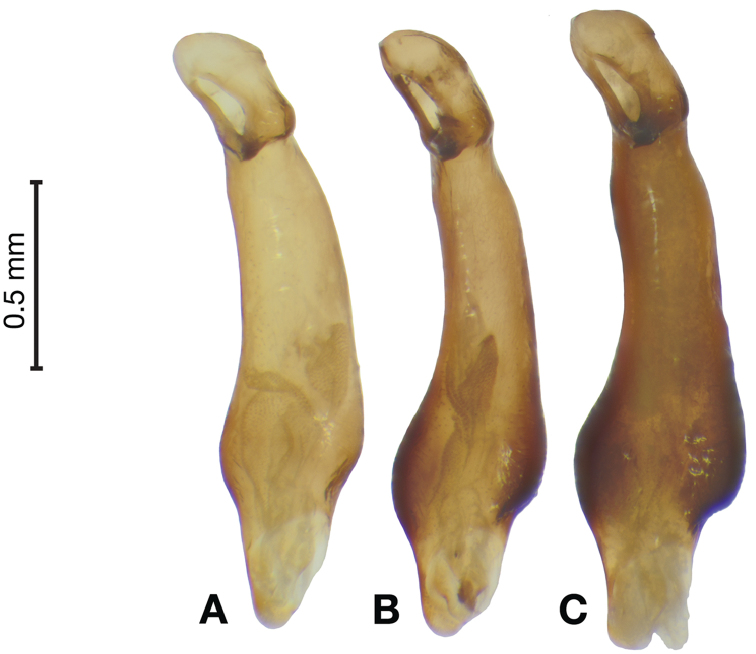
Digital images showing intrapopulation variation of form and scleritization of male phallus of *Brachichilahypocrita* Chaudoir, ventral aspect. **A** lightly sclerotized with slight swelling towards apex **B** moderately sclerotized with moderate swelling towards apex **C** heavily sclerotized with widely expanded apex.

*Male genitalia*. Figs 18A–D, 19A–C. Length 1.60 – 1.74 mm. Ostium left pleuropic. Phallus base cylindrical, narrowing and distinctly curved medially toward apex in lateral view; expanded on both left and right side from median towards apex in ventral view, constricted again before apex; endophallus with several distinctive lobes, no sclerites.

*Female genitalia*. Fig. 15B. Width 0.76 – 0.96 mm. Gonocoxite 2 (gc2) long and narrow; two lateral ensiform (les) setae and one dorsal ensiform seta (des) present; sensory furrow, furrow pegs and associated nematiform setae not observed; two spermathecae; spermatheca 1 (sp1) and 2 (sp2) with ducts narrowly ribbed in appearance, ducts proximal; one spermathecal accessory gland (sg); spermathecal gland duct (sgd) attachment site on associated diverticulum (div).

########## Habitat, habits, and seasonal occurrence.

The known elevational range of *B.hypocrita* in Taiwan is from 200 to 1000 meters. Adults of this species are found in mixed primary and secondary forest of montane areas, as well as disturbed areas. They are crepuscular or nocturnal with most activity observed on tree trunks and deadwood at night. Several specimens were collected from the underside of fallen trees. Specimens have been collected all year round but are most commonly collected from May to October. They readily come to u.v. light. Other methods of collecting include flight intercept trap, malaise trap, sweep netting, hand collecting, and insecticidal fogging at night. Several individuals of *B.hypocrita* were fogged from *Pinusmorrisonicola* Hayata at night.

########## Geographical distribution.

**Figure 20.**
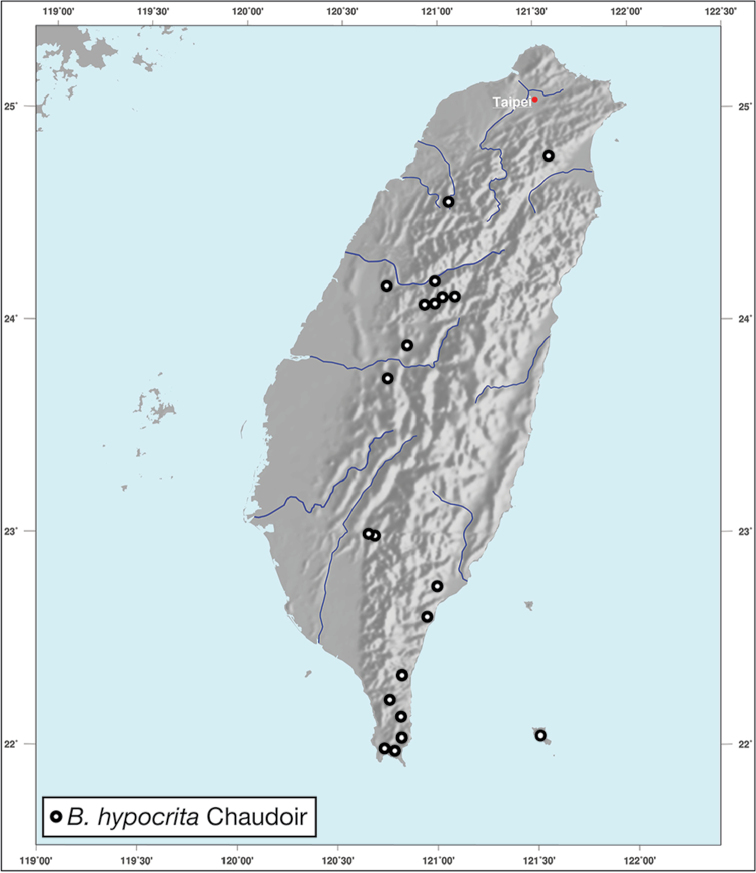
Map showing known localities for species of the genus *Brachichila* Chaudoir, in Taiwan.

*Brachichilahypocrita* is apparently diffuse in Asia. It has been recorded in Japan from Okinawa, the Ryukus (Irimote Island). From Hong Kong, Vietnam, India, and Taiwan. For Taiwan collecting localities see Figure 20.

######## Genus *Catascopus* Kirby

######### Subgenus Catascopus

Taxon classificationAnimaliaColeopteraCarabidae

s. str.

Catascopus Kirby, 1825: 94–97; Schmidt-Göbel 1846: 80; Lacordaire 1854: 145; Chaudoir 1861: 116; Saunders 1863: 455; Bates 1883: 280; 1892: 409; Dupuis 1914: 418; Csiki 1932: 1362; Andrewes 1937: 185; Jedlička 1963: 379; Habu 1967: 77; Straneo 1994: 141; Baehr 1997: 227; 2012: 25; Shpeley and Ball 2000: 112; Lorenz 2005: 454.

########## Type species.

*Catascopushardwickii* Kirby, 1825 (monotypic).

########## Type locality.

India.

########## Recognition of Taiwanese species of *Catascopus* s. str.

*Color.* Various.

*Fixed setae.* Two pairs of supraorbital setae; clypeus with two lateral setae; labrum with six setae along apical margin; one pair of suborbital setae. ; 16–17 lateral (umbilical) setae in interval 9; two setae on each of abdominal sterna III to VI, two setae along apical margin of sternum VII in males, four setae along apical margin of sternum VII in females.

*Head*. Mandibles curved, left mandible slightly more curved and pointed at apex then right mandible, right mandible with additional, small tooth on inside cutting surface near mid-length of mandible; labrum bilobed; mentum with single broad tooth; eyes convex; palpi cylindrical, elongate.

*Elytra*. Humeri broadly rounded; elytral margin shallowly impressed in basal 1/3.

*Hind Wings.* Macropterus.

*Legs*. Tarsal claws smooth. Males with adhesive vestiture ventrally, two rows of squamo-setae on tarsomeres 1–3 of fore-leg.

*Male genitalia*. Ostium left pleuropic. Phallus cylindrical.

*Female genitalia*. Gonocoxite 2 (gc2) wide at base, narrowing sharply toward apex, moderately curved. Sensory furrow, furrow pegs and associated nematiform setae not observed.

######### Key to the Taiwanese species of the subgenus Catascopus Kirby

**Table:** 

1	Apex of elytra not spinous	**2**
–	Apex of elytra distinctively spinous, spines present at apex lateral margin and apex of suture	***C.sauteri* Dupuis**
2	Striae moderately impressed, intervals convex	**3**
–	Striae distinctively faint, intervals flat with exception of carinate inside edge of interval 7	***C.asaharti* sp. n.**
3	Both intervals 5 and 7 broadly rounded to carinate along portions of length, dorsal surface typically metallic purple, or green	***C.ignicinctus* Bates**
–	Only interval 7 distinctly carinate on inside margin, always dorsally metallic green	***C.viridiorchis* sp. n.**

########## Catascopus (s. str.) asaharti sp. n.

Taxon classificationAnimaliaColeopteraCarabidae

http://zoobank.org/B1CE7B67-8D5C-4BE7-B83E-47EF7A349B1B

**Figure 21.**
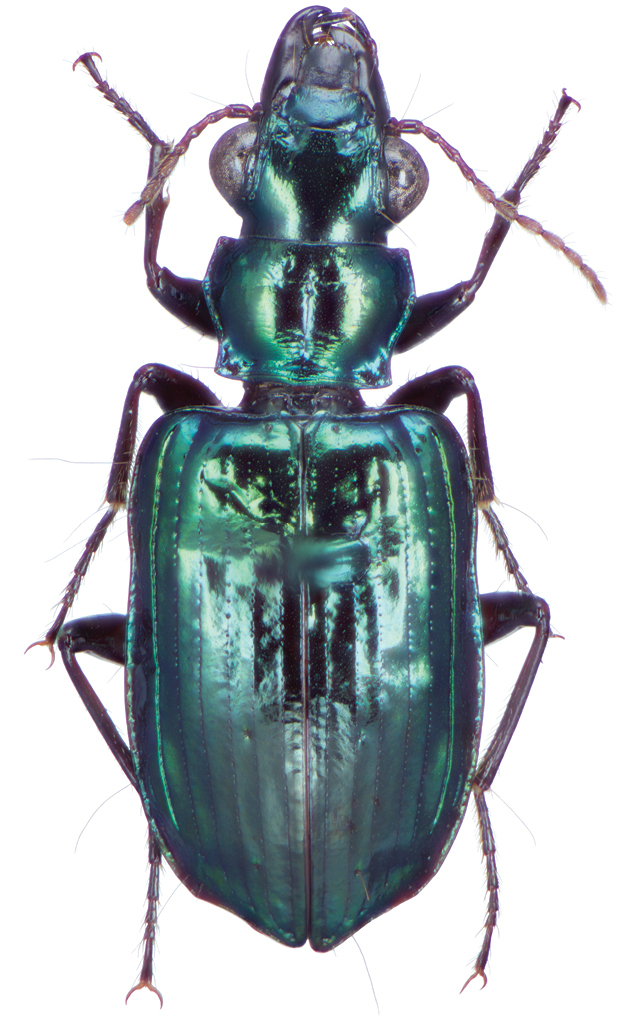
Dorsal habitus and color pattern of *Catascopus* (*s. str.*) *asaharti* sp. n. (OBL 8.75 mm).

**Figure 22.**
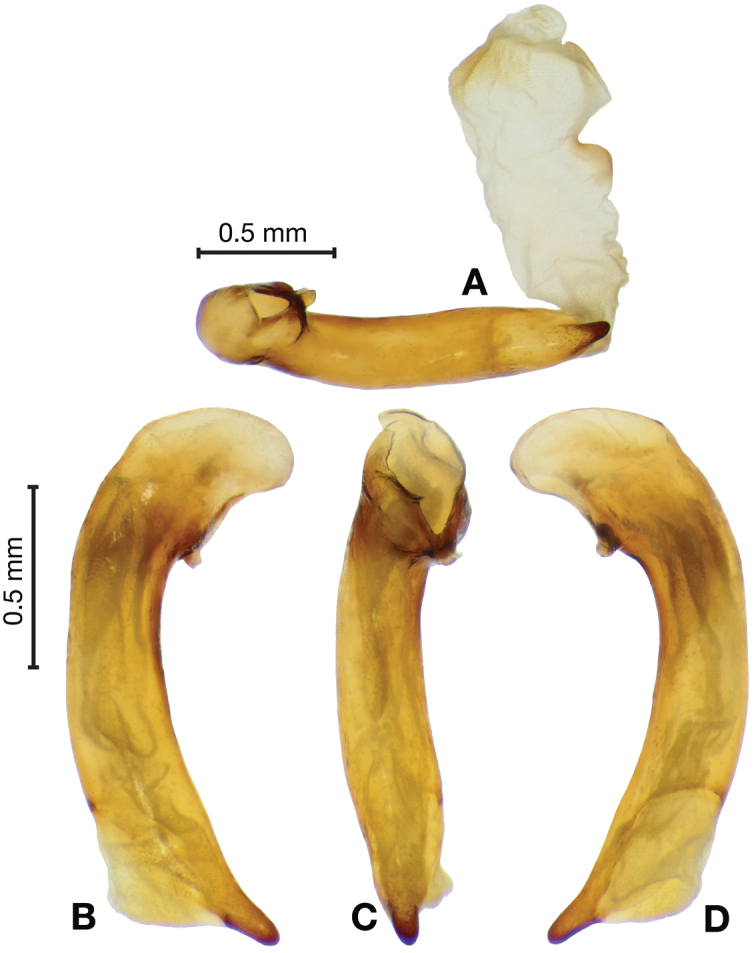
Digital images of male genitalia of *Catascopus* (*s. str.*) *asaharti* sp. n. **A** ventral aspect, endophallus everted **B** right lateral aspect **C** ventral aspect **D** left lateral aspect.

**Figure 23.**
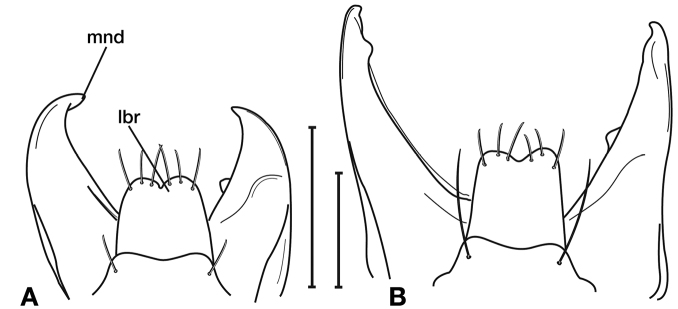
Line drawings comparing mandible form of **A***Catascopus* (*s. str.*) *asaharti* sp. n. **B**Catascopus (Catascopoides) horni Jedlička. Legend: **lbr** labrum; **mnd** mandible. Scale bars: 0.5 mm.

Figs 21 , 22A–D , 23A , 35

########### Specific epiphet.

This species is named after the first author’s son, Asa Hart Hunting, who was born in Taiwan while collecting material for this work. Both specimens of this species were collected at the same locality that we took him for his first collecting trip when he was two weeks old.

########### Types and other material examined.

**Holotype** (male) labeled “Holotype” [circular, ringed with red]; “TAIWAN: Taichung Co./Heping Township, Xueshan/Rd. km 32.5, Dasyueshan/Nat. For. Rec. Area/24.2315N, 120.9784E”; “bridge lights, Acc. Ti-81b/May 9, 2011, 2000 m/Coll. W. M. Hunting”; “NCHU# 101731”. **Paratype** (Male) labeled: “Paratype” [circular, ringed with yellow]”; “TAIWAN: Taichung Co./Heping Township, Xueshan/Rd. km 34, Dasyueshan/Nat. For. Rec. Area/24.2383N, 120.9779E”; “u.v. light, Acc. Ti-81c/ May 9, 2011, 2030 m/Coll. W. M. Hunting”; NCHU# 101732.

########### Type locality.

Taiwan: Taichung county, Heping township, Dasyueshan National Forest Recreation Area.

########### Diagnosis.

Specimens of this species are easily distinguished from all other described species by a combination of the almost flat elytral intervals, except interval 6 which is carinate on the inside margin; and distinctly flat striae.

########### Description.

OBL 8.75 – 8.83 mm. Length (two males): head 0.88 – 0.90, pronotum 1.28 –1.30, elytra 5.00 – 5.08, metepisternum 1.28 – 1.32 mm; width: head 2.08 – 2.12, pronotum 0.08, elytra 3.16 – 3.33, metepisternum 0.64 mm.

*Body proportions*. HW/HL 2.36 – 2.37; PWM/PL 1.54 – 1.56; EL/EW 1.53 – 1.58; ML/MW 2.00 – 2.06.

*Color*. Fig. 21. Dorsum of head and clypeus dark metallic green; labrum, antennae and palpi rufo-brunneous; disc of pronotum dark metallic green; elytral disc dark metallic green; ventral surface rufo-brunneous to rufo-piceous; legs with trochanter, femora and tibia rufo-brunneous to rufo-piceous.

*Microsculpture*. Dorsum of head and pronotum with microsculpture isodiametric, faintly visible in patches at 50× magnification; elytra with faintly visible, +/- isodiametric sculpticells; ventral surface of head and majority of body with microsculpture not visible at 50×; epipleuron of elytra and portions of abdominal sternum IV with faint, +/- isodiametric sculpticells.

*Macrosculpture*. Dorsum of head, clypeus and pronotum disc with very fine, randomly scattered setigerous punctures, setae almost not visible in side view, at 50×; pronotum with anterior transverse impression very shallow; elytra with interval 7 carinate on inside margin nearest to stria 6, from just behind base to 5/6 of the elytra length; carinate the entire length; all other intervals distinctly flat, with very fine, randomly scattered, setigerous punctures throughout; striae faintly impressed, more visible due to being evenly punctate along length. Ventrally with very fine, randomly scattered setigerous punctures.

*Fixed setae.* Pronotum with two pairs of setae, one at base of lateral margin and one on lateral margin at pronotum max width; elytra with one seta at basal quarter of interval 3, one seta in interval 3 just beyond mid-length, one seta in apical quarter of interval 3; femur of meso-leg with several moderately long seta on dorsal surface and two long setae ventrally in males.

*Luster*. Glossy.

*Head*. Fig. 23A.

*Pronotum*. Anterior transverse impression shallow; posterior transverse impression deep, median longitudinal impression moderately deep; lateral margins constricted just before mid-way toward apex, becoming parallel at ¾ length; posterio-lateral margins almost right-angled.

*Metepisternum*. Elongate, at least 2× longer than wide.

*Male genitalia*. Fig. 22A–D. Length 1.52 mm. Phallus width consistant along length; apical area, short with rounded point at apex; endophallus moderately long, with distinctive widening from base to apex.

*Female genitalia*. Unknown

########### Habitat, habits, and seasonal occurrence.

The known elevation of *C.asaharti* sp. n. is 2000 meters. Adults of this species are found in mixed forest of montane areas. Little is known of the habits of this species. Adults are crepuscular or nocturnal and both specimens of the species were collected on a single night in the month of May. One was collected from a u.v. light sheet and the other was collected from a bridge light approximately one kilometer away. During a three-year period, more than 60 collection times did not reveal any more specimens.

########### Geographical distribution.

*Catascopusasaharti* is only known from Taiwan. See Figure 35.

########## Catascopus (s. str.) ignicinctus

Taxon classificationAnimaliaColeopteraCarabidae

Bates

**Figure 24.**
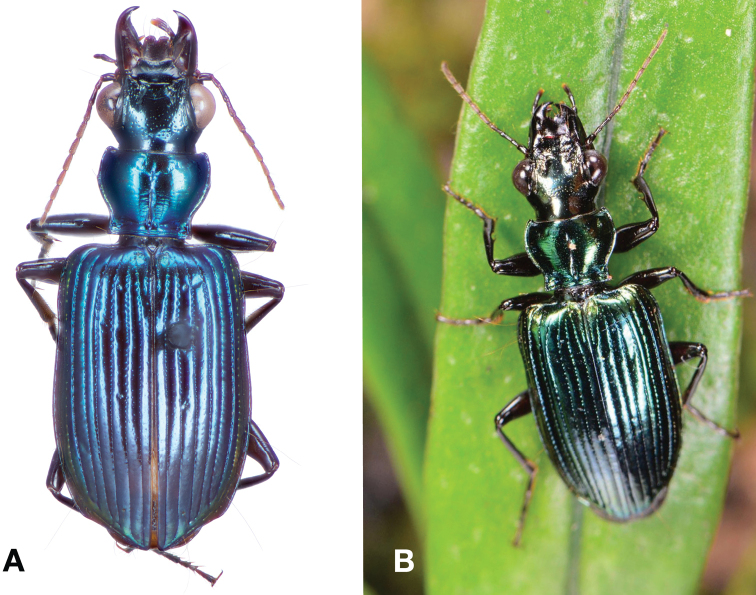
Dorsal habitus and color pattern of *Catascopus* (*s. str.*) *ignicinctus* Bates. **A** common purple metallic morph (OBL 10.85 mm) **B** live image of green color morph.

**Figure 25.**
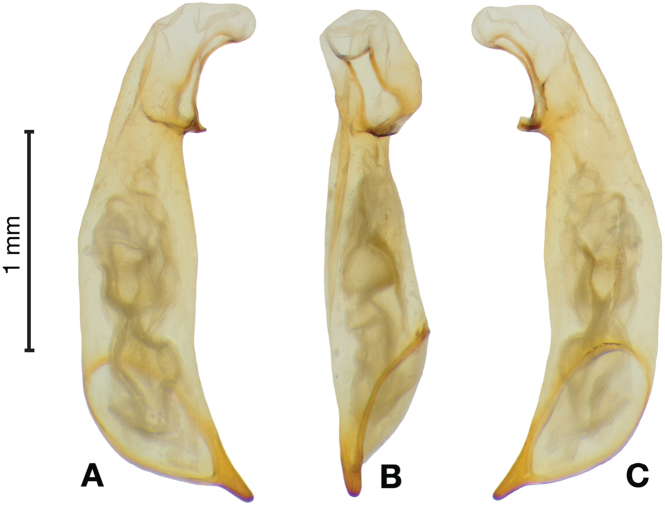
Digital images of male genitalia of *Catascopus* (*s. str.*) *ignicinctus* Bates, teneral. **A** right lateral aspect **B** ventral aspect **C** left lateral aspect.

Figs 24A, B , 25A–C , 34A , 35

Catascopus ignicinctus Bates, 1883: 280; Csiki 1932: 1365; Jedlička 1963: 386; Habu 1976: 79; Straneo 1994: 166; Lorenz 2005: 454.
Catascopus szekessyi Jedlička, 1952: 81.

########### Types and other material examined.

Six specimens of *C.ignicinctus*, two males and four females. For further details see EH Strickland Virtual Entomology Museum Database.

########### Type locality.

Japan: “Yuyama and Konose”.

########### Taxonomic notes.

*Catascopusaequatus* was previously recorded from Taiwan (Kano 1930; Miwa 1931) but is not present and was likely mistaken for *C.ignicinctus*, which is present in Taiwan and similar in general form but easily distinguished from *C.aequatus* by the smoothly rounded elytral apices (*C.aequatus* having distinctive spines).

########### Diagnosis.

Specimens of this species are easily distinguished from other species of Taiwanese *Catascopus* by the typically metallic purple dorsal coloration and having both intervals 5 and 7 with broadly convex to carinate portions along the length of the elytra.

########### Redescription.

OBL 10.5 – 12.83 mm. Length (two males, four females): head 1.08 – 1.32, pronotum, 1.80 – 2.04, elytra 6.50 – 7.20, metepisternum 1.80 – 1.52 mm; width: head 2.44 – 2.88, pronotum 2.36 – 2.76, elytra 4.00 – 4.33, metepisternum 0.64 – 0.88 mm.

*Body proportions*. HW/HL 2.09 – 2.57; PWM/PL 1.29 – 1.41; EL/EW 1.50 – 1.65; ML/MW 2.00 – 2.65.

*Color*. Various. Fig. 24A, B. Dorsum of head metallic purple to almost black, rarely metallic green (see Green morph below); clypeus and labrum rufo-brunneous to piceous; antennae with segments 1–4 rufo-brunneous to rufo-piceous, segments 5–11 rufo-piceous to rufo-testaceous; palpi rufo-brunneous to rufo-piceous; disc of pronotum metallic purple, margins metallic purple-piceous with faint cupreous margin, median longitudinal impression faintly cupreous along length; elytral disc metallic purple, margins (from interval 7) and outside margin of elytral suture cupreous; proepipleuron, prosternum and elytral epipleura rufo-piceous to piceous; thoracic sclerites rufo-piceous to piceous; abdominal sterna rufo-brunneous to piceous; legs with trochanter, femora and tibia rufo-piceous.

*Green morph.* The green morph of this species is identical in all characters except coloration of head, dorsum of pronotum, and disc of elytra are all metallic green.

*Microsculpture*. Dorsum of head with microsculpture faintly visible at 50× magnification, isodiametric; pronotum with transverse mesh pattern faintly visible at 50× magnification; elytra with shallow, moderately transverse sculpticells; ventral surface of head with microsculpture transverse, faintly visible at 50×; prosternum, proepipleuron, mesepisternum, and metepisternum with sculpticells forming a shallow transverse mesh.

*Macrosculpture*. Dorsum of head with disc smooth centrally, shallow impressions between eyes, few shallow furrows from front of eye to behind clypeus base, one-two deep furrows along contour of eye, longest ending at basal supraorbital setae; scattered punctation from clypeus to constriction of head, shallower centrally, not confluent; pronotum with several shallow lateral impressions from apex to baso-lateral depression, fine scattered punctures throughout; elytra with intervals 1–4 and 6 moderately flat, interval 5 broadly convex from behind shoulder down ¾ of elytra length, interval 7 carinate on inside margin nearest to stria 6; striae punctate along length; ventrally: prosternum, prosternal process, mesosternum, mesocoxa and mesosternal intercoxal process and hind coxa with scattered, shallow punctures; baso-lateral portion of metasternum with deeper, scattered punctures; abdominal sterna with scattered, shallow punctures.

*Fixed setae.* Two pairs of supraorbital setae; clypeus with two lateral setae; labrum with six setae along apical margin; one pair of suborbital setae; fore femur of males and females with two fixed setae in basal half; pronotum with two pairs of setae, one at base of lateral margin and one on lateral margin at pronotum max width; elytra with one seta at basal quarter of interval 3, one seta in interval 3 at mid-length, one seta in apical quarter of interval 3.

*Luster*. Head capsule, pronotum and elytra moderately glossy to glossy; ventral thoracic sterna and abdominal sterna moderately glossy.

*Head*. Labrum bilobed, left lobe slightly longer than right lobe in some specimens.

*Pronotum*. No more than 1.41× wider than long. Anterior transverse impression shallow; posterior transverse impression and median longitudinal impression moderately deep; apical margin narrowly curved forming short, acute latero-apical lobes; lateral margins constricted in basal 1/3; posterio-lateral margins almost right-angled.

*Male genitalia*. Fig. 25A–C. Length 2.20 – 2.44 mm. Ostium with relatively large opening. Phallus narrowest at base of shaft, somewhat expanded on left side from median towards ostium in ventral view; apical area, a short rounded point, somewhat narrow, slightly curved ventrally; endophallus not observed in detail as both males available were too teneral to evert.

*Female genitalia*. Fig. 34A. Width 1.48 – 1.64 mm. Gonocoxite 2 (gc2) with apices spatulate when observed in lateral view; three lateral ensiform setae (les) and one dorsal ensiform seta (des) present (not visible from dorsal view). One spermatheca (sp1) long and narrow; one spermathecal accessory gland (sg) long and narrow; spermathecal gland duct (sgd) relatively long, attachment site near base of spermathecal gland.

########### Habitat, habits, and seasonal occurrence.

The known elevational range of *C.ignicinctus* is from 480 to 700 meters. Adults of this species are found in mixed primary and secondary forest of montane areas. Adults are crepuscular or nocturnal with most activity observed on trunks of fallen or dying trees at night. Specimens have been collected from July to late September. Methods of collecting include m.v. light sheet and hand collecting.

########### Geographical distribution.

*Catascopusignicinctus* is known from southern Japan, Laos, China, and Taiwan. For Taiwan localities see Figure 35.

########## Catascopus (s. str.) sauteri

Taxon classificationAnimaliaColeopteraCarabidae

Dupuis

**Figure 26.**
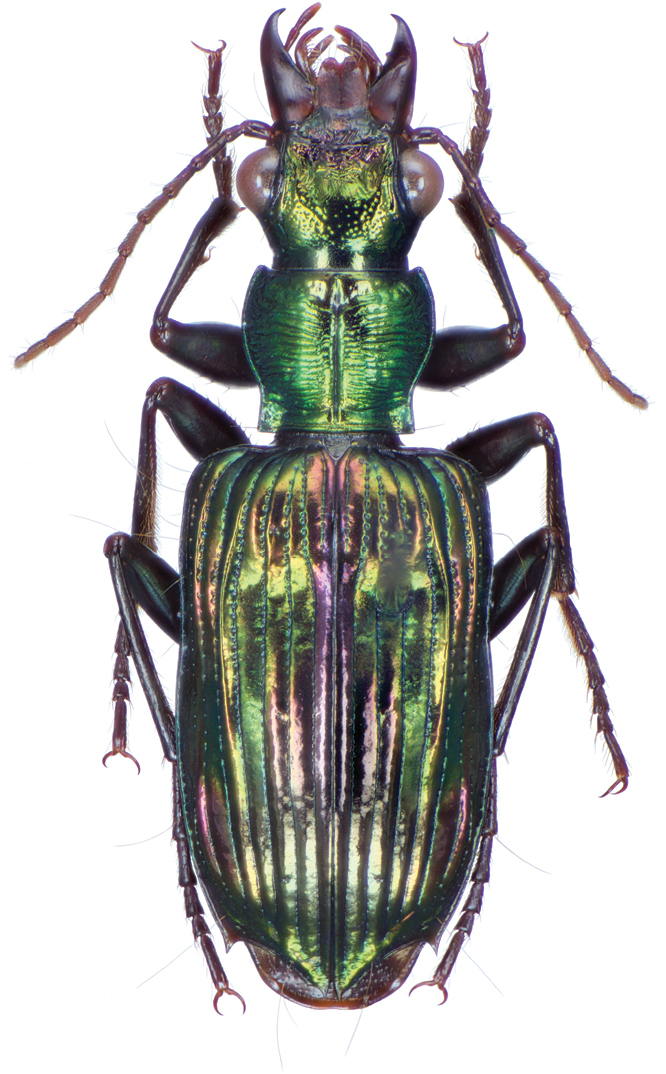
Dorsal habitus and color pattern of *Catascopus* (*s. str.*) *sauteri* Dupuis. (OBL 13.13 mm).

**Figure 27.**
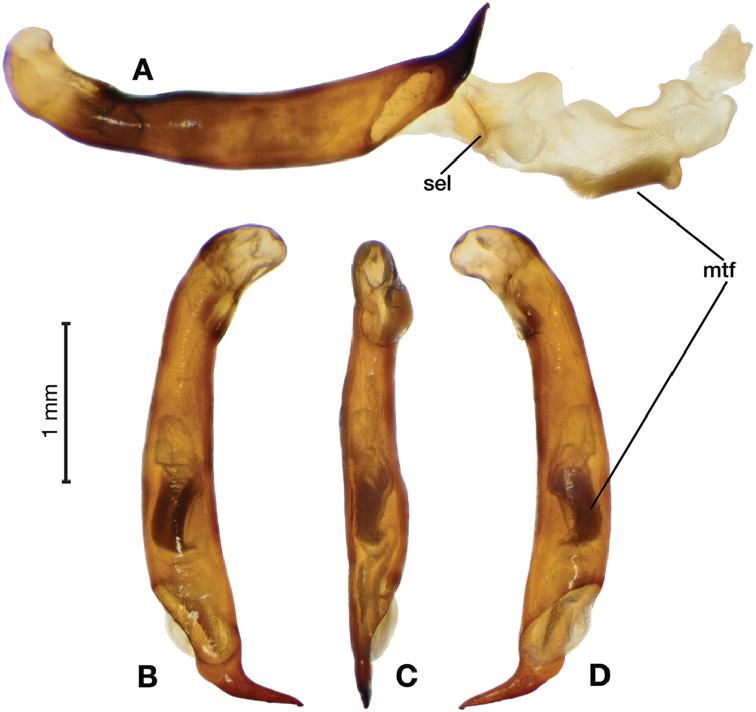
Digital images of male genitalia of *Catascopus* (*s. str.*) *sauteri* Dupuis. **A** right lateral aspect, endophallus everted **B** right lateral aspect **C** ventral aspect **D** left lateral aspect. Legend: **mtf** microtrichial field **sel** scleritization of endophallic lobe (fully visible from left lateral aspect).

Figs 26 , 27A–D , 33B , 34B , 35

Catascopus sauteri Dupuis, 1914: 419: Jedlička 1963: 392; Lorenz 2005: 454.

########### Types and other material examined.

**Holotype** (female) labeled “Typus”; “Syntypus”; “Holotypus” [rectangular, red paper]; “Kosempo/Formosa/H. Sauter”; “22. VII.”; “Catascopus/sauteri Dupuis/Dupuis det.” 25 specimens: 17 males and 8 females. For further details see EH Strickland Virtual Entomology Museum Database.

########### Type locality.

“Formosa, Hoozan”. Hoozan refers to Fengshan, Kaohsiung county.

########### Diagnosis.

Specimens of this species are easily distinguished from other species of Taiwanese *Catascopus* having being metallic green and having elytral spines on both the apex of the lateral margins and the suture.

########### Redescription.

OBL 12 – 16 mm. Length (n = 17 males, 8 females): head 1.12 – 1.40, pronotum, 2.04 – 2.52, elytra 7.17 – 8.66, metepisternum 1.52 – 2.20 mm; width: head 2.76 – 3.32, pronotum 2.48 – 3.04, elytra 4.10 – 4.83, metepisternum 0.72 – 0.92 mm.

*Body proportions*. HW/HL 2.26 – 2.55; PWM/PL 1.18 – 1.29; EL/EW 1.65 – 1.82; ML/MW 1.90 – 2.55.

*Color*. Fig. 26. Dorsum of head metallic green; clypeus and mentum rufo-brunneous to piceous; antennae with articles 1–4 rufo-piceous to piceous, articles 5–11 rufo-brunneous to brunneous, apex of article 11 testaceous; palpi rufo-brunneous to rufo-piceous; disc of pronotum metallic green, margins metallic green to metallic purple, basolateral impressions metallic green to metallic purple; elytra metallic green, margins and elytral suture metallic green to metallic purple; proepipleuron, prosternum and elytral epipleura aeneous to piceous; thoracic sclerites rufo-piceous to piceous; abdominal sterna rufo-brunneous to piceous; legs with trochanter and femora rufo-piceous, tibia rufo-piceous to piceous.

*Microsculpture*. Dorsum of head with microsculpture faintly visible at 50× magnification, +/- isodiametric; pronotum with transverse mesh pattern faintly visible at 50× magnification; elytra with shallow, transverse sculpticells on majority of disc, near isodiametric near apex of disc; transverse microsculpture in basal third depression of striae parallel to elytral margins, easily visible at 50× magnification; ventral surface of head with microsculpture transverse, faintly visible at 50×; prosternum, proepipleuron, mesepisternum and metepisternum with sculpticells forming a shallow transverse mesh.

*Macrosculpture*. Dorsum of head rugulose laterally between eyes from basal supraorbital setae to clypeus, smooth centrally, deep scattered punctation from apical lateral setae to constriction of head, some confluent; pronotum rugulose, several lateral striations from apex to baso-lateral depression, fine scattered punctures throughout; elytra with one impression in basal 1/3 of disc, laterally from basal fixed setae of interval 3 to middle fixed setae of interval 3, horizontally from first stria to carina of interval 5; most intervals moderately flat, interval 5 carinate from just beyond basal fixed seta of interval 3 to apical fixed setae of interval 3, interval 7 carinate in basal third, intervals 7 and 8 distinctly carinate and rounded at apical 1/4, disrupting normal contour of elytra; striae punctate along length; ventrally: prosternum, prosternal process, mesosternum, mesocoxa and mesosternal intercoxal process, basal portion of metasternum and hind coxa with shallow, scattered, setigerous punctures; abdominal sterna with scattered, shallow punctures.

*Pilosity*. Dorsum of head, pronotum and disc of elytra with scattered micro-punctures; ventrally: prosternum, prosternal process, mesosternum, mesocoxa and mesosternal intercoxal process and metasternum with short to moderately long, blonde, setae associated with moderately shallow punctures; abdominal sterna with unevenly scattered setigerous punctures.

*Fixed setae.* Fore femur of males and females with two fixed setae in basal 1/3; pronotum with one seta at base of lateral margin; elytra with one seta at basal quarter of interval 3, one seta in interval 3 at mid-length, one seta in apical quarter of interval 3.

*Luster*. Head capsule, pronotum and elytra moderately glossy to glossy; ventral thoracic sterna and abdominal sterna moderately glossy.

*Pronotum*. Anterior transverse impression moderately deep; posterior transverse impression and median longitudinal impression deep; apical margin narrowely curved forming short, acute latero-apical lobes; lateral margins sinuate toward base; posterio-lateral margins almost right-angled.

*Elytra*. Humeri broadly rounded; elytral margin shallowly impressed in basal 1/3, elytral apices each with one small lateral spine and two apical spines, inside apical spine always longer than outside apical spine.

*Male genitalia*. Fig. 27A–D. Length 2.70 – 3.20 mm. Phallus narrowest at base of shaft, somewhat expanded on left side from median towards ostium in ventral view; apical area, long and narrow, sharply curved ventrally, curve visible in left and right view; apex distinctive, most constricted just before apex, tip curved and flattened; endophallus relatively long, some sclerotization evident on basal endophallic lobe when viewed from left lateral aspect; one large microtrichial field (mtf) on dorsal surface, from midway of endophallus to just beyond apical endophallic lobe; microtrichia fine and densely packed.

*Female genitalia*. Figs 33B, 34B. Width 1.48 – 1.68 mm. Gonocoxite 2 (gc2) with two lateral ensiform setae (les) and one dorsal ensiform seta (des) present, apical lateral ensiform setae longer and more spatulate then basal. Bursa copulatrix moderately textured, some infoldings; +/- circular sclerite (bsc) (type 1) internally at base, between apex of lateral tergites, not visible in dorsal view of most specimens and difficult to observe in others due to lack of pigmentation, differing from the bursal sclerite of *Horniulusandrewesi* Jedlička (Fig. 94B); one spermatheca (sp1) long and narrow; one spermathecal accessory gland (sg) long and narrow; spermathecal gland duct (sgd) highly variable in length, ranging from approximately 1.0 mm to 4.0 mm, attachment site on associated diverticulum (div).

########### Habitat, habits, and seasonal occurrence.

The known elevational range of *C.sauteri* is from 500 to 1275 meters. Only a few specimens have been collected over 1000 meters with the majority being collected between 600 and 800 meters. Adults of this species are found in mixed primary and secondary forest of montane areas. Adults are crepuscular or nocturnal with most activity observed on trunks of fallen or dying trees at night. Specimens have been collected from March to December but are most commonly collected from July to December. Methods of collecting include light trap, u.v. light and hand collecting. Adults are very fast runners and it was observed that when they are lit by flashlight or headlamp at night, they will quickly run to the dark side of the tree.

########### Geographical distribution.

*Catascopussauteri* is known only from Taiwan. For collecting localities see Figure 35.

########## Catascopus (s. str.) viridiorchis sp. n.

Taxon classificationAnimaliaColeopteraCarabidae

http://zoobank.org/D52D8CBB-ACE1-4930-87D5-6F368EB115BC

**Figure 28.**
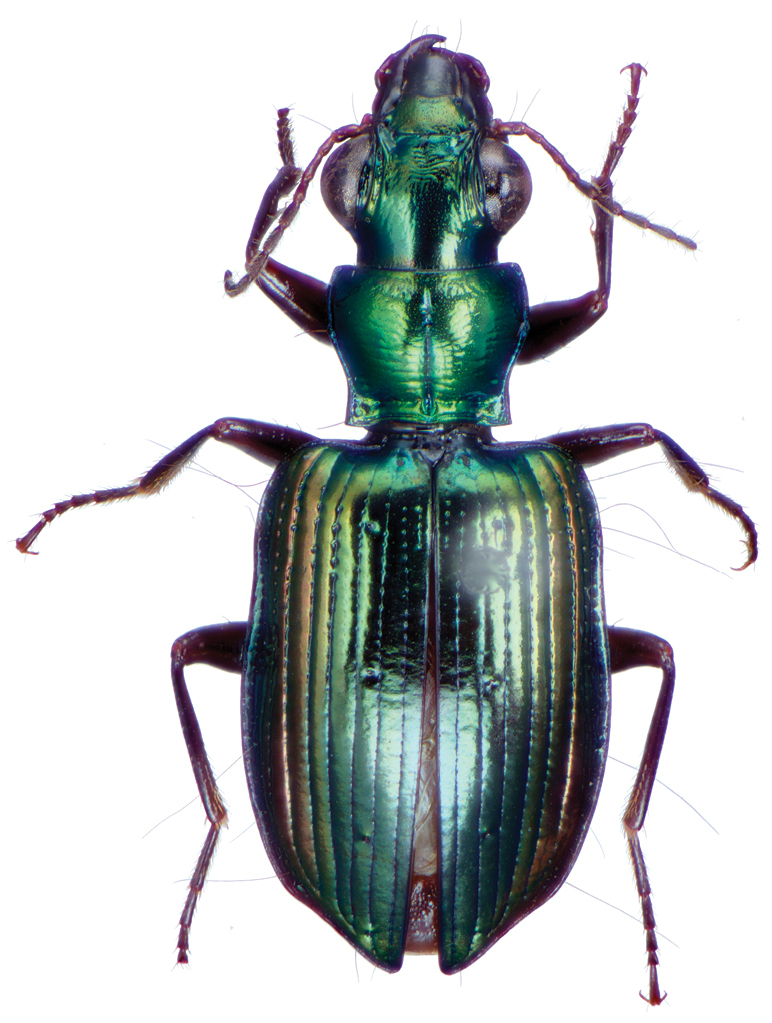
Dorsal habitus and color pattern of *Catascopus* (*s. str.*) *viridiorchis* sp. n.. (OBL 9.0 mm).

**Figure 29.**
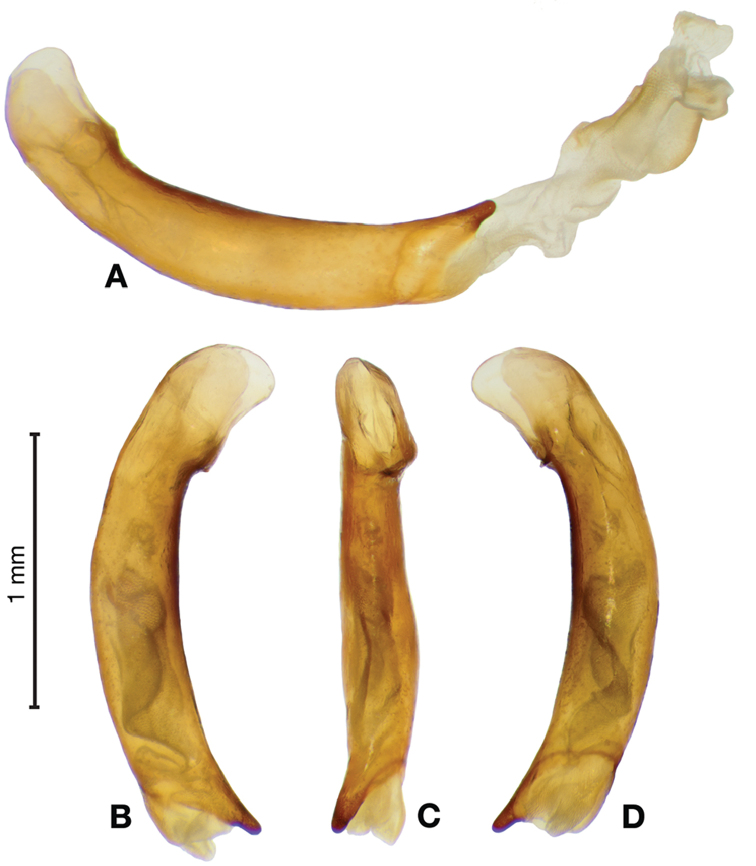
Digital images of male genitalia of *Catascopus* (*s. str.*) *viridiorchis* sp. n.. **A** right lateral aspect, endophallus everted **B** right lateral aspect **C** ventral aspect **D** left lateral aspect.

Figs 28 , 29A–D , 34C , 35

########### Specific epiphet.

From Latin *viridi*, in reference to the green dorsal coloration and *orchis*, which refers to the type locality, Orchid Island (Lanyu), Taiwan.

########### Types and other material examined.

**Holotype** (male) labeled “Holotype” [circular, ringed with red]; “Taiwan Taitung/Lanyu Yongshing Farm/VII/13–14/2000/M.M.Yang/UV light trap (upper)”; “NMNS ENT/4450-875”; “NCHU# 101729. This species is known from two specimens. For further details see EH Strickland Virtual Entomology Museum Database.

########### Type locality.

Taiwan: Orchid Island, Taitung county, Lanyu township.

########### Diagnosis.

This species is closely allied to *Catascopuselegansphilippinus* Baehr but can be distinguished by the darker green, less cupreous elytra; deeper, more pronounced fovea associated with the three pairs of fixed setae in interval 3; distinctively short and angled apex of phallus and form of endophallus (when viewed dorsally).

########### Description.

OBL 8.83 – 9.2 mm. Length (one male, one female): head 1.04, pronotum 1.44 – 1.56, elytra 5.00 – 5.33, metepisternum 1.16 – 1.20 mm; width: head 2.04 – 2.12, pronotum 1.96, elytra 3.33 – 3.50, metepisternum 0.60 – 0.64 mm.

*Body proportions*. HW/HL 1.96 – 2.04; PWM/PL 1.26 – 1.36; EL/EW 1.50 – 1.52; ML/MW 1.81 – 2.00.

*Color*. Fig. 28. Dorsum of head metallic green, clypeus black with faint cupreous sheen, labrum rufo-brunneous; antennae rufo-brunneous; palpi rufo-brunneous; disc of pronotum metallic green, lateral margins metallic blue in apical 4/5; elytral disc metallic green, margins (from interval 4) metallic green to slightly cupreous; ventral surface rufo-piceous to piceous with exception of last four sterna which are rufo-brunneous with rufo-piceous margins; legs with trochanter, femora and tibia rufo-brunneous.

########### Teneral specimen.

This species is known from only two specimens, one male and one female. The female specimen is teneral. Dorsum of the head is a metallic blue-black; pronotum bicolored with apical margin of the pronotum to the anterior transverse impression being rufo-brunneous and the remaining dorsal surface metallic blue-black; dorsal surface of the elytra rufo-brunneous with cupreous to metallic green lateral margins.

*Microsculpture*. Dorsum of head with microsculpture faintly visible at 50× magnification, isodiametric; pronotum with transverse mesh pattern faintly visible at 50× magnification; elytra with shallow, transverse sculpticells; ventral surface of head with microsculpture transverse, faintly visible at 50×; prosternum, proepipleuron, mesepisternum, and metepisternum with sculpticells forming a shallow transverse mesh.

*Macrosculpture*. Dorsum of head, clypeus and pronotum disc with fine, randomly scattered setigerous punctures; two deep furrows along contour of eye, longest ending at basal supraorbital setae; pronotum with several shallow lateral impressions from apex to baso-lateral depression; elytra with interval 7 carinate on inside margin nearest to stria 6, from just behind base to 5/6 of the elytra length, most carinate in basal half but diminishing and becoming more flattened and rounded along length; striae evenly punctate along length; intervals with fine, randomly scattered, setigerous punctures throughout; ventrally: prosternum, prosternal process, mesosternum, mesocoxa and mesosternal intercoxal process and hind coxa with scattered, shallow setigerous punctures; abdominal sterna with scattered, shallow setigerous punctures, setae slightly longer medially.

*Fixed setae.* Pronotum with two pairs of setae, one at base of lateral margin and one on lateral margin at pronotum max width; elytra with one seta at basal quarter of interval 3, one seta in interval 3 at mid-length, one seta in apical quarter of interval 3.

*Luster*. Head capsule and pronotum glossy; elytra moderately glossy; ventral thoracic sterna and abdominal sterna moderately glossy.

*Pronotum*. Anterior transverse impression shallow; posterior transverse impression deep, median longitudinal impression moderately deep; lateral margins constricted just before mid-way toward apex, becoming parallel at ¾ length; posterio-lateral margins almost right-angled.

*Male genitalia*. Fig. 29A–D. Length 1.88 mm. Phallus narrowest at base of shaft, slightly expanded on left side from median towards ostium in ventral view; apical area, a relatively short, rounded point, somewhat narrow, distinctively angled to the right when viewed from ventral aspect; endophallus long, relatively narrow, one basal endophallic lobe and two additional lobes towards apex.

*Female genitalia*. Fig. 34C. Width 1.48 – 1.64 mm. Gonocoxite 2 (gc2) with three lateral ensiform setae (les) and one dorsal ensiform seta (des) present (not visible from dorsal view). One spermatheca (sp1) relatively short, cylindrical; one spermathecal accessory gland (sg); spermathecal gland duct (sgd) slightly longer than length of spermatheca, attachment site medially on distinctively large diverticulum (div) of spermatheca.

########### Habitat, habits, and seasonal occurrence.

The known elevational range of *C.viridiorchis* sp. n. is from 35 and 160 meters. The two adults of this species were found in mixed forest of montane areas. Little is known of the habits of this species. The specimens were collected in July and August and methods of collecting include u.v. light trap and hand collecting.

########### Geographical distribution.

*Catascopusviridiorchis* is known only from Orchid Island, Taiwan. See Figure 35.

########## Subgenus Catascopoides

Taxon classificationAnimaliaColeopteraCarabidae

Habu

Catascopoides Habu, 1967: 78; Shpeley and Ball 2000: 112; Lorenz 2005: 454.
Dentiscopus Straneo, 1994: 148.

########### Type species.

*Catascopusmirabilis*Bates 1892 (by original designation).

########### Type locality.

Southeast Asia (Myanmar and the Malay Peninsula).

########### Taxonomic notes.

In a short footnote, Habu (1967) introduced the subgenus Catascopoides. His rationale for this subgenus included both unique mandibular form (Fig. 23B) and distinctive pubescence on the ventral surface of the fore and mid femora. Later, and apparently unaware of Habu’s short treatment, Straneo (1994), erected the now synonymized subgenus Dentiscopus, that was based largely on the same characters. These characteristics are indeed unique within the *Catascopus* s. l. and the three currently known species appear to be quite closely related.

########## Catascopus (Catascopoides) horni

Taxon classificationAnimaliaColeopteraCarabidae

Jedlička

**Figure 30.**
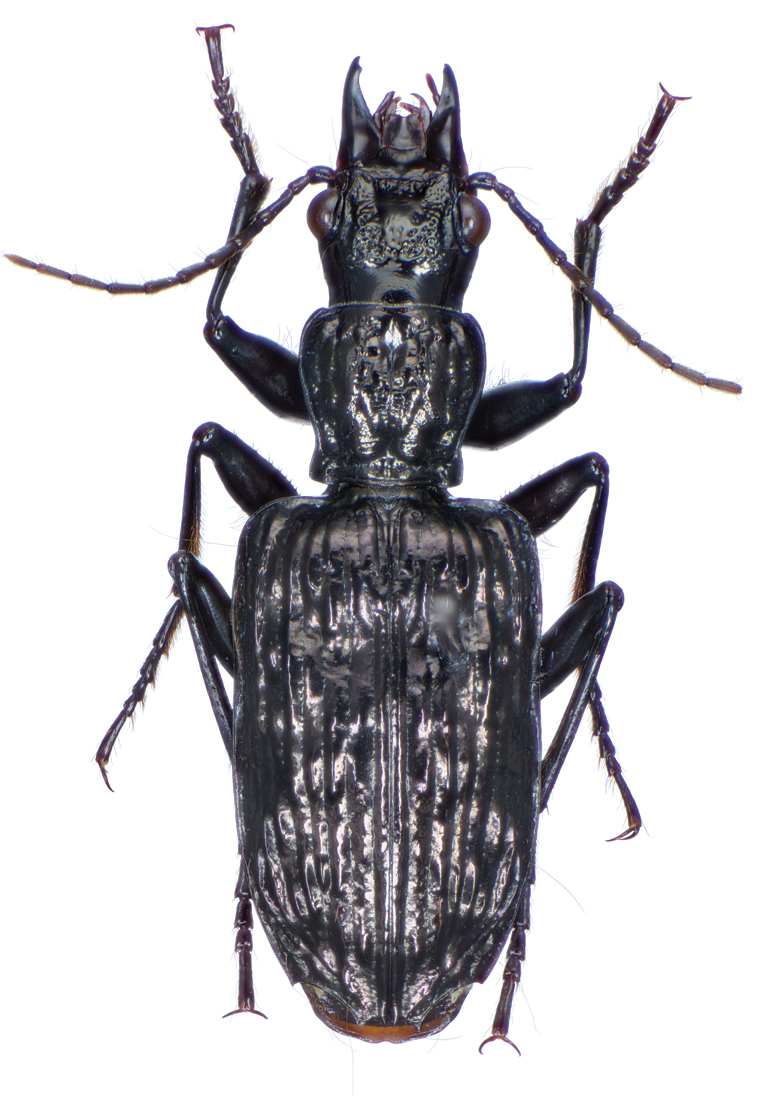
Dorsal habitus and color pattern of C. (Catascopoides) horni Jedlička (OBL 17.20 mm).

**Figure 31.**
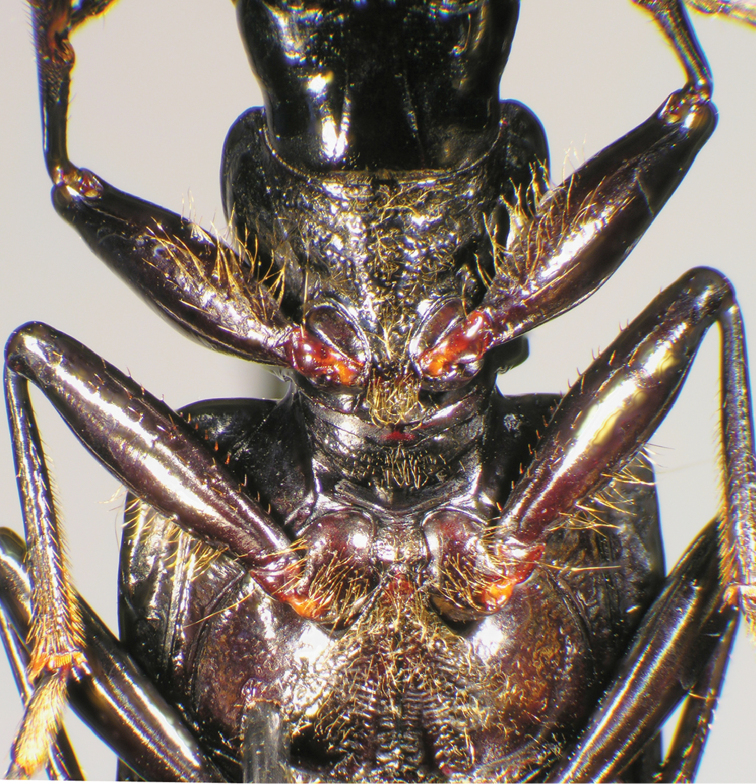
Digital image of the upper ventral surface and associated seta pattern of Catascopus (Catascopoides) horni Jedlička.

**Figure 32.**
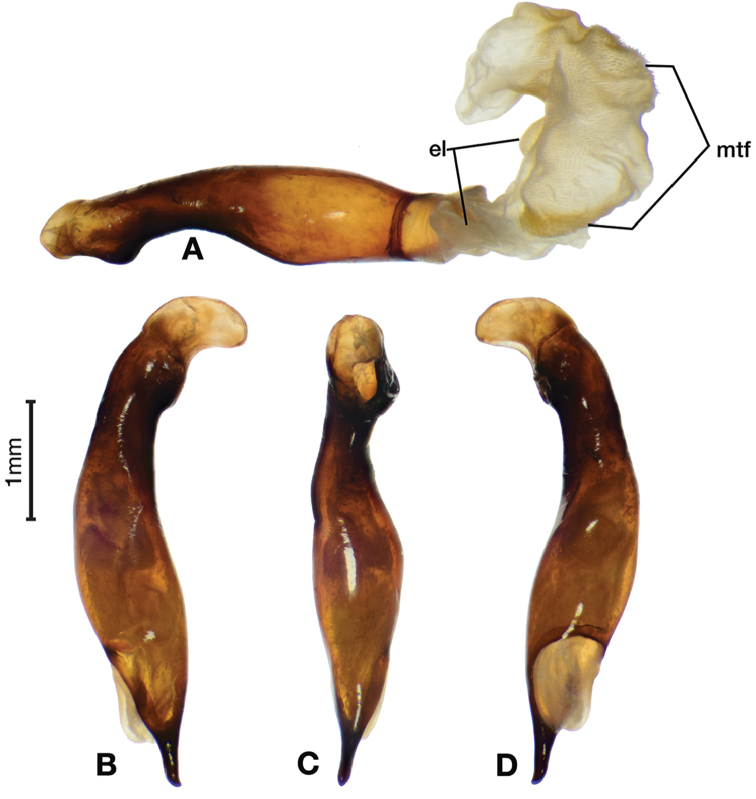
Digital images of male genitalia of C. (Catascopoides) horni Jedlička **A** left lateral aspect endophallus everted **B** right lateral aspect **C** ventral aspect **D** left lateral aspect. Legend: **el** endophallic lobe; **mtf** microtrichial field.

**Figure 33.**
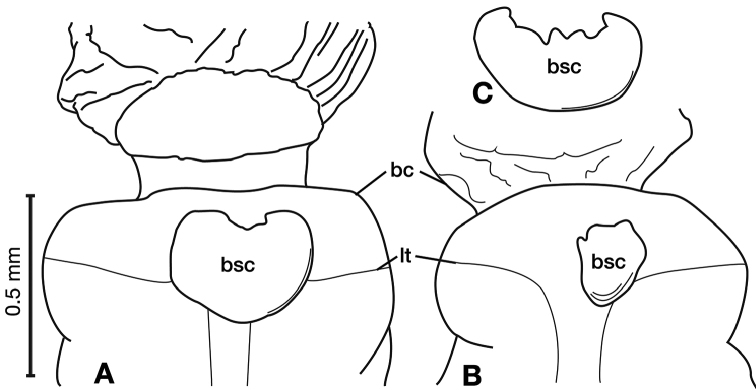
Line drawings comparing the bursal sclerite, ventral aspect of **A**Catascopus (Catascopoides) horni Jedlička **B***Catascopus* (*s. str.*) *sauteri* Dupuis **C***C.* (*Catascopoides*) *mirabilis* Bates (not present in Taiwan) Legend: **bc** bursa copulatrix; **bsc** bursal sclerite (type 1); **lt** lateral tergites.

**Figure 34.**
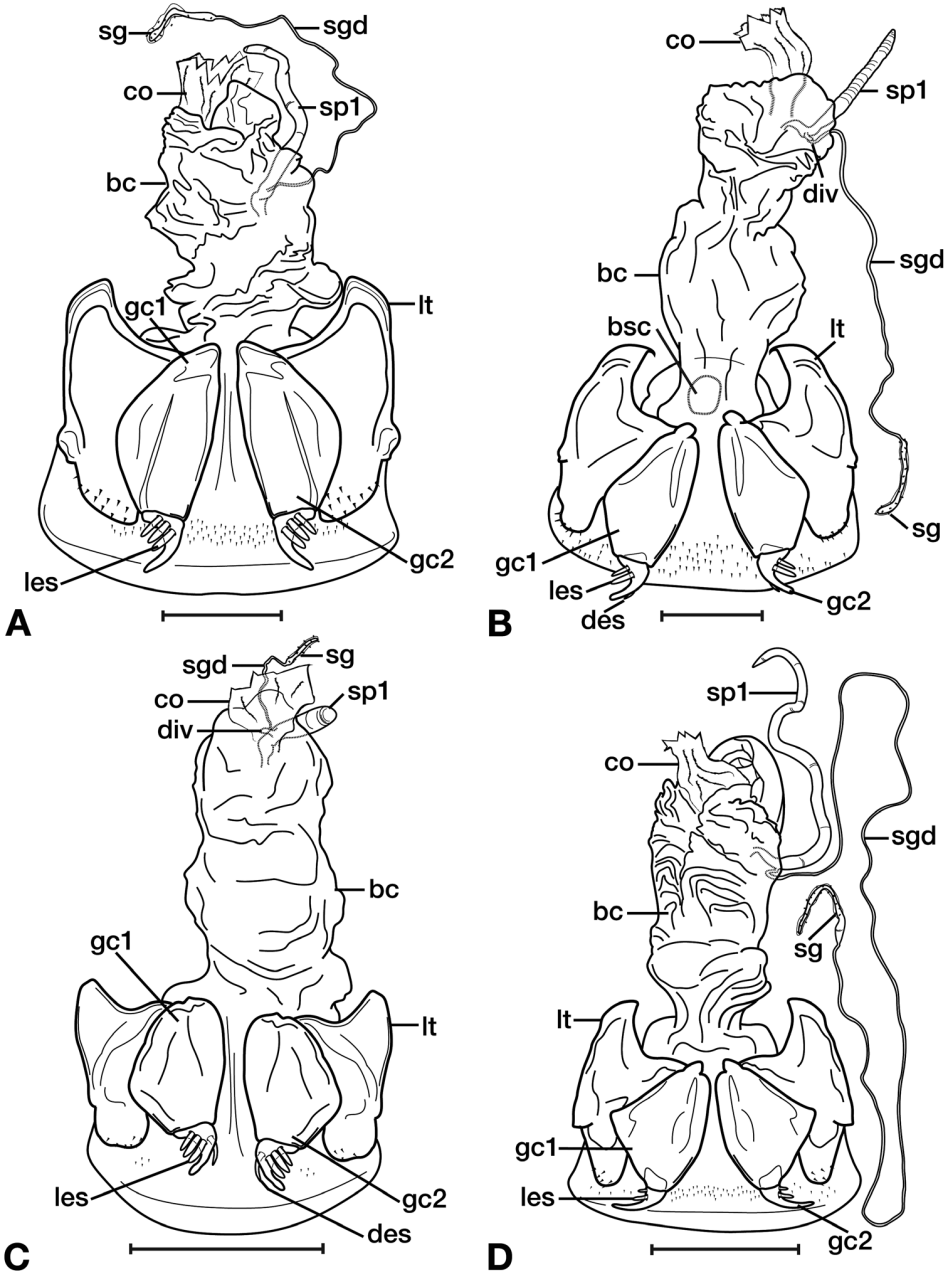
Line drawings of the female reproductive tract of species of the genus *Catascopus* Kirby, known from Taiwan, ventral aspect. **A***C.* (*s. str.*) *ignicinctus* Bates **B***C.* (*s. str.*) *sauteri* Dupuis **C***C.* (*s. str.*) *viridiorchis* sp. n. **D**C. (Catascopoides) horni Jedlička. Legend: **bc** bursa copulatrix; **bsc** bursal sclerite; **co** common oviduct; **des** dorsal ensiform setae; **div** diverticulum; **gc1** gonocoxite 1; **gc2** gonocoxite 2; **les** lateral ensiform setae; **lt** lateral tergite; **sg** spermathecal gland; **sgd** spermathecal gland duct; **sp1** spermatheca 1. Scale bars: 1 mm.

**Figure 35.**
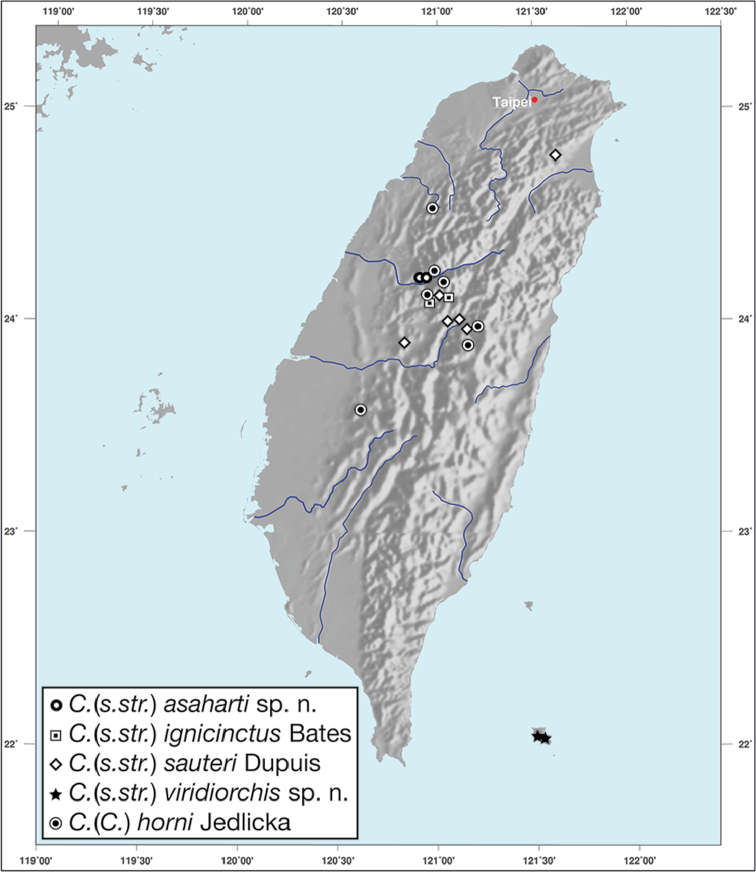
Map showing known localities for species of the genus CatascopusKirby and subgenus Catascopoides Habu, in Taiwan.

Figs 23B , 30 , 31 , 32A–D , 33A , 34D , 35

Catascopus (Catascopoides) horni Jedlička, 1932: 82; Jedlička 1963: 383; Straneo 1994: 143; Lorenz 2005: 454.
Catascopus (Dentiscopus) horni Jedlička: Straneo, 1994: 148.

########### Types and other material examined.

**Holotype** (female) labeled: “Banshoryo – Disfr./Sokutsu (Formosa)/H. Sauter VI. 1912”; “Holotypus”[rectangular, red paper]; “TYPE”[rectangular, red paper, black border]; “Catascopus Horni sp. n./mihi/DET.ING.JEDLICKA”; “DEI Coleoptera/# 200416”. 23 specimens of *C.horni*: seven males and 16 females. For further details see EH Strickland Virtual Entomology Museum Database.

########### Type locality.

“Formosa, Sokutsu”. Formosa is the old name for Taiwan and Sokutsu refers to Hsiaolin, Kaohsiung county.

########### Taxonomic notes.

Both *C.* (*C.*) *mirabilis* and *C.* (*C.*) *horni* were recorded from Taiwan (Taiwan being the type locality of *C.* (*C.*) *horni* Jedlička). From the images and illustrations, it appeared that two species were superficially very similar so it is possible that they might be conspecific. All of the major collections in Taiwan were examined, and most known Taiwanese material was borrowed. Fresh material was collected from the wild. The holotype of *C.* (*C.*) *horni* and three paratypes of *C.* (*C.*) *mirabilis* are deposited SDEI, Germany where, upon examination, it was clear that the species could be distinguished from one another but that it was more of a total collection of subtleties than any one obvious external character that set them apart. *Catascopus* (*C.*) *mirabilis* differs from *C.* (*C.*) *horni* in that it has slightly larger eyes, a slightly more cupreous sheen, apical and basal angles of pronotum that are that are slightly more lobed and explanate, elytral microsculpture that is more granulate and raised, giving a duller appearance, and broken striae (primarily 3, 5, 7) that are somewhat more raised. All of these characters are slightly variable and difficult to define so it became clear that without a lot of material to compare, confusing these two species would be easy. After going through the material however, a few good characters were consistent and allowed for discrimination of species. *C.* (*C.*) *mirabilis* has females with the ventral surface of the fore-femur with a dense field of yellow setae (more than 20), while *C.* (*C.*) *horni* only has a field of ~twelve setae. Females of *C.* (*C.*) *mirabilis* also have a genitalic character, the bursa copulatrix sclerite (bsc, Fig. 33C), that makes them easily distinguishable from *C.* (*C.*) *horni*. After dissecting the available material including the specimens that were examined and thought to be *C.* (*C.*) *mirabilis* by both Jedlička (1963) and Habu (1967), it seems apparent that *C.* (*C.*) *horni* is restricted to Taiwan while *C.* (*C.*) *mirabilis* is known from Vietnam, Laos and China. See also “Geographical distribution”.

########### Diagnosis.

Specimens of this species are easily distinguished from other pericalines by the distinctly asymmetrical mandibles and metallic black dorsal coloration.

########### Redescription.

OBL 15.5 – 19 mm. Length (n = seven males, ten females): head 1.24 – 1.56, pronotum, 2.76 – 3.44, elytra 8.25 – 10.50, metepisternum 2.08 – 2.68 mm; width: head 3.00 – 3.72, pronotum 2.92 – 3.76, elytra 5.00 – 5.83, metepisternum 1.08 – 1.20 mm.

*Body proportions*. HW/HL 2.35 – 2.49; PWM/PL 1.07 – 1.16; EL/EW 1.65 – 1.83; ML/MW 1.93 – 2.31.

*Color*. Fig. 30. Dorsum of head brunneo-piceous to piceous, with faintly metallic violaceous sheen; antennae with segments 1–4 rufo-piceous to piceous, segments 5–11 brunneous to rufo-piceous; palpi rufo-brunneous to rufo-piceous; clypeus and mentum rufo-piceous; pronotum piceous, with faintly metallic violaceous sheen; elytra piceous, with faintly metallic violaceous sheen; elytral epipleura piceous; thoracic sclerites rufo-piceous to piceous; abdominal sterna rufo-piceous medially, rufo-piceous to piceous at lateral margins; legs with trochanter and femora rufo-piceous, tibia rufo-piceous to piceous.

*Microsculpture*. Dorsum of head with microsculpture not visible at 50× magnification; pronotum with transverse mesh pattern faintly visible at 50× magnification; elytra with shallow, transverse sculpticells on majority of disc, lower depressions of striae are nearly isodiametric, easily visible at 50× magnification; ventral surface of head, prosternum, proepipleuron, mesepisternum, and metepisternum with sculpticells forming a shallow transverse mesh.

*Macrosculpture*. Dorsum of head with scattered punctures laterally in front of eye to mid-way between basal supraorbital setae and pronotum apex, punctures near base of head are deep and confluent, more separated and shallow towards apex; pronotum rugulose; elytra with 3–4 diagonal impressions on disc, impressions evenly spaced with first impression near lateroapical angle of elytral base to suture, joining suture ~1/3 from base, first and third impressions deeper than others; intervals carinate, intervals 3,5,7, more strongly carinate in basal half than others, intervals convex to flat where interrupted by diagonal impressions; intervals 7 and 8 distinctly carinate and rounded at apical 1/4, disrupting normal contour of elytra; striae punctate along length; ventrally: prosternum, prosternal process, mesosternum, mesocoxa, and mesosternal intercoxal process, metasternum, hind coxa and base of abdominal sternite 3 with scattered and deep punctation, often confluent and rugulose in appearance; metasternum with several lateral striations on either side of suture (comb-like in appearance); abdominal sterna with scattered, shallow punctures; fore femur of males with more than twenty deep punctures in basal half of ventral surface, females with less (eight to fifteen), not all bearing setae.

*Pilosity*. Fig. 31. Dorsum of head, pronotum and disc of elytra with scattered micro-punctures; ventrally: prosternum, prosternal process, mesosternum, mesocoxa, and mesosternal intercoxal process and metasternum with moderate to long, blonde, setae associated with deep punctures; fore and mid femora of males with long, blonde setae associated with each puncture; fore and mid femora of females with several (fewer than males) blonde setae of various lengths in most punctures, some punctures without setae.

*Fixed setae.* Two pairs of supraorbital setae; clypeus with two lateral setae; labrum with six setae along apical margin; one pair of suborbital setae; pronotum with one seta at base of lateral margin; elytra with one seta at basal quarter of interval 3, one seta in interval 3 at mid-length, one seta in apical quarter of interval 3; 16–17 lateral (umbilical) setae in interval 9; two setae on each of abdominal sterna III to VI, two setae along apical margin of sternum VII in males, four setae along apical margin of sternum VII.

*Luster*. Head capsule, pronotum and elytra moderately glossy to glossy; ventral thoracic sterna and abdominal sterna moderately glossy.

*Head*. Fig. 23B. Mandible long and almost straight, obtuse tooth near apex of inside margin of each mandible, tooth of left mandible larger and situated lower than right, left mandible with additional tooth on inside cutting surface above mid-length of mandible; labrum unevenly bilobed, right lobe always longer that left; mentum with single broad tooth; eyes convex; palpi cylindrical, elongate.

*Pronotum*. Anterior transverse impression moderately deep; posterior transverse impression and median longitudinal impression deep; apical margin narrowly curved forming short, acute latero-apical lobes; lateral margins sinuate toward base; posterio-lateral margins almost right-angled.

*Elytra*. Humeri broadly rounded; elytral margin shallowly impressed in basal 1/3; elytral apices each with one small lateral spine and two apical spines, outside apical spine always longer than inside apical spine.

*Hind wings*. Macropterous.

*Legs*. Tarsal claws smooth. Males with adhesive vestiture ventrally, two rows of squamo-setae on tarsomeres 1–3 of fore-leg.

*Abdominal sterna.* Abdominal sternum VII bilobed, with shallow notch apically.

*Male genitalia*. Fig. 32A–D. Length 3.52 – 4.00 mm. Ostium left pleuropic. Phallus cylindrical, narrowest at base of shaft, expanded on left side from base towards median in ventral view, constricted again before apex; apical area distinctively long and narrow, apical curve visible in left and right view. Endophallus relatively long and distinctly curled left when viewed from left lateral aspect; sclerite near endophallus base, left side of ostium when viewed from left lateral aspect; one basal endophallic lobe near center of ostium opening; one meso-endophallic lobe on inside of the curled endophallus when viewed from left lateral aspect; two microtrichial fields present (see fig. 32A)

*Female genitalia*. Figs 33A, 34D. Width 1.84 – 2.12 mm. Gonocoxite 2 (gc2) wide at base, narrowing sharply toward apex, highly curved; two lateral ensiform setae (les) and one dorsal ensiform seta (des) present (not visible from dorsal view). Sensory furrow, furrow pegs and associated nematiform setae not observed; Bursa copulatrix highly textured with many infoldings; large, distinctively shaped sclerite (bsc) (type 1) internally at base, between apex of lateral tergites (not visible from dorsal view), differing from the bursal sclerite of *Horniulusandrewesi* Jedlička (Fig. 94B) in that the sclerite appears to be inside the tissue of the dorsal surface of the bursa and not open to the interior cavity; one spermatheca (sp1) long and narrow; One spermathecal accessory gland (sg) long and narrow; spermathecal gland duct (sgd) very long and narrow, attachment site at base of spermatheca.

########### Habitat, habits, and seasonal occurrence.

The known elevational range of *C.horni* is from 300 to 2000 meters. Only two specimens have been collected below 1000 meters altitude, with most specimens being collected from 1500 to 2000 meters. Adults of this species are found in mixed primary and secondary forest of montane areas. Adults are crepuscular or nocturnal with most activity observed on trunks of fallen or dying trees at night. Specimens have been collected from March to December but are most commonly collected from March to June. Methods of collecting include u.v. light, sugar baits painted on tree trunks (have not been observed at actual bait, only near), hand collecting and malaise trap. The only confirmed tree species from which *C.* (*C.*) *horni* has been collected is *Pinusmorrisonicola* Hayata. Adults are very fast runners and when they are lit at night, they will quickly run to the dark side of the tree.

########### Geographical distribution.

*Catascopushorni* is known only from Taiwan. See Figure 35.

########## Genus Coptodera

Taxon classificationAnimaliaColeopteraCarabidae

Dejean

Coptodera Dejean, 1825: 273.

########### Type species.

*Coptoderafestiva* Dejean.

########### Type locality.

Cuba.

########## Subgenus Coptoderina

Taxon classificationAnimaliaColeopteraCarabidae

Jeannel

Coptoderina Jeannel, 1949: 1924; Nakane and Ohkura 1956: 45; Habu 1957: 114; Shibata 1964: 40; Ball 1975: 177; Lorenz 2005: 457; Baehr 2005: 33; 2007: 175; 2014: 249; Park et al. 2011: 103; Gamboa and Ortuno 2015: 593.

########### Type species.

*Catascopusequestris* Boheman, 1848.

########### Type locality.

South Africa.

########### Recognition of Taiwanese species of *Coptoderina*

########### .

*Color.* Various.

*Fixed setae.* Two pairs of supraorbital setae; clypeus with two lateral setae; labrum with six setae along apical margin; one pair of suborbital setae; pronotum with two pairs of setae set in raised punctures, one at base of lateral margin and one on lateral margin at pronotum max width; 17 lateral (umbilical) setae in interval 9; ventrally, two setae on each of abdominal sterna III to VI, four setae along apical margin of sternum VII.

*Head*. Mentum with no tooth; eyes large, convex; palpi cylindrical, elongate, and setose.

*Elytra*. Humeri broadly rounded; lateral margin explanate, slightly more so in basal 1/3.

*Hind wings*. Macropterous.

*Legs*. Tarsal claws denticulate, three to four denticles per claw, males with adhesive vestiture ventrally, two rows of squamo-setae on tarsomeres 1–3 of fore-leg.

*Female genitalia*. Gonocoxite 2 (gc2) long, narrow, slightly curved; three lateral ensiform setae (les) and one dorsal ensiform seta (des) present. Sensory furrow, furrow pegs and associated nematiform setae not observed.

########### Taxonomic notes.

All species from the Oriental Region belong to the subgenus Coptoderina Jeannel, 1949. They are distinguished by the median lobe of males with ostium catopic.

######### Key to the Taiwanese species of the subgenus Coptoderina Jeannel

**Table:** 

1	Two fixed setae in basal 1/3 of stria 3	**2**
–	One fixed seta in basal 1/4 of stria 3	**3**
2	Head rugulose between eyes; elytra with small spine at apex, disc with six maculae	***C.eluta* Andrewes**
–	Head smooth or only faintly rugulose between eyes; elytra with no spine at apex, disc with four maculae	***C.chaudoiri* Andrewes**
3	Stria 2 of elytra with or without setae but none in apical third	**4**
–	Elytra with fixed seta in apical 1/3 of stria 2	**5**
4	No fixed setae in stria 2; head and pronotal disc black	***C.proksi* Jedlička**
–	One fixed seta at mid-length of stria 2; head and pronotal disc metallic green	***C.taiwana* (Nakane)**
5	Pronotum and elytra with lateral margins obviously lighter in coloration than disc	**6**
–	Pronotum and elytra with margins not or only slightly lighter in color than disc	***C.japonica* Bates**
6	Apex of elytra clearly sinuate; head brunneous to dark brunneous	**7**
–	Apex of elytra almost straight; head piceous to black	***C.occulta* sp. n**
7	Apical macula of elytra extended from suture to stria 8	***C.maculata* (Dupuis)**
–	Apical macula of elytra extended from stria 1 to stria 8, never touching suture	***C.marginata* (Dupuis)**

########## Coptodera (Coptoderina) chaudoiri

Taxon classificationAnimaliaColeopteraCarabidae

Andrewes

**Figure 36.**
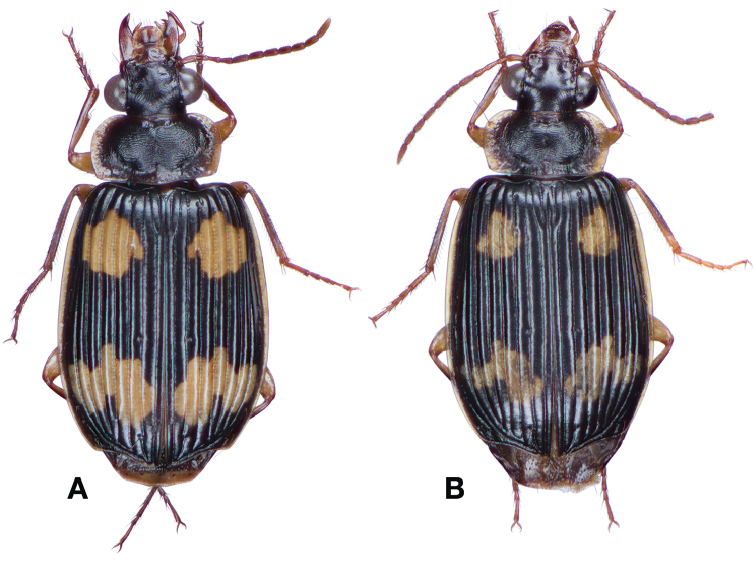
Dorsal habitus and intrapopulation variation of color pattern of C. (Coptoderina) chaudoiri Andrewes. **A** large elytral macula (OBL 8.23 mm) **B** small elytral macula (OBL 7.20 mm).

**Figure 37.**
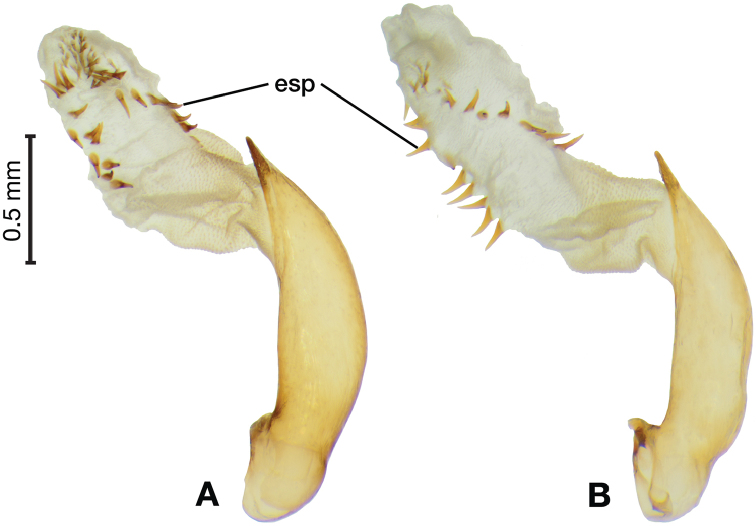
Digital images showing intrapopulation variation of form and number of endophallic spines of C. (Coptoderina) chaudoiri Andrewes, right lateral aspect, endophallus everted. **A** heavily sclerotized spines with many additional spines towards apex of endophallus **B** lightly sclerotized spines with fewer spines near apex of endophallus. Legend: **esp** endophallic spines.

**Figure 38.**
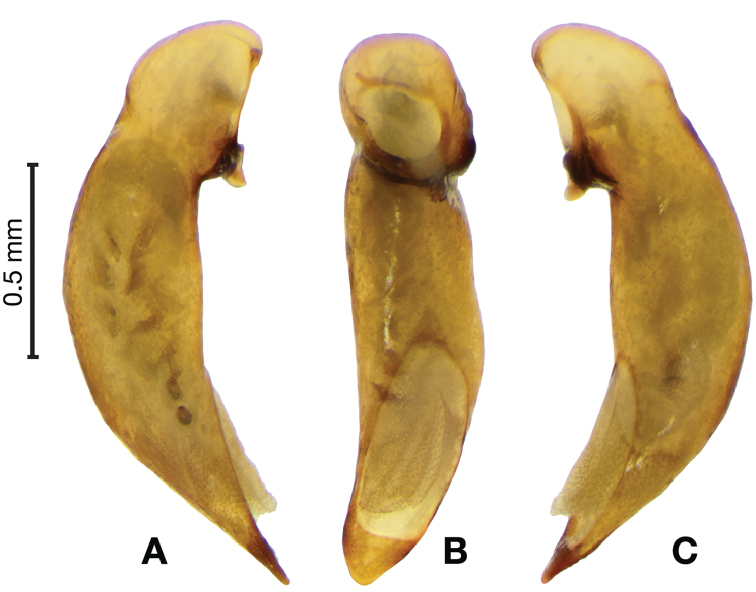
Digital images of male genitalia of C. (Coptoderina) chaudoiri Andrewes. **A** right lateral aspect **B** ventral aspect **C** left lateral aspect.

Figs 36A, B , 37A, B , 38A–C , 57A , 59

Coptodera chaudoiri Andrewes, 1919: 179; Lorenz 2005: 457.
Coptoderina transversa anguilipennis Nakane & Okhura, 1956: 47; Jedlička 1963: 349.
Coptoderina chaudoiri anguilipennis (Nakane & Okhura): Lorenz 2005: 457. syn. n.
Coptodera nobilis Jedlička, 1963: 349; Lorenz 2005: 458. syn. n.

########### Types and other material examined.

28 specimens of *C.chaudoiri*: 17 males and eleven females. Types of both *C.transversaanguilipennis* and *C.nobilis* were examined and dissected. Specimens of these proposed species did not differ in any way from *C.chaudoiri*, other than the very slight and normal variability typical of the subgenus Coptoderina. For further details see EH Strickland Virtual Entomology Museum Database.

########### Type locality.

Hong Kong.

########### Diagnosis.

Specimens of this species are distinguished from other species of Taiwanese *Coptodera* by a combination of having two setae in the basal 1/3 of stria 3, a head that is smooth or only slightly rugulose between eyes and males with adhesive vestiture on tarsomeres 1–3 of mid-leg (all others with only 1–2).

########### Description.

OBL 6.33 – 8.66 mm. Length (n = ten males, ten females): head 0.76 – 0.96, pronotum 0.96 – 1.36, elytra 3.50 – 5.33, metepisternum 0.92 – 1.28 mm; width: head 1.44 – 1.88, pronotum 1.84 – 2.56, elytra 2.83 – 4.00, metepisternum 0.52 – 0.80 mm.

*Body proportions*. HW/HL 1.89 – 2.26; PWM/PL 1.79 – 2.00; EL/EW 1.32 – 1.38; ML/MW 1.32 – 1.76.

*Color*. Fig. 36A, B. Dorsum of head brunneo-piceous to piceous, clypeus and labrum brunneo-testaceous to rufo-brunneous, antennae and palpi rufo-brunneous; disc of pronotum brunneo-piceous to piceous, lateral margins testaceous to brunneous, always lighter then disc, some transparent at margins; disc of elytra black, with four testaceous macula, two anterior and two posterior, variable, anterior macula from interval 3 to 7 (some 3 to 6), closest to base of elytra in interval 5, closest to apex of elytra in interval 4 and 5, posterior macula from stria 1 to interval 8, closest to base of elytra in interval 5, closest to apex of elytra in interval 3 and 4; margins of elytra testaceous to rufo-brunneous; ventral surface with thoracic and abdominal tergites rufo-brunneous to rufo-piceous, margins of abdominal tergites and metepisternum darker; legs contrastingly lighter, with trochanter and femora and tarsi testaceous to brunneo-testaceous, tibia rufo-brunneous to darker on dorsal surface.

*Microsculpture*. Dorsum of head with microsculpture granulate, isodiametric, easily visible at 50× magnification; pronotum microsculpture granulate, isodiametric; elytral disc with sculpticells somewhat granulate, nearly isodiametric, cells up to 1.5× longer than wide; ventral surface of head, smooth with microsculpture not visible at 50×; prosternum, proepipleuron, mesepisternum and metepisternum with sculpticells forming a shallow, somewhat transverse to transverse mesh.

*Macrosculpture*. Dorsum of head faintly rugulose to smooth between eyes, clypeus faintly rugulose to smooth, both head and clypeus with relatively dense, fine and scattered setigerous punctures, punctures not visible and setae hardly visible at 50×; pronotum with disc rugulose, entire surface with fine and scattered setigerous punctures, punctures not visible but setae visible in side view at 50×; elytra with intervals rounded, fine scattered setigerous punctures on entire dorsal surface, hardly visible in lateral view at 50×, striae relatively wide (three to four cells), concave and blending into intervals smoothly, punctate, with single row of fine scattered setigerous punctures, hardly visible in lateral view at 50×; ventrally, thoracic and abdominal sclerites with scattered setigerous punctures throughout.

*Fixed setae.* Elytra with two setae in apical half of stria 2, two setae in basal 1/3 of stria 3.

*Luster*. Dorsal surface moderately dull.

*Head*. Mandibles somewhat curved at apex, somewhat long and narrow in form; labrum bilobed, widely emarginate, broadly rounded and relatively short.

*Pronotum*. Transverse impression deep; posterior transverse impression moderately shallow, median longitudinal impression shallow; lateral margins explanate, apico-lateral margins rounded, broadly lobed, posterio-lateral margins broadly rounded, obtuse.

*Elytra*. Apex almost truncate.

*Legs*. Two rows of small squamo-setae on tarsomeres 1–3 of mid-leg, males with one notch apically on ventral side of mid-tibia.

*Male genitalia*. Figs 37A, B, 38A–C. Length 1.28 – 1.58 mm. Ostium catopic, positioned slightly more to left side of dorsal surface. Phallus cylindrical, distinctively curved to the left from mid-length to apex in ventral view, apical area short, apex broadly rounded; endophallus wide and straight, two rows of spines (esp) from mid-length towards apex, joining before apex, spine rows variable, some specimens with more sclerotized spines and additional spines where rows become confluent.

*Female genitalia*. Fig. 57A. Width 1.26 mm. One spermatheca (sp1), cylindrical and long, ribbed laterally along length; one spermathecal accessory gland (sg), narrow and somewhat cylindrical; spermathecal gland duct (sgd) with swelling towards apex, attachment site apically on small diverticulum (div) of spermatheca; bursa copulatrix (bc) with distinctive sac at apical end (bs), large and bulbous.

########### Habitat, habits, and seasonal occurrence.

The known elevational range of *C.chaudoiri* is from 250 to 1125 meters with the majority of specimens collected from 480 to 750 meters. Adults of this species are found in mixed forest of montane areas and are crepuscular and can be found on trunks of live trees, and on bracket fungus. Specimens have been collected from May to September. Methods of collecting include u.v. light sheet, sweep netting near lights at night and hand collecting.

########### Geographical distribution.

*Coptoderachaudoiri* is known from Japan and Taiwan. For Taiwan localities see Figure 59.

########## Coptodera (Coptoderina) eluta

Taxon classificationAnimaliaColeopteraCarabidae

Andrewes

**Figure 39.**
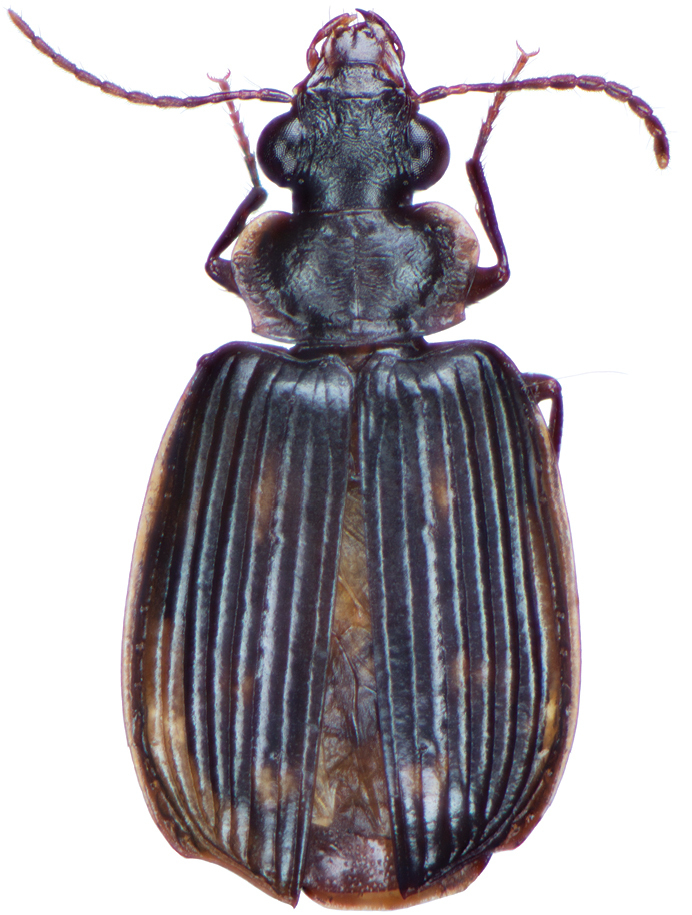
Dorsal habitus and color pattern of C. (Coptoderina) eluta Andrewes. (OBL 6.44 mm).

**Figure 40.**
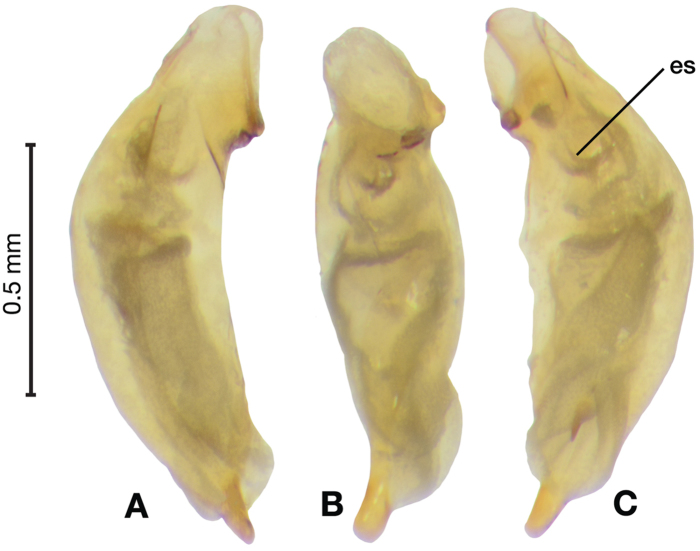
Digital images of male genitalia of C. (Coptoderina) eluta Andrewes. **A** right lateral aspect **B** ventral aspect **C** left lateral aspect.

Figs 39 , 40A–C , 57b , 59

Coptodera eluta Andrewes, 1923: 30 (without type designation); Andrewes 1930: 336; Csiki 1932: 1370; Louwerens 1956: 225; Jedlička 1963: 341; Habu 1967: 93; Lorenz 2005: 457; Park et al. 2011: 103.
Coptodera interrupta Schmidt-Goebel, 1846: 53; Chaudoir 1869: 194; Bates 1886: 203; Bates 1892: 411.
Coptodera elegantula Schmidt- Goebel, 1846: 54; Bates 1889: 111; Bates 1892: 411.
Coptoderina eluta reductemaculata Nakane & Ohkura, 1956: 46; Habu 1959: 259; 1961: 45; Jedlička 1963: 350; Shibata 1964: 40.
Coptodera madara Habu, 1957: 114; Jedlička 1963: 350.

########### Types and other material examined.

**Holotype** (male) labeled: “Tenasserim/Vall Houmdanau/Fea. Maggio 1887” [black border]; H. E. Andrewes Coll./B.M.1945-97.”; “TYPE” [rectangular, red paper]; “Coptodera /eluta/Type Andr./H. E. Andrewes det.”. **Cotype** (female) and three other specimens of *C.eluta* (one male and two females); for further details see EH Strickland Virtual Entomology Museum Database.

########### Type locality.

(Myanmar) Burma.

########### Taxonomic note.

Jedlička (1963) suggested that there was a unique form of C.eluta from Taiwan that he called ab. *unicolor*. His justification was that specimens he observed had elytra with no maculae visible basally and only small maculae apically. This pattern of coloration falls within the normal variability of coloration in *C.eluta* and because of this his delineation has no taxonomic value.

########### Diagnosis.

This species is easily distinguished from other Taiwanese *Coptodera* by a combination of small size, spined elytral apex, and elytra with two setae in basal 1/3 of stria 3.

########### Description.

OBL 6.08 – 6.66 mm. Length (n = two males, three females): head 0.72 – 0.76, pronotum 1.00 – 1.04, elytra 3.92 – 4.00, metepisternum 0.88 – 0.92 mm; width: head 1.44 – 1.52, pronotum 1.80 – 1.92, elytra 2.67 – 3.17, metepisternum 0.52 – 0.60 mm.

*Body proportions*. HW/HL 1.89 – 2.11; PWM/PL 1.80 – 1.88; EL/EW 1.26 – 1.47; ML/MW 1.53 – 1.77.

*Color*. Fig. 39. Various. Dorsum of head and clypeus rufo-brunneous to piceous, labrum, antennae, and palpi rufo-brunneous; disc of pronotum rufo-brunneous to brunneous, lateral margins somewhat translucent, rufo-brunneous to testaceous; elytral disc brunneous to brunneo-piceous, with six testaceous to rufo-testaceous maculae, two anterior, two meso-posterior and two posterior, anterior macula variable, just above the second fixed seta of stria 3 and centered in interval 4, this can range from a hardly visible light spot, to a stretched ovoid shape in interval 4 only, to a larger ovoid shape extending into interval 3 and 5, meso-posterior macula variable, +/- half-crescent shape with crescent open towards apex of elytra, extended from stria 3–7, widest in interval 4 and 5, posterior macula variable, a small patch extended across intervals 2 and 3 but uneven, portion of macula on interval 2, shorter then portion on interval 3; margins of elytra somewhat translucent, rufo-brunneous to testaceous; ventral surface brunneous to brunneo-piceous with exception of proepipleuron and elytral epipleuron which are brunneous to testaceous; legs with trochanter and femora brunneous to rufo-brunneous, tibia rufo-brunneous to rufo-piceous.

*Microsculpture*. Dorsum of head with microsculpture somewhat granulate, easily visible at 50× magnification, slightly transverse to almost isodiametric; pronotum somewhat granulate, transverse mesh pattern easily visible at 50×; elytra with transverse sculpticells, mesh almost touching center of striae, throughout length; ventral surface of head with microsculpture transverse, faintly visible at 50×; prosternum, proepipleuron, mesepisternum and metepisternum with sculpticells forming a shallow transverse mesh.

*Macrosculpture and pilosity*. Dorsum of head finely rugulose medially, with finely scattered setigerous punctures, visible only in side view at 50×, 2–3 furrows along contour of eye; pronotum with finely scattered setigerous punctures, visible in side view at 50×, outside margin of disc to lateral margin shallowly rugulose; elytra with intervals broadly rounded, single row of fine setigerous punctures in the center of each interval, striae narrow, single row of setigerous punctures in each stria, hardly visible at 50×.

*Fixed setae.* Elytra with two setae in basal 1/3 of stria 3, two setae in apical 1/3 of stria 2.

*Luster*. Head capsule and pronotum moderately dull; elytra moderately glossy; ventral thoracic sterna and abdominal sterna moderately glossy.

*Head*. Mandibles curved at apex, relatively short and narrow in form, mostly covered by labrum; labrum bilobed, emargination triangular in form, somewhat elongate and rounded towards apex.

*Pronotum*. Disc with one round shallow depression on either side; anterior transverse impression shallow; posterior transverse impression deep, median longitudinal impression moderately deep; lateral margins explanate, apico-lateral margins broadly rounded and curled up at margin, posterio-lateral margins slightly sinuate, obtuse.

*Elytra*. Apex with small spine.

*Legs*. A few small squamo-setae on tarsomere 2 of mid-leg, males with one notch apically on ventral side of mid-tibia.

*Male genitalia*. Fig. 40A–C. Length 1.16 mm. Ostium left pleuropic. Phallus cylindrical, distinctively wide medially, apical area with short, bluntly rounded apex, curved to the right when viewed from ventral aspect; endophallus only viewed through phallus, one basal endophallic spine apparent (es), other sclerotized areas may also be present but have not been confirmed. Habu (1961) suggested that the endophallus contained “two small copulatory pieces”.

*Female genitalia*. Fig. 57B. Width 1.00 – 1.04 mm. One spermatheca (sp1), cylindrical, ribbed laterally along length; one spermathecal accessory gland (sg), large and somewhat cylindrical, from apex of duct appears as two chambers, a small chamber followed by a constriction approximately the width of the gland duct, to a larger apical chamber; spermathecal gland duct (sgd) more than twice as long as length of spermatheca, attachment site medially on large diverticulum (div) of spermatheca; bursa copulatrix (bc) with distinctive sac at apical end, large and somewhat cylindrical, coming to a point at the apex (bs).

########### Habitat, habits, and seasonal occurrence.

The known elevational range of *C.eluta* is from 100 to 220 meters. Adults of this species are found in mixed forest of montane areas. Little is known of the habits of this species. Specimens have been collected from June and September in Taiwan and methods of collecting include “at light” and hand collecting.

########### Geographical distribution.

*Coptoderaeluta* has a wide, Asian distribution. It is known from Japan, Korea, the Philippines, Indo-China, Malaysia, Thailand, Myanmar, Ceylon, India, and Taiwan. For Taiwan collecting localities see Figure 59.

########## Coptodera (Coptoderina) japonica

Taxon classificationAnimaliaColeopteraCarabidae

Bates

**Figure 41.**
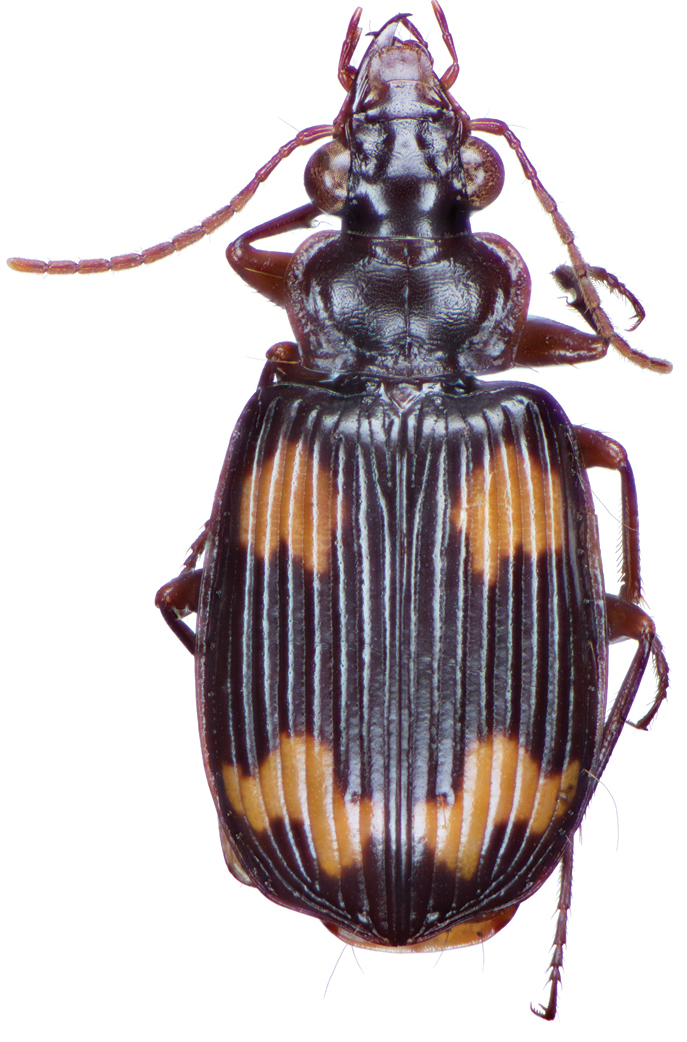
Dorsal habitus and color pattern of C. (Coptoderina) japonica Bates. (OBL 10.24 mm).

**Figure 42.**
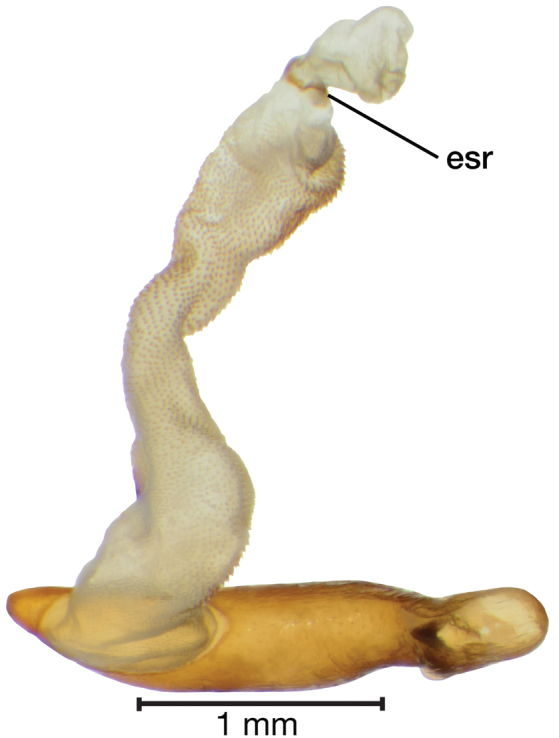
Digital image of male genitalia of C. (Coptoderina) japonica Bates, ventral aspect, endophallus everted. Legend: **esr** endophallic sclerotized ring.

**Figure 43.**
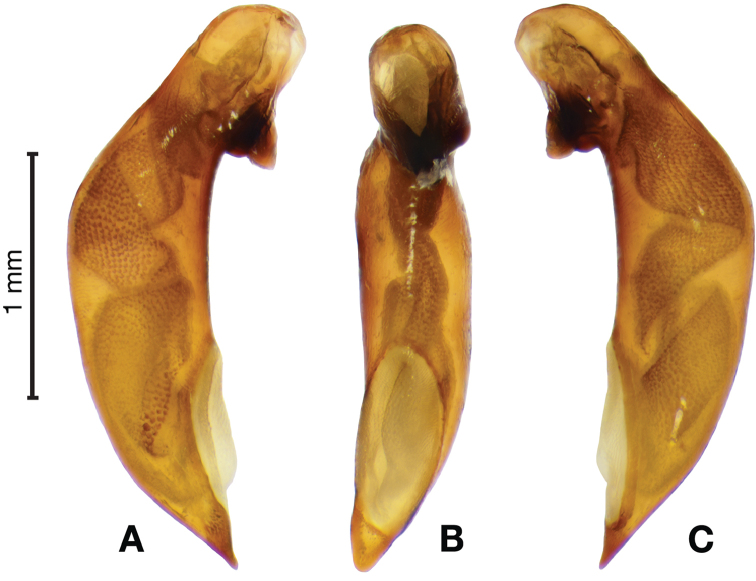
Digital images of male genitalia of C. (Coptoderina) japonica Bates. **A** right lateral aspect **B** ventral aspect **C** left lateral aspect.

Figs 41 , 42 , 43A–C , 57C , 59

Coptodera japonica Bates, 1883: 281; Csiki 1932: 1371; Jedlička 1963: 348; Lorenz 2005: 458; Park et al. 2011: 103.
Coptodera formosana Dupuis, 1912: 328; Jedlička 1963: 347.

########### Types and other material examined.

Cotype (male) labeled: “Japan./G. Lewis./1910-320”; “Cotype” [circular, ringed with green]”; “Ex Coll./Brit. Mus.”; “Coptodera/japonica/cotype Bates/H. E. Andrewes det.”; “Syntypus”[rectangular, red paper]. Eight specimens of *C.japonica*, three males and five females. For further details see EH Strickland Virtual Entomology Museum Database.

########### Type locality.

Japan: Kyushu (Kiushiu).

########### Diagnosis.

Specimens of this species are easily distinguished from other Taiwanese *Coptodera* by a combination of elytra with a seta in the apical 1/3 of stria 2 and black lateral margins.

########### Description.

OBL 9.16 – 10.33 mm. Length (n = three males, five females): head 1.00 – 1.12, pronotum 1.36 – 1.58, elytra 5.50 – 6.33, metepisternum 1.32 – 1.44 mm; width: head 2.00 – 2.20, pronotum 2.40 – 2.76, elytra 4.16 – 4.50, metepisternum 0.80 – 0.84 mm.

*Body proportions*. HW/HL 1.93 – 2.12; PWM/PL 1.64 – 1.82; EL/EW 1.30 – 1.42; ML/MW 1.57 – 1.67.

*Color*. Fig. 41. Dorsum of head piceous, area nearest to antennal socket brunneous to rufo-piceous, clypeus black with outer margin brunneous to rufo-brunneous, labrum, antennae, and palpi brunneous to rufo-brunneous; disc of pronotum rufo-piceous to piceous, lateral margins rufo-brunneous, only slightly lighter then disc; elytral disc black, with four testaceous maculae, two anterior and two posterior, anterior macula large, from interval 3 to 7 in most specimens, closest to base of elytra in interval 5 and 6, closest to apex of elytra in interval 4, posterior macula, from interval 2–8 (some from interval 1), closest to base of elytra in intervals 4 and 5, closest to apex of elytra in interval 4; margins of elytra rufo-brunneous to piceous; ventral surface rufo-brunneous to piceous; legs with trochanter and femora and tarsi brunneous to rufo-brunneous, tibia rufo-brunneous to piceous on dorsal surface.

*Microsculpture*. Dorsum of head with microsculpture somewhat granulate and isodiametric, easily visible at 50× magnification; pronotum somewhat granulate, isodiametric to somewhat transverse mesh pattern easily visible at 50×; elytral intervals with transverse sculpticells, center of striae with isodiametric sculpticells a few cells wide; ventral surface of head with microsculpture transverse, faintly visible at 50×; prosternum, proepipleuron, mesepisternum and metepisternum with sculpticells forming a shallow transverse mesh.

*Macrosculpture and pilosity*. Dorsum of head and clypeus smooth, with relatively dense, fine, scattered setigerous punctures, setae hardly visible at 50×; pronotum with relatively dense, fine and scattered setigerous punctures, visible in side view at 50×, disc shallowly rugulose; elytra with intervals broadly rounded, single row of fine setigerous punctures in the center of each interval, these punctures larger than additional scattered setigerous punctures throughout disc, striae with single row of fine setigerous punctures hardly visible in lateral view at 50×; ventrally, thoracic and abdominal sclerites with scattered setigerous punctures throughout.

*Fixed setae.* Elytra with two setae in apical 1/3 of stria 2, one seta near base in stria 3.

*Luster*. Dorsal and ventral surfaces moderately glossy.

*Head*. Mandibles somewhat curved at apex, relatively long and narrow in form, when measured on outside diameter, visible portion longer than length of labrum; labrum relatively stout and rounded, slightly wider than clypeus at max width, broadly bilobed to almost flat apically.

*Pronotum*. Transverse impression deep; posterior transverse impression deep, median longitudinal impression moderately deep; lateral margins explanate, apico-lateral margins broadly rounded, posterio-lateral margins obtuse.

*Elytra*. Apex slightly sinuate.

*Legs*. Two rows of small squamo-setae on tarsomeres 1–2 of mid-leg; males with two notches apically on ventral side of mid-tibia. Note: one individual with two notches on left mid-tibia and three notches on right mid-tibia has been observed.

*Male genitalia*. Figs 42, 43A–C Length 1.86 – 1.92 mm. Ostium catopic, positioned slightly to right when viewed ventrally, phallus cylindrical, slanting right from base of ostium towards apex when viewed ventrally, apical area with short, bluntly rounded apex; endophallus long and narrow, positioned to right of phallus when everted and viewed ventrally, microtrichia dispersed evenly and rather divergently on most of surface, one large and typically more dense microtrichial field (mtf) present on left side of phallus, just before apical constriction, sclerotized ring of spines (esr) at narrowest portion of apical constriction.

*Female genitalia*. 57C. Width 1.28 mm. One spermatheca (sp1), cylindrical and long, ribbed laterally along length, distinctively curved at base; one spermathecal accessory gland (sg), large and bulbous; spermathecal gland duct (sgd) only slightly longer than length of spermatheca, slightly swollen at apical end, just before gland, attachment site medially on distinctively shaped diverticulum (div) of spermatheca; bursa copulatrix (bc) with distinctive sac at apical end (bs), highly constricted near opening to common oviduct, then expanding out into and oblong mushroom top shaped chamber.

########### Habitat, habits, and seasonal occurrence.

The known elevational range of *C.japonica* is from 640 to 2770 meters. Adults of this species are found in mixed forest of montane areas. Little is known of the habits of this species but one specimen of this species was collected on shelf fungus. Specimens have been collected from April to October in Taiwan and methods of collecting include u.v. light, malaise trap, and hand collecting.

########### Geographical distribution.

*Coptoderajaponica* is known from Japan, Korea, and Taiwan. For Taiwan collecting localities see Figure 59.

########## Coptodera (Coptoderina) maculata

Taxon classificationAnimaliaColeopteraCarabidae

Dupuis

**Figure 44.**
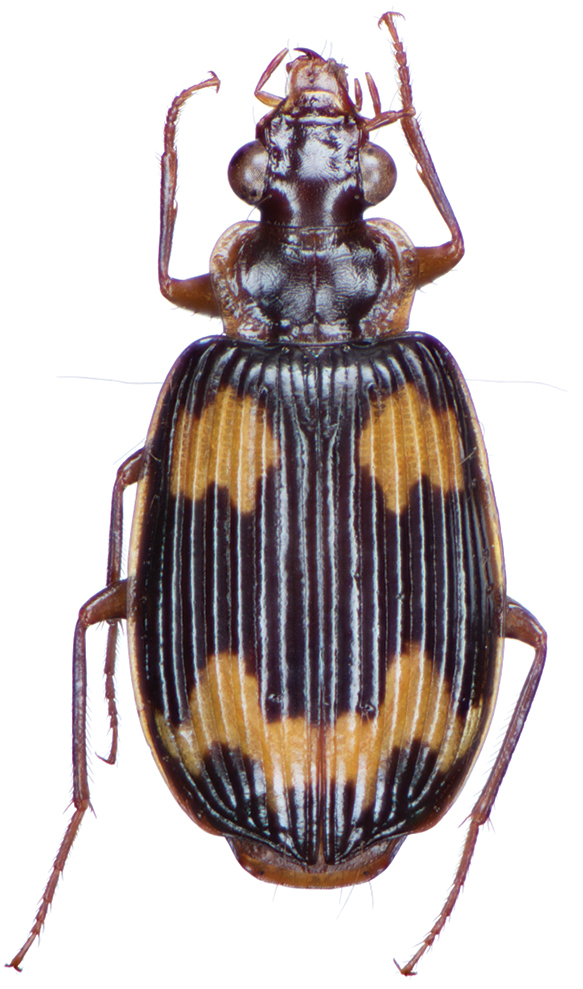
Dorsal habitus and color pattern of C. (Coptoderina) maculata (Dupuis). (OBL 6.66 mm).

**Figure 45.**
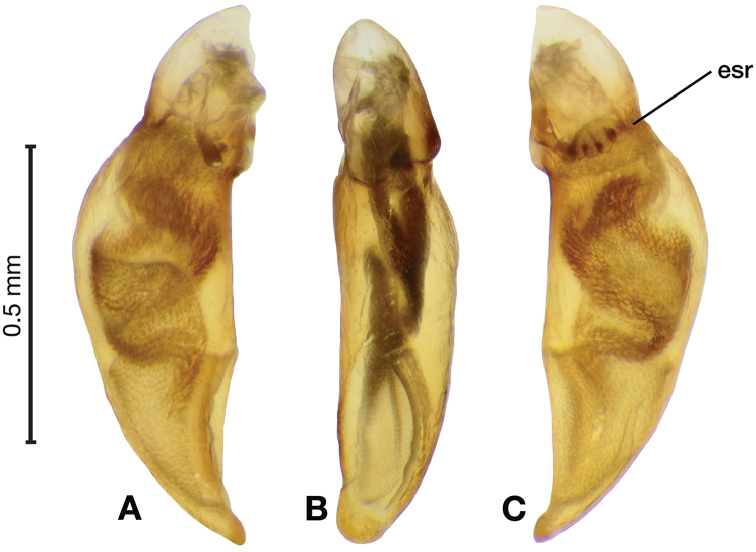
Digital images of male genitalia of C. (Coptoderina) maculata (Dupuis). **A** right lateral aspect **B** ventral aspect **C** left lateral aspect. Legend: **esr** endophallic sclerite ring.

**Figure 46.**
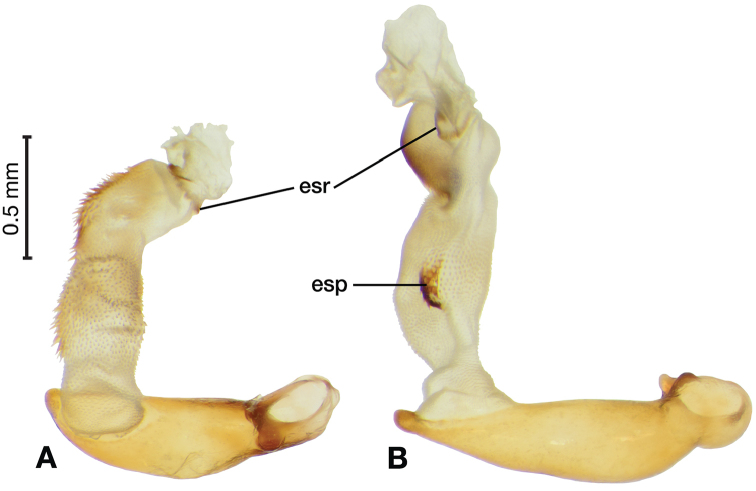
Digital images comparing the everted endophallus characteristics, left lateral aspect, of **A**C. (Coptoderina) maculata (Dupuis) **B**C. (Coptoderina) marginata (Dupuis) Legend: **esp** endophallic spine patch; **esr** endophallus sclerite ring.

Figs 44 , 45A–C , 46A , 57D , 59

Coptodera formosana var. maculata Dupuis, 1912: 329.
Coptodera maculata Dupuis: Nakane and Ohkura 1956: 48; Jedlička 1963: 342.

########### Types and other material examined.

**Holotype** (female) labeled: “Hoozan/Formosa/H. Sauter II 10”; “TYPUS” [rectangular, red paper]; “DUPUIS DET.”; “Coptodera/formosana D/maculata D”. One **paratype** and six other specimens of *C.maculata*: five males and one female. For further details see EH Strickland Virtual Entomology Museum Database.

########### Type locality.

Taiwan (Formosa: Hoozan).

########### Diagnosis.

Specimens of this species are easily distinguished from other Taiwanese *Coptodera* by a combination of elytra with one seta in apical 1/4 of stria 3, one seta in apical 1/3 of stria 2, light lateral margins, and an apical macula that extends to suture.

########### Description.

OBL 6.08 – 6.66 mm. Length (n = five males, one female): head 0.78 – 0.92, pronotum 1.04 – 1.20, elytra 4.33 – 5.00, metepisternum 0.96 – 1.20 mm; width: head 1.52 – 1.72, pronotum 1.76 – 2.08, elytra 3.16 – 3.66, metepisternum 0.60 – 0.68 mm.

*Body proportions*. HW/HL 1.86 – 2.00; PWM/PL 1.57 – 1.79; EL/EW 1.32 – 1.40; ML/MW 1.56 – 1.76.

*Color*. Fig. 44. Dorsum of head brunneous to rufo-piceous, clypeus, antennae, and palpi brunneous to rufo-brunneous; disc of pronotum brunneous to rufo-piceous, lateral margins testaceous to rufo-brunneous, contrastingly lighter then disc; elytral disc, with four testaceous maculae, two anterior and two posterior, anterior macula large, from interval 3 to 7, closest to base of elytra in interval 5, closest to apex of elytra in interval 4, posterior macula, from suture to interval 8, closest to base of elytra in intervals 4 and 5, closest to apex of elytra in interval 3; margins of elytra testaceous to rufo-brunneous; ventral surface with thoracic and abdominal tergites rufo-testaceous to rufo-piceous, proepipleuron and elytral epipleuron contrastingly lighter, brunneo-testaceous to rufo-brunneous; legs with trochanter and femora and tarsi brunneo-testaceous to rufo-brunneous, tibia rufo-brunneous to piceous on dorsal surface.

*Microsculpture*. Dorsum of head with sculpticells granulate and isodiametric, easily visible at 50× magnification; pronotum somewhat granulate, transverse mesh pattern easily visible at 50×; elytral intervals with transverse sculpticells, center of striae with isodiametric sculpticells, one to two cells wide; ventral surface of head, smooth with microsculpture not visible at 50×; prosternum, proepipleuron, mesepisternum and metepisternum with sculpticells forming a shallow, somewhat transverse to transverse mesh.

*Macrosculpture*. Dorsum of head and clypeus faintly rugulose to smooth, with relatively dense, fine and scattered setigerous punctures, setae hardly visible at 50×; pronotum with fine and scattered setigerous punctures, visible in side view at 50×, disc shallowly regulose; elytra with intervals broadly rounded, single row of fine setigerous punctures in the center of each interval, these punctures larger than additional scattered setigerous puncture throughout disc, striae with single row of fine setigerous punctures hardly visible in lateral view at 50×; ventrally, thoracic and abdominal sclerites with scattered setigerous punctures throughout.

*Fixed setae.* Elytra with two setae in apical 1/3 of stria 2, one seta near base in stria 3.

*Luster*. Dorsal and ventral surfaces moderately glossy.

*Head*. Mandibles somewhat curved at apex, somewhat long and narrow in form, when measured on outside diameter, visible portion shorter than length of labrum; labrum slightly wider than clypeus at max width, broadly bilobed.

*Pronotum*. Anterior transverse impression deep; posterior transverse impression deep, median longitudinal impression moderately deep; lateral margins explanate, apico-lateral margins broadly rounded, posterio-lateral margins obtuse.

*Elytra*. Apex slightly sinuate.

*Legs*. Two rows of small squamo-setae on tarsomeres 1–2 of mid-leg; males with two notches apically on ventral side of mid-tibia.

*Male genitalia*. Figs 45A–C, 46A. Length 1.08 – 1.44 mm. Ostium catopic. Phallus cylindrical, right side straight along length, left side curving slightly from mid-length to apex when viewed ventrally, distinctively wide medially in lateral view, apical area very short and bluntly rounded apex, slightly curved upwards in lateral view; endophallus, relatively short and stout, one large and distinctive microtrichial field, microtrichia longer and more dense on right side, sclerotized ring of distinctively large spines (esr) at narrowest portion of apical constriction.

*Female genitalia*. Fig. 57D. Width 0.96 mm. One spermatheca (sp1), cylindrical, ribbed laterally along length; spermathecal accessory gland not observed; spermathecal gland (sgd) duct broken before apex but distinctively swollen along length, attachment site near apex of large diverticulum (div) of spermatheca; bursa copulatrix (bc) with distinctive sac at apical end (bs), large and somewhat cylindrical, coming to a point at the apex.

########### Habitat, habits, and seasonal occurrence.

The known elevational range of *C.maculata* is from 100 to 950 meters. Adults of this species are found in mixed forest of montane areas. Little is known of the habits of this species. Specimens have been collected from January to September with the most being collected in June. Methods of collecting include u.v. light sheet, light trap, and hand collecting.

########### Geographical distribution.

*Coptoderamaculata* is known only from Taiwan. See Figure 59.

########## Coptodera (Coptoderina) marginata

Taxon classificationAnimaliaColeopteraCarabidae

Dupuis

**Figure 47.**
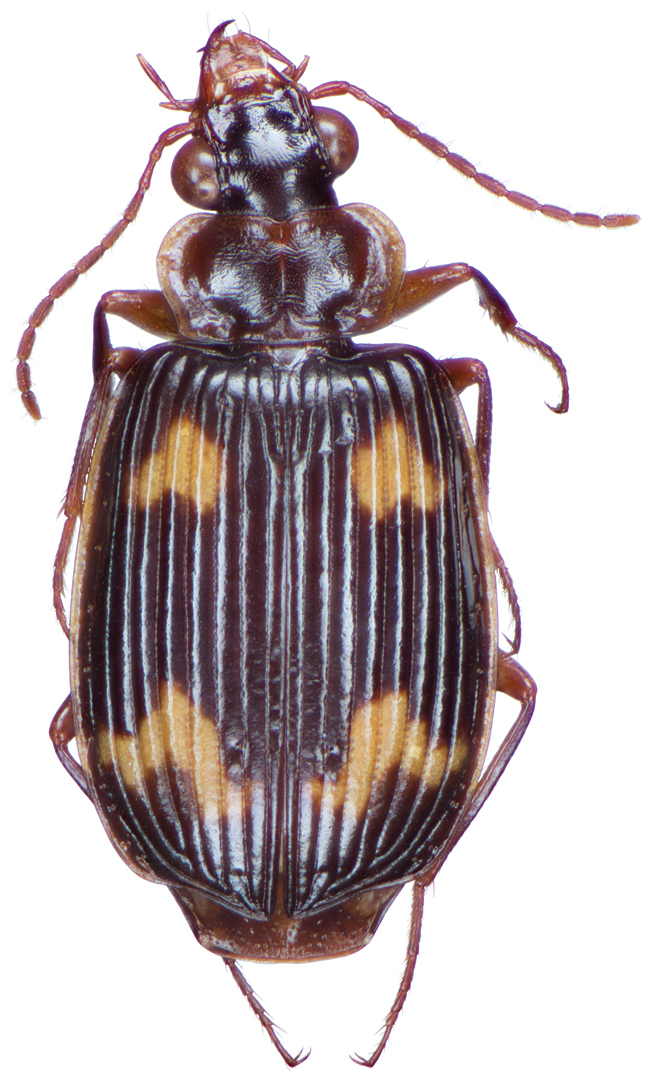
Dorsal habitus and color pattern of C. (Coptoderina) marginata (Dupuis). (OBL 9.24 mm).

**Figure 48.**
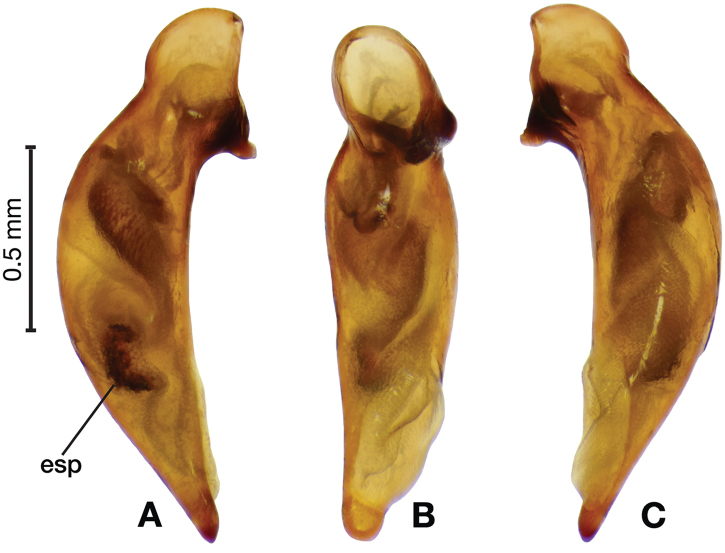
Digital images of male genitalia of C. (Coptoderina) marginata (Dupuis). **A** right lateral aspect **B** ventral aspect **C** left lateral aspect. Legend: **esp** endophallic spine patch.

Figs 46b , 47 , 48A–C , 58A , 59

Coptodera formosana var. marginata Dupuis, 1912: 329.
Coptodera marginata Dupuis: Nakane and Ohkura 1956: 48; Habu 1967: 88; Lorenz 2005: 458.

########### Types and other material examined.

Paratype (female) labeled: “Hoozan/Formosa/H. Sauter II 10”; “Paratyp” [rectangular, red paper]; “DUPUIS DET.”; “Coptodera/formosana/marginata/D”. six specimens of *C.marginata*, five males and one female. For further details see EH Strickland Virtual Entomology Museum Database.

########### Type locality.

Taiwan (Formosa: Hoozan).

########### Diagnosis.

Specimens of this species are easily distinguished from other Taiwanese *Coptodera* by a combination of elytra with one seta in apical 1/4 of stria 3, one seta in apical 1/3 of stria 2, light lateral margins and an apical macula that does not extend to suture.

########### Description.

OBL 8.16 – 9.83 mm. Length (n = five males, one female): head 0.88 – 1.00, pronotum 1.16 – 1.44, elytra 5.16 – 6.16, metepisternum 1.20 – 1.32 mm; width: head 1.76 – 2.04, pronotum 2.24 – 2.68, elytra 3.66 – 4.66, metepisternum 0.68 – 0.84 mm.

*Body proportions*. HW/HL 1.91 – 2.14; PWM/PL 1.79 – 1.97; EL/EW 1.30 – 1.42; ML/MW 1.57 – 1.76.

*Color*. Fig. 47. Dorsum of head brunneous to rufo-piceous, area nearest to antennal socket brunneous to rufo-brunneous, clypeus, antennae, and palpi brunneous to rufo-brunneous; disc of pronotum brunneous to rufo-piceous, lateral margins testaceous to rufo-brunneous, always contrastingly lighter then disc; elytral disc, with four testaceous maculae, two anterior and two posterior, anterior macula large, from interval 3 to 7 in most specimens, closest to base of elytra in interval 5 and 6, closest to apex of elytra in interval 4, posterior macula, from interval 2 to 8, closest to base of elytra in intervals 4 and 5, closest to apex of elytra in interval 3 or 4; margins of elytra rufo-testaceous to brunneous; ventral surface with thoracic and abdominal tergites rufo-brunneous to rufo-piceous, proepipleuron and elytral epipleuron always contrastingly lighter, brunneo-testaceous to rufo-brunneous; legs with trochanter and femora and tarsi brunneous to rufo-brunneous, tibia rufo-brunneous to piceous on dorsal surface.

*Microsculpture*. Dorsum of head with microsculpture somewhat granulate and isodiametric on center of head and towards neck, outer portions less granulate to almost flat, easily visible at 50×; pronotum somewhat granulate, isodiametric to somewhat transverse mesh pattern easily visible at 50×; elytral intervals with transverse sculpticells, center of striae with isodiametric sculpticells a few cells wide; ventral surface of head with microsculpture transverse, faintly visible at 50×; prosternum, proepipleuron, mesepisternum and metepisternum with sculpticells forming a shallow, somewhat transverse to transverse mesh.

*Macrosculpture and pilosity*. Dorsum of head and clypeus faintly rugulose, with relatively dense, fine, scattered setigerous punctures, setae hardly visible at 50×; pronotum with relatively dense, fine and scattered setigerous punctures, visible in side view at 50×, disc shallowly rugulose; elytra with intervals broadly rounded, single row of fine setigerous punctures in the center of each interval, these punctures larger than additional scattered setigerous puncture throughout disc, striae with single row of fine setigerous punctures hardly visible in lateral view at 50×; ventrally, thoracic and abdominal sclerites with scattered setigerous punctures throughout.

*Fixed setae.* Elytra with two setae in apical 1/3 of stria 2, one seta near base in stria 3.

*Luster*. Dorsal and ventral surfaces moderately glossy.

*Head*. Mandibles somewhat curved at apex, somewhat long and narrow in form, when measured on outside diameter, visible portion always shorter than length of labrum; labrum slightly wider than clypeus at max width, broadly bilobed.

*Pronotum*. Anterior transverse impression deep; posterior transverse impression deep, median longitudinal impression moderately deep; lateral margins explanate, apico-lateral margins broadly rounded, posterio-lateral margins obtuse.

*Elytra*. Apex slightly sinuate.

*Legs*. Two rows of small squamo-setae on tarsomeres 1–2 of mid-leg; males with two notches apically on ventral side of mid-tibia.

*Male genitalia*. Figs 46B, 48A–C. Length 1.54 – 1.64 mm. Ostium catopic, opening positioned slightly left of center. Phallus cylindrical, right side straight along length, left side curving from mid-length to apex when viewed ventrally, apical area with short, bluntly rounded apex; endophallus long and narrow, with constriction near base, two distinctive microtrichial fields, one large one towards apical end, one smaller and more dense one (esp) between basal constriction and larger microtrichial field, sclerotized ring of spines at narrowest portion of apical constriction.

*Female genitalia*. Fig. 58A. Width 1.38 mm. The single female specimen dissected was in very poor condition and all other accessory organs were destroyed in prep.

########### Habitat, habits, and seasonal occurrence.

The known elevational range of *C.marginata* is from 1000 to 1200 meters. Adults of this species are found in mixed forest of montane areas. Little is known of the habits of this species. Specimens have been collected from April to September in Taiwan and methods of collecting include malaise trap and hand collecting.

########### Geographical distribution.

*Coptoderamarginata* is known from Japan and Taiwan. For Taiwan collecting localities see Figure 60.

########## Coptodera (Coptoderina) occulta sp. n.

Taxon classificationAnimaliaColeopteraCarabidae

http://zoobank.org/323C7DCE-566E-4A9D-A9F9-346254056831

**Figure 49.**
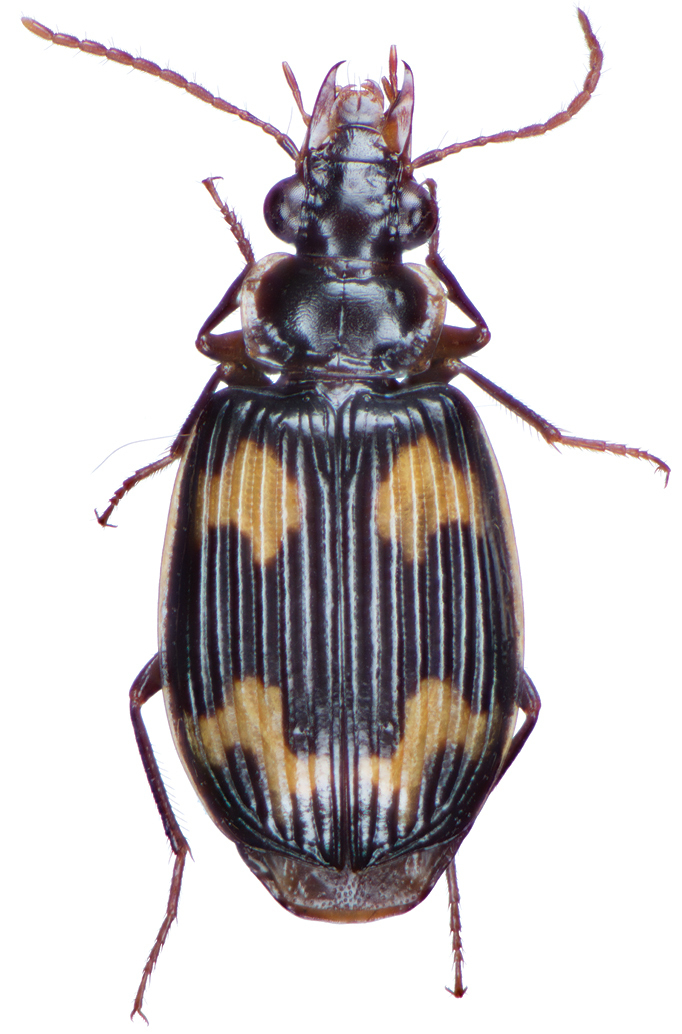
Dorsal habitus and color pattern of C. (Coptoderina) occulta sp. n.. (OBL 8.24 mm).

**Figure 50.**
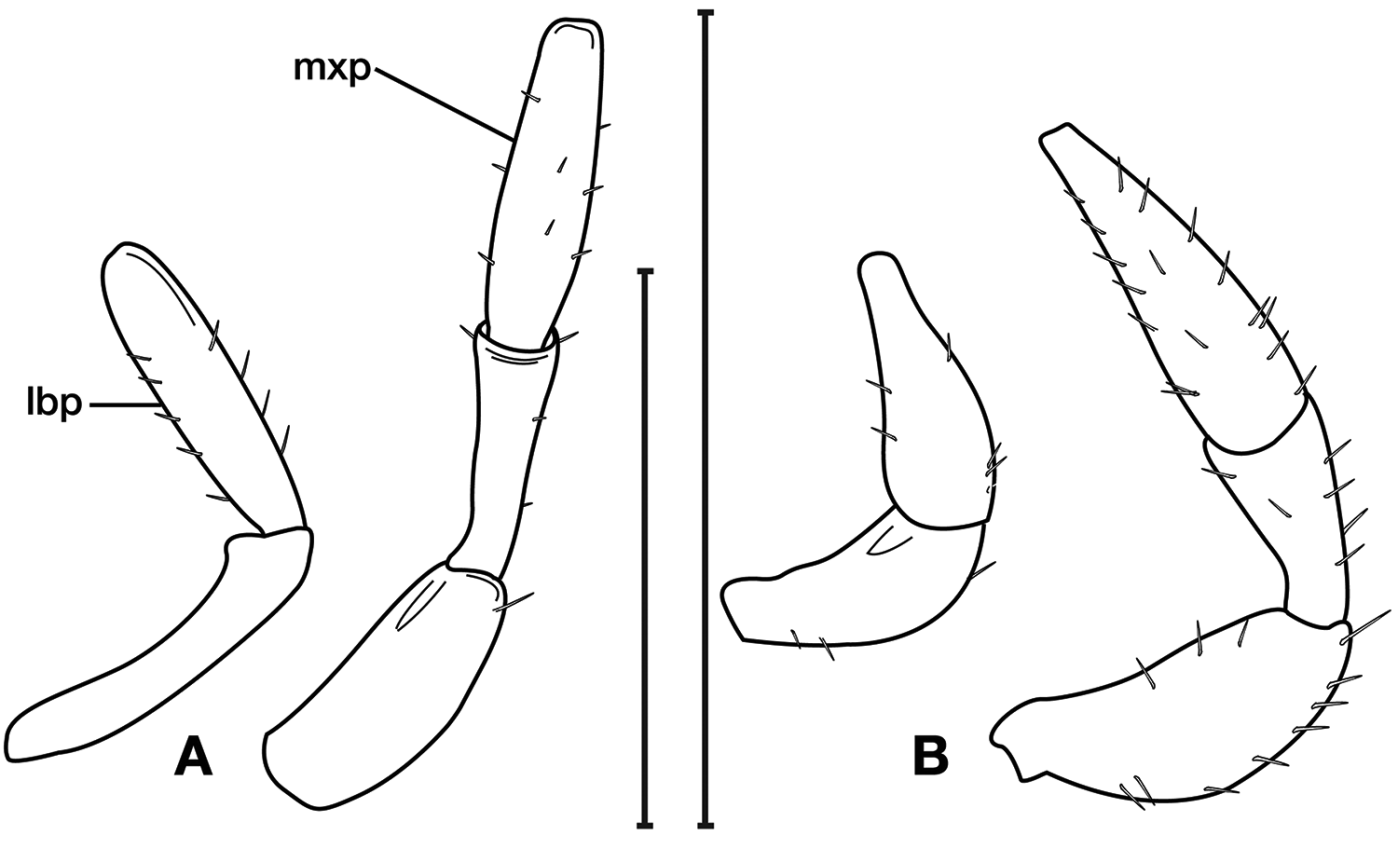
Line drawings comparing the form and setae pattern of labial (**lbp**) and maxillary palpi (**mxp**), right ventral aspect, of **A**C. (Coptoderina) occulta sp. n. and **B***Formosiellaflavomaculata* (Shibata). Scale bars: 0.5 mm.

**Figure 51.**
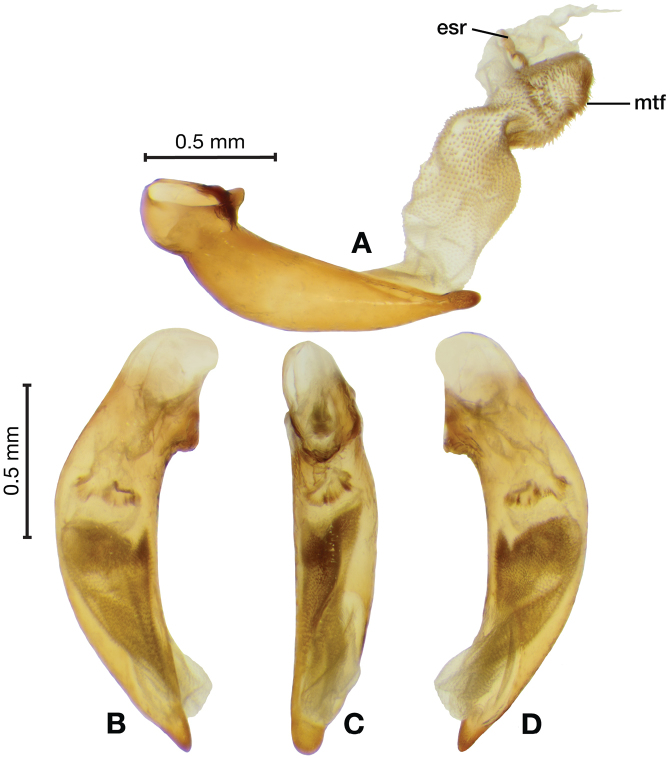
Digital images of male genitalia of C. (Coptoderina) occulta sp. n.. **A** right lateral aspect, endophallus everted **B** right lateral aspect **C** ventral aspect **D** left lateral aspect. Legend: **esr** endophallus sclerite ring; **mtf** microtrichial field.

Figs 49 , 50A , 51A–D , 58B , 60

########### Specific epithet.

From Latin *occulta*, meaning hidden or mysterious. Previous to this work only two specimens were known from collections.

########### Types and other material examined.

**Holotype** (male) labeled “Holotype” [circular, ringed with red]; “TAIWAN Pingdong co./Lanren River/2011.IV.30 by light”; leg. Kenting National Park Insect Survey/22°2'37.3"N, 120°51'5.5"E/#348”; “6446”; “NCHU# 101601”. Seven **paratypes** of *C.occulta*: one male and six females. For further details see EH Strickland Virtual Entomology Museum Database.

########### Type locality.

Taiwan. Lanren River area, Pingdong county.

########### Diagnosis.

This species is most similar to *C.japonica* but is easily distinguished by the testaceous margin of the pronotum (black or just slightly lighter in color in *C.japonica*) and the almost straight elytral apex.

########### Description.

OBL 7.33 – 8.67 mm. Length (n = two males, six females): head 0.80 – 0.92, pronotum 1.12 – 1.28, elytra 4.33 – 5.33, metepisternum 1.08 – 1.36 mm; width: head 1.60 – 1.88, pronotum 1.84 – 2.24, elytra 3.16 – 3.83, metepisternum 0.64 – 0.72 mm.

*Body proportions*. HW/HL 1.91 – 2.12; PWM/PL 1.64 – 1.75; EL/EW 1.27 – 1.43; ML/MW 1.5 – 2.13.

*Color*. Fig. 49. Dorsum of head rufo-piceous to piceous, area nearest to antennal socket brunneous to rufo-piceous, clypeus and labrum with lateral margins testaceous to brunneo-testaceous, centrally, brunneo-piceous to piceous, antennae and palpi brunneo-testaceous to rufo-brunneous; disc of pronotum rufo-brunneous to rufo-piceous, margins lighter, brunneo-testaceous to rufo-brunneous; elytral disc black, with four testaceous maculae, two anterior and two posterior, anterior macula large, from interval 3 to 8, closest to base of elytra in interval 5, closest to apex of elytra in interval 4, posterior macula, from interval 2 to 8 (sometimes 1), closest to base of elytra in intervals 4 and 5, closest to apex of elytra in interval 4; margins of elytra rufo-brunneous to rufo-piceous; ventral surface rufo-brunneous to piceous; legs with trochanter and femora and tarsi brunneous to rufo-brunneous, tibia rufo-brunneous to piceous on dorsal surface.

*Microsculpture*. Dorsum of head with microsculpture somewhat granulate and isodiametric, easily visible at 50× magnification; pronotum somewhat granulate, transverse to almost isodiametric in margins, mesh pattern easily visible at 50×; elytral intervals with transverse sculpticells, center of striae with isodiametric sculpticells a few cells wide; ventral surface of head with microsculpture not visible at 50×; prosternum, proepipleuron, mesepisternum and metepisternum with sculpticells forming a shallow transverse to nearly isodiametric mesh.

*Macrosculpture*. Dorsum of head and clypeus smooth, with fine and scattered setigerous punctures, setae hardly visible at 50×; pronotum with fine, scattered setigerous punctures, visible in side view at 50×, disc shallowly rugulose; elytra with intervals rounded, single row of fine setigerous punctures in the center of each interval, these punctures larger than additional scattered setigerous puncture throughout disc, striae punctate, with single row of fine setigerous punctures hardly visible in lateral view at 50×; ventrally, thoracic and abdominal sclerites with scattered setigerous punctures throughout.

*Fixed setae.* Elytra with two setae in apical 1/3 of stria 2, one seta near base in stria 3.

*Luster*. Dorsal and ventral surfaces moderately glossy.

*Head*. Fig. 50A. Mandibles somewhat curved at apex, relatively long and narrow in form, when measured on outside diameter, visible portion shorter than length of labrum; labrum stout and rounded, slightly wider than clypeus at max width, broadly bilobed and widely emarginated.

*Pronotum*. Anterior transverse impression deep; posterior transverse impression deep; median longitudinal impression moderately deep; lateral margins explanate, apico-lateral margins broadly rounded, posterio-lateral margins obtuse.

*Elytra*. Apex slightly sinuate.

*Legs*. Two rows of small squamo-setae on tarsomeres 1–2 of mid-leg, males with two notches apically on ventral side of mid-tibia.

*Male genitalia*. Fig. 51A–D. Length 1.32 – 1.34 mm. Ostium slightly left pleuropic. Phallus cylindrical, right side straight along length, left side curving slightly from mid-length to apex of ostium when viewed ventrally, apical area with short, bluntly rounded apex; endophallus moderately short and stout, microtrichia dispersed somewhat evenly in apical portion (mtf), small microtrichia in basal 1/3 and increasing in length towards apex, sclerotized ring of spines (esr) near apex .

*Female genitalia*. Fig. 58B. Width 1.04 mm. One spermatheca (sp1), cylindrical and long, ribbed laterally along length; one spermathecal accessory gland (sg), large and distinctively elongate; spermathecal gland duct (sgd) longer than length of spermatheca, slightly swollen at apical end, just before gland, attachment site on apex of bilobed diverticulum (div) of spermatheca; bursa copulatrix (bc) with distinctive, sac at apical end (bs), constricted near opening to common oviduct, then expanding out into a distinctively large chamber.

########### Habitat, habits, and seasonal occurrence.

The known elevational range of *C.occulta* sp. n. is from 200 to 640 meters. Adults of this species are found in mixed forest of montane areas. Little is known of the habits of this species. Specimens have been collected from March to September in Taiwan and methods of collecting include u.v. light, light trap, flight intercept trap, and hand collecting.

########### Geographical distribution.

*Coptoderaocculta* is known only from Taiwan. See Figure 60.

########## Coptodera (Coptoderina) proksi

Taxon classificationAnimaliaColeopteraCarabidae

Jedlička

**Figure 52.**
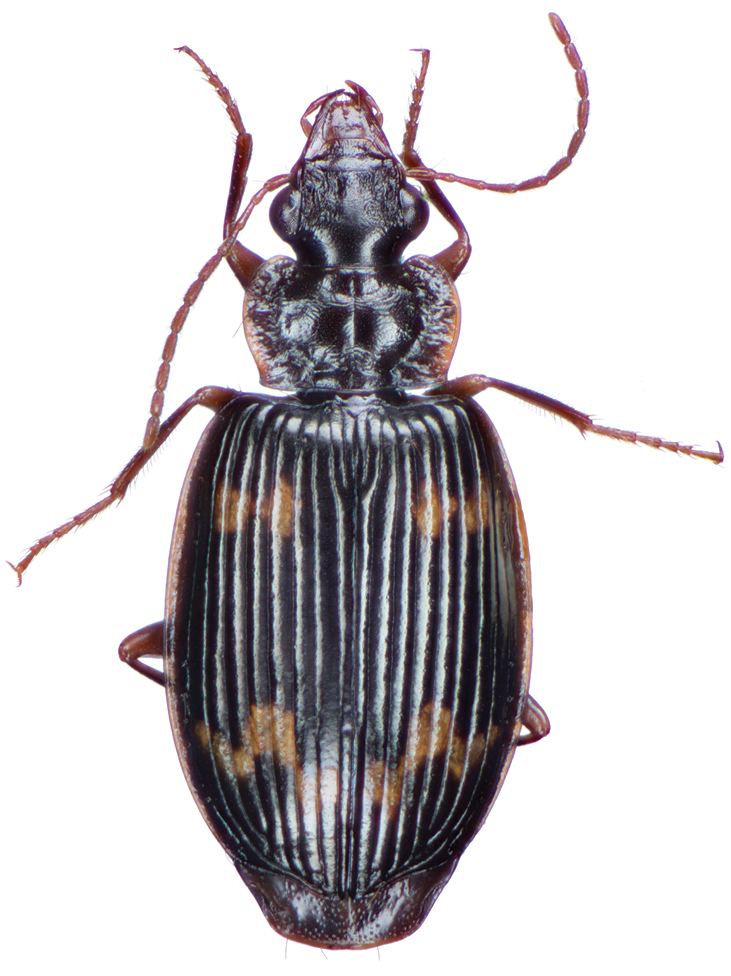
Dorsal habitus and color pattern of C. (Coptoderina) proksi Jedlička. (OBL 6.98 mm).

**Figure 53.**
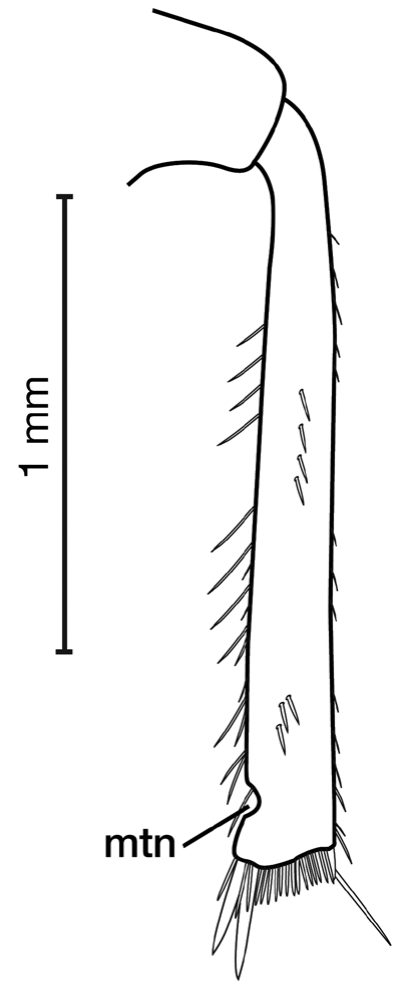
Line drawing of the meso-tibia of C. (Coptoderina) proksi Jedlička, showing seta pattern and placement of meso-tibial notch (**mtn**).

**Figure 54.**
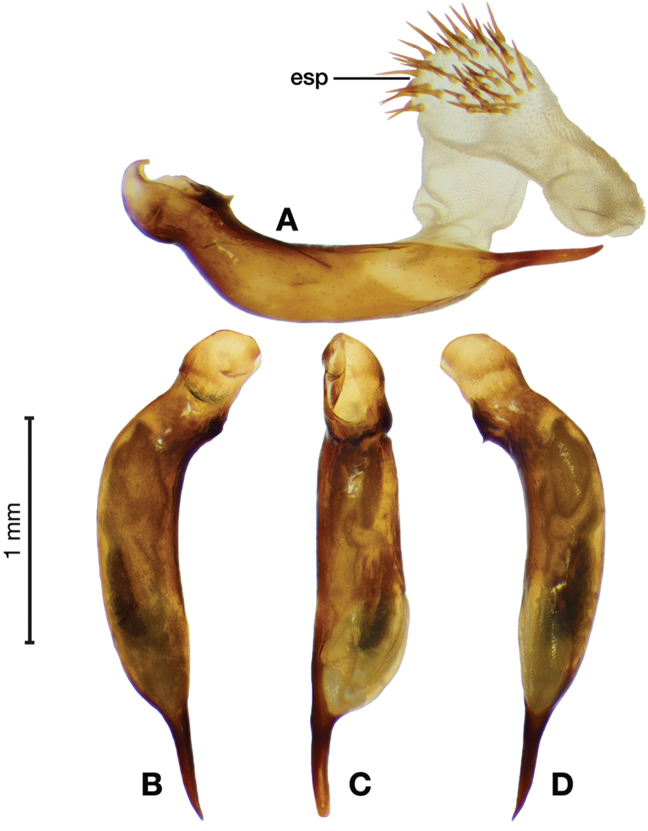
Digital images of male genitalia of C. (Coptoderina) proksi Jedlička. **A** right lateral aspect, endophallus everted **B** right lateral aspect **C** ventral aspect **D** left lateral aspect. Legend: **esp** endophallic spine patch.

Figs 52 , 53 , 54A–D , 58C , 60

Coptodera proksi Jedlička, 1963: 345; Lorenz 2005: 458.

########### Types and other material examined.

**Holotype** (female) labeled “Formosa/H. Kono”; “TYPUS” [red rectangle, black border]; “Mus Nat. Pragae/Inv. 65 210”; Proksi sp.n./det.ING.JEDLICKA”; “NCHU# 101670”. 54 specimens of *C.proksi*: 38 males and 16 females. For further details see EH Strickland Virtual Entomology Museum Database.

########### Type locality.

Taiwan.

########### Diagnosis.

Specimens of this species are easily distinguished from other Taiwanese *Coptodera* by a combination of elytra with one seta in apical 1/4 of stria 3 and no setae in stria 2.

########### Description.

OBL 6.66 – 8.50 mm. Length (n = ten males, ten females): head 0.62 – 0.80, pronotum 1.08 – 1.40, elytra 4.08 – 5.16, metepisternum 0.96 – 1.32 mm; width: head 1.40 – 1.72, pronotum 1.86 – 2.36, elytra 3.16 – 3.83, metepisternum 0.52 – 0.72 mm.

*Body proportions*. HW/HL 2.00 – 2.37; PWM/PL 1.47 – 1.81; EL/EW 1.29 – 1.42; ML/MW 1.69 – 2.08.

*Color*. Fig. 52. Dorsum of head rufo-piceous to piceous, clypeus rufo-brunneous to rufo-piceous, always lighter than head, labrum rufo-brunneous to rufo-piceous, always lighter than clypeus, antennae and palpi brunneous to rufo-brunneous; disc of pronotum rufo-piceous to piceous, lateral margins rufous to rufo-piceous, always somewhat lighter then disc; disc of elytra black, with four testaceous to rufo-testaceous maculae, two anterior and two posterior, anterior macula relatively narrow, variable, from interval 3 or 4 to 7 or 8, closest to base of elytra in interval 7 (when reduced, sometimes 4), closest to apex of elytra in interval 4, posterior macula relatively narrow, from interval 1 (sometimes suture) to interval 8, closest to base of elytra in intervals 4 and 5, closest to apex of elytra in interval 3; margins of elytra brunneous to rufo-brunneous; ventral surface with thoracic and abdominal tergites brunneous to rufo-piceous; legs with trochanter and femora and tarsi brunneous to rufo-brunneous, tibia rufo-brunneous to piceous on dorsal surface.

*Microsculpture and pilosity*. Dorsum of head with microsculpture somewhat granulate and +/- isodiametric, easily visible at 50× magnification; pronotum variable, transverse on disc to almost isodiametric near margins; elytral intervals with transverse sculpticells, center of striae with isodiametric sculpticells, one to two cells wide; ventral surface of head, smooth with microsculpture not visible at 50×. Prosternum, proepipleuron, mesepisternum and metepisternum with sculpticells forming a shallow, somewhat transverse to transverse mesh.

*Macrosculpture*. Dorsum of head somewhat rugulose between eyes, clypeus faintly rugulose to smooth, both head and clypeus with relatively dense, fine and scattered setigerous punctures, setae hardly visible at 50×; pronotum with fine and scattered setigerous punctures, visible in side view at 50×, disc shallowly rugulose to smooth; elytra with intervals somewhat flat, entire dorsal surface with fine scattered setigerous punctures, hardly visible in lateral view at 50× , striae impunctate; ventrally, thoracic and abdominal sclerites with scattered setigerous punctures throughout.

*Fixed setae.* Elytra with one seta near apex of stria 2, one seta near base in stria 3; ventrally, prosternal process with dense, circular patch of setae at base in males.

*Luster*. Dorsal and ventral surfaces moderately glossy.

*Head*. Mandibles somewhat curved at apex, somewhat long and narrow in form, when measured on outside diameter, visible portion approximately half the length of the labrum; labrum bilobed, right lobe occasionally slightly longer than left.

*Pronotum*. Anterior transverse impression shallow; posterior transverse impression moderately shallow, median longitudinal impression moderately shallow; lateral margins explanate, apico-lateral margins rounded into distinctive lobes, posterio-lateral margins obtuse.

*Elytra*. Apex slightly sinuate to almost truncate.

*Legs*. Fig. 53. Two rows of small squamo-setae on tarsomeres 1–2 of mid-leg, finer and more difficult to observe than other species, males with one meso-tibial notch (mtn) apically on ventral side of meso-tibia.

*Male genitalia*. Fig. 54A–D. Length 1.80 – 1.92 mm. Ostium left pleuropic. Phallus cylindrical, right side straight along length, left side curving slightly from mid-length to apex of ostium when viewed ventrally, apical area distinctively long and narrow along length, rounded apex, slightly curved upwards in lateral view; endophallus, relatively short and stout, distinctive form, bent medially, one large field of long spines (esp) on outer surface of medial bend.

*Female genitalia*. Fig. 58C. Width 1.20 mm. One spermatheca (sp1), cylindrical, ribbed laterally along length, bent medially; one spermathecal accessory gland, relatively long and narrow in form; spermathecal gland (sgd) duct slightly swollen in apical half, attachment site near apex of diverticulum (div) of spermatheca; bursa copulatrix (bc) with bursa split into two portions basally, left side with large bursal diverticulum (bd) of similar width as remaining bursa on right side, main duct of bursa with distinctive sac at apical end (bs), large and somewhat spherical in form, additional tissue behind chamber with many infoldings.

########### Habitat, habits, and seasonal occurrence.

The known elevational range of *C.proksi* is from 640 to 2095 meters. Notably, only one of the 54 specimens collected was found at 640 meters, all remaining specimens were collected from 1400 to 2095 meters. Adults of this species are found in mixed forest of montane areas. This species is crepuscular and found on both trunks of live trees and recently dead or dying trees as night. Specimens have been collected from May to September with most being collected in July. Methods of collecting include u.v. light sheet, m.v. light sheet, light trap, at sugar bait, and hand collecting.

########### Geographical distribution.

*Coptoderaproksi* is known only from Taiwan. See Figure 60.

########## Coptodera (Coptoderina) taiwana

Taxon classificationAnimaliaColeopteraCarabidae

(Nakane)

**Figure 55.**
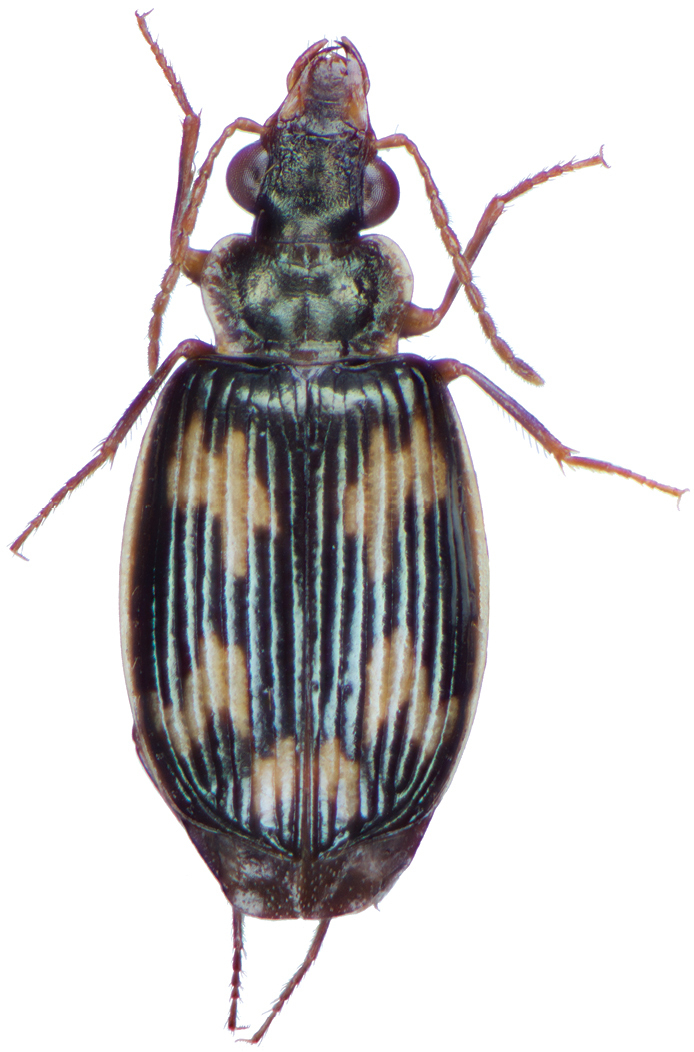
Dorsal habitus and color pattern of C. (Coptoderina) taiwana (Nakane). (OBL 6.02 mm).

**Figure 56.**
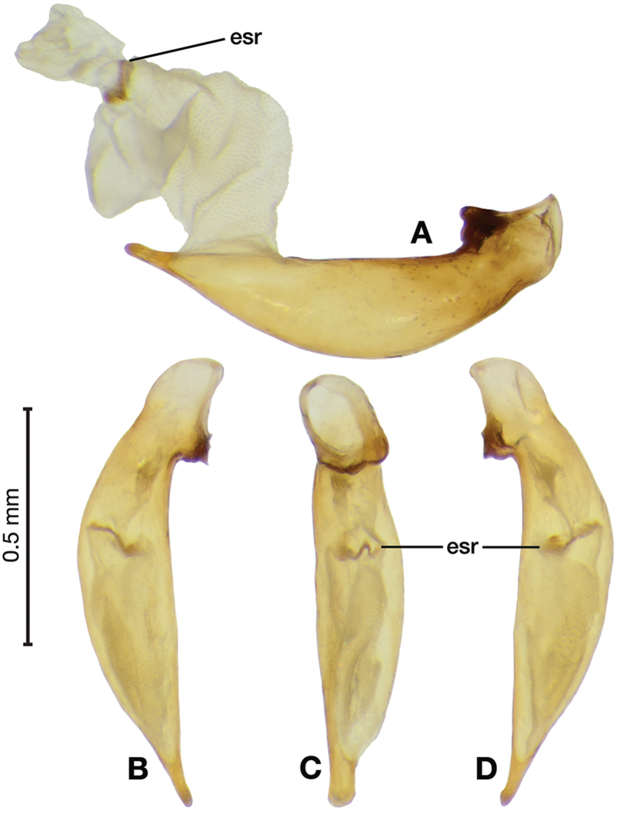
Digital images of male genitalia of C. (Coptoderina) taiwana (Nakane). **A** left lateral aspect, endophallus everted **B** right lateral aspect **C** ventral aspect **D** left lateral aspect. Legend: **esr** endophallus sclerite ring.

Figs 55 , 56A–D , 58D , 60

Coptoderina esakii taiwana Nakane, 1956: 104; Nakane and Ohkura 1956: 46; Jedlička 1963: 348.

########### Types and other material examined.

**Holotype** (male) labeled “HOLOTYPE” [rectangular, red paper]; “27 VII 1936/Tyokakurai/DAIBU”; From Coll./Asahina”; NAKANE Coll./SEHU JAPAN/1999”; “0000005409/Sys. Ent/Hokkaido Univ./Japan [SEHU]; “HOLOTYPE/Appended label by/N. Inari/2008; “NCHU#/101663”. One **allotype** (female on same pin as type specimen) and 62 other specimens of *C.taiwana*: 35 males and 27 females. For further details see EH Strickland Virtual Entomology Museum Database.

########### Type locality.

Taiwan. Taitung county, Dawu township.

########### Diagnosis.

Specimens of this species are easily distinguished from other Taiwanese *Coptodera* by a head a pronotum that is metallic green.

########### Description.

OBL 5.00 – 6.33 mm. Length (n = ten males, ten females): head 0.56 – 0.74, pronotum 0.80 – 1.00, elytra 3.00 – 3.83, metepisternum 0.68 – 0.88 mm; width: head 1.10 – 1.40, pronotum 1.36 – 1.80, elytra 2.33 – 3.00, metepisternum 0.40 – 0.52 mm.

*Body proportions*. HW/HL 1.77 – 2.07; PWM/PL 1.67 – 1.82; EL/EW 1.26 – 1.42; ML/MW 1.55 – 1.70.

*Color*. Fig. 55. See notes on coloration below. Dorsum of head piceous with faint metallic sheen, clypeus rufo-brunneous to rufo-piceous, always lighter than head, labrum rufo-brunneous to rufo-piceous, always slightly lighter than clypeus, contrasting with testaceous mandible base, antennae and palpi testaceous to rufo-brunneous; disc of pronotum brunneo-piceous to piceous, with faint metallic sheen, lateral margins testaceous to rufo-brunneous, always lighter then disc; disc of elytra black, with four testaceous macula, two anterior and two posterior, variable, anterior macula from interval 2 or 3 to 6 or 7, closest to base of elytra in interval 6 (when reduced, sometimes 4), closest to apex of elytra in interval 4, macula distinctively long in interval 4 compared to other intervals; posterior macula from suture (few specimens from interval 1) to interval 8 (few specimens to interval 7), closest to base of elytra in interval 5, closest to apex of elytra in interval 3; margins of elytra brunneous to rufo-brunneous; ventral surface with thoracic and abdominal tergites rufo-brunneous to rufo-piceous, margins of abdominal tergites and metepisternum darker; legs contrastingly lighter, with trochanter and femora and tarsi testaceous to brunneo-testaceous, tibia rufo-brunneous to darker on dorsal surface.

########### Notes on coloration.

In certain light conditions this species appears to have a metallic green to cupreous head and pronotum and elytra with a faint cupreous sheen. This is so in the habitus photograph as well as our experience with them in the field, under a bright LED headlamp. Other light sources such as the Wild M5 incandescent bulb source show no apparent metallic sheen to only a faint metallic sheen. The above description is what you will typically see through a microscope.

*Microsculpture*. Dorsum of head with microsculpture granulate, +/- isodiametric, easily visible at 50× magnification; pronotum somewhat granulate, transverse on disc, less so at margins; elytral intervals with shallowly impressed, transverse sculpticells; ventral surface of head, smooth with microsculpture not visible at 50×; prosternum, proepipleuron, mesepisternum and metepisternum with sculpticells forming a shallow, somewhat transverse to transverse mesh.

*Macrosculpture*. Dorsum of head somewhat rugulose between eyes, clypeus faintly rugulose to smooth, both head and clypeus with relatively dense, fine and scattered setigerous punctures, punctures not visible and setae hardly visible at 50×; pronotum with fine and scattered setigerous punctures, punctures not visible but setae visible in side view at 50×, disc faintly rugulose to smooth; elytra with intervals somewhat flat, +/- single row of fine scattered setigerous punctures in center of each interval, hardly visible in lateral view at 50×; striae narrow, appear impunctate when viewed dorsally but with single row of fine scattered setigerous punctures, hardly visible in lateral view at 50×; ventrally, thoracic and abdominal sclerites with scattered setigerous punctures throughout.

*Fixed setae.* Elytra with two setae in apical 1/3 stria 2, one seta near base in stria 3; ventrally, prosternal process with dense, circular patch of setae at base in males.

*Luster*. Dorsal surface of head and pronotum moderately shiny; elytra shiny.

*Head*. Mandibles somewhat curved at apex, somewhat long and narrow in form, when measured on outside diameter, visible portion approximately half the length of the labrum; labrum bilobed, right lobe occasionally slightly longer than left.

*Pronotum*. Anterior transverse impression shallow; posterior transverse impression moderately shallow; median longitudinal impression moderately shallow; lateral margins explanate, apico-lateral margins rounded, somewhat lobed, posterio-lateral margins sinuate, almost right angled.

*Elytra*. Apex almost truncate.

*Legs*. Two rows of small squamo-setae on tarsomeres 1–2 of mid-leg, males with one notch apically on ventral side of mid-tibia.

*Male genitalia*. Fig. 56A–D. Length 1.00 – 1.14 mm. Ostium catopic, positioned on left side of dorsal surface. Phallus cylindrical, right side straight along length, left side curving slightly from mid-length to apex of ostium when viewed ventrally, apical area somewhat elongate, apex bluntly rounded, slightly curved upwards in lateral view; endophallus, relatively short, stout and bulbous, distinctive form, sclerotized ring (esr) at narrowest portion of apical constriction, spines no evident.

*Female genitalia*. Fig. 58D. Width 0.96 mm. One spermatheca (sp1), cylindrical, ribbed laterally along length; one spermathecal accessory gland (sg), large and somewhat cylindrical, from apex of duct appears as two chambers, a small chamber followed by a constriction approximately the width of the gland duct, to a larger apical chamber; spermathecal gland duct (sgd) attachment site apically on large diverticulum (div) of spermatheca; bursa copulatrix (bc) with distinctive sac at apical end (bs), large and somewhat cylindrical, coming to a rounded point at the apex.

**Figure 57.**
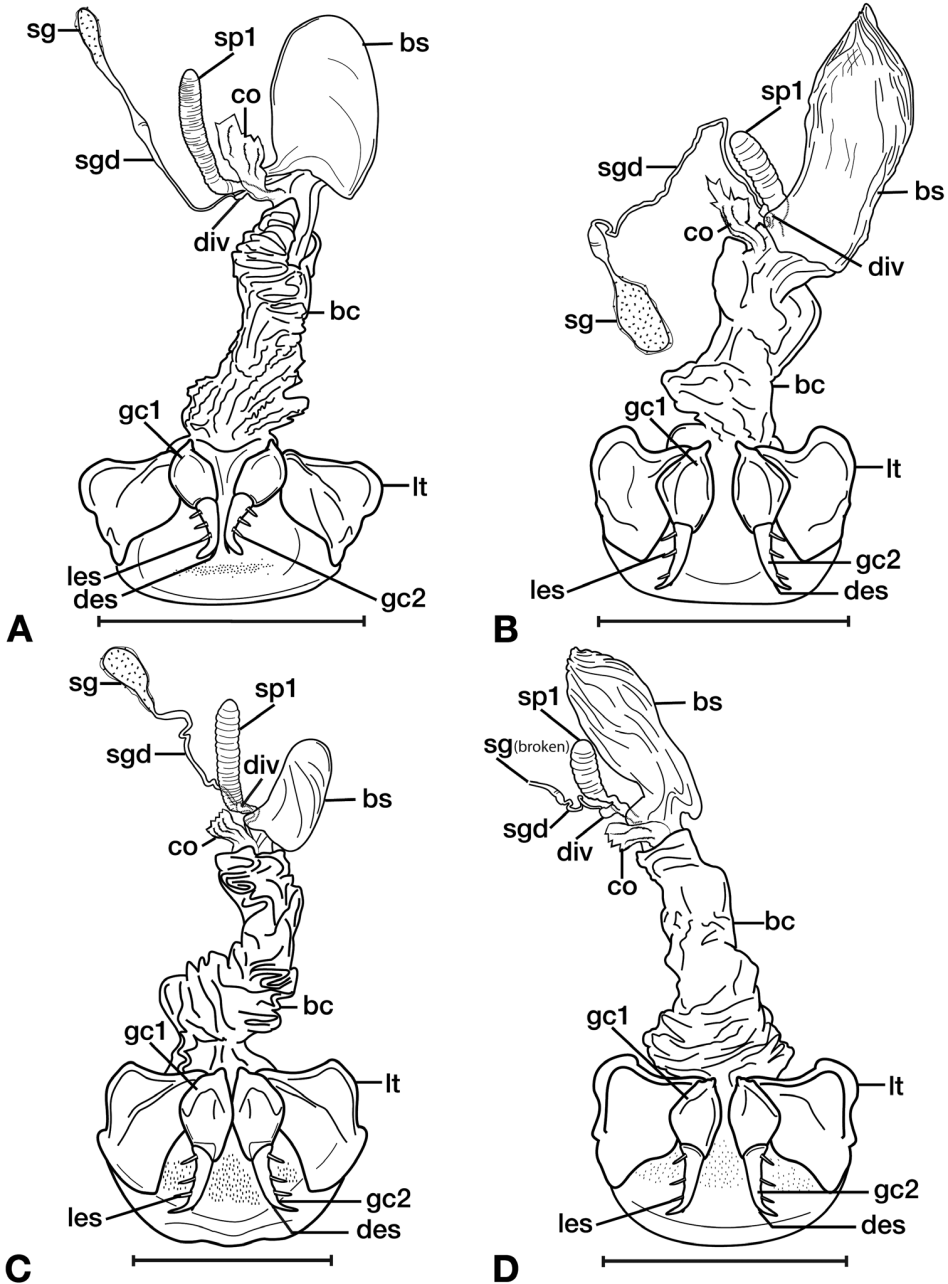
Line drawings of the female reproductive tract of species of the subgenus Coptoderina Jeannel, known from Taiwan (in part), ventral aspect. **A**C. (C.) chaudoiri Andrewes **B**C. (C.) eluta Andrewes **C**C. (C.) japonica Bates **D**C. (C.) maculata (Dupuis). Legend: **bc** bursa copulatrix; **bs** blind sac; **co** common oviduct; **des** dorsal ensiform setae; **div** diverticulum; **gc1** gonocoxite 1; **gc2** gonocoxite 2; **les** lateral ensiform setae; **lt** lateral tergite; **sg** spermathecal gland; **sgd** spermathecal gland duct; **sp1** spermatheca 1. Scale bars: 1 mm.

**Figure 58.**
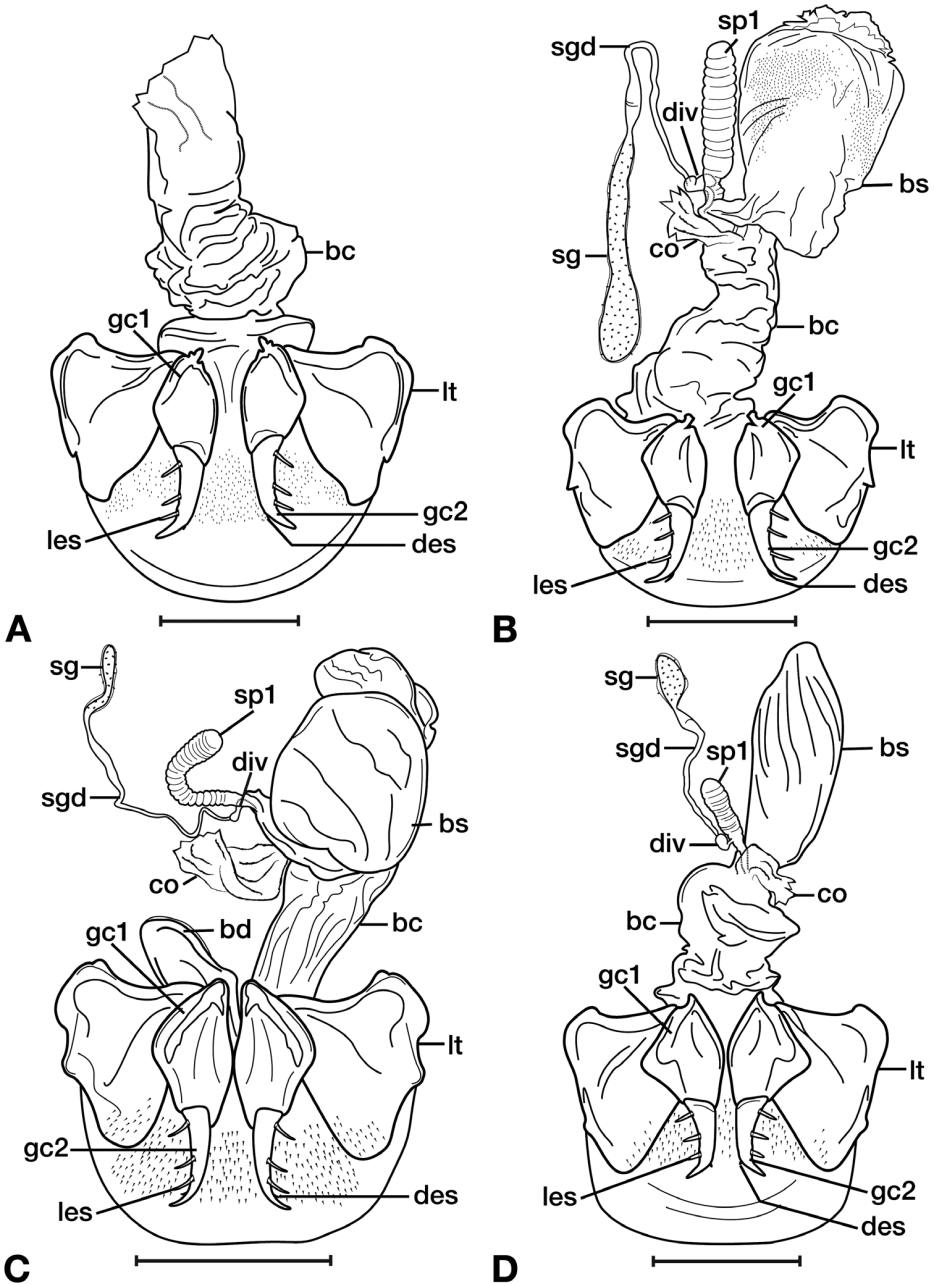
Line drawings of the female reproductive tract of species of the subgenus Coptoderina Jeannel, known from Taiwan (in part), ventral aspect. **A**C. (C.) marginata (Dupuis), badly damaged **B**C. (C.) occulta sp. n. **C**C. (C.) proksi Jedlička **D**C. (C.) taiwana (Nakane). Legend: **bc** bursa copulatrix; **bs** blind sac; **co** common oviduct; **des** dorsal ensiform setae; **div** diverticulum; **gc1** gonocoxite 1; **gc2** gonocoxite 2; **les** lateral ensiform setae; **lt** lateral tergite; **sg** spermathecal gland; **sgd** spermathecal gland duct; **sp1** spermatheca 1. Scale bars: 0.5 mm.

########### Habitat, habits, and seasonal occurrence.

The known elevational range of *C.taiwana* is from 100 to 1150 meters. Adults of this species are found in mixed forest of montane areas. This species is crepuscular and specimens can occasionally be found on trunks of live trees but is most commonly collected on deadwood. Specimens have been collected from March to December. Methods of collecting include u.v. light sheet, light trap, sweep netting, hand collecting, and insecticidal fogging of *Pinusmorrisonicola* Hayata.

########### Geographical distribution.

*Coptoderataiwana* is known from Japan and Taiwan. For Taiwan collecting localities see Figure 60.

**Figure 59.**
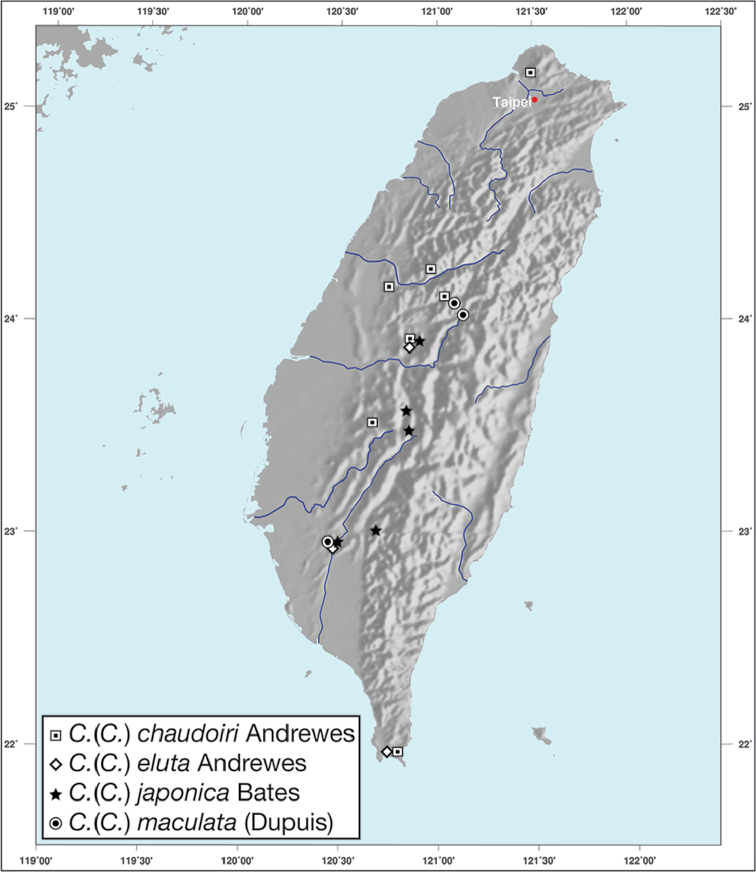
Map showing known localities for species of the genus CoptoderaDejean, subgenus Coptoderina Jeannel, in Taiwan (in part).

**Figure 60.**
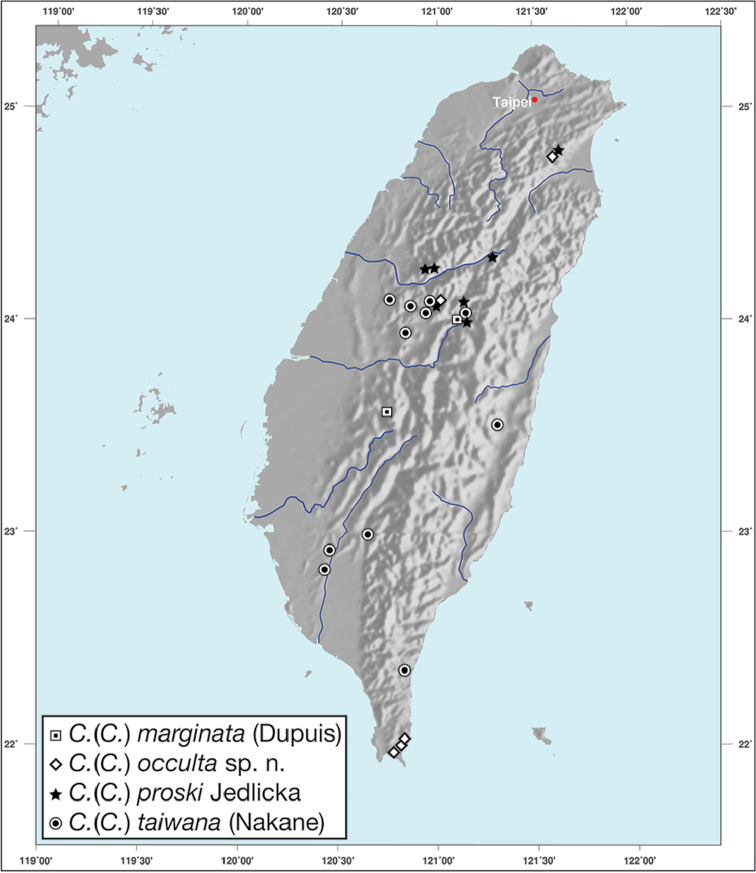
Map showing known localities for species of the genus CoptoderaDejean, subgenus Coptoderina Jeannel, in Taiwan (in part).

########## Genus Dolichoctis

Taxon classificationAnimaliaColeopteraCarabidae

Schmidt-Goebel

Dolichoctis Schmidt-Goebel, 1846: 62; Chaudoir 1869: 245; Csiki 1932: 1383; Jedlička 1963: 356; Habu 1967: 99; Darlington 1968: 15; Moore et al. 1987: 293; Lorenz 2005: 459; Baehr 1999: 124; Baehr 2006: 39; Baehr 2007: 79; Baehr 2013: 249; Baehr 2013b: 149.

########### Type species.

*Dolichoctisstriata* Schmidt-Goebel, 1846 (monotypic).

########### Type locality.

Burma.

########### Recognition of Taiwanese species of *Dolichoctis*

########### s. str.

*Color.* Various.

*Microsculpture*. Elytra with shallow, transverse sculpticells; ventral surface of head with microsculpture transverse, visible at 50×; prosternum, proepipleuron, mesepisternum and metepisternum with sculpticells forming a shallow transverse to almost isodiametric mesh.

*Macrosculpture and pilosity*. Dorsum of head smooth.

*Fixed setae.* Two pairs of supraorbital setae; clypeus with two lateral setae; labrum with six setae along apical margin; one pair of suborbital setae; pronotum with two pairs of setae, one at base of lateral margin and one on lateral margin at pronotum max width; 16 lateral (umbilical) setae in interval 9; ventral surface with fine, scattered setigerous punctures, two setae on each of abdominal sterna III to VI; two setae along apical margin of sternum VII in males, females with four setae near apical margin of sternum VII.

*Head*. Mandibles curved at apex; setose towards apex, visible when viewed laterally; mentum with no tooth; eyes convex; palpi cylindrical, elongate, setose.

*Hind wings*. Macropterous.

*Legs*. Tarsal claws denticulate, three to four denticles per claw; males with adhesive vestiture ventrally, two rows of squamo-setae at apex of tarsomeres 1–3 of fore-leg.

*Female genitalia*. Gonocoxite 2 (gc2) wide at base, narrowing and curving outwards from mid-length; two lateral ensiform setae (les) and one dorsal ensiform seta (des) present. Sensory furrow, furrow pegs and associated nematiform setae not observed.

######## Key to the Taiwanese species of the genus *Dolichoctis* Schmidt-Goebel

**Table:** 

1	Elytra with four testaceous maculae, contrasting strikingly with darker disc coloration	**2**
–	Elytra concolorous or if macula present, faint and hardly contrasting with elytral disc coloration	**3**
2	Anterior and posterior maculae near circular, smaller, anterior macula from interval 5 (sometimes into 4) to interval 8, posterior macula, extended from interval 3 to interval 5 or less (rarely from interval 2 to 6)	***Dolichoctistaiwanensis* Baehr**
–	Anterior macula sub-circular, slightly crescent shaped towards base, larger, anterior macula from interval 4 to interval 8, posterior macula, extended from interval 2 to interval 6 (sometimes 5)	***Dolichoctisrotundata* (Schmidt-Goebel)**
3	Pronotum with disc testaceous, contrasting strikingly with darker head and elytra; basal 1/4 of elytra diffuse	***Dolichoctisdilatata* sp. n.**
–	Pronotum only slightly lighter in coloration than head and elytra; four very faint macula, two anterior and two posterior, anterior maculae from interval 4 to interval 8 more or less ovoid, posterior maculae extended from suture to interval 5 or 6, forming single oval across center of disc	***Dolichoctisbadiadorsis* sp. n.**

######### Dolichoctis badiadorsis sp. n.

Taxon classificationAnimaliaColeopteraCarabidae

http://zoobank.org/7B61D836-D00C-470F-A666-94AC72AF4652

**Figure 61.**
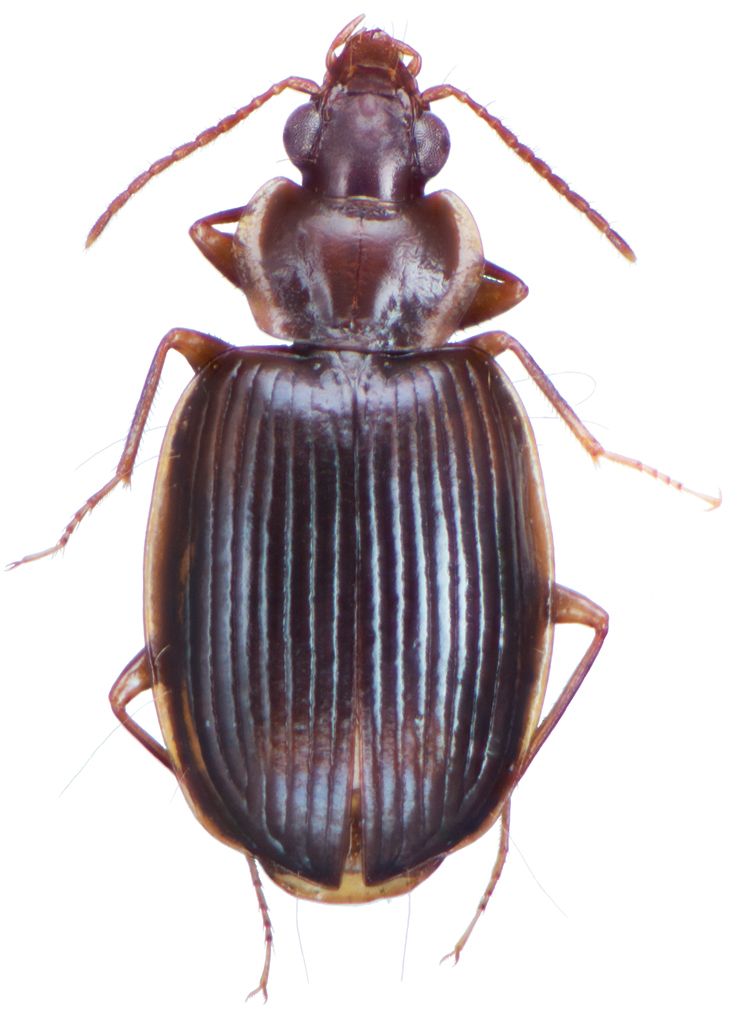
Dorsal habitus and color pattern of *Dolichoctisbadiadorsis* sp. n.. (OBL 5.22 mm).

**Figure 62.**
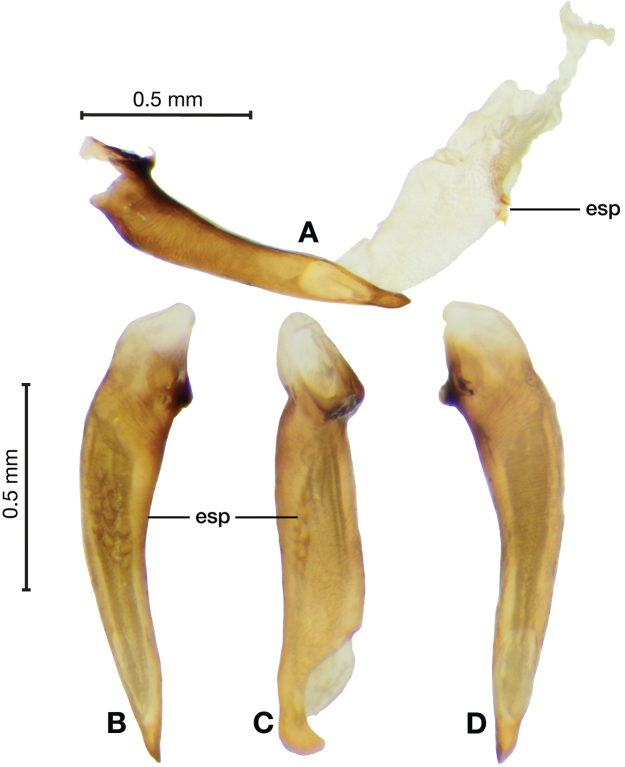
Digital images of male genitalia of *Dolichoctisbadiadorsis* sp. n.. **A** left lateral aspect, endophallus everted **B** right lateral aspect **C** ventral aspect **D** left lateral aspect. Legend: **esp** endophallic spine patch.

**Figure 63.**
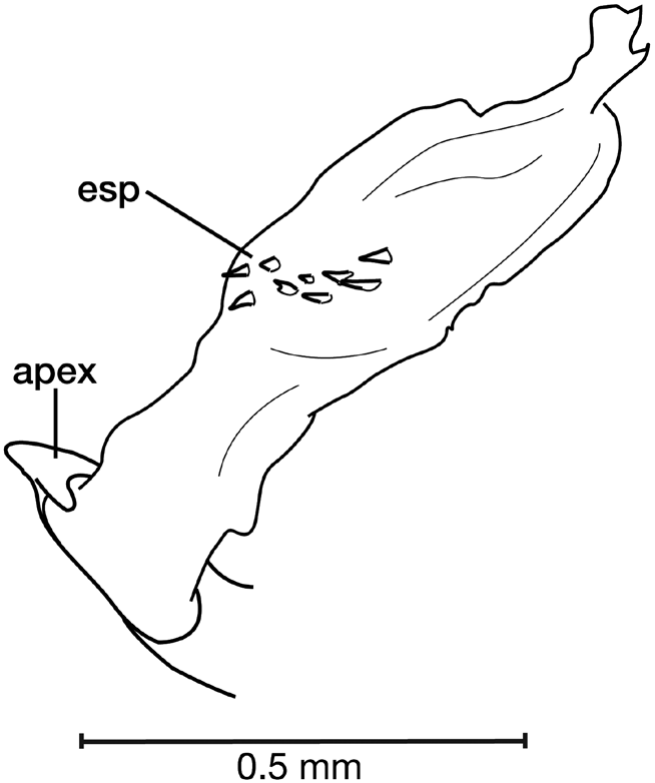
Line drawing of the phallus apex and endophallus, right lateral aspect, of *Dolichoctisbadiadorsis* sp. n.. Legend: **esp** endophallic spine patch; **apex** distinctively hooked phallus apex.

Figs 61 , 62A–D , 63 , 71A , 72

########## Etymology.

From Latin *badia* and *dorsis*, in reference to the relatively uniform, reddish brown coloration of the dorsal surface.

########## Types and other material examined.

**Holotype** (male) labeled: “Holotype” [circular, ringed with red]; “TAIWAN: Taichung Co./Dakeng Scenic Area/base of hiking trail 4/24.1737N, 120.7882E”; “veg. nr. lights on trail/night. Acc. Ti-85/May 25, 2011, 475m/Coll. W. M. Hunting”; “NCHU# 101165”. 64 **paratypes**: 25 males and 40 females. For further details see EH Strickland Virtual Entomology Museum Database.

########## Type locality.

Taiwan. Nantou county, Dakeng Scenic Area.

########## Taxonomic note.

*Dolichoctisbadiadorsis* appears to be closely related to *D.jacobsoni* Anderewes (1929), which is known from Vietnam, Sumatra, Java, and Borneo. It is easily distinguished from *D.jacobsoni* by the following differences: pronotum brunneo-testaceous to rufo-brunneous and with basal and apical width equal in length; elytra with faint apical macula extending to suture (2^nd^ to 5^th^ interval in *D.jacobsoni*); phallus with shaft uniformly narrowing towards apex in lateral view, apex in the form of a more spatulate hook; endophallus with spines of spine patch with different placement and number of spines.

########## Diagnosis.

Specimens of this species are easily distinguished from other species of *Dolichoctis* by a combination of the absence of elytra with only very faintly visible macula and a head and pronotum that is only somewhat lighter in coloration than head.

########## Description.

OBL 4.33 – 5.33 mm. Length (n = ten males, ten females): head 0.44 – 0.56, pronotum 0.72 – 0.96, elytra 2.58 – 3.33, metepisternum 0.56 – 0.76 mm; width: head 0.92 – 1.08, pronotum 1.36 – 1.64, elytra 2.08 – 2.50, metepisternum 0.36 – 0.48 mm.

*Body proportions*. HW/HL 1.85 – 2.18; PWM/PL 1.59 – 1.83; EL/EW 1.11 – 1.36; ML/MW 1.40 – 1.90.

*Color*. Fig. 61. Dorsum of head brunneous, clypeus and labrum brunneous, typically slightly lighter, antennae and palpi testaceous to brunneous; disc of pronotum brunneo-testaceous to rufo-brunneous, margins rufo-testaceous to brunneo-testaceous, always lighter than disc; elytral disc rufo-brunneous to brunneo-piceous, iridescent, most specimens with four very faint macula, slightly lighter than disc, two anterior and two posterior, anterior macula near humerus, from interval 4 to interval 8 more or less ovoid, posterior macula extended from suture to interval 5 or 6, forming single oval apically, across center of disc; lateral margins brunneo-testaceous to brunneous, somewhat translucent and slightly lighter than disc; ventral surface brunneo-testaceous to brunneous, metepisternum darker, apical margin of abdominal sterna darker; legs with trochanter and femora brunneo-testaceous, tibia with dorsal surface partially piceous.

*Microsculpture*. Dorsum of head with microsculpture granulate, almost isodiametric in front of eyes, somewhat transverse behind eyes, easily visible at 50× magnification; pronotum with shallow, transverse mesh pattern.

*Macrosculpture*. Elytra with intervals somewhat convex.

*Fixed setae.* Elytra with interval 3 with two punctures bearing fine setae, first near mid-length and second ~2/3 to apex.

*Luster*. Head capsule moderately dull; pronotum and elytra moderately glossy; ventral thoracic sterna and abdominal sterna moderately glossy.

*Head*. Labrum more or less rectangular, some specimens slightly emarginated.

*Pronotum*. Disc rather flat, anterior transverse impression shallow; posterior transverse impression shallow; median longitudinal impression moderately shallow; lateral margins explanate, apico-lateral margins rounded forming lobes, posterio-lateral margins typically broadly rounded, obtuse.

*Elytra*. Hind angles nearly truncate.

*Male genitalia*. Figs 62A–D, 63. Length 0.88 – 0.96 mm. Ostium left pleuropic. Phallus cylindrical at base but flattened dorso-ventrally in apical half, phallus apex with distinctive form, hooked to the left when viewed ventrally; endophallus straight and wide, with two rows of 5–7 moderately large spines (esp) from mid-length towards apex.

*Female genitalia*. Fig. 71A. Width 0.76 – 0.84 mm. One spermatheca (sp1), dome shaped, dome relatively small; spermathecal ring sclerite (srs) dividing spermatheca from spermathecal duct; one spermathecal accessory gland (sg) with spermathecal gland duct (sgd) attachment on lobe (srsl) coming off of right side of ring sclerite when viewed from ventral aspect.

########## Habitat, habits, and seasonal occurrence.

The known elevational range of *D.badiadorsis* is from 310 to 1400 meters with most being collected between 475 and 800 meters in elevation. Adults of this species are found in mixed forest of montane areas. Many specimens have been collected both from deadwood and trunks of live trees. Specimens have been collected from April to December in Taiwan with the majority being collected in May. Methods of collecting include u.v. light, m.v. light, sweep netting, hand collecting, and malaise trap.

########## Geographical distribution.

*Dolichoctisbadiadorsis* is known only from Taiwan. See Figure 72.

######### Dolichoctis dilatata sp. n.

Taxon classificationAnimaliaColeopteraCarabidae

http://zoobank.org/562B4C39-E6DB-4841-862F-BCAFDEBCED60

**Figure 64.**
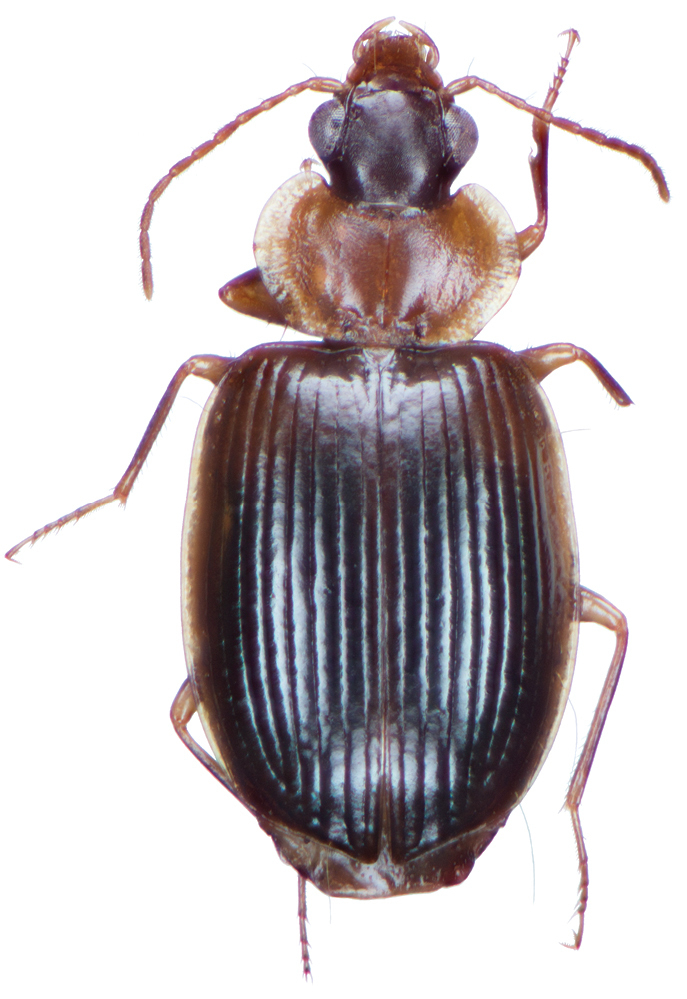
Dorsal habitus and color pattern of *Dolichoctisdilatata* sp. n.. (OBL 5.08 mm).

**Figure 65.**
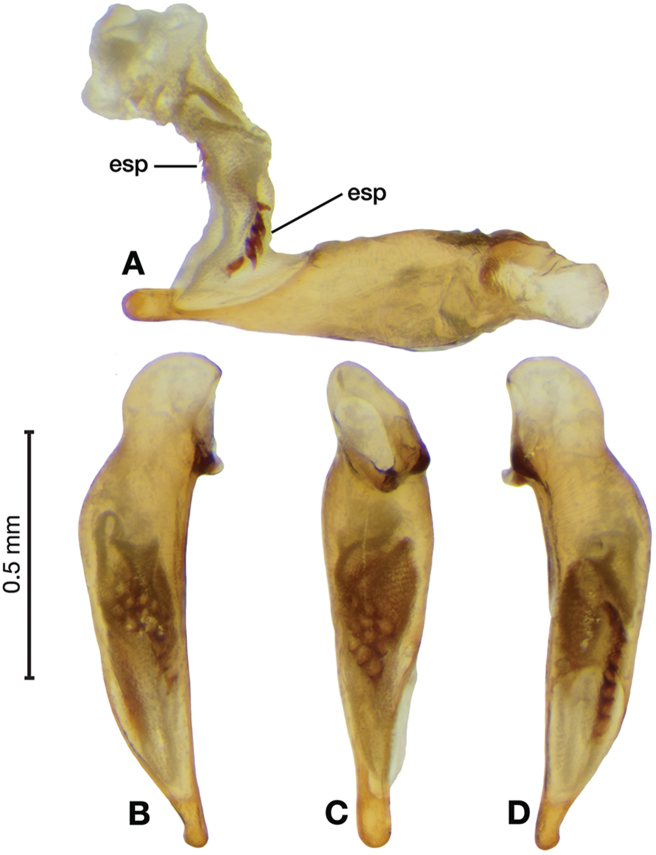
Digital images of male genitalia of *Dolichoctisdilatata* sp. n.. **A** ventral aspect, endophallus everted **B** right lateral aspect **C** ventral aspect **D** left lateral aspect. Legend: **esp** endophallic spine patch.

**Figure 66.**
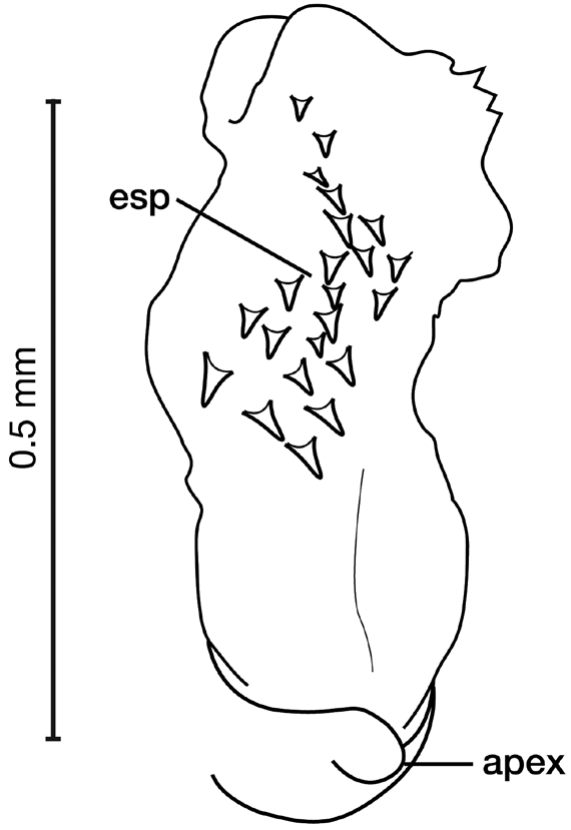
Line drawing of the phallus apex and endophallus, viewed laterally from phallus apex, of *Dolichoctisdilatata* sp. n.. Legend: **esp** endophallic spine patch; **apex** phallus apex.

Figs 64 , 65A–D , 66 , 71B , 72

########## Etymology.

From Latin *dilatata*, in reference to the distinctively wide and explanate pronotum.

########## Types and other material examined.

**Holotype** (male) labeled “Holotype” [circular, ringed with red]; “TAIWAN: Taichung Co./Dakeng Scenic Area/ base of hiking trail 4/ 24.1737N, 120.7882E”; “veg. nr. lights on trail/night. Acc. Ti-85/May 25, 2011, 475m/Coll. W. M. Hunting”; “NCHU# 101168”. Two **paratypes** of *D.dilatata*: one male and one female. For further details see EH Strickland Virtual Entomology Museum Database.

########## Type locality.

Taiwan. Dakeng Scenic Area, Nantou county.

########## Diagnosis.

Specimens of this species are easily distinguished from other species of *Dolichoctis* by a combination of the lack of elytral maculae and the striking contrast of testaceous pronotum with rest of dorsal surface.

########## Description.

OBL 4.92 – 5.08 mm. Length (n = two males, one female): head 0.48 – 0.50, pronotum 0.82 – 0.92, elytra 3.00, metepisternum 0.64 – 0.72 mm; width: head 1.04 – 1.08, pronotum 1.60 – 1.68, elytra 2.33, metepisternum 0.40 – 0.44 mm.

*Body proportions*. HW/HL 2.08 – 2.25; PWM/PL 1.78 – 1.95; EL/EW 1.29; ML/MW 1.45 – 1.70.

*Color*. Fig. 64. Dorsum of head rufo-brunneous, clypeus and labrum brunneo-testaceous, lighter that head, antennae and palpi testaceous to brunneous; disc of pronotum testaceous, strikingly lighter in coloration than head and elytra, margins translucent; elytral disc rufo-brunneous, hint of iridescence in basal 1/4, margins, suture, and apical area diffuse, rufous; ventral surface testaceous; legs with trochanter and femora testaceous to brunneo-testaceous, tibia with dorsal surface partially piceous.

*Microsculpture*. Dorsum of head with microsculpture granulate, isodiametric, sculpticells smallest on disc, between eyes, becoming gradually larger towards base, easily visible at 50× magnification; pronotum with transverse to somewhat transverse mesh pattern.

*Macrosculpture and pilosity*. Ventral surface of head distinctively rugulose; elytra with intervals somewhat convex, with several randomly scattered setigerous punctures, hardly visible at 50×; striae faintly punctate.

*Fixed setae.* Elytra with interval 3 with two punctures bearing fine setae, first near mid-length and second ~2/3 to apex.

*Luster*. Head capsule and pronotum moderately dull; elytra moderately glossy; ventral thoracic sterna and abdominal sterna moderately glossy.

*Head*. Mandibles almost entirely covered by pronotum; labrum more or less rectangular.

*Pronotum*. Anterior transverse impression shallow; posterior transverse impression shallow; median longitudinal impression moderately shallow; lateral margins widely explanate, apical margin deeply emarginate, forming distinctive apico-lateral lobes, posterio-lateral margins broadly rounded, obtuse.

*Elytra*. Hind angles slightly sinuate.

*Male genitalia*. Figs 65A–D, 66. Length 0.88 – 0.96 mm. Ostium left pleuropic. Phallus cylindrical, narrowing from mid-length to apex, apex somewhat elongate and slightly spatulate, rounded at apex and with slight constriction before apex, in ventral view; endophallus, short, slightly curved just above mid-length, with two large spine fields present, one row of vertical spines basally on side closest to phallus base, one larger, vertical spine field on apical side from below mid-length to endophallus apex.

*Female genitalia*. Fig. 71B. Width 0.76 mm. One spermatheca (sp1), elongate and domed at the apex; spermathecal ring sclerite (srs) dividing spermatheca from spermathecal duct; one spermathecal accessory gland (sg) with spermathecal gland duct (sgd) attachment site on relatively large lobe (srsl) coming off of right side of ring sclerite when viewed from ventral aspect.

########## Habitat, habits, and seasonal occurrence.

The known elevational range of *D.dilatata* is from 475 to 740 meters. This species in only known from three specimens. One specimen was collected during the day while sweeping vegetation along a walking trail. The other two were collected at night from live tree trunks. Adults of this species are found in mixed forest of montane areas. Specimens have been collected in May, July, and October in Taiwan. Methods of collecting include sweep netting and hand collecting.

########## Geographical distribution.

*Dolichoctisdilatata* is known only from Taiwan. See Figure 72.

######### Dolichoctis rotundata

Taxon classificationAnimaliaColeopteraCarabidae

(Schmidt-Goebel)

**Figure 67.**
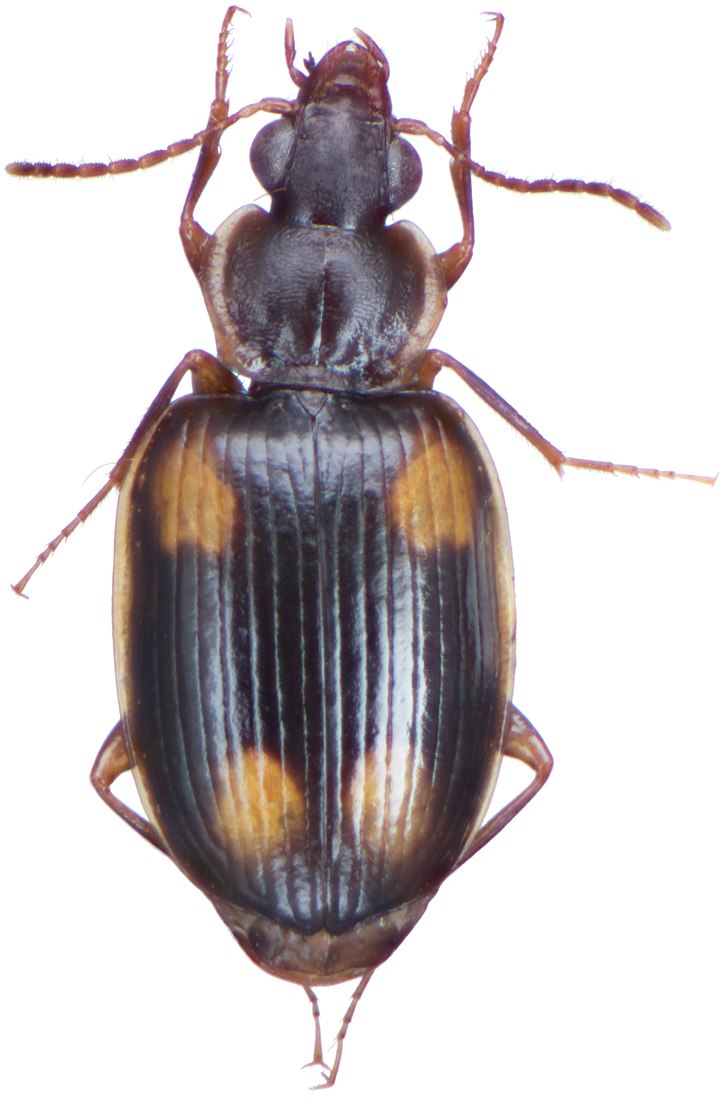
Dorsal habitus and color pattern of *Dolichoctisrotundata* (Schmidt- Goebel). (OBL 4.72 mm).

**Figure 68.**
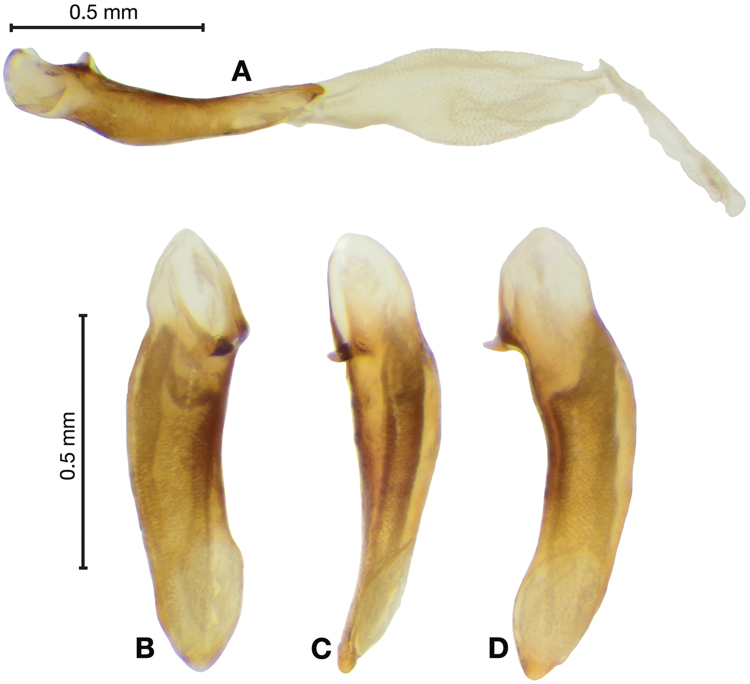
Digital images of male genitalia of *Dolichoctisrotundata* (Schmidt- Goebel). **A** right lateral aspect, endophallus everted **B** right lateral aspect **C** ventral aspect **D** left lateral aspect.

Figs 67 , 68A–D , 71C , 72

Mochtherus rotundatus Schmidt-Goebel, 1846: 77; Chaudoir 1869: 246; Bates 1892: 413; Andrewes 1923: 45; Jedlička 1963: 358; Habu 1967: 101.
Dolichoctis rotundata (Schmidt-Goebel); Jedlička, 1963: 358; Habu 1967: 102; Stork 1986: 15; Lorenz 2005: 459 (synonym of D.striata Schmidt-Goebel); Baehr 2013: 137.
Dolichoctis ornatella Bates, 1883: 282; Andrewes 1933: 347; Jedlička 1963: 357; Habu 1967: 101 (synonym of D.striata Schmidt-Goebel); Lorenz 2005: 459 (synonym of subspecies D.striatastriata Schmidt-Goebel); Baehr 2013: 138.
Dolichoctis kohpodaensis Kirschenhofer, 2012: 217.
Dolichoctis striatus formosanus Habu, 1967: 103. syn. n.
Dolchoctis striata formosana Habu: Lorenz, 2005: 45.

########## Types and other material examined.

84 specimens of *D.rotundata*: 44 males and 40 females. For further details see EH Strickland Virtual Entomology Museum Database.

########## Taxonomic notes.

In 2013, Baehr elevated *D.rotundata* back to species level and sited male genitalic features, among other things as his rationale for this. Examination of many specimens of this species, as well as other *Dolichoctis* from Taiwan, fully confirm his findings. Baehr also suspected that the subspecies formosana (Habu, 1967), which was described as a subspecies of *D.striata* at the time, was likely also *D.rotundata*. Examination of the types of this subspecies confirms this synonomy with confidence.

########## Type locality.

“Tenasserim” Myanmar.

########## Diagnosis.

Specimens of this species are distinguished from other species of *Dolichoctis* in Taiwan by the large and often cresent-shaped anterior maculae.

########## Redescription.

OBL 4.30 – 5.20 mm. Length (n = ten males, ten females): head 0.48 – 0.60, pronotum 0.76 – 0.98, elytra 2.83 – 3.50, metepisternum 0.64 – 0.76 mm; width: head 0.96 – 1.08, pronotum 1.28 – 1.56, elytra 2.08 – 2.50, metepisternum 0.40 – 0.48 mm.

*Body proportions*. HW/HL 1.73 – 2.08; PWM/PL 1.50 – 1.89; EL/EW 1.25 – 1.43; ML/MW 1.45 – 1.80.

*Color*. Fig. 67. Dorsum of head brunneous to rufo-piceous, clypeus and labrum brunneous to rufo-brunneous, somewhat darker centrally, antennae and palpi brunneous; disc of pronotum brunneous to rufo-piceous, margins brunneo-testaceous, always lighter than disc; elytral disc rufo-piceous to piceous, with four testaceous macula, two anterior and two posterior, anterior macula near humerus, from interval 4 to interval 8, closest to apex in interval 5 (sometimes 4), sub-circular, slighty cresent shaped at base of macula, posterior maculae extended from interval 2 to interval 6 (sometimes 5), more or less circular; lateral margins testaceous to brunneous, somewhat translucent; ventral surface brunneo-testaceous to brunneous, metepisternum darker, apical margin of abdominal sterna rufo-brunneous to piceous; legs with trochanter and femora brunneo-testaceous to brunneous, tibia with dorsal surface partially piceous.

*Microsculpture*. Dorsum of head with granulate microsculpture, isodiametric, easily visible at 50× magnification; pronotum with transverse mesh pattern, ~2× longer than wide.

*Macrosculpture and pilosity*. Pronotum shallowly rugulose near base; elytra with intervals somewhat flat, interval 3 with two punctures visible, no setae apparent, first near mid-length and second in apical macula, occasionally other punctures apparent but not as distinctive as the two in interval 3.

*Luster*. Head capsule moderately glossy; pronotum and elytra glossy; ventral thoracic sterna and abdominal sterna moderately glossy.

*Head*. Mandibles relatively short, mostly covered by labrum; labrum more or less rectangular, rounded at apex.

*Pronotum*. Anterior transverse impression very shallow; posterior transverse impression moderately shallow, median longitudinal impression moderately shallow; lateral margins explanate, apico-lateral margins rounded forming distinctive lobes, posterio-lateral margins slightly sinuate from behind lateral fixed setae, obtuse.

*Elytra*. Lateral margins slightly explanate, hind angles nearly truncate.

*Male genitalia*. Fig. 68A–D. Length 0.72 – 0.82 mm. Ostium left pleuropic. Phallus cylindrical but distinctly flattened dorso-ventrally in apical half, phallus apex with distinctive form in right lateral view, apical area short, endophallus straight and somewhat expanded at mid-length.

*Female genitalia*. Fig. 71C. Width 0.70 – 0.72 mm. One spermatheca (sp1), dome shaped; distinctive ring sclerite (srs) dividing spermatheca from spermathecal duct; one spermathecal accessory gland (sg) with spermathecal gland duct (sgd) attachment site coming off of distinctly long and narrow lobe (srsl) on left side of ring sclerite when viewed from ventral aspect.

########## Habitat, habits, and seasonal occurrence.

The known elevational range of *D.rotundata* is from 61 to 725 meters. Adults of this species are found in mixed forest of montane areas. Many specimens of this species were collected both from deadwood and trunks of live trees. Specimens have been collected from March to December in Taiwan. Methods of collecting include u.v. light, sweep netting, hand collecting, malaise trap, flight intercept trap, and insecticidal fogging the canopy of tree species *Ficusirisana* Elmer, at night.

########## Geographical distribution.

*Dolichoctisrotundata* is widespread in Asia. It is known from India, Myanmar, Thailand, Malaysia, Vietnam, China, Japan, Sumatra, Java, Bali, Borneo, and Taiwan. For Taiwan collecting localities see Figure 72.

######### Dolichoctis taiwanensis

Taxon classificationAnimaliaColeopteraCarabidae

Baehr

**Figure 69.**
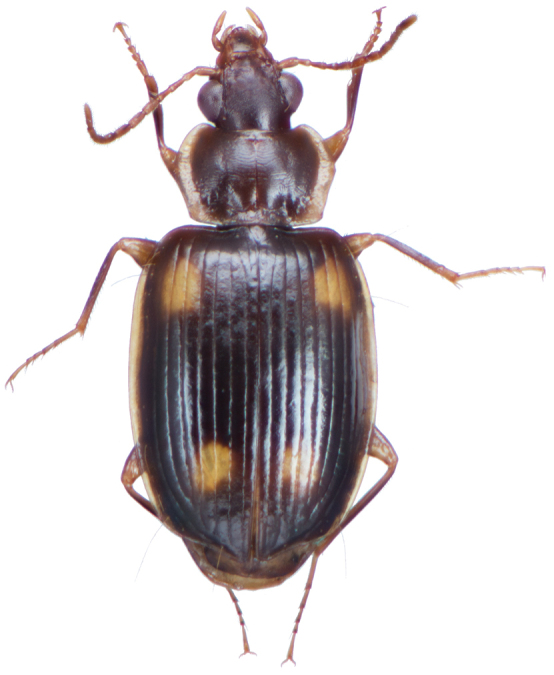
Dorsal habitus and color pattern of *Dolichoctistaiwanensis* Baehr. (OBL 5.22 mm).

**Figure 70.**
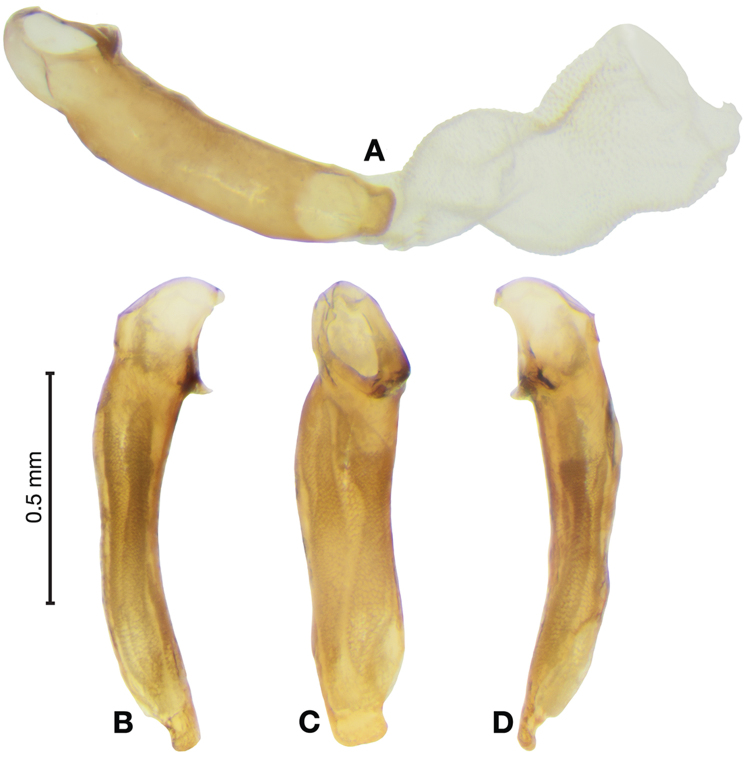
Digital images of male genitalia of *Dolichoctistaiwanensis* Baehr. **A** ventral aspect, endophallus everted **B** right lateral aspect **C** ventral aspect **D** left lateral aspect.

**Figure 71.**
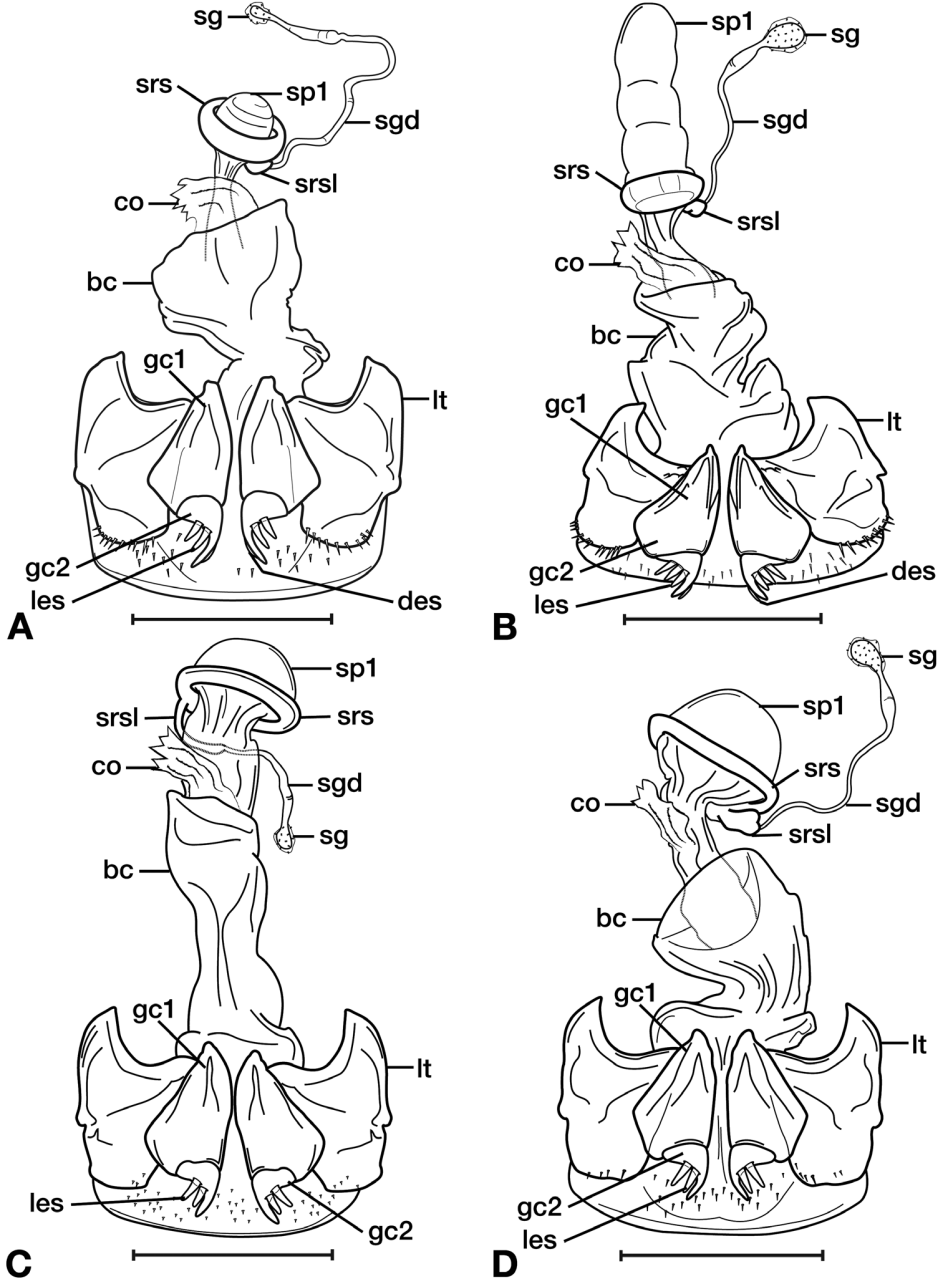
Line drawings of the female reproductive tract of species of the genus *Dolichoctis* (Schmidt-Goebel) known from Taiwan, ventral aspect. **A***D.badiadorsis* sp. n. **B***D.dilatata* sp. n. **C***D.rotundata* (Schmidt-Goebel) **D***D.taiwanensis* Baehr. Legend: **bc** bursa copulatrix; **co** common oviduct; **des** dorsal ensiform setae **gc1** gonocoxite 1; **gc2** gonocoxite 2; **les** lateral ensiform setae; **lt** lateral tergite; **sg** spermathecal gland; **sgd** spermathecal gland duct; **sp1** spermatheca 1; **srs** spermathecal ring sclerite; **srsl** spermathecal ring sclerite lobe. Scale bars: 0.5 mm.

**Figure 72.**
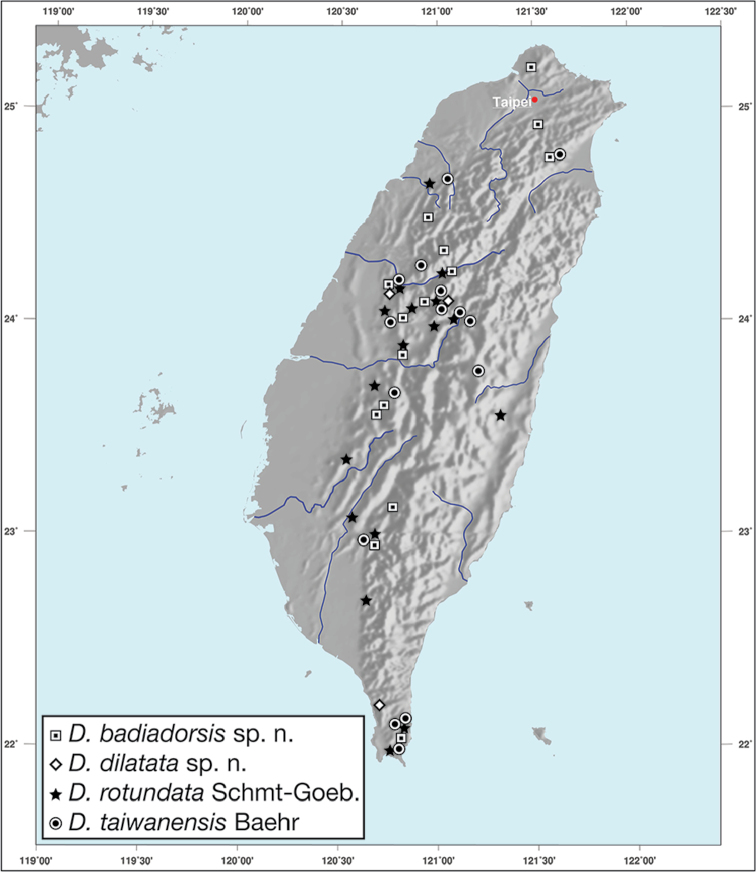
Map showing known localities for species of the genus *Dolichoctis* Schmidt-Goebel, in Taiwan.

Figs 69 , 70A–D , 71D , 72

Dolichoctis taiwanensis Baehr, 2013: 152.

########## Types and other material examined.

86 specimens: 49 males and 37 females. For further details see EH Strickland Virtual Entomology Museum Database.

########## Type locality.

Taiwan. “Mt. Ari” = Alishan, Chiayi County, “Karapin” = Chaoliping.

########## Diagnosis.

Specimens of this species are distinguished from other species of *Dolichoctis* by elytra with small and circular maculae with anterior macula typically from only extending from interval 5 to 8.

########## Redescription.

OBL 4.50 – 5.67 mm. Length (n = ten males, ten females): head 0.48 – 0.56, pronotum 0.80 – 1.04, elytra 2.67 – 3.50, metepisternum 0.60 – 0.80 mm; width: head 0.88 – 1.10, pronotum 1.32 – 1.76, elytra 2.00 – 2.50, metepisternum 0.36 – 0.48 mm.

*Body proportions*. HW/HL 1.83 – 2.00; PWM/PL 1.62 – 1.80; EL/EW 1.29 – 1.43; ML/MW 1.64 – 1.90.

*Color*. Fig. 69. Dorsum of head brunneous to rufo-brunneous, dark, clypeus and labrum brunneous to rufo-brunneous, somewhat darker centrally, antennae and palpi brunneous; disc of pronotum brunneous to rufo-piceous, margins brunneo-testaceous, always lighter than disc; elytral disc rufo-piceous to piceous, with four testaceous maculae, two anterior and two posterior, anterior maculae near humerus, from interval 5 (sometimes into 4) to interval 8, closest to apex in interval 5 (sometimes 4), more or less circular, posterior maculae, extended from interval 3 to interval 5 or less, rarely from interval 2 to 6, circular; lateral margins testaceous to brunneous, somewhat translucent; ventral surface brunneo-testaceous to brunneous, metepisternum darker, apical edge of abdominal sterna darker; legs with trochanter and femora brunneo-testaceous to brunneous, tibia with dorsal surface partially piceous.

*Microsculpture*. Dorsum of head with granulate microsculpture, isodiametric, easily visible at 50× magnification; pronotum with transverse mesh pattern, ~2× longer than wide.

*Macrosculpture*. Pronotum shallowly rugulose near base; elytra with intervals somewhat flat, interval 3 with two punctures visible, no setae apparent, first near mid-length and second in apical macula, occasionally other punctures apparent but not as distinctive as the two in interval 3.

*Luster*. Head capsule moderately glossy; pronotum and elytra glossy; ventral thoracic sterna and abdominal sterna moderately glossy.

*Head*. Mandibles relatively short, mostly covered by labrum; labrum more or less rectangular, rounded at apex.

*Pronotum*. Anterior transverse impression very shallow; posterior transverse impression moderately shallow; median longitudinal impression moderately shallow; lateral margins explanate, apico-lateral margins rounded forming distinctive lobes, posterio-lateral margins typically broadly rounded, obtuse.

*Elytra*. lateral margins slightly explanate, hind angles nearly truncate.

*Male genitalia*. Fig. 70A–D. Length 0.88 – 0.96 mm. Ostium catopic. Phallus cylindrical but distinctly flattened dorso-ventrally in apical half, phallus apex with distinctive form, more or less rectangular with rounded edges, curving ventrally at apex, endophallus straight and expanded apically.

*Female genitalia*. Fig. 71D. Width 0.76 – 0.84 mm. One spermatheca (sp1), dome shaped; distinctive spermathecal ring sclerite (srs) dividing spermatheca from spermathecal duct; one spermathecal accessory gland (sg) with spermathecal gland duct (sgd) attachment site on relatively large lobe (srsl) coming off of right side of ring sclerite when viewed from ventral aspect.

########## Habitat, habits, and seasonal occurrence.

The known elevational range of *D.taiwanensis* is from 170 to 2069 meters. Only two specimens have been collected at over 1000 meters, with the most being collected between 600 and 800 meters in elevation. Adults are found in mixed forest of montane areas. Many specimens of this species have been collected both from deadwood and trunks of live trees. Specimens have been collected from April to December. Methods of collecting include u.v. light, sweep netting, sugar baiting, canopy bagging, hand collecting, malaise trap, flight intercept trap, sticky trap, and insecticidal fogging the canopy of tree species *Ficusirisana* Elmer, *Machiluszuhoensis* Hayata, and *Pinusmorrisonicola* Hayata, at night.

########## Geographical distribution.

*Dolichoctistaiwanensis* is known only from Taiwan. See Figure 72.

######### Genus Formosiella

Taxon classificationAnimaliaColeopteraCarabidae

Jedlička

Formosiella Jedlička, 1951: 112; Kirschenhofer 1994: 1027; Lorenz 2005: 460.

########## Type species.

*Formosiellabrunnea* Jedlička, 1951 (monobasic).

######### Pseudomenarus

Taxon classificationAnimaliaColeopteraCarabidae

Shibata, 1964: 44.

########## Type species.

*Pseudomenarusflavomaculatus* Shibata (monotypic); Lorenz 2005: 463. comb. n.

########## Type locality.

Taiwan. Arisan = Alishan, Chiayi County.

########## Recognition of Taiwanese species of *Formosiella*

########## .

*Color.* Various.

*Microsculpture*. Labrum with microsculpture almost isodiametric to isodiametric.

*Fixed setae.* Two pairs of supraorbital setae; clypeus with two long, lateral setae; labrum with six setae along apical margin; one pair of suborbital setae; ventral surface with two setae on each of abdominal sterna III to VI; four setae along apical margin of sternum VII in females, two setae along apical margin of sternum VII in males.

*Abdomen.* Sternum VII of males distinctly bilobed.

*Hind wings*. Macropterous.

*Legs*. Tarsal claws denticulate, 3 – 4 denticles per claw, tarsomere 4 not bilobed; males with one notch apically on ventral side of mid-tibia.

*Male genitalia*. Ostium left pleuropic. Phallus cylindrical.

########## Taxonomic notes.

Upon close examination of the monobasic genus *Pseudomenarus* (Shibata, 1964), it became apparent that there were several diagnostic similarities with members of the genus *Formosiella* Jedlička, 1951. For the genus *Formosiella*, Jedlička cited defining characteristics as being: tarsomere 4 not bilobed; pronotum wider than head and strongly indented; elytra with three fixed seta in interval 3 and a dorsal surface that is finely and densely ciliated. Shibata’s *Pseudomenarusflavomaculatus* shares all of these characteristics and externally, also shares a toothed mentum and distinctively acuminate apices of both labial and maxillary palpi (Fig. 50B). Internally, the female genital tract is also very similar to *Formosiellabrunnea* (See Fig. 77A, B) and distinctive from all other pericaline genera. With this in mind, *Pseudomenarus* is synonymized with the generic name *Formosiella*.

######## Key to the Taiwanese species of the genus *Formosiella* Jedlička

**Table:** 

1	Disc of elytra concolorous, rufo-brunneous to brunneous	***F.brunnea* Jedlička**
–	Disc of elytra with four diffuse, testaceous maculae (appearing as six or eight smaller macula when apical macula is reduced and broken) anterior macula crescent shaped, crescent open towards elytra base	***F.flavomaculata* (Shibata), comb. n.**

######### Formosiella brunnea

Taxon classificationAnimaliaColeopteraCarabidae

Jedlička

**Figure 73.**
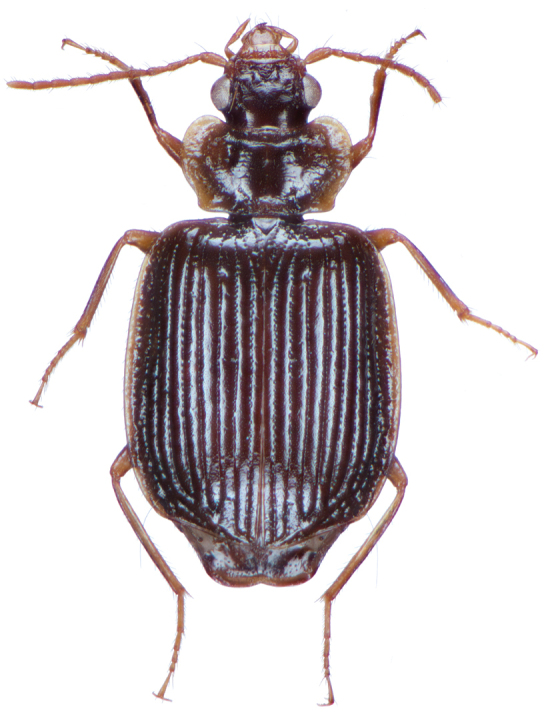
Dorsal habitus and color pattern of *Formosiellabrunnea* Jedlička. (OBL 5.50 mm).

**Figure 74.**
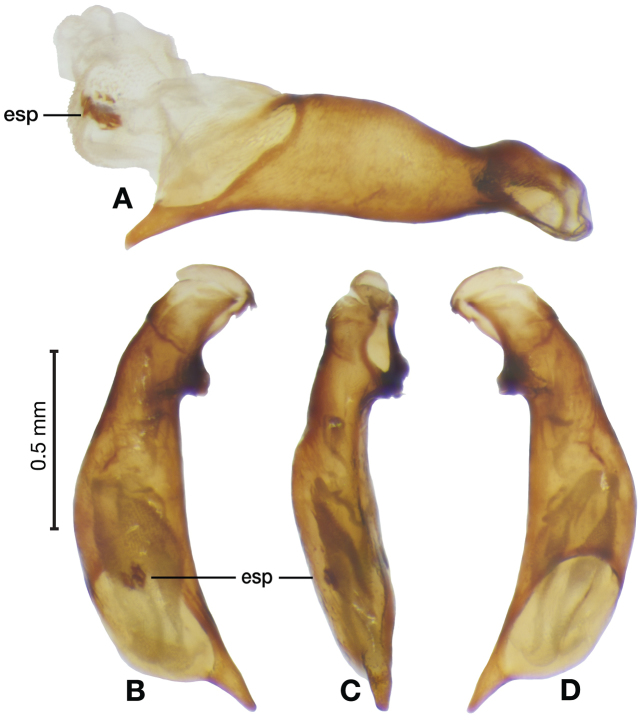
Digital images of male genitalia of *Formosiellabrunnea* Jedlička. **A** left lateral aspect, endophallus everted **B** right lateral aspect **C** ventral aspect **D** left lateral aspect. Legend: **esp** endophallic spine patch.

Figs 73 , 74A–D , 77A , 78

Formosiella brunnea Jedlička, 1951: 113; Kirschenhofer 1994: 1029; Lorenz 2005; 460.

########## Types and other material examined.

**Holotype** (male) labeled “ARISAN/FORMOSA/22.x. 1931/COL. M. CHUJO”; “TYPUS” [rectangular, red paper, black border]; Formosiella/brunnea sp. n./ DET.ING.JEDLICKA”. 51 specimens of *F.brunnea*: 26 males and 25 females. For further details see EH Strickland Virtual Entomology Museum Database.

########## Type locality.

Taiwan. “Arisan” = Alishan, Chiayi County.

########## Diagnosis.

Specimens of this species are easily distinguished from the only other Taiwanese species of the genus by the concolorous elytral disc.

########## Redescription.

OBL 5.42 – 6.50 mm. Length (n = ten males, ten females): head 0.52 – 0.64, pronotum 0.92 – 1.12, elytra 3.50 – 4.16, metepisternum 0.80 – 1.00 mm; width: head 1.12 – 1.32, pronotum 1.68 – 2.04, elytra 2.75 – 3.50, metepisternum 0.48 – 0.64 mm.

*Body proportions*. HW/HL 1.97 – 2.38; PWM/PL 1.65 – 2.00; EL/EW 1.17 – 1.26; ML/MW 1.54 – 1.85.

*Color*. Fig. 73. Dorsum of head rufo-brunneous to rufo-piceous, dark, clypeus and labrum brunneo-testaceous to testaceous, much lighter that head, antennae and palpi brunneo-testaceous to testaceous; disc of pronotum rufo-brunneous to rufo-piceous, always slightly lighter than head, margins translucent, rufo-brunneous; elytral disc rufo-brunneous to rufous, margins and suture slightly lighter; ventral surface brunneous to rufo-brunneous, darker at margins; metepisternum slightly darker; legs with trochanter and femora rufo-testaceous, tibia with surface partially rufo-piceous to piceous.

*Microsculpture*. Remaining dorsal surface with microsculpture not visible at 50×; ventral surface of pronotum and lateral margins of abdominal segments with faint transverse microsculpture; metepisternum with contrasting granulate and easily visible isodiametric microsculpture. Remaining ventral surface with microsculpture not visible or hardly so at 50×.

*Macrosculpture and pilosity*. Dorsum of head and clypeus with fine, scattered setigerous punctures, punctures easily visible but setae hardly so at 50×; pronotum with fine, scattered setigerous punctures, setae of two lengths, longer setae more sparsely dispersed over surface, margin with evenly spaced setae along border; elytra with intervals somewhat convex, entire surface with fine, scattered setigerous punctures with setae hardly visible at 50×, intervals 1, 3, 5, 7, 8, 9 with rows of longer setae with larger punctures, +/- evenly spaced along length of interval apex, striae punctate, margin with evenly spaced setae along border; ventral surface with several randomly scattered setigerous punctures.

*Fixed setae.* Pronotum with two pairs of setae, one at base of lateral margin and one on lateral margin at pronotum max width; 16–17 lateral (umbilical) setae in interval 9; elytra with interval 3 with three punctures not bearing setae, first on outside of interval in basal 1/6, second on inside of interval at mid-length, third on inside of interval in apical 1/6.

*Luster*. Dorsal surface glossy; ventral surface moderately glossy to glossy.

*Head*. Mandibles curved at apex; labrum long, bilobed, setose towards apex, setae visible when viewed laterally; mentum with tooth; eyes moderately convex; palpi cylindrical, elongate and setose.

*Pronotum*. Anterior transverse impression shallow; posterior transverse impression shallow, median longitudinal impression shallow; lateral margins widely explanate, apical margin deeply emarginate, forming broad apico-lateral lobes, posterio-lateral margins slightly sinuate, obtuse.

*Elytra*. Elytra relatively short and wide, hind angles truncate.

*Legs*. Males with adhesive vestiture ventrally, two rows squamo-setae on tarsomeres 1–3 of fore-leg.

*Male genitalia*. Fig. 74A–D. Length 1.08 – 1.20 mm. Phallus narrowest at base and widening towards apex in lateral view, apex narrow and pointed; endophallus short, with one microtrichial field (esp) present near mid-length on left side.

*Female genitalia*. Fig. 77A. Width 0.96 – 1.00 mm. Gonocoxite 2 (gc2) relatively narrow along length; two lateral ensiform setae (les), one dorsal ensiform seta. Two spermathecae present, first spermatheca (sp1) with attachment site near junction of bursa copulatrix (bc) and common oviduct (co), cylindrical and elongate; second spermatheca (sp2) with attachment site in basal 1/3 of spermatheca 1, cylindrical and slightly expanded towards apex; one spermathecal accessory gland (sg), round, associated spermathecal gland duct (sgd) somewhat expanded before gland, attachment site at base of spermatheca 2.

########## Habitat, habits, and seasonal occurrence.

The known elevational range of *F.brunnea* is from 700 to 2230 meters, with the majority being collected from between 1830 and 2000 meters. Most specimens collected have come from trees that have recently fallen or been uprooted and are dying. Adults are nocturnal or crepuscular and are found in mixed forest of both primary and disturbed secondary forests. Specimens have been collected from March to September. Methods of collecting include u.v. light sheet, m.v. light, sugar baiting tree trunks and hand collecting.

########## Geographical distribution.

*Formosiellabrunnea* is known only from Taiwan. See Figure 78.

######### Formosiella flavomaculata

Taxon classificationAnimaliaColeopteraCarabidae

(Shibata) comb. n.

**Figure 75.**
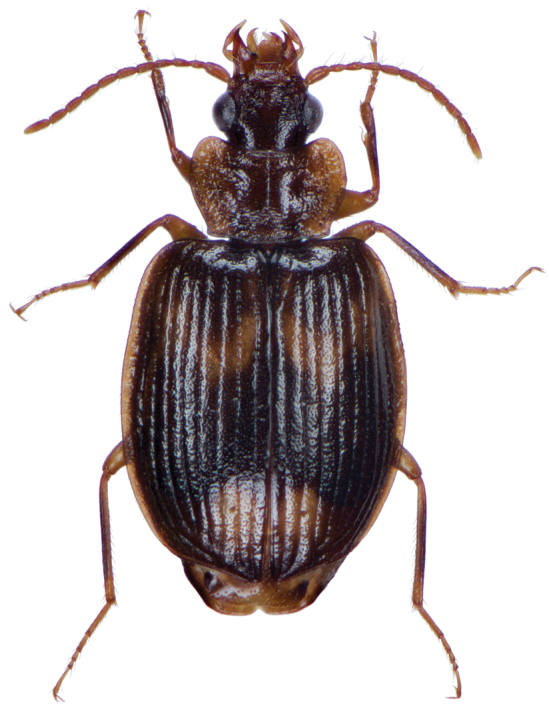
Dorsal habitus and color pattern of *Formosiellaflavomaculata* (Shibata). (OBL 5.12 mm).

**Figure 76.**
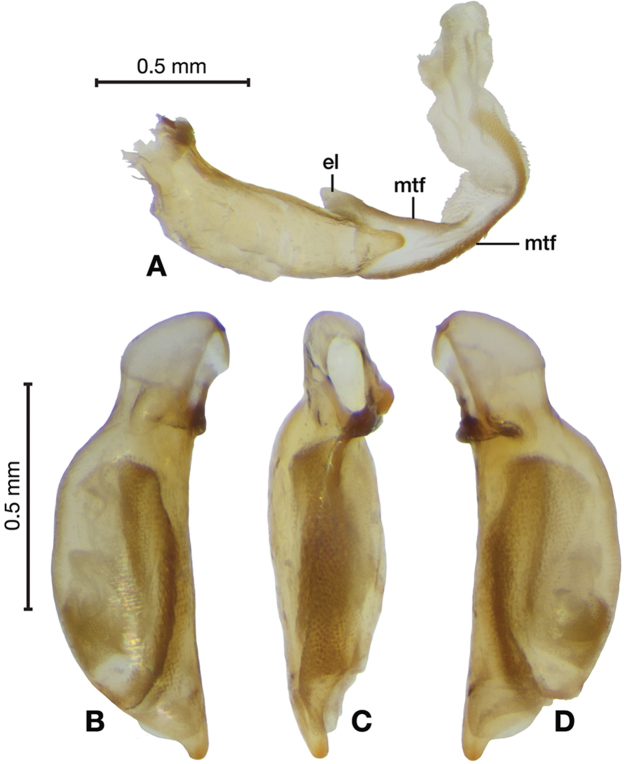
Digital images of male genitalia of *Formosiellaflavomaculata* (Shibata). **A** dorsal aspect, endophallus everted **B** right lateral aspect **C** ventral aspect **D** left lateral aspect. Legend: **el** endophallic lobe; **mtf** microtrichial field.

**Figure 77.**
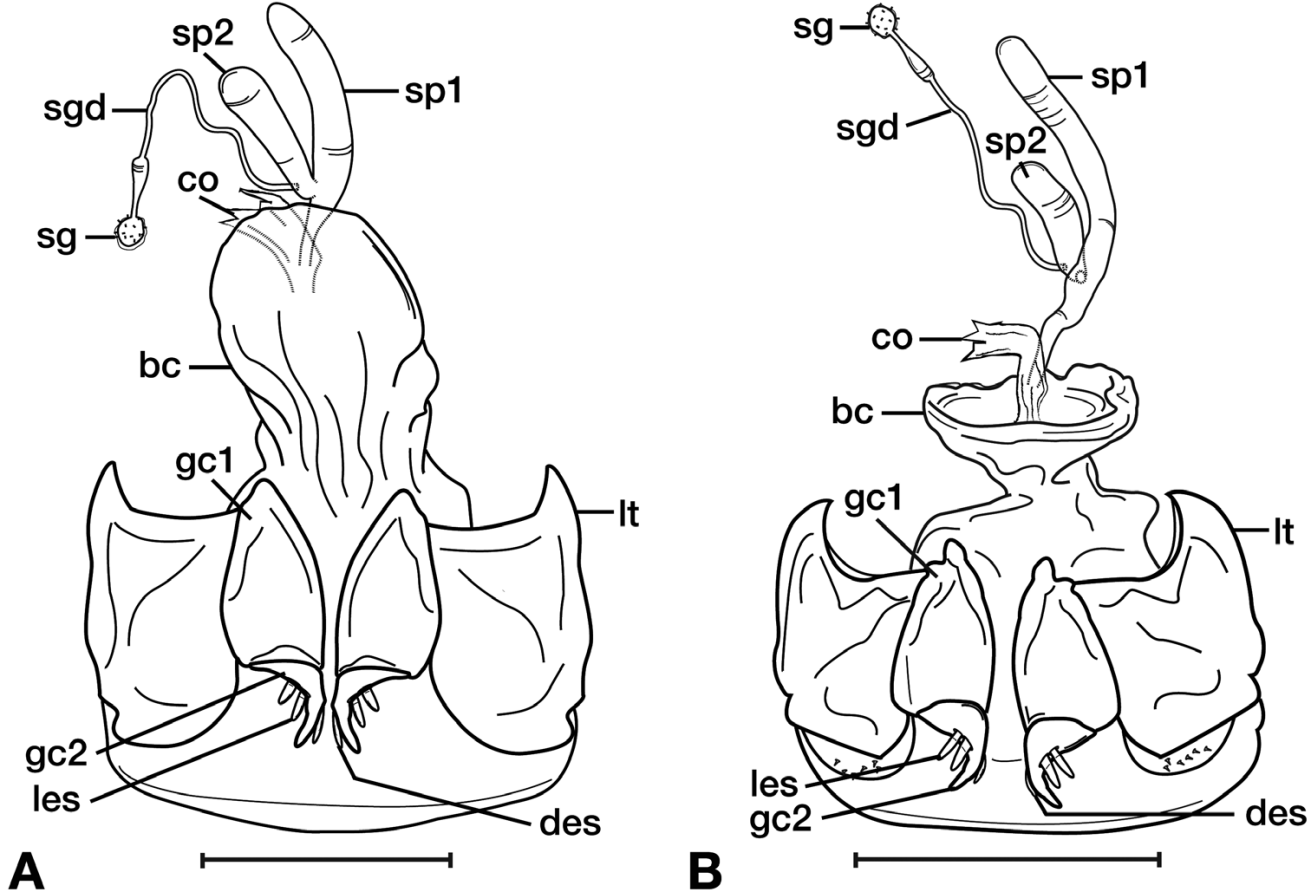
Line drawings of the female reproductive tract of species of the genus *Formosiella* Jedlička, known from Taiwan, ventral aspect. **A***F.brunnea* Jedlička **B***F.flavomaculata* (Shibata). Legend: **bc** bursa copulatrix; **co** common oviduct; **des** dorsal ensiform setae; **gc1** gonocoxite 1; **gc2** gonocoxite 2; **les** lateral ensiform setae; **lt** lateral tergite; **sg** spermathecal gland; **sgd** spermathecal gland duct; **sp1** spermatheca 1; **sp2** spermatheca 2. Scale bars: 0.5 mm.

**Figure 78.**
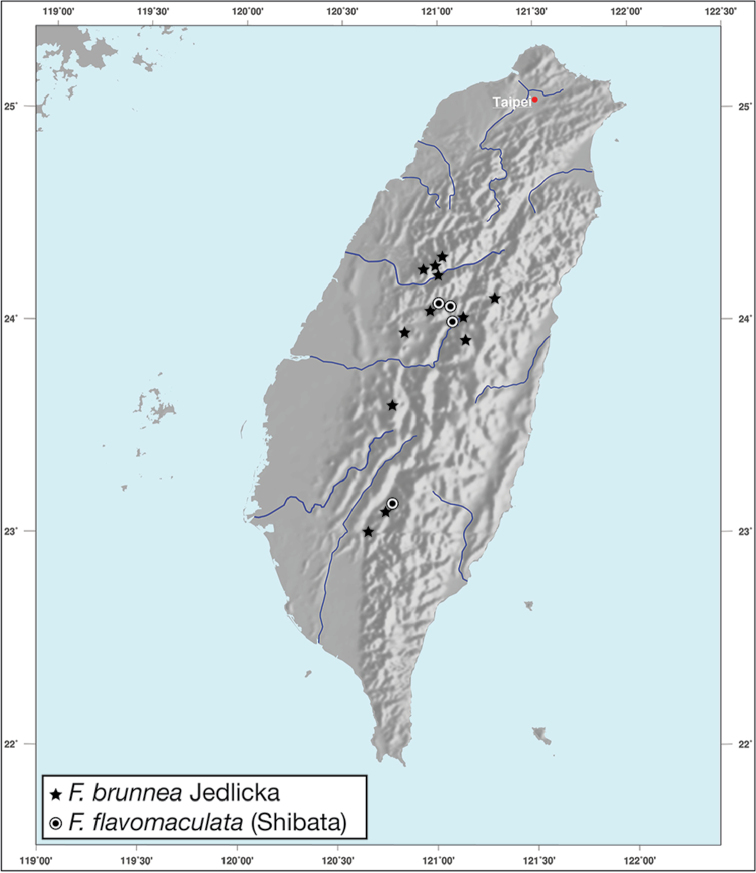
Map showing known localities for species of the genus *Formosiella* Jedlička, in Taiwan.

Figs 50B , 75 , 76A–D , 77B , 78

Pseudomenarus flavomaculatus Shibata, 1964: 44; Jedlička 1963: 109; Lorenz 2005: 458.

########## Types and other material examined.

**Holotype** (male) labeled “Yona/Okinawa Is./7. VIII. 1964/M. YASUI”; “Holotype” [rectangular, red paper]; Pseudomenarus/flavomaculatus/SHIBATA, 1964/gen. et sp. nov.”; “NCHU# 100137”. One **paratype** and 45 other specimens of *F.flavomaculata*: 27 males and 18 females. For further details see EH Strickland Virtual Entomology Museum Database.

########## Type locality.

Japan. Okinawa Island.

########## Diagnosis.

Specimens of this species are easily distinguished from the only other Taiwanese species of the genus by the elytral disc having four diffuse maculae.

########## Redescription.

OBL 4.08 – 5.25 mm. Length (n = four males, seven females): head 0.48 – 0.56, pronotum 0.64 – 0.88, elytra 2.84 – 3.33, metepisternum 0.68 – 0.84 mm; width: head 0.92 – 1.08, pronotum 1.24 – 1.52, elytra 2.25 – 2.79, metepisternum 0.46 – 0.52 mm.

*Body proportions*. HW/HL 1.82 – 2.08; PWM/PL 1.59 – 1.94; EL/EW 1.15 – 1.25; ML/MW 1.42 – 1.78 mm.

*Color*. Fig. 75. Dorsum of head, clypeus and labrum brunneous to rufo-brunneous, antennae and palpi testaceous to brunneous; pronotum brunneous, lighter than head, margins brunneo-testaceous to brunneous, slightly light than disc; elytral disc brunneous to rufo-brunneous, margins slightly lighter, elytral disc with four diffuse, testaceous macula (appearing as six or eight smaller maculae when apical macula is reduced and broken), two to six anterior and two posterior, anterior macula crescent shaped, crescent open towards elytra base, from suture or stria 1 to interval 7 or 8, typically interrupted in intervals 3 and 5, posterior macula in apical 1/3, ovoid, from interval 1 to interval 4 (sometimes stria 1); ventral surface testaceous to brunneo-testaceous, metepisternum and lateral margins darker; legs testaceous, tibia with dorsal surface rufo-piceous to piceous.

*Microsculpture*. Remaining dorsal surface with microsculpture not visible at 50×; ventral surface with microsculpture of metepisternum and lateral margins very faintly and shallowly transverse, all other surfaces with microsculpture not visible at 50×.

*Macrosculpture*. Dorsum of head with, scattered, setigerous punctures, punctures easily visible at 50×, setae very short in comparison to pronotum and elytra; pronotum disc with single, shallow depression medially on each side, lateral margins shallowly rugulose, entire surface with scattered, setigerous punctures, some bearing longer setae, easily visible in lateral view; elytra with intervals somewhat flat, entire surface with scattered, setigerous punctures, punctures relatively large and dense, some confluent, some punctures with longer setae (typically at center of intervals), easily visible in lateral view, striae with fine setigerous punctures along length; ventral surface of body, except base of head, with scattered setigerous punctures, easily visible at 50×.

*Fixed setae.* Pronotum with one pair of setae at base of lateral margin; 15–16 lateral (umbilical) setae in interval 9; elytra with interval 3 with three setae, one seta in basal 1/4, one seta at mid-length, one seta in apical 1/4, these setae can be difficult to observe in some specimens.

*Luster*. Dorsal and ventral surface moderately glossy.

*Head*. Mandibles short, almost entirely covered by labrum; labrum bilobed, longer than wide, rounded at apex; mentum with tooth; eyes somewhat convex; palpi (Fig. 50B) cylindrical, pointed at apex and setose.

*Pronotum*. Lateral margins explanate, with margins curved slightly upwards; anterior transverse impression moderately shallow; posterior transverse impression deep; median longitudinal impression moderately shallow; apico-lateral margins broadly lobed, posterio-lateral margins sinuate, slightly obtuse.

*Elytra*. Broadly rounded, hind angles truncate.

*Legs*. Males with adhesive vestiture ventrally, few squamo-setae at apex of tarsomeres 2 and 3 of fore-leg; males with one notch apically on ventral side of mid-tibia.

*Male genitalia*. Fig. 80A–D. Length 0.88 – 1.00 mm. Phallus short and distinctly wide medially, apex short, rounded at tip; endophallus with base distinctively wide and somewhat triangulate, narrowing to approximately basal 1/3 and then expanding again somewhat towards apex, two somewhat sclerotized and elongate endophallic flagellum (ef), laterally on each side of triangular basal lobe, one from base to constriction, one from base and well beyond constriction.

*Female genitalia*. Fig. 77B. Width 0.72 – 0.76 mm. Gonocoxite 2 (gc2) narrowing from base to apex; two lateral ensiform setae in close proximity (les), one dorsal ensiform seta. Two spermatheca present, spermatheca 1 (sp1) distinctly elongate and cylindrical, rounded at apex, spermatheca 2 (sp2) similar in form to spermatheca 1 but much shorter, attachment site on spermatheca 1 at approximately 1/3 length; one ovoid spermathecal accessory gland (sg), associated spermathecal gland duct (sgd) with swelling before apex, attachment site near base of spermatheca 2.

########## Habitat, habits, and seasonal occurrence.

The known elevational range of *F.flavomaculata* is from 500 to 1610 meters, with only one specimen known from over 920 meters. Adults are crepuscular or nocturnal and found in mixed primary and secondary forest of montane areas. Little is known about the habits of this species. Seven of the eleven known specimens were collected in disturbed forest from discrete hiding spots or under bark of deadwood by WH. Specimens have been collected from August to December. Known methods of collection are m.v. light sheet and hand collecting.

########## Geographical distribution.

*Formosiellabrunnea* is known from Japan (Ryukus and Okinawa Islands) and Taiwan See Figure 78.

######### Genus Holcoderus

Taxon classificationAnimaliaColeopteraCarabidae

Chaudoir

Holcoderus Chaudoir, 1869: 153; Csiki 1932: 1360; Andrewes 1933: 350; Jedlička 1940: 41; Louwerens 1949: 50; Louwerens 1953: 93; Darlington 1968: 94; Darlington 1970: 41; Habu 1979: 2; Habu 1979b: 51; Lorenz 2005: 455.

########## Type species.

*Holcoderuspraemorsus* Chaudoir, 1869.

######### Genus Wagneria

Taxon classificationAnimaliaColeopteraCarabidae

Jedlička, 1935: 7 (preoccupied)

Neritola Strand, 1942: 392.

########## Type locality.

Sri Lanka (Ceylon).

######### Holcoderus formosanus

Taxon classificationAnimaliaColeopteraCarabidae

Jedlička

**Figure 79.**
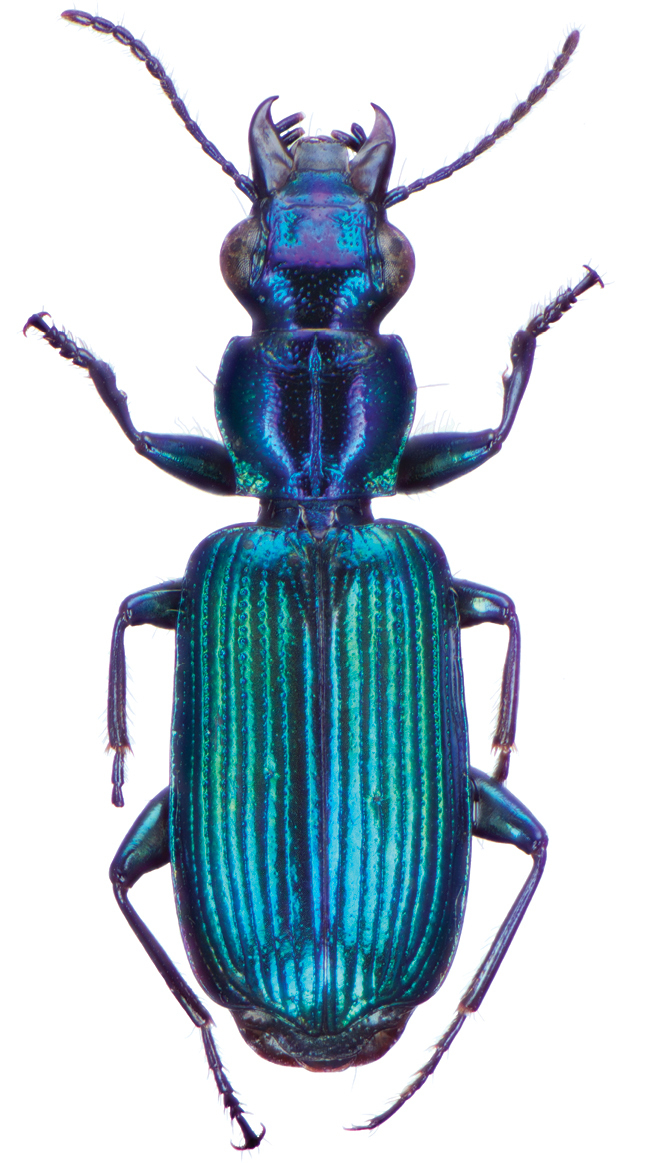
Dorsal habitus and color pattern of *Holcoderusformosanus* Jedlička. (OBL 8.58 mm).

**Figure 80.**
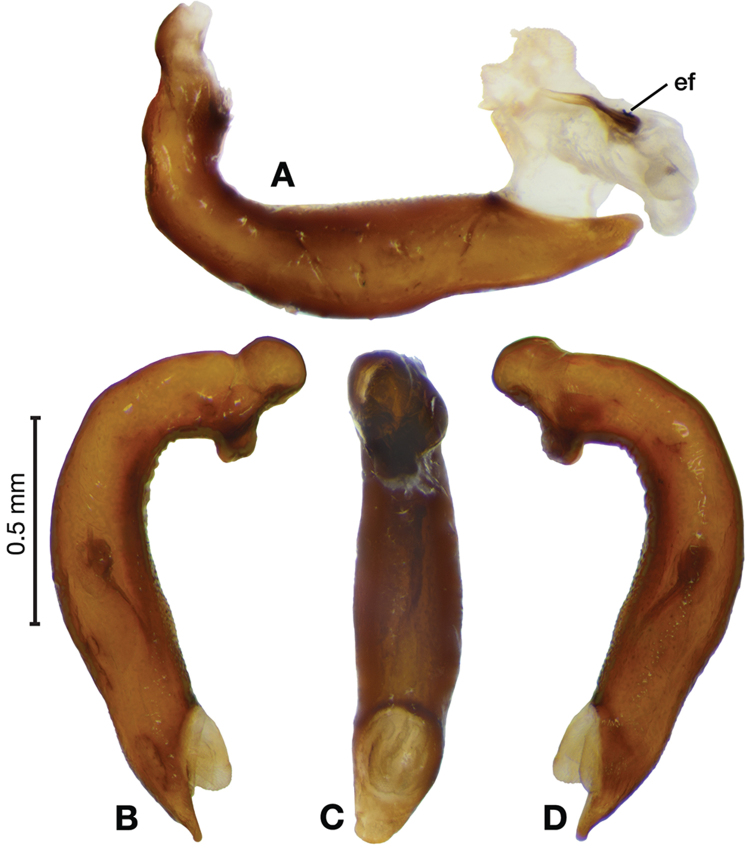
Digital images of male genitalia of *Holcoderusformosanus* Jedlička. **A** right lateral aspect, endophallus everted **B** right lateral aspect **C** ventral aspect **D** left lateral aspect. Legend: **ef** endophallic flagellum.

**Figure 81.**
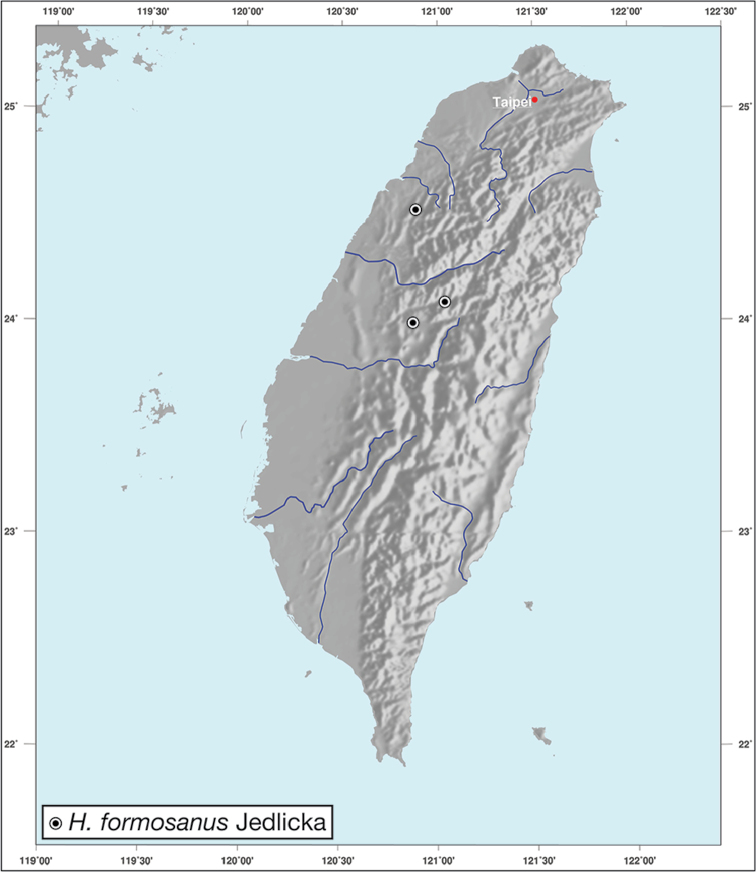
Map showing known localities for species of the genus *Holcoderus* Chaudoir, in Taiwan.

Figs 79 , 80A–D , 81 , 94A

Holcoderus formosanus Jedlička, 1940: 14; Habu 1979b: 51; Lorenz 2005: 455.

########## Types and other material examined.

**Holotype** (male) labeled “Hakure/9. IV. 1929/Col. T. Shiraki”; “TYPUS” [rectangle, red, black border]; “Holcoderus/formosanus/sp.n./DET. ING. JEDLICKA”; “NCHU# 100117”. Four specimens of *H.formosanus*: three males and one female. For further details see EH Strickland Virtual Entomology Museum Database.

########## Type locality.

Taiwan. “Horisha” = Puli

########## Diagnosis.

Specimens of this species are easily distinguished from other Taiwanese pericalines by a combination of the small size, pronotum with narrow bead along margin lateral margin and the metallic blue to green dorsal coloration.

########## Redescription.

OBL 8.17 – 8.58 mm. Length (n = four males, one female): head 0.88 – 0.92, pronotum 1.44 – 1.62, elytra 4.42 – 4.75, metepisternum 1.14 – 1.20 mm; width: head 1.72 – 1.88, pronotum 1.76 – 1.96, elytra 2.58 – 2.92, metepisternum 0.52 – 0.54 mm.

*Body proportions*. HW/HL 1.95 – 2.09; PWM/PL 1.19 – 1.23; EL/EW 1.60 – 1.71; ML/MW 2.19 – 2.30.

*Color*. Fig. 79. Varies depending on lighting and angle of observation. Dorsum of head and clypeus metallic blue to violaceous, dark, labrum metallic green to cupreous, antennae and palpi brunneo-piceous; disc of pronotum metallic blue to violaceous, some specimens with basal fovea and lateral margins metallic green; elytral disc metallic blue to metallic green, some specimens slightly violaceous; ventral surface piceous; legs brunneo-piceous to piceous.

*Microsculpture.* Head with very shallow microsculpture, almost isodiametric to isodiametric; pronotum with very shallow microsculpture, transverse; disc of elytra with transverse microsculpture, some places near suture approaching isodiametric, easily visible at 50×; ventral surface with transverse microsculpture.

*Macrosculpture and pilosity*. Dorsum of head and pronotum with scattered setigerous punctures, punctures easily visible but setae hardly so at 50×, pronotum faintly rugulose laterally; elytra with shallow lateral depression at basal 1/5, interval 7 carinate in basal half, other intervals moderately flat, each interval with a single row of setigerous puncture centrally; ventral surface with randomly scattered punctures, some bearing setae.

*Fixed setae.* Two pairs of supraorbital setae; clypeus with two long, lateral setae; labrum with six setae along apical margin; one pair of suborbital setae; pronotum with two pairs of setae, one at base of lateral margin and one on lateral margin at pronotum max width; 15–16 lateral (umbilical) setae in interval 9; elytra with interval 3 with three setae, first on outside of interval in basal 1/5, second on outside of interval at mid-length, third on inside of interval near apex; ventral surface with two setae on each of abdominal sterna III to VI; four setae along apical margin of sternum VII; legs with ventral surface of fore-femur with dense brush of long setae, less so in females.

*Luster*. Dorsal surface glossy; ventral surface moderately glossy to glossy.

*Head*. Mandibles curved at apex; labrum bilobed, shallowly emarginate; mentum with tooth; eyes moderately convex; palpi cylindrical and elongate.

*Pronotum*. Anterior transverse impression shallow; posterior transverse impression moderately deep, median longitudinal impression deep; lateral margins narrow, posterio-lateral margins slightly sinuate, almost right-angled.

*Elytra*. Elytra relatively long and narrow, hind angles truncate.

*Hind wings*. Macropterous.

*Legs*. Tarsal claws denticulate, three to four denticles per claw, claws relatively short; males with adhesive vestiture ventrally, two rows squamo-setae on tarsomeres 1–3 of fore-leg, males with several shallow notches apically on ventral side of mid-tibia.

*Male genitalia*. Fig. 80A–D. Length 1.28 – 1.44 mm. Ostium catopic. Phallus cylindrical, distinctly curved at base, almost to right angle, apex short, broadly rounded; endophallus short, widely expanded laterally in apical portion, two long, distinctive flagellum (ef) near apex of lateral expansion

*Female genitalia*. Fig. 94A. Width 1.16 mm. Gonocoxite 2 (gc2) wide from base to midlength, beyond midlength, narrowing, somewhat spatulate; two lateral ensiform setae in close proximity (les), one dorsal ensiform seta (des). Sensory furrow, furrow pegs and associated nematiform setae not observed. One spermatheca present (sp1), somewhat elongate, cylindrical and pointed at apex, distinctive diverticulum (div) at spermatheca base; one spermathecal accessory gland (sg), round, associated spermathecal gland duct (sgd) with attachment site near apex of spermatheca.

########## Habitat, habits, and seasonal occurrence.

From the few specimens examined, it appears that this is a low-land species. It is possible that this species is diurnal because in three years of night collecting, we have not encountered it. Specimens have been collected in April and May. All known specimens were hand collected.

########## Geographical distribution.

*Holcoderusformosanus* is known only from Taiwan. See Figure 81.

######### Genus Horniulus

Taxon classificationAnimaliaColeopteraCarabidae

Jedlička

Horniulus Jedlička, 1932: 85; Louwerens 1953: 88; Jedlička 1963: 371; Lorenz 2005: 463.

########## Type species.

*Horniulusandrewesi* Jedlička, 1932.

########## Type locality.

Taiwan. Suisharyo.

######### Horniulus andrewesi

Taxon classificationAnimaliaColeopteraCarabidae

Jedlička

**Figure 82.**
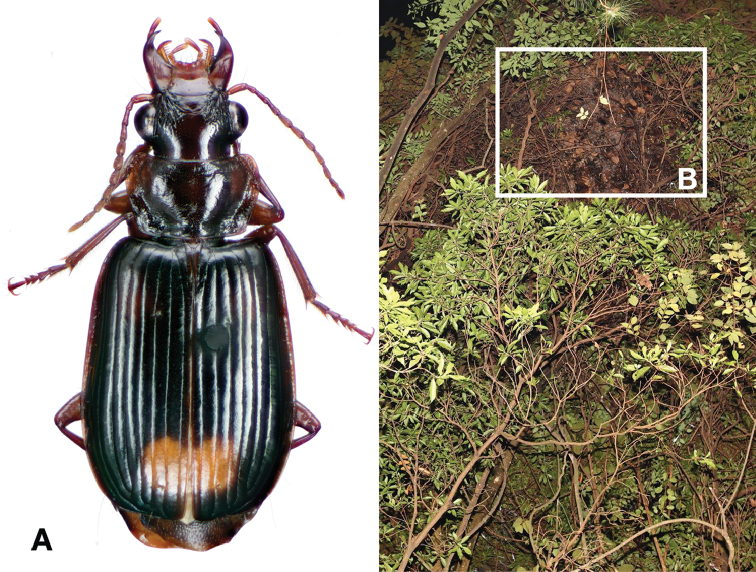
**A** Dorsal habitus and color pattern of *Horniulusandrewesi* Jedlička. (OBL 10.20 mm). **B** mixed forest dead leaf mass where several specimens were collected by insecticidal fogging.

**Figure 83.**
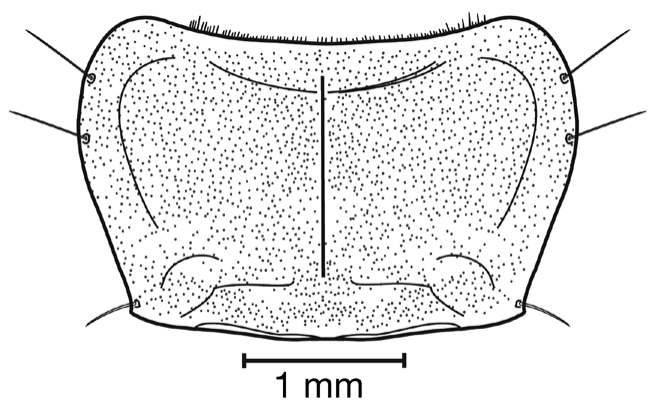
Line drawing, dorsal view, showing the form and 3 pairs of latero-marginal setae of the pronotum of *Horniulusandrewesi* Jedlička.

**Figure 84.**
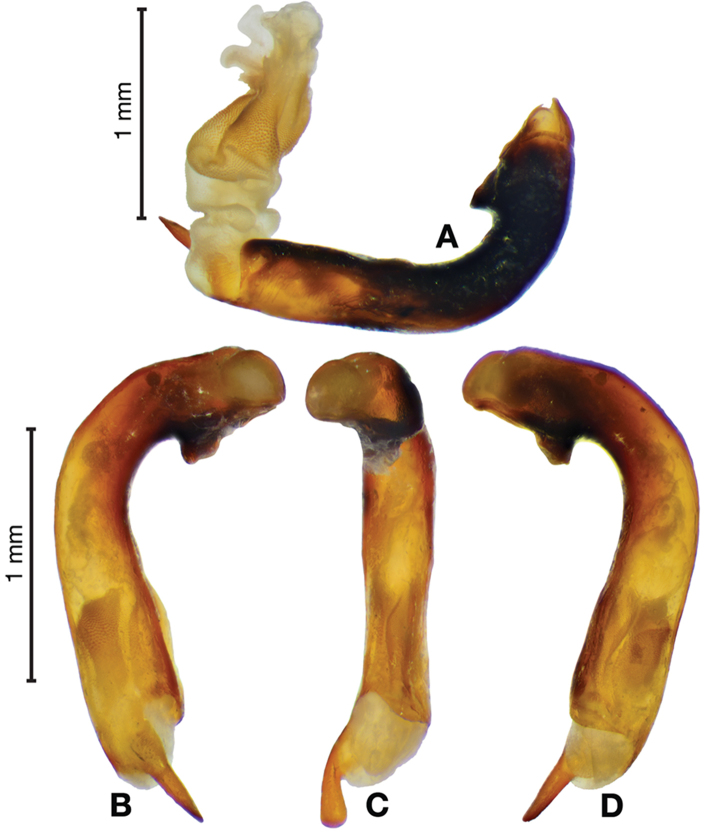
Digital images of male genitalia of *Horniulusandrewesi* Jedlička. **A** right lateral aspect, endophallus everted **B** right lateral aspect **C** ventral aspect **D** left lateral aspect.

**Figure 85.**
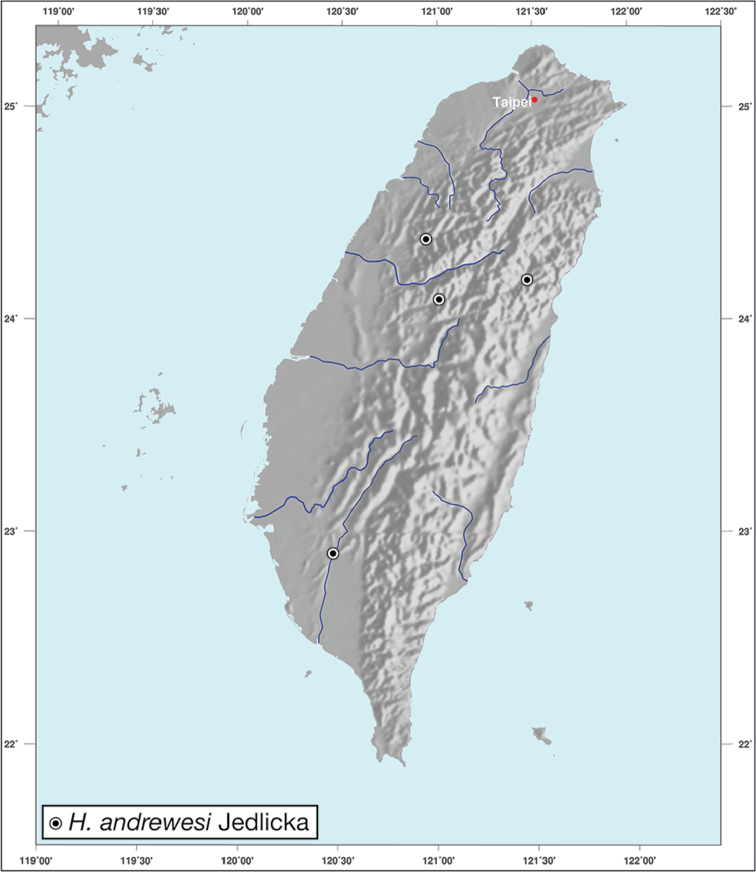
Map showing known localities for species of the genus *Horniulus* Jedlička, in Taiwan.

Figs 82A, B , 83 , 84A–D , 85 , 94B

Horniulus andrewesi Jedlička, 1932: 86; Louwerens 1953: 93; Lorenz 2005: 463.

########## Types and other material examined.

**Holotype** (male) labeled “Banshoryo – Disfr./Sokutsu (Formosa)/H. Sauter VI.1912”; “7.VIII”; “TYPE”[rectangular, red paper, black border]; Horniulus n.g./Andrewesi mihi/DET.ING.JEDLICKA”; “Holotypus”; “DEI Coleoptera/# 200417”. One **cotype** and 36 other specimens of *H.andrewesi*: 15 males and 21 females. For further details see EH Strickland Virtual Entomology Museum Database.

########## Type locality.

Taiwan. “Sokutsu, Banshoryo dist. = Chisan, Suisharyo.

########## Diagnosis.

Specimens of this species are easily distinguished from other Taiwanese pericalines by a combination of two pairs of supraorbital setae and three pairs of latero-marginal setae on the pronotum.

########## Redescription.

OBL 9.33 – 10.50 mm. Length (n = ten males, ten females): head 0.90 – 1.00, pronotum 1.60 – 1.76, elytra 5.33 – 6.17, metepisternum 1.36 – 1.64 mm; width: head 2.32 – 2.52, pronotum 2.68 – 2.94, elytra 4.00 – 4.75, metepisternum 0.70 – 0.84 mm.

*Body proportions*. HW/HL 2.44 – 2.74; PWM/PL 1.63 – 1.76; EL/EW 1.25 – 1.42; ML/MW 1.81 – 2.10.

*Color*. Fig. 82A. Dorsum of head, clypeus and labrum rufous to rufo-piceous, dark; antennae and palpi rufo-brunneous; disc of pronotum rufous to rufo-piceous; elytral disc piceous, some specimens with faint and rufous to testaceous macula in basal 1/3, diffuse, all specimens with testaceous macula in apical 1/3, from suture to stria 3 or 5; ventral surface brunneo-testaceous; legs brunneo-testaceous, tibia rufous, darker.

*Microsculpture.* Head and pronotum with microsculpture no visible at 50×; disc of elytra with shallow, transverse microsculpture; ventral surface with shallow transverse microsculpture.

*Macrosculpture*. Dorsum of head with center of disc smooth, laterally, somewhat rugulose, entire surface with scattered setigerous punctures, punctures easily visible but setae hardly so at 50×, punctures larger and more dense in rugulose area; pronotum with fine, scattered setigerous punctures, setae hardly visible at 50×; elytral intervals moderately flat, with some very fine, scattered punctures on disc, striae finely punctate, setae hardly visible at 50×; ventral surface with randomly scattered punctures.

*Fixed setae.* Two pairs of supraorbital setae; clypeus with two long, lateral setae; labrum with six setae along apical margin; one pair of suborbital setae; pronotum with three pairs of setae, one at base of lateral margin, one on lateral margin at pronotum max width and one half way between pronotum max width and apex of lateral margin; 15–16 lateral (umbilical) setae in interval 9; elytra with interval 3 with two setae, first on outside of interval at basal 1/3 elytra length, second on inside of interval in apical 1/6; ventral surface with two setae on each of abdominal sterna III to VI; four to six setae along apical margin of sternum VII.

*Luster*. Dorsal surface moderately glossy; ventral surface moderately glossy to glossy.

*Head*. Mandibles markedly curved in apical 1/3; labrum emarginate, apical setae punctures confluent with apical edge, making edge look bumpy; mentum with shallow tooth; eyes moderately convex; palpi cylindrical and elongate.

*Pronotum*. Fig. 83. Anterior transverse impression shallow; posterior transverse impression shallow, median longitudinal impression shallow; posterio-lateral margins slightly sinuate, obtuse.

*Hind wings*. Macropterous.

*Legs*. Tarsal claws smooth, males with adhesive vestiture ventrally, two rows squamo-setae on tarsomeres 1–3 of fore-leg; males with one notch apically on ventral side of mid-tibia.

*Male genitalia*. Fig. 84A–D. Length 1.80 – 1.92 mm. Ostium markedly catopic, open laterally on both left and right side. Phallus cylindrical, distinctly curved at base, almost to right angle, apex expanded and rounded, spatulate in ventral view; endophallus with microtrichia somewhat sclerotized, relatively dark from mid-length to apex.

*Female genitalia*. Fig. 94B. Width 1.28 – 1.56 mm. Gonocoxite 2 (gc2) narrowing from base to apex; two lateral ensiform setae (les), one dorsal ensiform seta (des). Sensory furrow, furrow pegs and associated nematiform setae not observed. One spermatheca present (sp1), somewhat elongate and cylindrical, distinctive diverticulum (div) at spermatheca base; one spermathecal accessory gland (sg), associated spermathecal gland duct (sgd), long, with attachment site near apex of diverticulum; bursa copulatrix sclerite (bsc) (type 2) in basal portion of bursa, with distinctive taco-shaped structure internally, differing from those seen in some members of *Catascopus* (Fig. 33A–C) in that it is open to the inside of the bursa, opening facing apically.

########## Habitat, habits, and seasonal occurrence.

The known elevational range of *H.andrewesi* is from 100 to 1000 meters. Adults are found in mixed primary and secondary forest of montane areas. Previous to this study, *H.andrewesi* was known from only six specimens and no collection data was available. An additional 28 specimens were collected, one from malaise trap and 27 from insecticidal fogging of one particular mass of dead leaves and brush (Fig. 82B) that was suspended in the forest canopy. The malaise trap specimen and all presumed hand collected specimens were collected from May to August. All material collected from fogging was in October and December. It is possible that the dead leaf mass provided a good estivation site for this species.

########## Geographical distribution.

*Horniulusandrewesi* is known only from Taiwan. See Figure 85.

########## Material examined.

36 specimens of *H.andrewesi*: 15 males and 21 females. For further details see EH Strickland Virtual Entomology Museum Database.

######### Genus Lioptera

Taxon classificationAnimaliaColeopteraCarabidae

Chaudoir

Lioptera Chaudoir, 1869: 208; Heller 1903: 241; Csiki 1932: 1376; Louwerens 1953: 91; Jedlička 1963: 339; Lorenz 2005: 458; Park et al. 2006: 102.

########## Type species.

*Liopteraquadriguttata* Chaudoir, 1869 (monotypic).

########## Type locality.

Philippines.

######### Lioptera erotyloides

Taxon classificationAnimaliaColeopteraCarabidae

Bates

**Figure 86.**
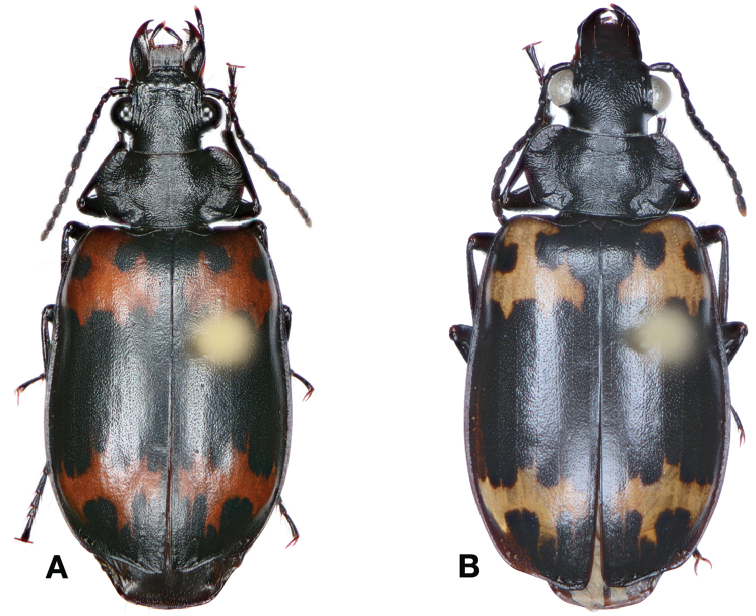
Dorsal habitus and intrapopulation variation of color pattern of *Liopteraerotyloides* Bates. **A** red elytral macula (OBL 15.20 mm) **B** orange elytral macula (OBL 11.92 mm).

**Figure 87.**
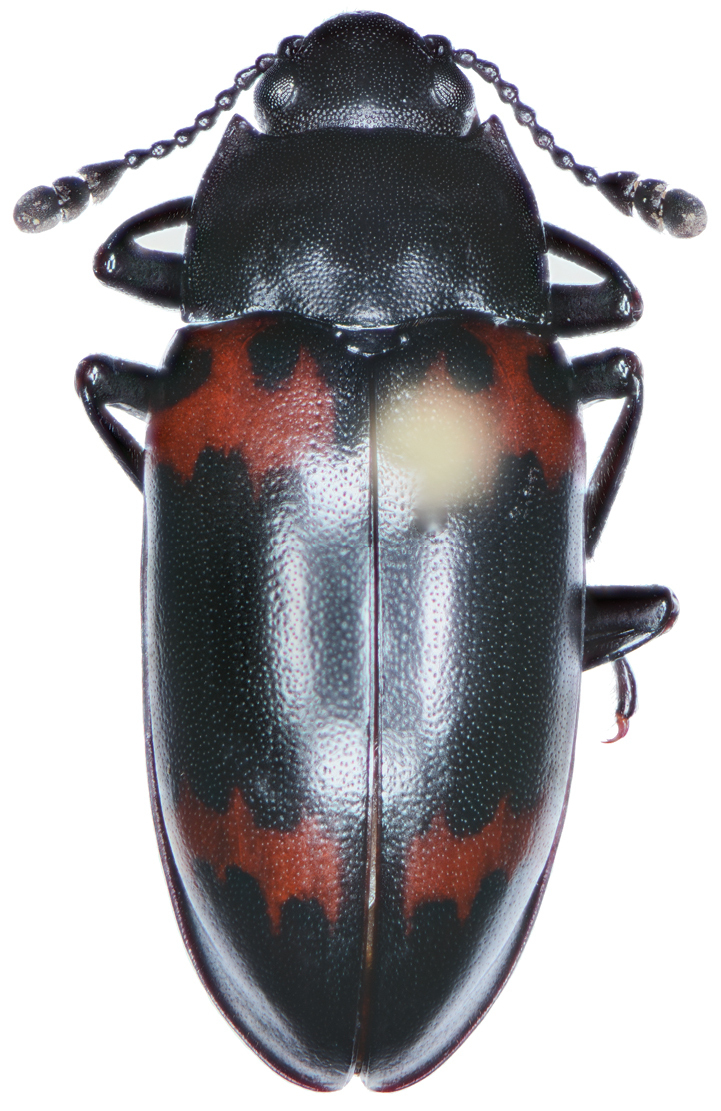
Dorsal habitus and color pattern of fungus beetle sp. (Erotylidae: Megalodacninae) observed on bracket fungus living together with both larvae and adults of *Liopteraerotyloides* Bates.

**Figure 88.**
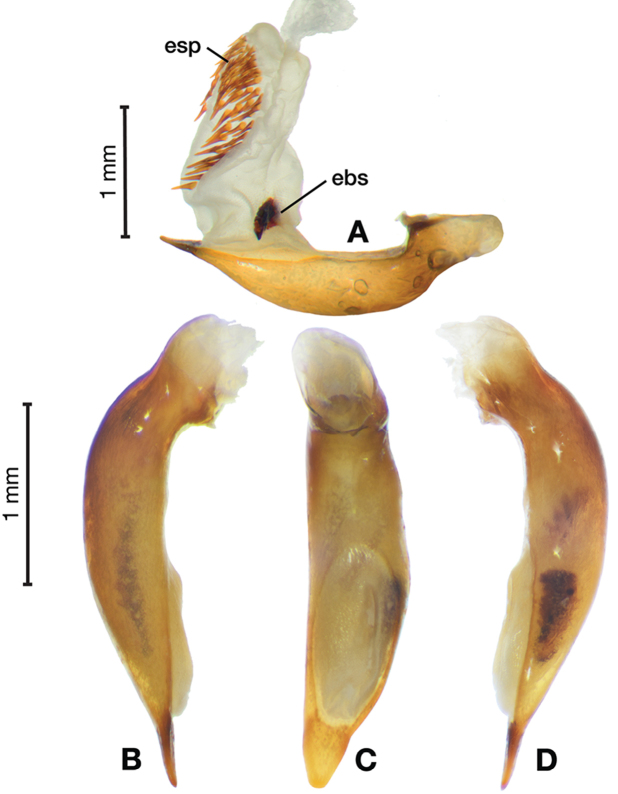
Digital images of male genitalia of *Liopteraerotyloides* Bates. **A** left lateral aspect, endophallus everted **B** right lateral aspect **C** ventral aspect **D** left lateral aspect. Legend: **ebs** endophallic basal spine; **esp** endophallic spine patch.

**Figure 89.**
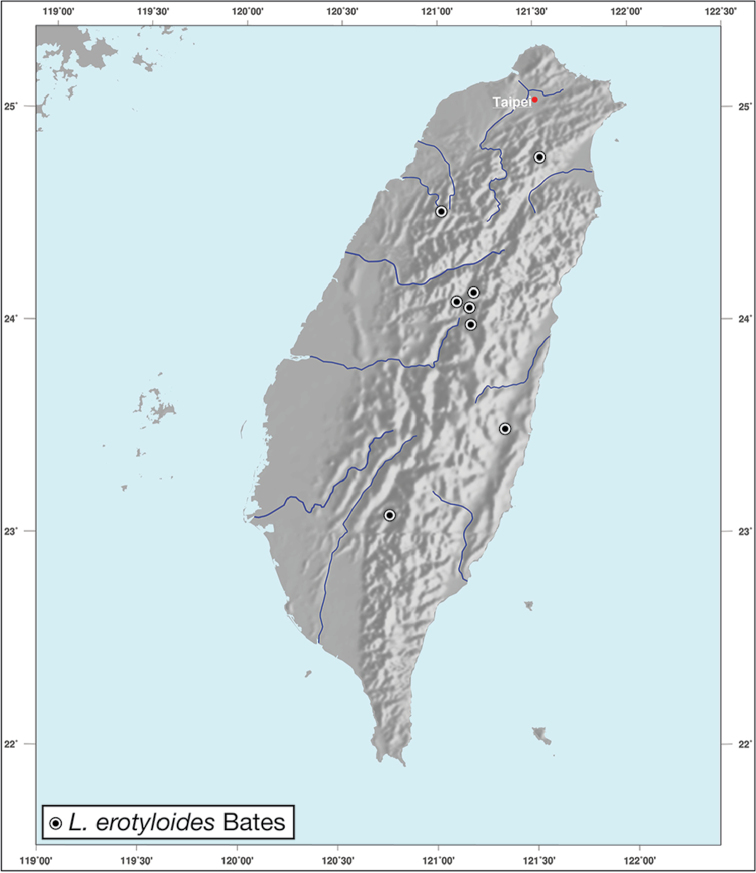
Map showing known localities for species of the genus *Lioptera* Chaudoir, in Taiwan.

Figs 86A, B , 87 , 88A–D , 89 , 94C

Lioptera erotyloides Bates, 1883: 280; Heller 1903: 245; Csiki 1932: 1376; Louwerens 1953: 92; Jedlička 1963: 340; Habu 1967: 97; Lorenz 2005: 458; Park et al. 2006: 102.

########## Types and other material examined.

**Holotype** (female) labeled “Type/HT” [circular, ringed with red]; “Japan/ G. Lewis/ 1910-320”; “Yuyama/ 16. V.-14. V.81”; Liopteraerotyloides Bates [handwritten]; NCHU#/100013. [NMNH]. 14 specimens: six males and eight females. For further details see EH Strickland Virtual Entomology Museum Database.

########## Type locality.

Japan. Yuyama.

########## Diagnosis.

Specimens of this species are easily distinguished from other Taiwanese pericalines by the large size (more than 11 mm), almost flat elytral intervals, and a mentum with no tooth.

########## Redescription.

OBL 11.50 – 15.33 mm. Length (n = six males, eight females): head 1.12 – 1.32, pronotum 1.68 – 2.08, elytra 7.75 – 9.83, metepisternum 1.76 – 2.32 mm; width: head 2.60 – 3.20, pronotum 3.28 – 4.20, elytra 5.50 – 7.08, metepisternum 0.96 – 1.32 mm.

*Body proportions*. HW/HL 2.30 – 2.58; PWM/PL 1.96 – 2.09; EL/EW 1.25 – 1.48; ML/MW 1.70 – 2.11.

*Color*. Fig. 86A, B. Various. Dorsum of head, clypeus, labrum, pronotum and antennae piceous; palpi piceous, lighter at apex; elytral disc piceous, with four yellow-orange to red maculae, two anterior and two posterior, anterior macula large and rather dentate, from interval 2, to just before outer margin, reaching basal border of elytra in interval 4 to 7, posterior macula more narrow laterally but also and rather dentate, from stria 1, to just before outer margin, closest to base of elytra in stria 4 and 5, closest to apex of elytra in stria 3 and 4; ventral surface piceous; legs rufo-piceous to piceous.

*Microsculpture.* Head and pronotum with microsculpture slightly transverse to isodiametric; disc of elytra with isodiametric sculpticells; ventral surface with shallow transverse to almost isodiametric microsculpture.

*Macrosculpture*. Dorsum of head, clypeus and pronotum rugulose, entire surface with scattered setigerous punctures, punctures blending with rugulose surface and setae hardly visible at 50×; elytral intervals flat, covered in dense, randomly scattered setigerous punctures, setae hardly visible at 50×, striae hardly visible, +/- evenly punctate along length; ventral surface with randomly scattered punctures.

*Fixed setae.* Two pairs of supraorbital setae; clypeus with two long, lateral setae; labrum with six setae along apical margin; one pair of suborbital setae; pronotum with two pairs of setae, one at base of lateral margin, one on lateral margin at pronotum max width, slightly inset; 29–30 lateral (umbilical) setae in interval 9; elytra with interval 3 with four setae, first in proximity to scutellar setae, second just beyond basal 1/5 of elytra, third 3/5 from base, fourth in proximity to last fixed umbilical seta; ventral surface with two setae on each of abdominal sterna III to VI; four setae along apical margin of sternum VII.

*Luster*. Dorsal surface moderately dull; ventral surface moderately glossy to glossy.

*Head*. Mandibles rather robust and long, only slightly curving at apex; labrum quadrate, some specimens with apical margin slightly emarginate; mentum without tooth; eyes large, convex; palpi cylindrical and elongate and setose.

*Pronotum*. Wide, twice as wide as long; lateral margins explanate, with margins curved upwards; anterior transverse impression moderately deep; posterior transverse impression moderately deep; median longitudinal impression very shallow; posterio-lateral margins obtuse.

*Elytra*. Hind angles truncate.

*Hind wings*. Macropterous.

*Legs*. Tarsal claws denticulate, 5–6 denticles per claw, males with adhesive vestiture ventrally, two rows squamo-setae on tarsomeres 1–3 of fore-leg, two rows of squamo-setae on tarsomeres 1–2 of mid-leg.

*Male genitalia*. Fig. 88A–D. Length 2.64 – 2.84 mm. Ostium catopic, rather elongate in ventral view, extended to mid-length of phallus. Phallus cylindrical, widest at mid-length, apex almost triangular in form, rounded at tip; endophallus relatively wide along length, with single, distinctively large basal spine (ebs) and field of moderately large spines (esp) visible on left side in lateral view.

*Female genitalia*. Fig. 94C. Width 1.68 – 1.92 mm. Gonocoxite 2 (gc2) relatively uniform in width along length, constricting sharply at base of dorsal ensiform setae fovia; two lateral ensiform setae spaced widely apart (les), one dorsal ensiform seta. Sensory furrow, furrow pegs and associated nematiform setae not observed. One spermatheca present (sp1), elongate and cylindrical, expanding slightly in apical half, distinctive diverticulum (div) at spermatheca base; one spermathecal accessory gland (sg), associated spermathecal gland duct (sgd), with attachment site near apex of divericulum.

########## Habitat, habits, and seasonal occurrence.

The known elevational range of *L.erotyloides* is from 250 to 1800 meters with the majority of adults being collected at around 1200 meters. Adults of this species are crepuscular and are found in mixed primary and secondary forest of montane areas. Specimens have been collected from April to December with most specimens collected in May and June. Methods of collecting include m.v. light sheet, malaise trap and hand collecting.

########## Collecting observations.

In June of 2011, collecting partner and laboratory colleague, Zong Hang Yang with WH collected for an evening at Aowanda National Forest Recreation Area, Nantou county. The site had been closed to the public for some time due to devastation caused by Typhoon Morakot, the previous year. Because of this, there was an abundance of deadwood and fallen trees in the area, being reclaimed by the land for several months. Situations like this can present an excellent opportunity for the collection of pericaline lebiines, due to their association with both the insects and fungi that require and use these microhabitats. That evening WH came across a broken stump that had the north side of it covered in a large patch of frilly, white, bracket fungus; within the folds of this fungus, were numbers of a large adults of large erotylid beetle (Erotylidae: Megalodacninae) (Fig. 87) together with their larvae, feeding on the fungus. There were dozens of adults and even more larvae. After observing for a time, several individuals of a pericaline lebiine also moving amongst them we observed, *L.erotyloides*, its name given due to the strikingly similar dorsal coloration it shares with several species of erotylids.

The erotylids and their larvae did not seem to be bothered at all by the presence of the carabids around them. Specimens of the erotylid beetle, the carabids, and also some larvae and associated fungus were collected. Upon researching this type of behavior, a paper by Erwin and Erwin (1976) that detailed the natural history of a New World species of pericaline beetle, *Eurycoleusmacularis* Chevrolat. Apparently, this species is closely associated with a fungus beetle of the genus *Amphix* (Endomychidae), with larvae and adults both relying on them as prey while living amongst them. Erwin considered the natural history of *E.macularis* as a form of incipient ectoparasitism. He postulated that this behavior could provide proof of an evolutionary intermediate step towards the true ectoparasitism recorded in a few other groups within the Carabidae.

The material was examined and no carabid larvae were present. Over the next three years of fieldwork, this phenomenon was not observed again. It seems possible that *L.erotyloides* may have a similar natural history as *E.macularis* but more observations are needed to uncover their true way of life and relationship to their erotylid namesake.

########## Geographical distribution.

*Liopteraerotyloides* is known from Korea, Japan, China, Vietnam, and Taiwan. For Taiwan collecting localities see Figure 89.

######### Genus Miscelus

Taxon classificationAnimaliaColeopteraCarabidae

Klug

Miscelus Klug, 1834: 82; Lacordaire 1854: 146; Putzeys 1845: 375; Putzeys 1875: 722; Chaudoir 1861: 125; Chaudoir 1869: 152; Bates 1869: 72; Andrewes 1922: 292; Csiki 1932: 1359; Darlington 1968: 91; Lorenz 2005: 454.
Leptodactyla Brulle, 1834: 130.

########## Type species.

*Miscelusjavanus* Klug, 1834 (monotypic)

########## Type locality.

Java.

######### Miscelus javanus

Taxon classificationAnimaliaColeopteraCarabidae

Klug

**Figure 90.**
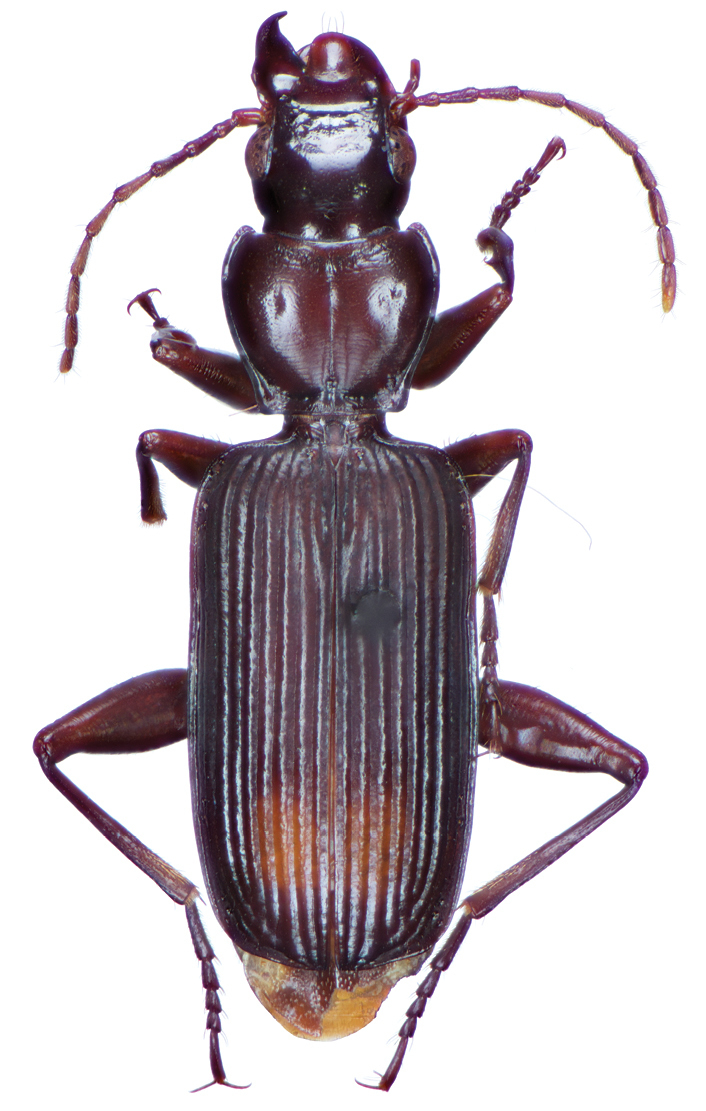
Dorsal habitus and color pattern of *Miscelusjavanus* Klug. (OBL 10.18 mm).

**Figure 91.**
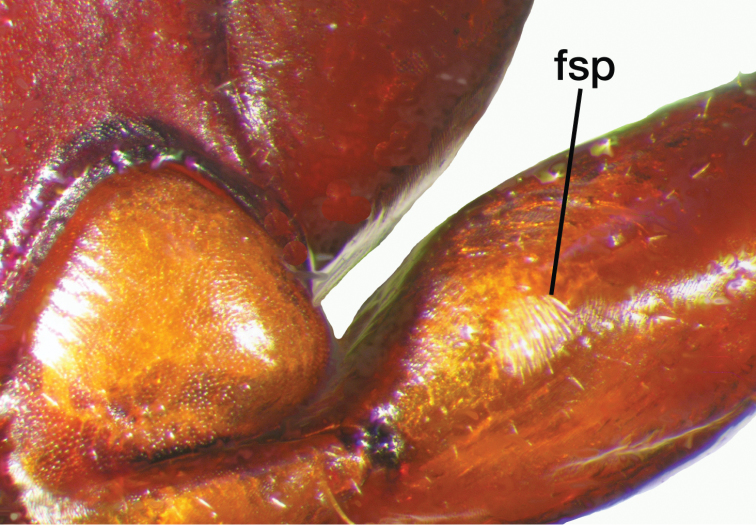
Digital image showing the patch of short dense seta present near the base of the fore-femur, ventral surface, of males of *Miscelusjavanus* Klug. Legend: **fsp** femoral setae patch.

**Figure 92.**
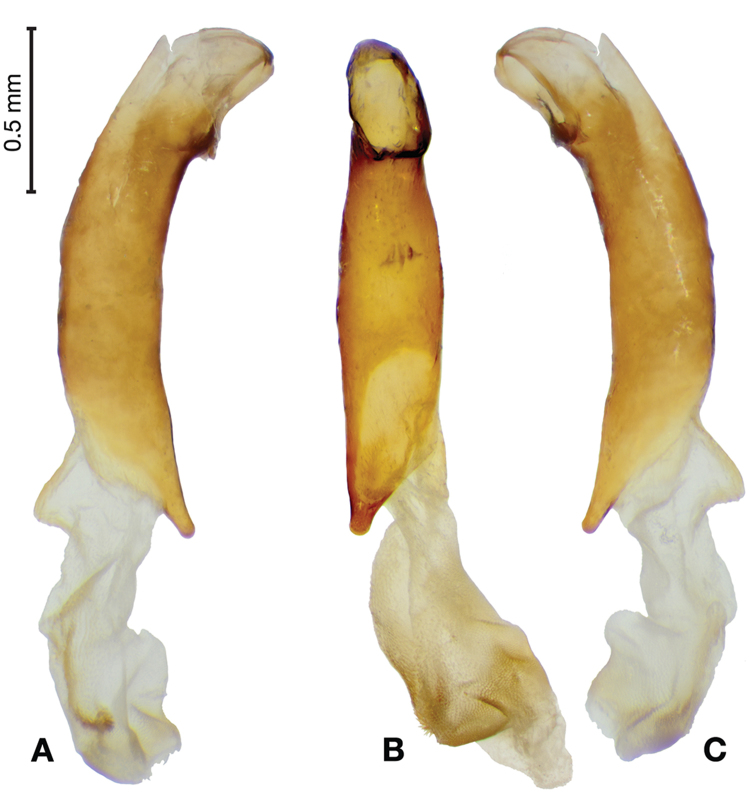
Digital images of the male genitalia, endophallus everted, of *Miscelusjavanus* Klug. **A** right lateral aspect **B** ventral aspect **C** left lateral aspect.

**Figure 93.**
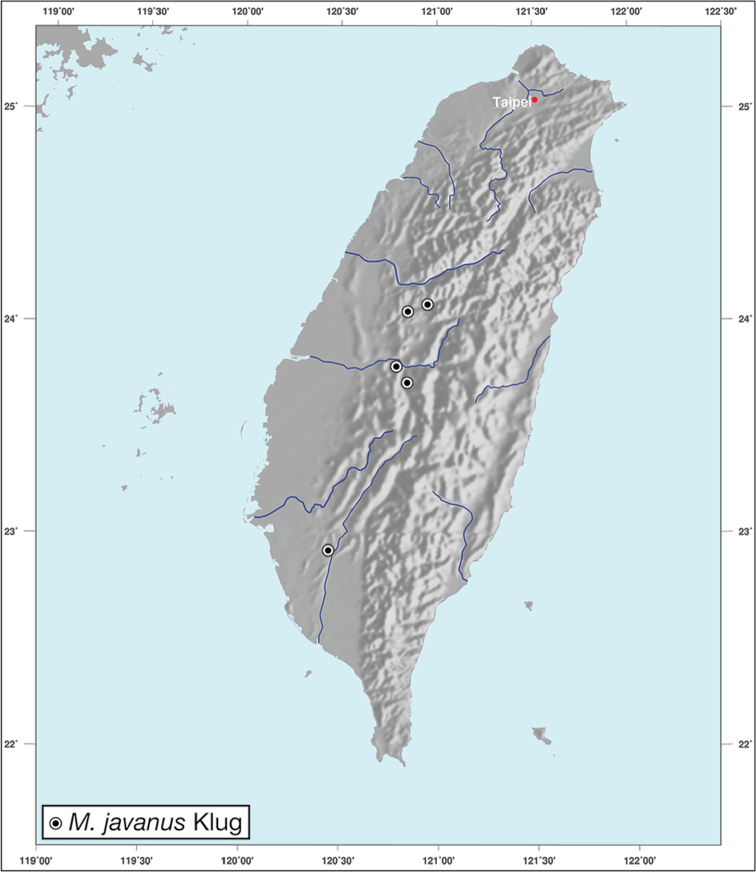
Map showing known localities for species of the genus *Miscelus* Klug, in Taiwan.

**Figure 94.**
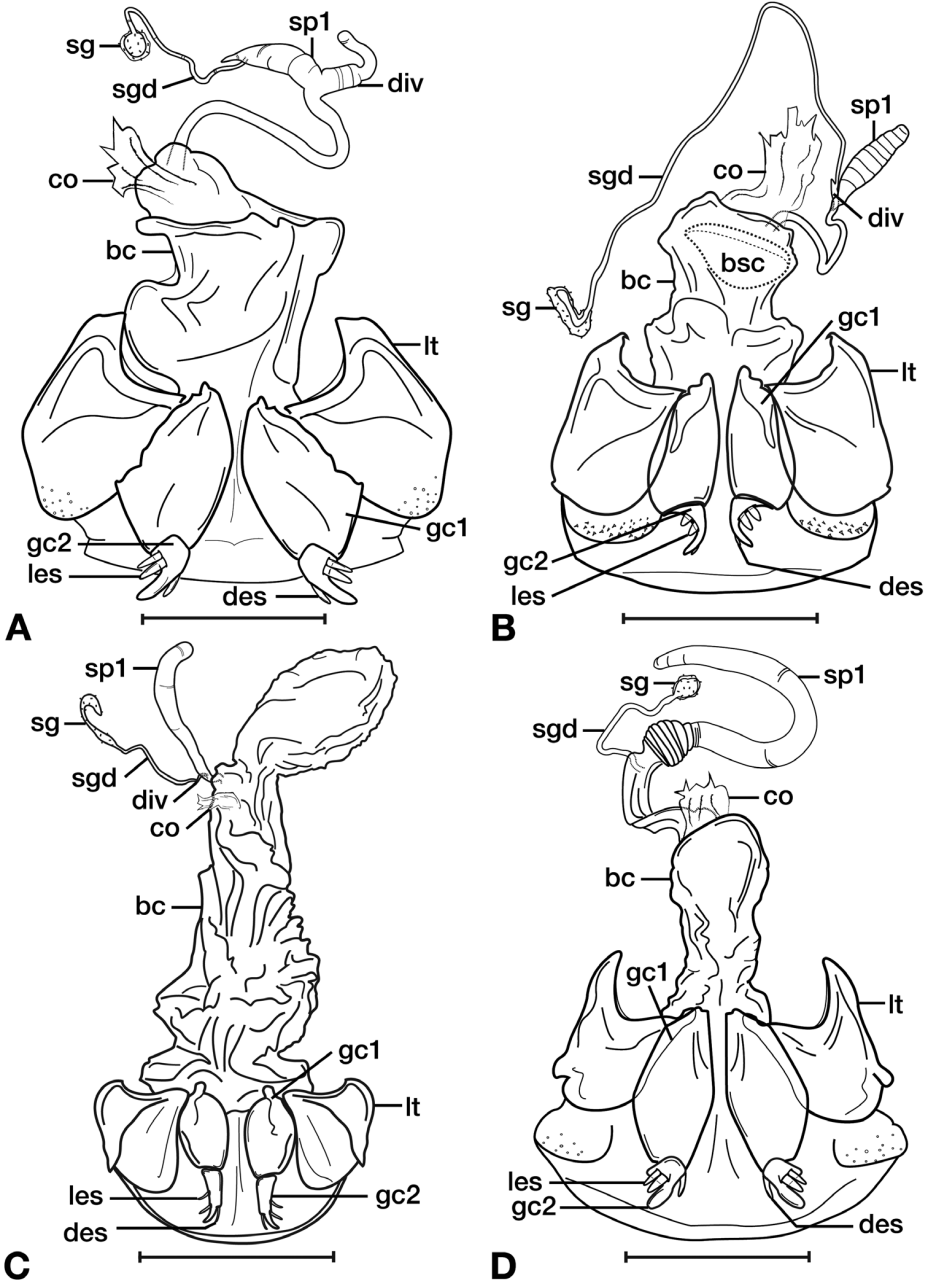
Line drawings of the female reproductive tract, ventral aspect, of **A***Holcoderusformosanus* Jedlička **B***Horniulusandrewesi* Jedlička **C***Liopteraerotyloides* Bates **D***Miscelusjavanus* Klug. Legend: **bc** bursa copulatrix; **bsc** bursal sclerite; **co** common oviduct; **des** dorsal ensiform setae; **div** diverticulum **gc1** gonocoxite 1; **gc2** gonocoxite 2; **les** lateral ensiform setae; **lt** lateral tergite; **sg** spermathecal gland; **sgd** spermathecal gland duct; **sp1** spermatheca 1. Scale bars: 0.5 mm.

Figs 90 , 91 , 92A–C , 93 , 94d

Miscelus javanus Klug, 1834: 82; Chaudoir 1861: 125; Putzeys 1875: 723; Bates 1892: 408; Andrewes 1922: 293; Csiki 1932: 1359; Jedlička 1963: 398; Darlington 1969: 94; Lorenz 2005: 454.
Miscelus apicalus Brulle, 1834: 130.
Miscelus convexicollis Putzeys, 1875: 724.
Miscelus paradoxus Putzeys, 1875: 724.
Miscelus vulneratus Putzeys, 1875: 725.
Miscelus planatus Schaufuss, 1885: 183; Andrewes 1922: 294.

########## Types and other material examined.

Nine specimens of *M.javanus*: four males and five females. For further details see EH Strickland Virtual Entomology Museum Database.

########## Type locality.

Java.

########## Diagnosis.

Specimens of this species are easily distinguished from other Taiwanese pericalines by having smooth tarsal claws and only a single pair of supraorbital setae

########## Redescription.

OBL 9.17 – 11.67 mm. Length (n = four males, five females): head 0.80 – 0.92, pronotum 1.64 – 2.00, elytra 5.17 – 6.60, metepisternum 1.32 – 1.72 mm; width: head 1.64 – 2.52, pronotum 2.14 – 2.68, elytra 2.83 – 3.00, metepisternum 0.68 – 0.84 mm.

*Body proportions*. HW/HL 2.05 – 2.86; PWM/PL 1.26 – 1.44; EL/EW 1.55 – 1.87; ML/MW 1.89 – 2.05.

*Color*. Fig. 90. Dorsum of head brunneous to rufo-piceous, clypeus brunneous to rufo-piceous, slightly lighter than head, labrum brunneous, slightly lighter than clypeus; antennae and palpi brunneous to rufo-brunneous; pronotum brunneous to rufo-piceous, slightly iridescent; elytral disc rufo-brunneous to rufo-piceous, dark, with testaceous macula, centrally in apical 1/3, from suture to interval 5, ovoid in appearance; ventral surface rufous to brunneo-testaceous; legs brunneo-testaceous, tibia brunneous to rufo-piceous, darker.

*Microsculpture*. Dorsum of head with microsculpture shallow, isodiametric; pronotum and elytra and ventral surface with microsculpture shallow, transverse.

*Macrosculpture*. Dorsum of head with fine, scattered, setigerous punctures, setae hardly visible at 50×, labrum rugulose in basal half of lateral margins; pronotum with fine, scattered setigerous punctures, setae hardly visible at 50×, very faintly rugulose laterally across disc, one small depression on either side of disc medially; elytral intervals somewhat raised, intervals 3, 5, 7 raised more than others, all intervals with +/- single row of setigerous punctures centrally, intervals 5, 6, 7 slightly rugulose laterally along length, striae with row of setigerous punctures, setae hardly visible at 50×; ventral surface with randomly scattered punctures, setae easily visible, metasternum with two paramedian rows of seven to eight tubercles.

*Fixed setae.* One pair of supraorbital setae; clypeus with two long, lateral setae; labrum with six setae along apical margin; two suborbital setae, two long setae in gula; pronotum with two pairs of setae, one at base of lateral margin, one in apical 1/3 of lateral margin; 16 lateral (umbilical) setae in interval 9; ventral surface with two setae on each of abdominal sterna III to VI; four setae along apical margin of sternum VII in females, two setae along apical margin of sternum VII in males; base of fore-femur of males with small patch of short, dense setae on ventral surface.

*Luster*. Dorsal surface moderately dull; ventral surface moderately glossy to glossy.

*Head*. Mandibles rather robust and short, curving rather sharply at apex; labrum elongate, distinctively convex in apical half; mentum with tooth; eyes relatively flat; palpi cylindrical.

*Pronotum*. Lateral margins narrow, apical margin emarginate, anterior transverse impression shallow, slightly rugulose; posterior transverse impression moderately deep, median longitudinal impression moderately shallow, apico-lateral margin with distinctive lobes posterio-lateral margin slightly sinuate, almost right-angled.

*Elytra*. Hind angles truncate.

*Hind wings*. Macropterous.

*Legs*. Fig. 91. Tarsal claws smooth, males with adhesive vestiture ventrally, two rows squamo-setae on tarsomeres 1–3 of fore-leg, patch of short dense setae (fsp) near the base of the fore-femur, ventral surface.

*Male genitalia*. Fig. 92A–C. Length 1.28 – 1.44 mm. Ostium anopic. Phallus cylindrical, rather uniform along length, apex short, spatulate, rounded at tip; endophallus with distinctive basal lobe, small microtrichial field visible towards apex in right and ventral aspect.

*Female genitalia*. Fig. 94D. Width 1.06 – 1.08 mm. Gonocoxite 2 (gc2) with distinctive form, uniformly wide from base to mid-length, constricting sharply at right angle just after base of dorsal ensiform setae base, apex elongate and distinctly spatulate; two lateral ensiform setae (les), somewhat spatulate and close together, one dorsal ensiform seta. Sensory furrow, furrow pegs and associated nematiform setae not observed. One spermatheca present (sp1), elongate and cylindrical, distinctive, ribbed, lobe at base before elongate, curved portion; one spermathecal accessory gland (sg), ovoid to circular, associated spermathecal gland duct (sgd), with attachment site at base of spermatheca, below ribbed basal lobe.

########## Habitat, habits, and seasonal occurrence.

The known elevational range of *M.javanus* is from 100 to 650 meters in Taiwan. Over three years, only one specimen of this species was collected. It was during the day on a sandy riverbank. It is possible that this species is diurnal but little else is known. Specimens have been collected from February to December with most specimens collected in May. All known specimens were hand collected.

########## Geographical distribution.

*Miscelusjavanus* is known from Sri Lanka, India, Thailand, the Philippines, New Guinea, Australia, and Taiwan. For Taiwan collecting localities see Figure 89.

######### Genus Mochtherus

Taxon classificationAnimaliaColeopteraCarabidae

Schmidt-Goebel, 1846

Mochtherus Schmidt-Goebel, 1846: 76; Lacordaire 1854: 137; Bates 1869: 71; Chaudoir 1869: 240; Jakobson 1907:394; Csiki 1932:1382; Andrewes 1939: 185; Jedlička 1935: 18; Jedlička 1963: 361; Habu 1967: 100; Habu 1982: 86; Darlington 1968: 122; Lorenz 2005: 460.
Cyrtopterus Motschulsky, 1861: 106.

########## Type species.

*Mochtherusangulatus* Schmidt-Goebel (= *Dolichoctistetraspilotus* (MacLeay); designated by Andrewes 1939.

########## Type locality.

Burma (Myanmar).

########## Recognition of Taiwanese species of *Mochtherus*

########## .

*Color.* Various.

*Fixed setae.* Two pairs of supraorbital setae; clypeus with two lateral setae; labrum with six setae along apical margin; one pair of suborbital setae; pronotum with two setae along each margin, one at base of lateral margin and one on lateral margin at pronotum max width; 15 to 16 lateral (umbilical) setae in interval 9; two setae on each of abdominal sterna III to VI; two setae along apical margin of sternum VII in males, females with four setae near apical margin of sternum VII.

*Elytra*. Striae moderately impressed; elytral apices truncate.

*Hind wings*. Macropterous.

*Legs*. Males with adhesive vestiture ventrally, two rows of squamo-setae on tarsomeres 1–3 of fore-leg.

*Male genitalia*. Ostium left pleuropic. Phallus cylindrical.

*Female genitalia*. Gonocoxite 2 (gc2) wide at base, narrowing and curving outwards along length; one dorsal ensiform seta (des). Sensory furrow, furrow pegs and associated nematiform setae not observed.

########## Taxonomic notes.

Habu (1967, 1982) considered Mochtherus a subgenus of Dolichoctis. Based on the differences in mentum and female genitalic characters, *Mochtherus* is regarded as a valid genus.

######## Key to the Taiwanese species of the genus *Mochtherus* Schmidt-Goebel

**Table:** 

1	Elytra with four testaceous maculae	**2**
–	Dorsal surface entirely black	***Mochtherusluctuosus* Putzeys**
2	Size smaller, overall body length less than 7.5 mm; baso-medial half of elytra with seta and associated punctures not distinctively differing from remainder of disc	***Mochtherustetraspilotus* (MacLeay)**
–	Size larger, overall body length more than 7.5 mm; baso-medial half of elytra with setae long and dense, punctures wide, shallow and somewhat regularly spaced, giving a distinctive dull texture	***Moctherusobscurabasis* sp. n.**

######### Mochtherus luctuosus

Taxon classificationAnimaliaColeopteraCarabidae

Putzeys

**Figure 95.**
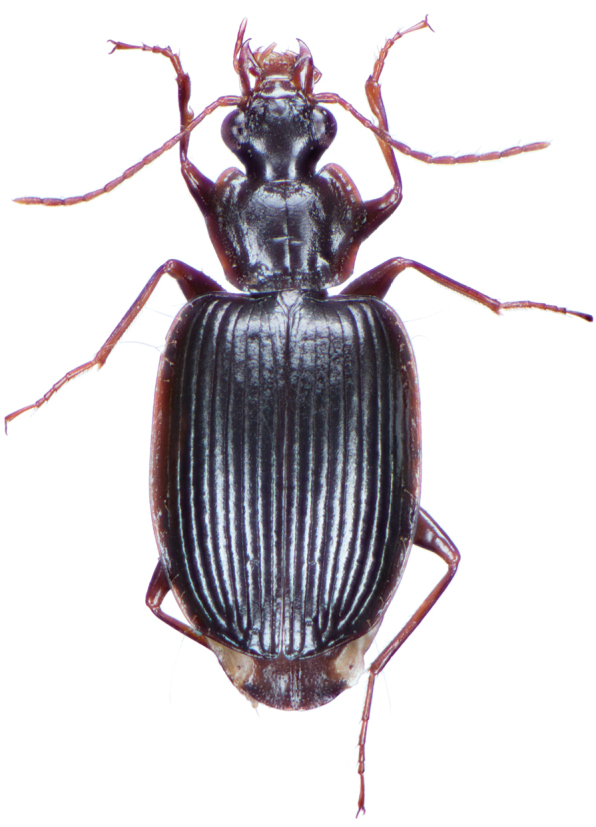
Dorsal habitus and color pattern of *Mochtherusluctuosus* Putzeys. (OBL 8. 43 mm).

**Figure 96.**
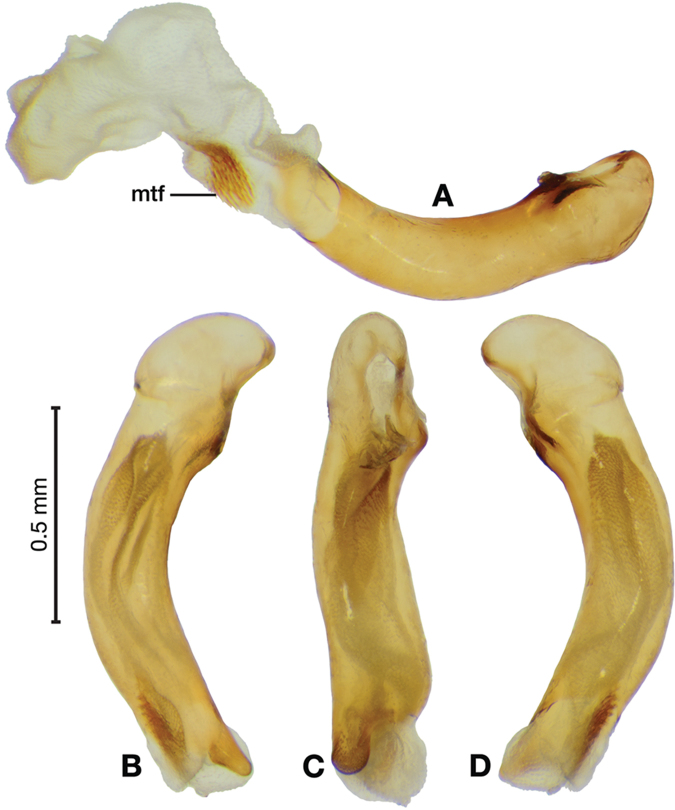
Digital images of male genitalia of *Mochtherusluctuosus* Putzeys. **A** left lateral aspect, endophallus everted **B** right lateral aspect **C** ventral aspect **D** left lateral aspect. Legend: **mtf** microtrichial field.

Figs 95 , 96A–D , 102A , 103

Mochtherus luctuosus Putzeys, 1875: pl. Lll; Bates 1883: 281; Csiki 1932: 1382; Jedlička 1963: 353; Lorenz 2005: 460.
Sinurus nitidus Bates: Habu, 1953: 50.
Sinurus luctuosus Putzeys: Habu and Baba, 1957: 17; Habu 1959: 9.
Dolichoctis (Mochtherus) uenoi Habu, 1967: 108. syn. n.

########## Types and other material examined.

39 specimens of *M.luctuosus*: 25 males and 14 females. For further details see EH Strickland Virtual Entomology Museum Database.

########## Type locality.

Japan. “S. Nipon”.

########## Taxonomic notes.

When Habu (1967) described *Mochtherusuenoi* from Taiwan, he acknowledged that it very closely resembled *M.luctuosus*. He based his description primarily on differences in two characters, pronotum form, and endophallus apex form. Upon examination of several specimens of this species, it became apparent that there was variation in both of these characters, as well as significant variation in overall body length.

Phallus apex form hardly differs from specimen to specimen; however, some individuals do have a slightly more pointed apex than others. Habu’s illustration of this is well within the range of variation observed. Variability in pronotum characteristics in this species is somewhat dramatic and if Habu had access to more material, he likely would have noticed that pronotum variation exists, even within local populations. Pronotum disc convexity is variable. Some individuals have a disc that is more convex and broadly rounded, while others are flatter in appearance. Pronotum margins are also variable. Some individuals have margins that are wider and more up-turned at the margins. This character can change the appearance on the sinuate baso-lateral margin that is typical of *Mochtherus*, making the sinuation appear less pronounced. Despite these differences, this species is easily distinguished in Taiwan as it is the only entirely black *Mochtherus* species present.

During the course of this work, a few specimens of the genus *Sinurus* looked very similar to specimens regarded as *M.luctuosus* from Taiwan. Non-type material from the type series of both *Sinurusnitidus* Bates and *Sinurusgraciliceps* Bates were dissected and the genitalic characters were very similar to all *Mochtherus* species examined.

Considering the variability observed within specimens of the species *M.luctuosus* from Taiwan, both of these *Sinurus* species belong in the genus *Mochtherus* and are likely conspecific with *M.luctuosus*. One female specimen of *S.opacus* Chaudoir, which was the first species described in *Sinurus*, was also dissected. It differs from the other two species in that the elytral microsculpture is granulated and the elytral margins are faintly serrate. The specimen was not in excellent condition but it was apparent that the general form of the gonocoxite, spermatheca, and associated gland were all very similar to that of *Mochtherus* species. It is possible that further work will show that this genus is congeneric with *Mochtherus*.

########## Diagnosis.

Specimens of this species are easily distinguished from other species of *Mochtherus* by the entirely black dorsal coloration,

########## Description.

OBL 7.50 – 11.00 mm. Length (n = ten males, ten females): head 0.72 – 0.90, pronotum 1.40 – 1.84, elytra 4.50 – 6.08, metepisternum 1.04 – 1.40 mm; width: head 1.44 – 1.92, pronotum 1.40 – 1.84, elytra 3.33 – 4.33, metepisternum 0.60 – 0.84 mm.

*Body proportions*. HW/HL 1.91 – 2.14; PWM/PL 1.36 – 1.53; EL/EW 1.24 – 1.45; ML/MW 1.42 – 1.89 mm.

*Color*. Fig. 95. Dorsum of head piceous, with small, diffuse patch just before clypeal suture, brunneous to brunneo-piceous, clypeus and labrum brunneous, some specimens with labrum darker centrally; palpi and antennae brunneous; pronotum disc brunneo-piceous to piceous, margins brunneous to piceous; elytral disc brunneous, dark, to piceous, suture and margins slightly lighter; ventral surface of head, metepisternum and lateral margins darker than other ventral surfaces, brunneous to piceous, all other surfaces brunneous, lighter to darker; legs brunneous to rufo-brunneous, most specimens with ventral surface of tibia darker, brunneo-piceous to piceous.

*Microsculpture*. Dorsum of head with mesh pattern somewhat granulate, isodiametric, somewhat transverse towards neck, clypeus with sculpticells stretched longitudinally, labrum with sculpticells shallow, almost isodiametric; pronotum with microsculpture transverse; elytra with sculpticells transverse; ventral surfaces with microsculpture transverse.

*Macrosculpture and pilosity*. Dorsum of head with scattered setigerous punctures, setae short and punctures fine, not distinctively visible, labrum with a few short setae in apical half; mandible with a few short setae just beyond apex of scrobe; pronotum with scattered setigerous punctures, punctures fine, setae short but uniform in length and longer than the setae of both head and elytra; elytral intervals with scattered setigerous punctures, setae short and punctures fine, not distinctively visible; striae with +/- evenly spaced setigerous punctures along length, both punctures and setae so fine that they are hardly visible at 50×; ventral surface with randomly scattered setigerous punctures, setae relatively dense and easily visible in lateral view.

*Fixed setae.* Elytra with two setae in interval 2, one seta posterior to apical 1/3, one seta posterior to apical 1/6.

*Luster*. Dorsal surface moderately glossy; ventral thoracic sterna and abdominal sterna moderately glossy.

*Head*. Labrum faintly emarginate, longer than wide; mentum with broad tooth; eyes convex.

*Pronotum*. Lateral margins wide, distinctively spatulate and turned up at edges, sinuate from lateral seta to base but edges rounded, not as dramatic as other *Mochtherus* species, basal angles obtuse, rounded; apical margin distinctly emarginate, large apico-lateral lobes; anterior transverse impression shallow, posterior transverse impression moderately shallow; median longitudinal impression moderately deep.

*Legs*. Tarsal claws pectinate, 3–4 denticles per claw.

*Male genitalia*. Fig. 96A–D. Length 1.04 – 1.32 mm. Phallus slightly expanding towards apex in ventral view, terminating bluntly at apical area, apex, short and broad, distinctively spatulate and positioned on the right side of phallus in ventral view; endophallus expanding and angled from mid-length, one basal lobe, one basal microtrichial field (mtf), microtrichia moderately long.

*Female genitalia*. Fig. 102A. Width 1.00 – 1.08 mm. Two lateral ensiform setae (les). One spermatheca (sp1), cylindrical and elongate tapering towards apex, length undetermined due to breakage; ring sclerite (srs) separating spermatheca duct from spermatheca, thin; one spermathecal accessory gland (sg), spermathecal gland duct (sgd) relatively long, attachment site medially on dorsal surface of ring sclerite when viewed from ventral aspect.

########## Habitat, habits, and seasonal occurrence.

The known elevational range of *M.luctuosus* is from 230 to 2000 meters. Adults are found in mixed primary and secondary forest of montane areas, as well as disturbed areas, and are crepuscular or nocturnal with most activity observed on tree trunks and deadwood at night. Several specimens were collected from the trunks of fallen trees. All other specimens were hand collected.

########## Geographical distribution.

*Mochtherusluctuosus* is known from Japan and Taiwan. For Taiwan collecting localities see Figure 103.

######### Moctherus obscurabasis sp. n.

Taxon classificationAnimaliaColeopteraCarabidae

http://zoobank.org/29ED5A42-C754-4922-9917-AD2ED7462211

**Figure 97.**
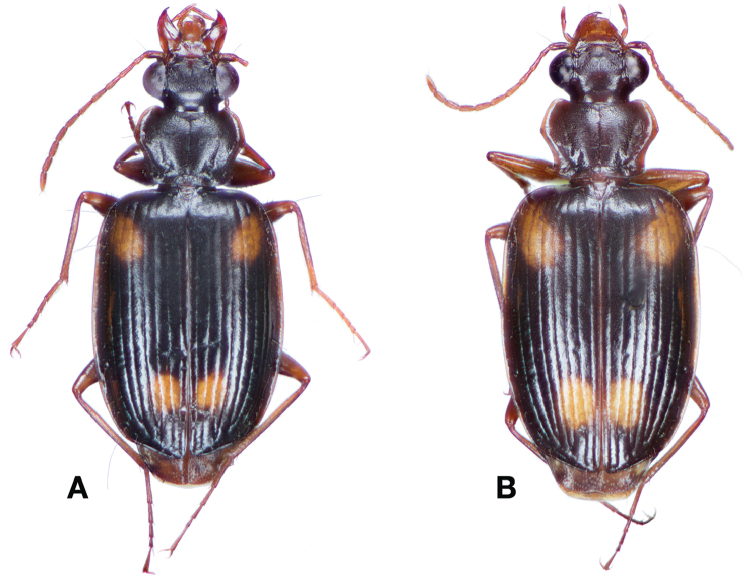
Dorsal habitus and intrapopulation variation of color pattern of *Mochtherusobscurabasis* sp. n.. **A** small elytral macula (OBL 8.17 mm) **B** large elytral macula (OBL 8.67 mm).

**Figure 98.**
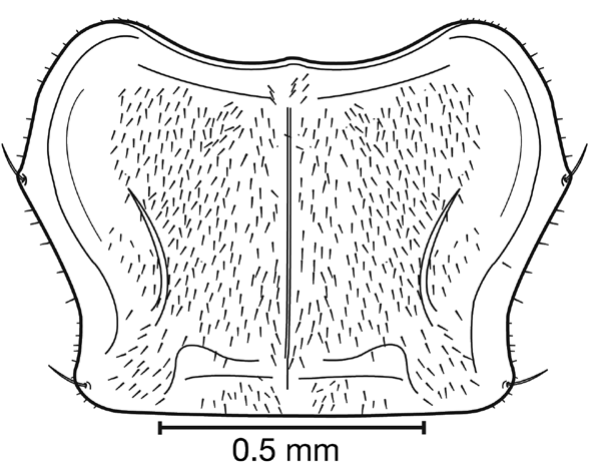
Line drawing, dorsal view, showing form and setae pattern of the pronotum of *Mochtherusobscurabasis* sp. n..

**Figure 99.**
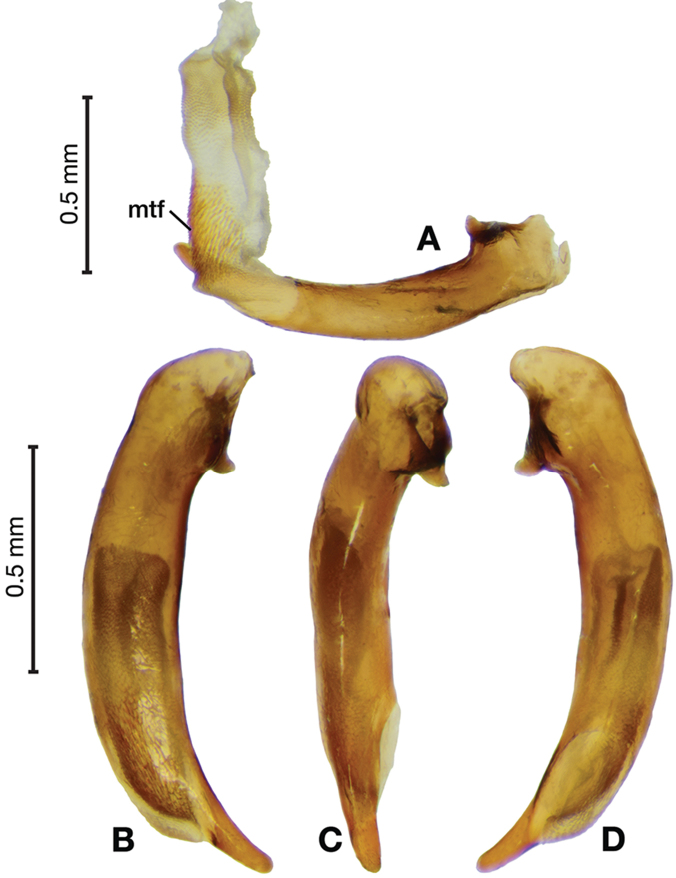
Digital images of male genitalia of *Mochtherusobscurabasis* sp. n.. **A** left lateral aspect, endophallus everted **B** right lateral aspect **C** ventral aspect **D** left lateral aspect. Legend: **mtf** microtrichial field.

Figs 97A, B , 98 , 99A–D , 102B , 103

########## Etymology.

From Latin *obscura* and *basis*, in reference to the dull appearance of the baso-medial half of the elytra due to the dense setae and relatively wide and shallow punctures.

########## Types and other material examined.

**Holotype** (male) labeled “Holotype” [circular, ringed with red]; “TAIWAN: Nantou Co./Huisun Forest Station/Area, May 23, 2012/24.0874N, 121.0301E”; “veg. on trail to waterfall/~750m, Acc. Ti-168a/Coll. W. M. Hunting”; “NCHU# 100522”. Two **paratypes** of *M.obscurabasis*: one male and one female. For further details see EH Strickland Virtual Entomology Museum Database.

########## Diagnosis.

Specimens of this species are easily distinguished from other species of *Mochtherus* by the large size (more than 7.5 mm) and the baso-medial half of the elytra with setae long and dense, punctures wide, shallow and somewhat regularly spaced, giving a distinctive dull texture.

########## Description.

OBL 8.17 – 8.67 mm. Length (n = one male, two females): head 0.84, pronotum 1.32 – 1.40, elytra 4.75 – 5.17, metepisternum 1.20 – 1.40 mm; width: head 1.76 – 1.84, pronotum 2.00 – 2.16, elytra 3.33 – 3.50, metepisternum 0.56 – 0.76 mm.

*Body proportions*. HW/HL 2.14 – 2.19; PWM/PL 1.52 – 1.55; EL/EW 1.43 – 1.55; ML/MW 1.94 – 2.19 mm.

*Color*. Fig. 97A, B. Dorsum of head rufo-brunneous to piceous, clypeus rufo-brunneous to brunneo-piceous, lighter than head, labrum brunneous, brunneo-piceous to piceous centrally; palpi and antennae brunneo-testaceous to brunneous; pronotum brunneous to brunneo-piceous, margins somewhat lighter; elytral disc brunneo-piceous to piceous, suture and margins slightly lighter, disc with four testaceous to rufo-testaceous maculaee, two anterior and two posterior, anterior macula ovoid, from stria 3 or 4 to interval 8, nearest to base at interval 7, closest to apex in interval 5 or 6, posterior macula circular to ovoid, from suture to interval 4 or 5), closest to base in interval 3 or 4, nearest to apex in interval 3 or 4; ventral surface brunneo-testaceous to brunneous, metepisternum brunneous to brunneo-piceous, darker; legs brunneous, light.

*Microsculpture*. Dorsum of head with mesh pattern somewhat granulate, isodiametric, labrum with sculpticells shallow, almost isodiametric; pronotum with microsculpture transverse; elytra with sculpticells transverse, most easily observed in apical half; metepisternum with microsculpture somewhat granulate, almost isodiametric to transverse; other ventral surfaces with microsculpture transverse.

*Macrosculpture and pilosity*. Dorsum of head faintly and longitudinally rugulose, with scattered setigerous punctures, setae short and fine between eyes, somewhat longer behind eye, clypeus relatively smooth, with several scattered setigerous punctures, labrum smooth, with several short setae in apical half; scrobe of mandible with few setae near base; pronotum faintly rugulose transversely, punctate and densely setose; elytra with intervals punctate and setose, baso-medial half with setae longer and more dense, punctures wide, shallow, dense and somewhat regularly spaced, giving distinctive texture, striae faintly punctate, setae hardly visible at 50×; ventral surface with randomly scattered setigerous punctures, setae relatively dense and easily visible in lateral view.

*Fixed setae.* Elytra with two setae in interval 2, one seta just back from mid-length, one seta in apical 1/6.

*Luster*. Dorsal surface moderately dull with basal third of elytra dull; ventral thoracic sterna and abdominal sterna moderately glossy.

*Head*. Mandibles with wide base, short; labrum longer than wide, rectangular; mentum with shallow tooth; eyes convex.

*Pronotum*. Fig. 98. Anterior transverse impression very shallow; posterior transverse impression moderately deep; median longitudinal impression moderately deep; disc convex, apical margin emarginate, basal angles obtuse; lateral margins broadly rounded in apical portion, markedly sinuate from lateral seta to base.

*Elytra*. Lateral margin smooth, slightly rounded along length.

*Legs*. Tarsal claws pectinate, 4–5 denticles per claw, apical denticles rather long.

*Male genitalia*. Fig. 99A–D. Length 1.2 mm. Phallus relatively narrow, uniform width along length, apex somewhat elongate and narrow, curved along length to follow contour of phallus; endophallus with width relatively uniform along length, right angled lobe at base, one basal microtrichial field (mtf).

**Figure 100.**
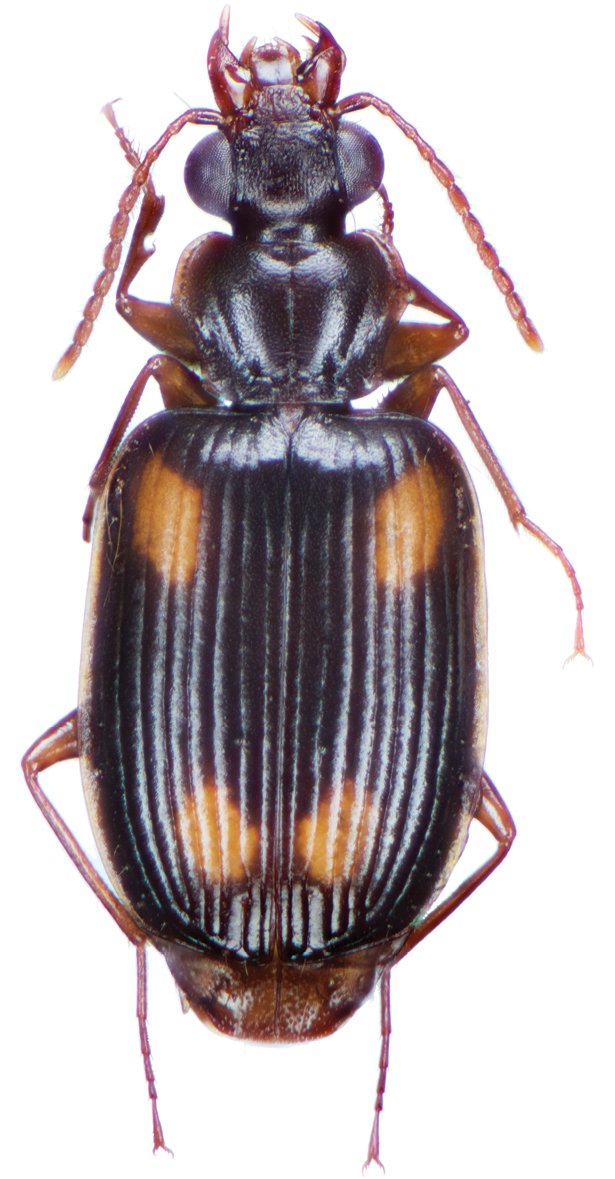
Dorsal habitus and color pattern of *Mochtherustetraspilotus* (MacLeay). (OBL 6.48 mm).

**Figure 101.**
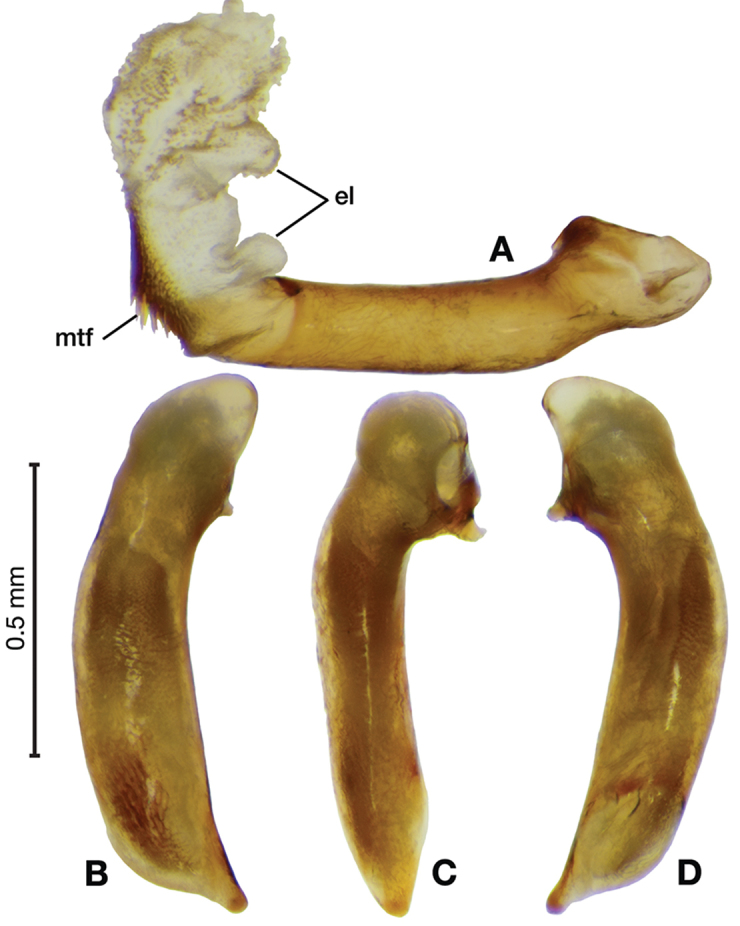
Digital images of male genitalia of *Mochtherustetraspilotus* (MacLeay). **A** left lateral aspect, endophallus everted **B** right lateral aspect **C** ventral aspect **D** left lateral aspect. Legend: **el**, endophallic lobe; **mtf** microtrichial field.

**Figure 102.**
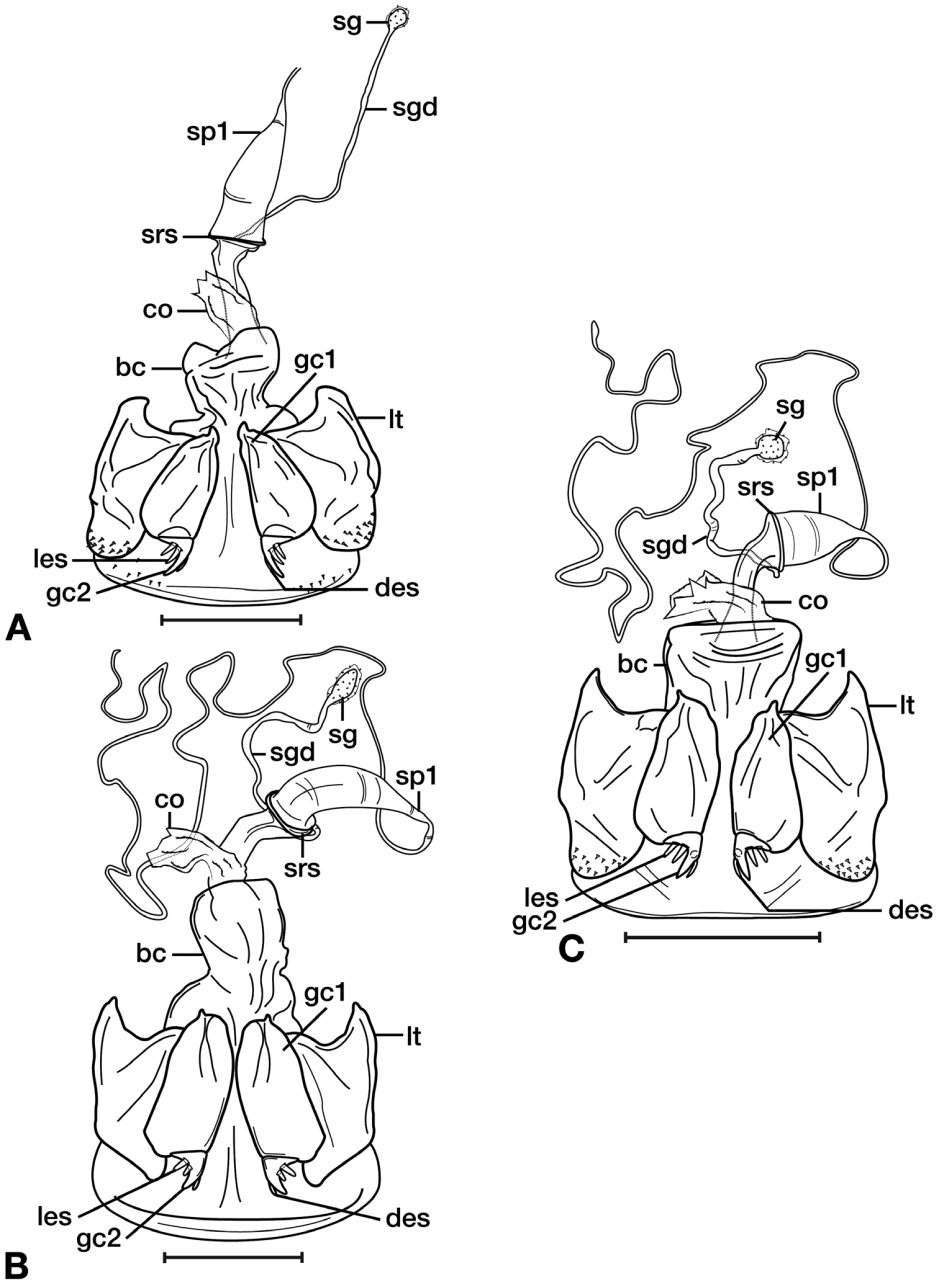
Line drawings of the female reproductive tract of species of the genus *Mochtherus* Schmidt-Goebel, known from Taiwan, ventral aspect. **A***M.luctuosus* Putzeys **B***M.obscurabasis* sp. n. **C***M.tetraspilotus* (MacLeay). Legend: **bc** bursa copulatrix; **co** common oviduct; **des** dorsal ensiform setae; **div** diverticulum; **gc1** gonocoxite 1; **gc2** gonocoxite 2; **les** lateral ensiform setae; **lt** lateral tergite; **sg** spermathecal gland; **sgd** spermathecal gland duct; **sp1** spermatheca 1; **srs** spermathecal ring sclerite. Scale bars: 0.5 mm.

*Female genitalia*. Fig. 102B. Width 0.96 mm. Two lateral ensiform setae (les), basal seta longer than apical seta. One spermatheca (sp1), cylindrical and elongate tapering towards apex, with markedly elongate apical end; ring sclerite (srs) separating spermatheca duct from spermatheca; one spermathecal accessory gland (sg), spermathecal gland duct (sgd) attachment site on right side of ring sclerite when viewed from ventral aspect.

########## Habitat, habits, and seasonal occurrence.

The known elevational range of *M.obscurabasis* is from 750 to 1300 meters. Little is known about the habits of this species however, the single specimen collected was found in secondary, mixed forest, on the trunk of a live tree. The tree was on a dirt path and the time was approximately 9:00 pm. Specimens have been collected in May and July and the only known method of collecting is by hand.

########## Geographical distribution.

**Figure 103.**
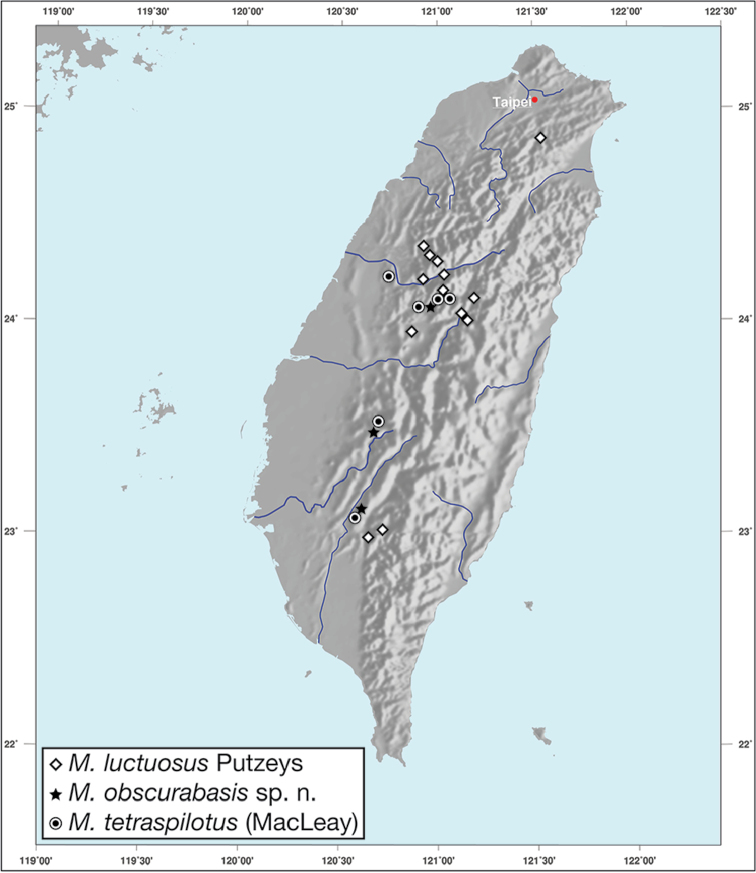
Map showing known localities for species of the genus *Mochtherus* Schmidt-Goebel, in Taiwan.

*Mochtherusobscurabasis* is known only from Taiwan. See Figure 103.

######### Mochtherus tetraspilotus

Taxon classificationAnimaliaColeopteraCarabidae

(MacLeay)

Figs 100 , 101A–D , 102C , 103

Dromius tetraspilotus MacLeay, 1825: 25.
Thyreopterus tetrasemus Dejan, 1831: 448.
Mochtherus angulatus Schmidt-Goebel, 1846: 76; Schaum 1860:187; Redtenbacher 1868: 7; Andrewes 1933: 461.
Panagaeus retractus Walker, 1858: 203.
Cyrtopterus quadrinolatus Motschulsky, 1861: 106.
Mochtherus tetraspilotus MacLeay: Chaudoir, 1869; Bates 1886: 203; 1892: 412; Andrewes 1919: 163; 164, 189; Andrewes 1923: 45; Andrewes 1927: 12; Andrewes 1929: 313; Andrewes 1930; 336; Andrewes 1933: 2; Heller 1919: 273; Csiki 1932: 1382; Shibata 1962: 18; Jedlička 1963: 353; Kisrchenhofer 1994: 1048; Lorenz 2005: 460.

########## Types and other material examined.

62 specimens of *M.tetraspilotus*: 28 males and 34 females. For further details see EH Strickland Virtual Entomology Museum Database.

########## Type locality.

Java.

########## Diagnosis.

Specimens of this species are easily distinguished from other species of *Mochtherus* by the smaller size (less than 7.5 mm) and baso-medial half of elytra with setae and associated punctures not distinctively differing from remainder of disc.

########## Redescription.

OBL 6.17 – 7.00 mm. Length (n = ten males, ten females): head 0.72 – 0.76, pronotum 1.08 – 1.20, elytra 3.67 – 4.17, metepisternum 0.84 – 0.96 mm; width: head 1.36 – 1.52, pronotum 1.60 – 1.88, elytra 2.67 – 3.17, metepisternum 0.52 – 0.56 mm.

*Body proportions*. HW/HL 1.89 – 2.11; PWM/PL 1.41 – 1.63; EL/EW 1.25 – 1.44; ML/MW 1.40 – 171 mm.

*Color*. Fig. 100. Dorsum of head rufo-brunneous to piceous, clypeus rufo-brunneous to brunneo-piceous, lighter than head, labrum brunneous, brunneo-piceous to piceous centrally; palpi and antennae brunneo-testaceous to brunneous; pronotum brunneous to brunneo-piceous, margins somewhat lighter; elytral disc brunneo-piceous to piceous, suture and margins brunneous to brunneo-piceous, somewhat lighter than disc, with four testaceous to rufo-testaceous maculae, two anterior and two posterior, anterior macula ovoid, from stria 4 (sometimes diffusely into interval 4) to interval 8, nearest to base at interval 7, closest to apex in interval 5 or 6, posterior macula circular to ovoid, from stria 1 to stria 5 (sometimes stria 4), closest to base in interval 4, nearest to apex in interval 3–4; ventral surface of head, pronotum and thoracic sclerites brunneous, abdominal sclerites testaceous to brunneo-testaceous; legs testaceous to brunneous.

*Microsculpture*. Dorsum of head with mesh pattern somewhat granulated, isodiametric, labrum with sculpticells shallow, stretched longitudinally; pronotum with microsculpture transverse; elytra with sculpticells slightly wider than long, almost isodiametric; metepisternum with microsculpture almost isodiametric to isodiametric; other ventral surfaces with microsculpture transverse.

*Macrosculpture and pilosity*. Dorsum of head faintly and longitudinally rugulose, with scattered setigerous punctures, setae short and fine, clypeus rugulose, with several scattered setigerous punctures, labrum relatively smooth, with several short setae in apical half; scrobe of mandible setose at base; pronotum faintly rugulose transversely, punctate and densely setose; elytra with intervals punctate and setose, setae longer and more dense medially in basal half, striae faintly punctate, setae hardly visible at 50×; ventral surface with randomly scattered setigerous punctures, setae relatively dense and easily visible in lateral view.

*Fixed setae.* Elytra with two setae in interval 2, one seta just back from mid-length, one seta in apical 1/6.

*Luster*. Dorsal surface moderately dull; ventral thoracic sterna and abdominal sterna moderately glossy.

*Head*. Mandibles with wide base, short, mostly concealed by labrum in resting position; labrum longer than wide, rectangular; mentum with shallow tooth; eyes convex.

*Pronotum*. Anterior transverse impression very shallow; posterior transverse impression moderately deep; median longitudinal impression moderately deep; Disc convex, apical edge slightly emarginate, basal angles obtuse; lateral margins broadly rounded in apical portion, markedly sinuate from lateral seta to base.

*Elytra*. Lateral margin smooth, parallel along length.

*Legs*. Tarsal claws pectinate, three to four denticles per claw, apical denticles rather long.

*Male genitalia*. Fig. 101A–D. Length 0.84 – 0.92 mm. Phallus uniform width along length, apex bluntly rounded and somewhat triangular in ventral view, more constricted and pointed in lateral view; endophallus relatively short, one basal microtrichial field (mtf), microtrichia distinctively long, two distinctive endophallic lobes (el), one near base and one medially.

*Female genitalia*. Fig. 102C. Width 0.84 – 0.88 mm. Two lateral ensiform setae (les), rather long. One spermatheca (sp1), cylindrical, elongate and narrowing towards apex with markedly elongate apical end; ring sclerite (srs) separating spermatheca duct from spermatheca; one spermathecal accessory gland (sg), spermathecal gland duct (sgd) attachment site on right side of ring sclerite when viewed from ventral aspect.

########## Habitat, habits, and seasonal occurrence.

The known elevational range of *M.tetraspilotus* in Taiwan is from 480 to 1300 meters. Only one specimen is known from over 725 meters. Adults are crepuscular or nocturnal and are found in mixed primary and secondary forest of montane areas, as well as disturbed areas. The vast majority of known specimens (54) were collected within a week of each other in 2013 on two nights in late November and early December. They were collected from the underside of fallen trees and deadwood. A single specimen is known from July and another from September. Known methods of collecting this species are sweep netting and hand collecting.

########## Geographical distribution.

*Mochtherustetraspilotus* is widespread and known from Japan, Burma, Philippines, Laos, Borneo, Java, Ceylon, India, USA, and Taiwan. For additional Taiwan collecting localities, see Figure 103.

######## Genus *Pericalus* W. S. MacLeay

######### Subgenus Pericalus

Taxon classificationAnimaliaColeopteraCarabidae

s. str.

Pericalus MacLeay, 1825: 15; Schmidt-Goebel 1846: 85; Lacordaire 1854: 147; Schaum 1860: 190; Chaudoir 1861: 123; Jedlička 1963: 373; Baehr 2000: 33; Lorenz 2005: 455; Fedorenko 2017: 303; Hongliang and Hongbin 2018: 19.

########## Type species.

*Pericaluscicindeloides* MacLeay, 1825 (monobasic).

######### Pericallus

Taxon classificationAnimaliaColeopteraCarabidae

Gemminger & Harold, 1868: 154; Bates 1869: 71.

########## Type locality.

Java.

######### Pericalus formosanus

Taxon classificationAnimaliaColeopteraCarabidae

Dupuis stat. resurr.

**Figure 104.**
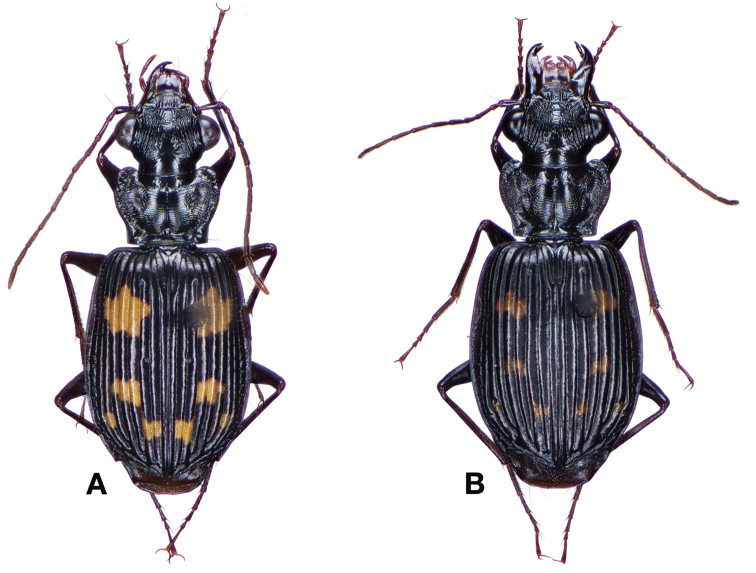
Dorsal habitus and intrapopulation variation of color pattern of *Pericalusformosanus* Dupuis. **A** larger elytral macula (common) (OBL 11.02 mm). **B** smaller elytral macula (OBL 8.02 mm).

**Figure 105.**
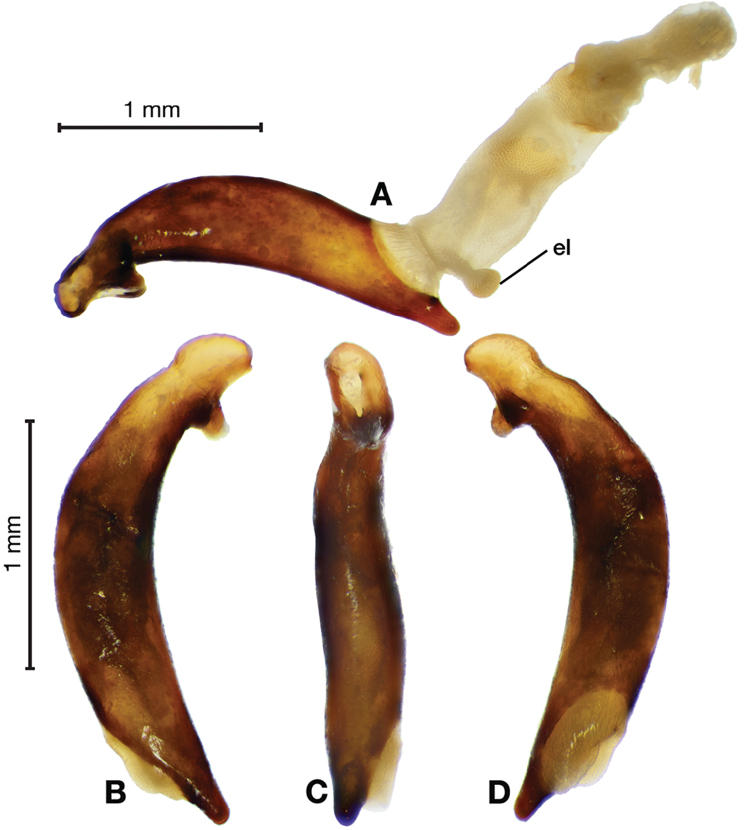
Digital images of male genitalia of *Pericalusformosanus* Dupuis. **A** left lateral aspect, endophallus everted **B** right lateral aspect **C** ventral aspect **D** left lateral aspect. Legend: **el** endophallic lobe.

**Figure 106.**
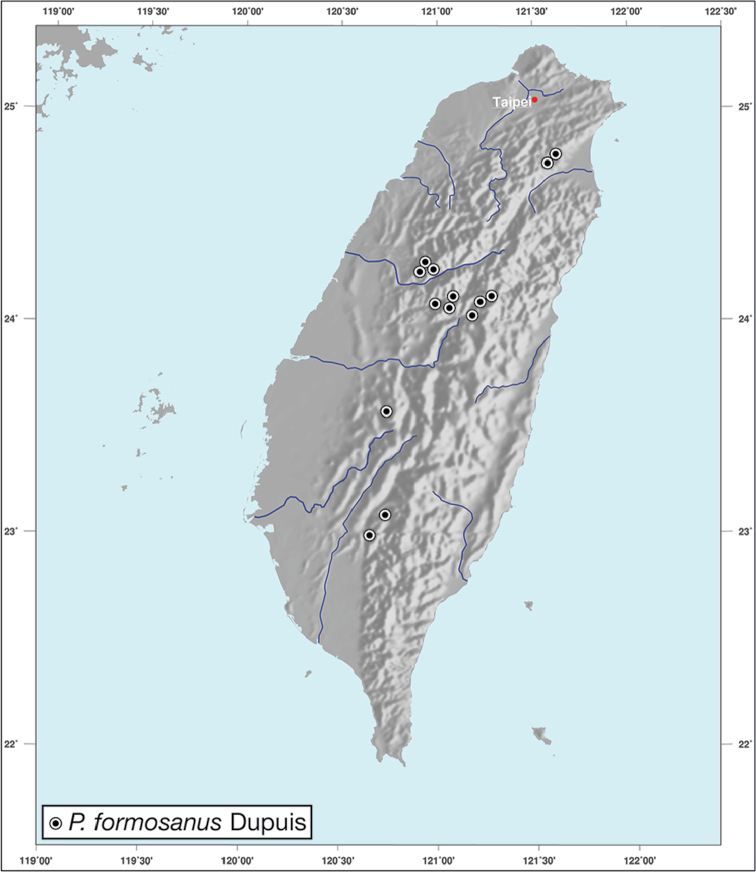
Map showing known localities for species of the genus *Pericalus* MacLeay, in Taiwan.

Figs 104A, B , 105A–D , 106 , 110A

Pericalus formosanus Dupuis, 1913: 83; Csiki 1932: 1369; Jedlička 1963: 379; Fedorenko 2017: 311.
Pericalus ornatus formosanus Dupuis: Shi and Liang 2018: 36.

########## Types and other material examined.

**Holotype** (male) labeled “Chip Chip/ II Formosa/Sauter 07-09”; “TYPUS” [rectangular, red paper]; “Pericalusformosanus Dupuis/Dupuis det. ”; “Syntypus”; “DEI Coleoptera/# 200415”. Five **paratypes** and 151 other specimens of *P.formosanus*: 75 males and 76 females. For further details see EH Strickland Virtual Entomology Museum Database.

########## Type locality.

Taiwan. Kaoshiung County, “Hoozan” = Fengshan City.

########## Taxonomic notes.

In a recent paper Shi and Liang (2018) considered *P.formosanus* to be a subspecies of *Pericalusornatus* Schmidt-Goebel, 1846. While these taxa are similar, we have deduced that there are sufficient differences to maintain the species status of *P.formosanus*. Along with differences in the hind angles of the elytra, and number of discal setae (one in *P.formosanus* and two in *P.ornatus*), there are slight but consistent differences in the genitalic characteristics. Females of *P.formosanus* have a spermathecal gland duct that is longer and a spermathecal gland (Fig. 110B) that is larger than seen in specimens of *P.ornatus*. Males of *P.formosanus* have a phallus with ostium more dorsally situated than in members of *P.ornatus* and an endophallus that is obviously narrower in form and has only one prominent endophallic lobe (Fig. 108A), located more basally than the most basal endophallic lobe of specimens of *P.ornatus*.

Shi and Liang observed that Fedorenko (2017), recorded *P.formosanus* from Vietnam based on misidentification of *P.acutidens*. After examining his images of the everted endophallus, it is clear that it was a mistaken identity. *Pericalusformosanus* is restricted to Taiwan and allopatric with all other species of *Pericalus*.

########## Diagnosis.

Specimens of this species are easily distinguished from other Taiwanese pericalines by having smooth tarsal claws, two pairs of supraorbital setae, two pairs of latero-marginal setae on the pronotum and a black dorsal coloration with eight maculae on the disc of the elytra.

########## Redescription.

OBL 7.67 – 11.33 mm. Length (n = ten males, ten females): head 0.92 – 1.24, pronotum 1.28 – 2.12, elytra 4.33 – 6.33, metepisternum 1.04 – 1.40 mm; width: head 2.14 – 3.10, pronotum 3.33 – 4.67, elytra 3.33 – 4.67, metepisternum 0.56 – 0.84 mm.

*Body proportions*. HW/HL 2.24 – 2.58; PWM/PL 1.45 – 1.58; EL/EW 1.25 – 1.46; ML/MW 1.40 – 1.94.

*Color*. Fig. 104A, B. Various. Dorsum of head piceous, clypeus piceous with rufo-brunneous apical edge, antennae and palpi rufo-brunneous, scape sometimes darker; pronotum piceous; elytral disc piceous, with eight testaceous maculae, two in basal 1/3, two near mid-length, and four in apical 1/3, basal macula somewhat dentate, from interval 3 or 4 to interval 6 or 7, always closest to base in interval 5 and closest to apex in interval 4, mid-length macula from interval 4 to interval 5 or 6, apical macula with small patch from interval 2 to 3 and small patch in interval 7; ventral surface rufo-piceous to piceous; legs rufo-brunneous, tibia darker.

*Microsculpture.* Head with microsculpture almost isodiametric to isodiametric; pronotum with microsculpture shallow, somewhat transverse to transverse; elytra with sculpticells transverse, single row of isodiametric cells down center of each stria; ventral surface with transverse to almost isodiameteric microsculpture.

*Macrosculpture and pilosity*. Dorsum of head and base of clypeus rugulose, surface with very fine, scattered, setigerous punctures, hardly visible at 50×; pronotum disc faintly rugulose centrally, more so along lateral margins, surface with very fine, scattered, setigerous punctures; elytral intervals convex, interval 7 slightly more raised than others in apical 1/3, entire dorsal surface with fine, scattered setigerous punctures, punctures hardly visible but setae easily viewed in lateral view at 50×, striae impunctate; ventral surface of head rugulose to gula suture, remaining ventral surface with randomly scattered setigerous punctures.

*Fixed setae.* Two pairs of supraorbital setae; clypeus with two long, lateral setae; labrum with six setae along apical margin; one pair of suborbital setae; pronotum with two pairs of setae, one at base of lateral margin, one on lateral margin at pronotum max width; 24 – 25 lateral (umbilical) setae in interval 9; elytra with interval 3 with one seta in basal 1/6 (See also, variation); ventral surface with two setae on each of abdominal sterna III to VI; four setae along apical margin of sternum VII.

########## Variation.

of 151 specimens observed only one was observed to have two setae in interval 3, the typical apical setae and one at mid-length; see Fig. 104A (two setae) vs. Fig. 104B (one seta).

*Luster*. Dorsal surface moderately glossy; ventral surface glossy.

*Head*. Mandibles long, left mandible with distinctive notch on inside and dorsal surface in apical 1/3; labrum deeply bilobed, mentum without tooth; eyes large, convex; palpi cylindrical and elongate and with fine setae.

*Pronotum*. Lateral margins explanate, with margins curved slightly upwards, anterior transverse impression moderately shallow, posterior transverse impression deep, median longitudinal impression moderately deep, apico-lateral margins broadly and distinctly lobed, posterio-lateral margins sinuate, right-angled.

*Elytra*. Hind angles slightly sinuate, apex of lateral margin with distinctly sharp edge, pointed.

*Hind wings*. Macropterous.

*Legs*. Tarsal claws smooth, rather long and slender, males with adhesive vestiture ventrally, two rows squamo-setae on tarsomeres 1–3 of fore-leg.

*Male genitalia*. Fig. 105A–D. Length 1.80 – 2.06 mm. Ostium catopic, open slightly more on the left side. Phallus cylindrical, widest at mid-length, apex short, rounded at tip; endophallus relatively long and narrow along length, with single distinctive lobe (el) at base.

*Female genitalia*. Fig. 110A. Width 1.15 – 1.30 mm. Gonocoxite 2 (gc2) distinctively long and narrow, relatively uniform in width along length; three lateral ensiform setae spaced widely apart (les), one dorsal ensiform seta. Sensory furrow, furrow pegs, and associated nematiform setae not observed. One spermatheca present (sp1), elongate and cylindrical, expanding slightly in apical half; one spermathecal accessory gland (sg), associated spermathecal gland duct (sgd), with attachment site just before widening of spermatheca.

########## Habitat, habits, and seasonal occurrence.

The known elevational range of *P.formosanus* is from 500 to 2095 meters with the majority of adults being collected between 1800 and 2000 meters. Adults of this species are crepuscular and are found in mixed primary and secondary forest of montane areas, typically in moist areas. They can be found inside and under deadwood during the day and on deadwood at night. Specimens are easily captured as they do not run or fly when illuminated. Specimens have been collected throughout the year with most collected from May to July. Methods of collecting include light trap on ground, u.v. light sheet, pitfall trap, and hand collecting.

########## Geographical distribution.

*Pericalusformosanus* is known only from Taiwan. See Figure 106.

######### Genus Serrimargo

Taxon classificationAnimaliaColeopteraCarabidae

Chaudoir

Serrimargo Chaudoir, 1869: 134; Andrewes 1939: 137; Jedlička 1963: 369; Lorenz 2005: 463; Fedorenko 2018: 1.
Peripristus Chaudoir, 1869: 135; Jedlička 1963: 370; Kirschenhofer 1994: 1048; Lorenz 2005: 463.

########## Type species.

*Thyreopterusguttiger* Schaum, 1860, by subsequent designation (Andrewes 1939).

########## Type locality.

India

######### Serrimargo schenklingi

Taxon classificationAnimaliaColeopteraCarabidae

(Dupuis)

**Figure 107.**
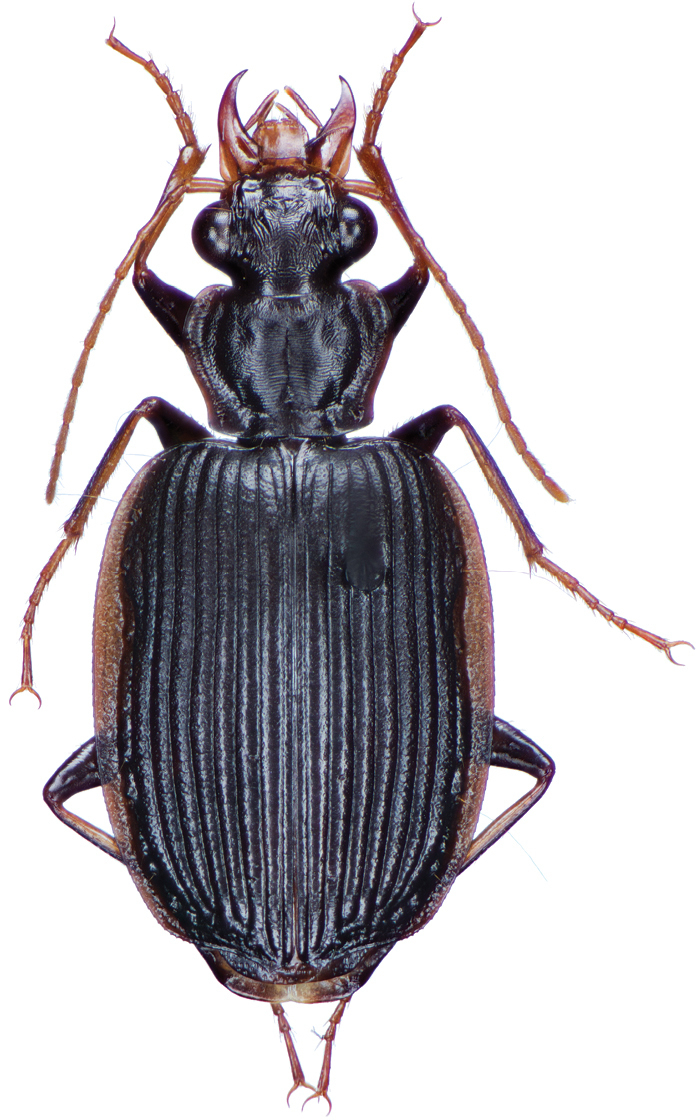
Dorsal habitus and color pattern of *Serrimargoschenklingi* (Dupuis). (OBL 12.00 mm).

**Figure 108.**
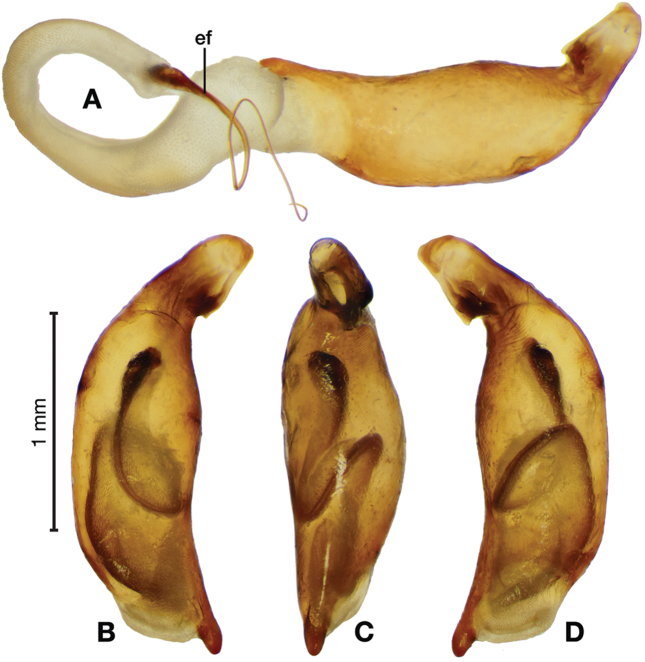
Digital images of male genitalia of *Serrimargoschenklingi* (Dupuis). **A** left lateral aspect, endophallus everted **B** right lateral aspect **C** ventral aspect **D** left lateral aspect. Legend: **ef** endophallic flagellum.

**Figure 109.**
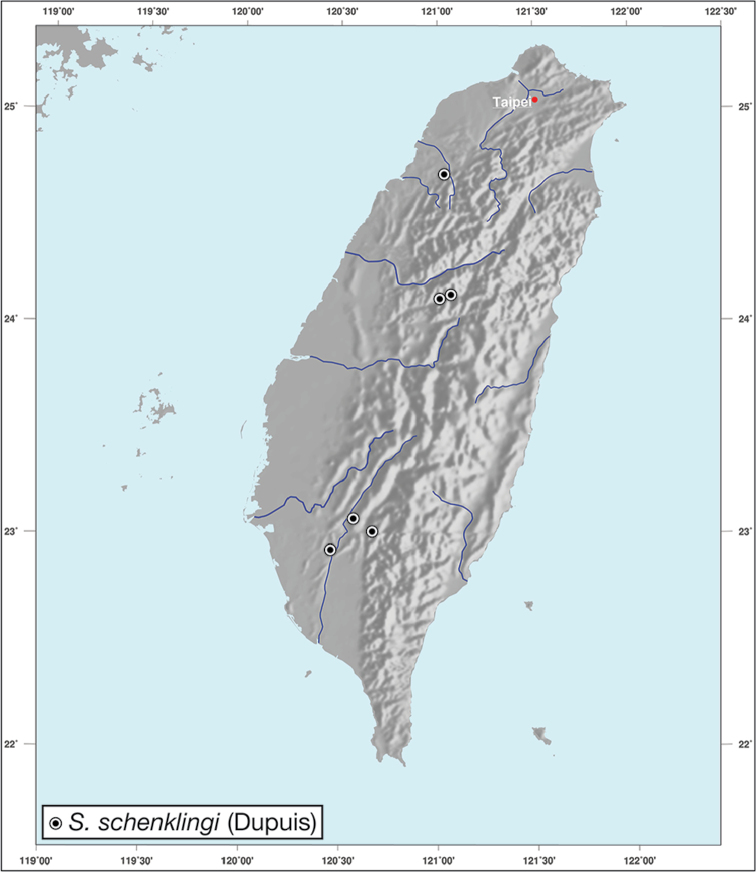
Map showing known localities for species of the genus *Serrimargo* Chaudoir, in Taiwan.

**Figure 110.**
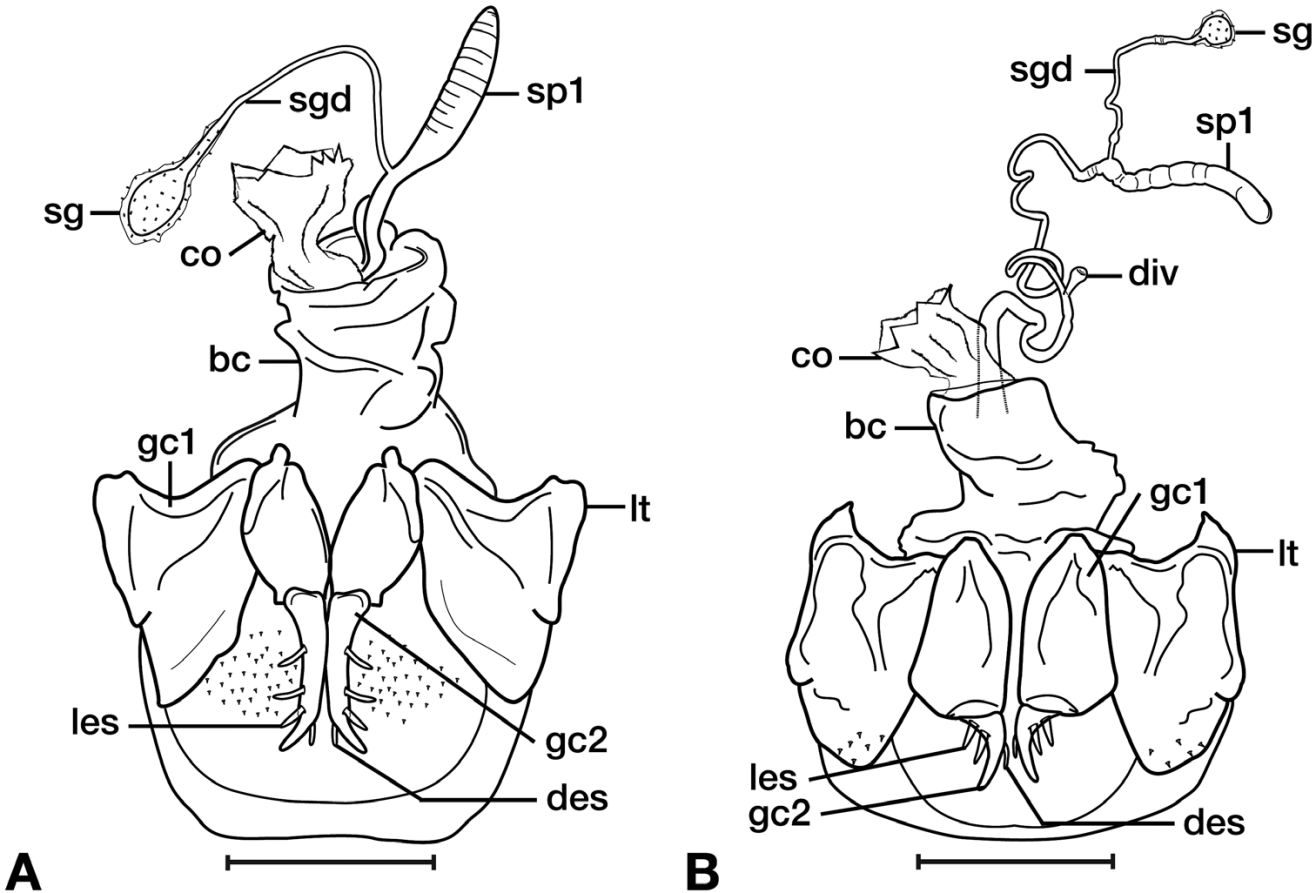
Line drawings of the female reproductive tract, ventral aspect, of **A***Pericalusformosanus* Dupuis **B***Serrimargoschenklingi* (Dupuis). Legend: **bc** bursa copulatrix; **co** common oviduct; **des** dorsal ensiform setae; **div** diverticulum; **gc1** gonocoxite 1; **gc2** gonocoxite 2; **les** lateral ensiform setae; **lt** lateral tergite; **sg** spermathecal gland; **sgd** spermathecal gland duct; **sp1** spermatheca 1. Scale bars: 0.5 mm.

Figs 107 , 108A–D , 109 , 110B

Thyreopterus (Peripristus) schenklingi Dupuis, 1912: 288.
Peripristus ater (var. schenklingi) Dupuis: Csiki 1932: 1356; Jedlička 1963: 370.
Peripristus ater schenklingi Dupuis: Lorenz 2005: 463.

########## Types and other material examined.

**Holotype** (male) labeled “Kosempo/Formosa/Sauter VII 09”; “TYPUS” [rectangular, red paper]; “DUPUIS DET.”; “Thyreopterus/schenklingi/Dupuis”[handwritten]; “DEI Muncheberg/Col-03827”; “NCHU#/ 00507”. One **paratype** and 44 other specimens of *S.schenklingi*: 28 males and 19 females. For further details see EH Strickland Virtual Entomology Museum Database.

########## Type locality.

Taiwan. “Kosempo” = Chia-hsien, Kaohshiung City.

########## Taxonomic notes.

Fedorenko (2018), recently raised *S.schenklingi* to species status. After examining the types of *S.schenklingi* and comparing them with material of *S.ater*, this is indeed a valid species. After dissecting both male and female examples of recognized *Peripristus* species and some *Serrimargo* that were available, it was clear that they were structurally very similar and closely related.

########## Diagnosis.

Specimens of this species are easily distinguished from other Taiwanese pericalines by the smooth tarsal claws and the distinctively black and granulate surface of the elytra.

########## Redescription.

OBL 9.50 – 13.33 mm. Length (ten males, ten females): head 1.00 – 1.28, pronotum 1.50 – 2.04, elytra 5.67 – 7.42, metepisternum 1.20 – 1.68 mm; width: head 1.08 – 2.80, pronotum 2.24 – 3.04, elytra 4.17 – 5.92, metepisternum 0.72 – 0.96 mm.

*Body proportions*. HW/HL 1.91 – 2.26; PWM/PL 1.42 – 1.57; EL/EW 1.18 – 1.37; ML/MW 1.39 – 1.86 mm.

*Color*. Fig. 107. Dorsum of head piceous, clypeus rufo-brunneous, darker centrally, labrum brunneo-testaceous to rufo-brunneous, antennae and palpi brunneo-testaceous to rufo-brunneous; pronotum piceous, margins brunneous to brunneo-piceous; elytral disc piceous, margins brunneous to brunneo-piceous; ventral surface of head rufous to rufo-piceous, gula lighter; ventral surface of pronotum, metepisternum and metasternum rufo-piceous; epipleuron of elytra and abdominal sterna lighter, brunneo-testaceous to brunneous; legs with coxa testaceous to brunneo-testaceous, femora rufo-brunneous to piceous, darker towards apex, tibia with ventral surface testaceous, dorsal surface darker, rufo-piceous to piceous.

*Microsculpture.* Head with microsculpture almost isodiametric to isodiametric; pronotum with microsculpture transverse; elytra with sculpticells isodiametric, somewhat granulate;d ventral surface with sculpticells of elytra epipleuron almost isodiametric to isodiametric, all other surfaces with transverse to almost isodiameteric microsculpture.

*Macrosculpture*. Dorsum of head rugulose, surface with very fine, scattered, setigerous punctures, hardly visible at 50×; pronotum disc transversely rugulose, with single, shallow depression medially on each side, lateral margins shallowly rugulose, surface with very fine, scattered, setigerous punctures; elytra with lateral margins distinctly explanate, finely serrate along edge, intervals convex, in some specimens intervals slightly pointed, more so in apical half, intervals slightly pitted in appearance to more rugulose laterally, entire surface with very fine, scattered, setigerous punctures; ventral surface of head rugulose to gula suture; abdominal segments 4, 5 and 6 with a few additional deep punctures between typical fixed setae; remaining ventral surface with fine, randomly scattered setigerous punctures.

*Fixed setae.* Two pairs of supraorbital setae; clypeus with two long, lateral setae; labrum with six setae along apical margin; one pair of suborbital setae; pronotum with one pair of setae at base of lateral margin; 16–17 lateral (umbilical) setae in interval 9; elytra with interval 3 with two setae, placement slightly variable, one seta at approximately 1/3 from apex, next half way between first setae and apex; ventral surface with two setae on each of abdominal sterna III to VI, four setae along apical margin of sternum VII.

*Luster*. Dorsal surface moderately dull; ventral surface moderately glossy.

*Head*. Mandibles long and narrow, longer in some males; labrum rectangular, longer than wide, some specimens with very slightly emarginate apical edge; mentum with tooth; eyes large, convex; palpi cylindrical and elongate and with fine setae.

*Pronotum*. Lateral margins explanate, with margins curved slightly upwards; anterior transverse impression moderately shallow; posterior transverse impression deep; median longitudinal impression moderately deep; apico-lateral margins broadly and distinctly lobed, posterio-lateral margins sinuate, right-angled.

*Elytra*. Broadly rounded, hind angles slightly sinuate, lateral margins serrated.

*Hind wings*. Macropterous.

*Legs*. Tarsal claws smooth, males with adhesive vestiture ventrally, two rows of squamo-setae on tarsomeres 1–3 of fore-leg.

*Male genitalia*. Fig. 108A–D. Length 1.80 – 1.96 mm. Ostium left pleuropic. Phallus cylindrical, distinctively wide, apex short, rounded at tip; endophallus long and narrowing from base to apex, curled along length, one long and curled flagellum (ef) at apex of endophallus.

*Female genitalia*. Fig. 110B. Width 1.16 – 1.28 mm. Gonocoxite 2 (gc2) long and narrow, narrowing from base to apex; two lateral ensiform setae spaced apart (les), one dorsal ensiform seta. Sensory furrow, furrow pegs and associated nematiform setae not observed. One spermatheca present (sp1), elongate and cylindrical, associated duct (sd) relatively long, with distinctive diverticulum (div) at approximately 1/3 from spermatheca duct base; one spermathecal accessory gland (sg), associated spermathecal gland duct (sgd), with attachment site near base of spermatheca.

########## Habitat, habits, and seasonal occurrence.

The known elevational range of *S.schenklingi* is from 584 to 750 meters. Adults of this species which are crepuscular are found in mixed primary and secondary forest of montane areas, and those that are crepuscular or nocturnal are typically found in moist areas on deadwood. Most collected specimens (31 individuals) came from two large dead trees that were in close proximity. The trees were lying down on a hill. One side received no light during the day and was covered, in places, by a mat of white fungus. Individuals were aggregated on these white mats. When disturbed with light, they would quickly move to find darkness. Specimens have been collected from April to December with most specimens collected October and December. The only know method of collection is by hand.

########## Geographical distribution.

*Serrimargoschenklingi* is known from south China, Vietnam, and Taiwan. For Taiwan collecting localities see Figure 109.

## Supplementary Material

XML Treatment for Amphimenes

XML Treatment for Amphimenes asahinai

XML Treatment for Amphimenes absensacidus

XML Treatment for Amphimenes beichatiensis

XML Treatment for Amphimenes carinacaulis

XML Treatment for Bellavalentis

XML Treatment for Bellavalentis kuzugamii

XML Treatment for Brachichila

XML Treatment for Brachichila hypocrita

XML Treatment for Catascopus

XML Treatment for Catascopus (s. str.) asaharti

XML Treatment for Catascopus (s. str.) ignicinctus

XML Treatment for Catascopus (s. str.) sauteri

XML Treatment for Catascopus (s. str.) viridiorchis

XML Treatment for Catascopoides

XML Treatment for Catascopus (Catascopoides) horni

XML Treatment for Coptodera

XML Treatment for Coptoderina

XML Treatment for Coptodera (Coptoderina) chaudoiri

XML Treatment for Coptodera (Coptoderina) eluta

XML Treatment for Coptodera (Coptoderina) japonica

XML Treatment for Coptodera (Coptoderina) maculata

XML Treatment for Coptodera (Coptoderina) marginata

XML Treatment for Coptodera (Coptoderina) occulta

XML Treatment for Coptodera (Coptoderina) proksi

XML Treatment for Coptodera (Coptoderina) taiwana

XML Treatment for Dolichoctis

XML Treatment for Dolichoctis badiadorsis

XML Treatment for Dolichoctis dilatata

XML Treatment for Dolichoctis rotundata

XML Treatment for Dolichoctis taiwanensis

XML Treatment for Formosiella

XML Treatment for Pseudomenarus

XML Treatment for Formosiella brunnea

XML Treatment for Formosiella flavomaculata

XML Treatment for Holcoderus

XML Treatment for Wagneria

XML Treatment for Holcoderus formosanus

XML Treatment for Horniulus

XML Treatment for Horniulus andrewesi

XML Treatment for Lioptera

XML Treatment for Lioptera erotyloides

XML Treatment for Miscelus

XML Treatment for Miscelus javanus

XML Treatment for Mochtherus

XML Treatment for Mochtherus luctuosus

XML Treatment for Moctherus obscurabasis

XML Treatment for Mochtherus tetraspilotus

XML Treatment for Pericalus

XML Treatment for Pericallus

XML Treatment for Pericalus formosanus

XML Treatment for Serrimargo

XML Treatment for Serrimargo schenklingi

## Footnotes

WH became fascinated with the Taiwan insect fauna during a 3-year visit, in the early 2000’s: experiences in the country, observing the huge diversity of life during that time inspired me to learn more so I visited the local National Chung Hsing University to see if there might be some learning opportunities for an undergraduate foreigner, such as myself. There I met friend, supervisor, and co-author, Dr Man Miao Yang. She provided me with literature, specimens, and the guidance to gain a cursory knowledge of the insect orders. I learned two important things during that time: I wanted to choose entomology as a field of study and my Mandarin was nowhere where it needed to be to pursue an undergraduate degree in Taiwan. I returned from Taiwan and began learning about carabid beetles from friend and mentor, Dr George E Ball (University of Alberta, Strickland Museum). Over the next several years I developed a particular interest in the lebiine ground beetles and a desire to return to the place where my entomological interests began. This provided the rationale for returning to Taiwan and taking on a review of the pericaline lebiines.
